# Discovery of an ancient MHC category with both class I and class II features

**DOI:** 10.1073/pnas.2108104118

**Published:** 2021-12-13

**Authors:** Kazuhiko Okamura, Johannes M. Dijkstra, Kentaro Tsukamoto, Unni Grimholt, Geert F. Wiegertjes, Akiko Kondow, Hisateru Yamaguchi, Keiichiro Hashimoto

**Affiliations:** ^a^Faculty of Clinical Engineering, School of Health Sciences, Fujita Health University, Toyoake 470-1192, Japan;; ^b^Institute for Comprehensive Medical Science, Fujita Health University, Toyoake 470-1192, Japan;; ^c^Laboratory for Proteins and Genes, Center for Joint Research Facilities Support, Fujita Health University, Toyoake 470-1192, Japan;; ^d^Fish Health Research Group, Norwegian Veterinary Institute 1433 Aas, Norway;; ^e^Aquaculture and Fisheries Group, Department of Animal Sciences, Wageningen University and Research, 6700 AH Wageningen, The Netherlands;; ^f^Advanced Comprehensive Research Organization, Teikyo University, Tokyo 173-0003, Japan;; ^g^Department of Medical Technology, School of Nursing and Medical Care, Yokkaichi Nursing and Medical Care University, Yokkaichi 512-8045, Japan;; ^h^Emeritus Professor of Department of Biomedical Polymer Science, Institute for Comprehensive Medical Science, Fujita Health University, Toyoake 470-1192, Japan

**Keywords:** major histocompatibility complex, MHC class divergence, MHC class I, MHC class II, molecular evolution

## Abstract

Two classes of major histocompatibility complex (MHC) molecules, MHC class I and class II, play important roles in our immune system, presenting antigens to functionally distinct T lymphocyte populations. However, the origin of this essential MHC class divergence is poorly understood. Here, we discovered a category of MHC molecules (W-category) in the most primitive jawed vertebrates, cartilaginous fish, and also in bony fish and tetrapods. W-category, surprisingly, possesses class II–type α- and β-chain organization together with class I–specific sequence motifs for interdomain binding, and the W-category α2 domain shows unprecedented, phylogenetic similarity with β_2_-microglobulin of class I. Based on the results, we propose a model in which the ancestral MHC class I molecule evolved from class II–type W-category. The discovery of the ancient MHC group, W-category, sheds a light on the long-standing critical question of the MHC class divergence and suggests that class II type came first.

## Significance

Two classes of major histocompatibility complex (MHC) molecules, MHC class I and MHC class II, constitute the basis of our elaborate, adaptive immune system as antigen-presenting molecules. They perform distinct, critical functions: especially, MHC class I in case of antivirus and antitumor defenses, and MHC class II, in case of effective antibody responses. This important class diversification has long been enigmatic, as vestiges of the evolutionary molecular changes have not been found. The revealed ancient MHC category represents a plausible intermediate group between the two classes, and the data suggest that class II preceded class I in molecular evolution. Fundamental understanding of the molecular evolution of MHC molecules should contribute to understanding the basis of our complex biological defense system.

The major histocompatibility complex (MHC) class I and class II groups each constitute a multigene family created by gene duplications and subsequent diversifications, with divergent members possessing distinct functions (1, 2). The classical MHC class I and class II molecules play central roles in our immune system by presenting antigens to T lymphocytes (2, 3). Classical MHC class I molecules present peptide antigens to T cell receptors (TCRs) on CD8^+^ T lymphocytes, whereas classical MHC class II molecules present peptide antigens to TCR on CD4^+^ T lymphocytes. After the interaction with the peptide antigen/MHC molecular complex, CD8^+^ T lymphocytes play important roles in the destruction of target cells (e.g., virus-infected cells or tumor cells), while CD4^+^ T lymphocytes play vital roles in helping or regulating antigen-presenting immune cells, including B lymphocytes, which can become antibody-secreting cells (3). Thus, the MHC class divergence is directly linked with our basic immune functions. However, despite decades of MHC research, there has been little progress in understanding the origin of this critical MHC class divergence (4–15).

MHC class I and class II genes have been identified not only in bony fish and tetrapods (2, 16) but also in cartilaginous fish, the most primitive jawed vertebrates (17–21). Authentic MHC class I– or class II–like genes have not been demonstrated in the extant jawless fish which possess distinct forms of immune defense. Therefore, the ancestral, antigen-presenting MHC molecule may have arisen, followed by its class diversification, in the common ancestor of jawed vertebrates, in concert with the appearance of their antibody and TCR antigen recognition systems (2).

The MHC molecules of the two classes show similarity to each other in their sequences and three-dimensional structures (3). Both classes possess a pair of membrane-distal extracellular domains (peptide-binding domains in the case of the classical MHC molecules) that together form a unique structure composed of an eight-stranded β-sheet topped by two α-helix components and a pair of membrane-proximal extracellular domains that each form an immunoglobulin (Ig)-like, C1-set (22) domain structure. However, the two classes display different combinatorial architectures of these four extracellular domains. A class I molecule is composed of a heavy chain with three extracellular domains (α1 and α2 for the membrane-distal domains; α3 for the membrane-proximal, Ig-like domain) and a noncovalently associated, single, Ig-like domain β_2_-microglobulin (β_2_-m). In contrast, a class II molecule is composed of two structurally similar chains, α and β, each consisting of two extracellular domains, namely, a membrane-distal domain and a membrane-proximal, Ig-like domain (α1 and α2, respectively, for α-chain; β1 and β2 for β-chain). Furthermore, a class I heavy chain and class II α- and β-chains each possess a connecting peptide (CP)/transmembrane (TM)/cytoplasmic (CY) region. Therefore, a class I molecule has a single CP/TM/CY region while a class II molecule has two.

Based on the similarities in the sequences and presumed structures between class I and class II, and on considerations of parsimony, creation of class I from class II was proposed previously (4, 7, 8, 10, 11). From different standpoints, the possible creation of class II from class I was also discussed (6, 9). However, findings of MHC molecules with features which suggest a specific direction of class diversification were not reported thus far (12–15). In the present study, we discovered a category of MHC molecules which possesses dual nature regarding the two MHC classes and, therefore, appears to be critical for the elucidation of the class diversification.

## Results and Discussion

### An Ancient MHC Group, W-category, Revealed.

**Fig. 1.**
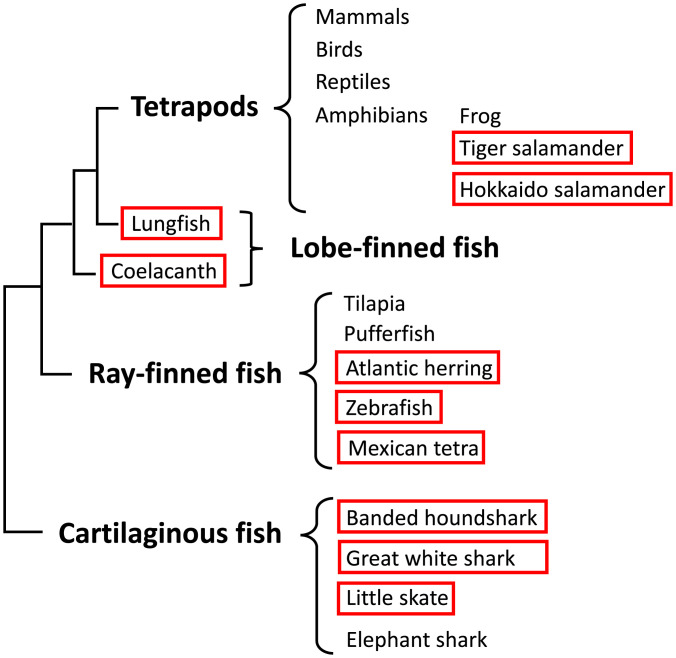
Identification of W-category genes (*WA* and *WB*) from diverse animal groups shown in a schematic phylogenetic classification of the major groups of jawed vertebrates. The schematic relative placements of major groups of jawed vertebrates (cartilaginous fish, ray-finned fish, lobe-finned fish, and tetrapods) are based on the reported phylogenetic tree of jawed vertebrates (34). Some representative names of animals or animal groups are shown in each major group. The names of animals in which we identified W-category *WA* and *WB* genes are surrounded by red lines. We experimentally identified the transcripts of W-category genes from banded houndshark, zebrafish, Mexican tetra, West African lungfish, and tiger salamander among others. The extended summary of the identification of W-category genes is shown in *SI Appendix*, Table S1.

Previously, we reported an MHC-like genomic fragment from cartilaginous fish (banded houndshark), which constituted a single, Ig-like, C1-set domain exon of an MHC molecule (23). The deduced, single-domain sequence was shown to possess MHC class I–like sequence features (23) and to have some clustering affinity with the class I group in a phylogenetic tree (10). Subsequently, as we succeeded in isolating the authentic classical MHC class I genes from banded houndshark (17), this single-exon sequence became an enigma. In the present study, from banded houndshark, we succeeded in isolating class II α-chain–type and β-chain–type genes, the latter including a full-length sequence that overlaps the above-mentioned single, Ig-like domain exon reported previously (23) and found these genes to be genetically linked in the genome (see the section *Like MHC class II, W-category α- and β-chain Genes Exist as a Pair in the Genome* *and Their Gene Products Appear to Form a Heterodimer*). Rigorous searches in genomic and transcriptomic databases and the isolation of relevant sequences eventually revealed that there exists a previously unrecognized, ancient category of MHC genes in jawed vertebrates (see *SI Appendix*, Table S1 for an overview). The molecules of this category possess a class II–type domain architecture but at the same time show unique class I sequence features. We named this group “W-category” in reference to the possession of a highly characteristic tryptophan (“W” in single-letter amino acid code) in the α-chain Ig-like domain. We revealed the existence of W-category genes in all major groups of jawed vertebrates, namely, cartilaginous fish, bony fish, and tetrapods, and representative animals are shown in Fig. 1 and *SI Appendix*, Table S1. However, in many other jawed vertebrates (e.g., euteleosts among teleost fish, frogs among amphibians, reptiles, birds, and mammals), the W-category genes have not been identified thus far and may have been lost in evolution.

### W-category Possesses Class II Domain Architecture.

**Fig. 2.**
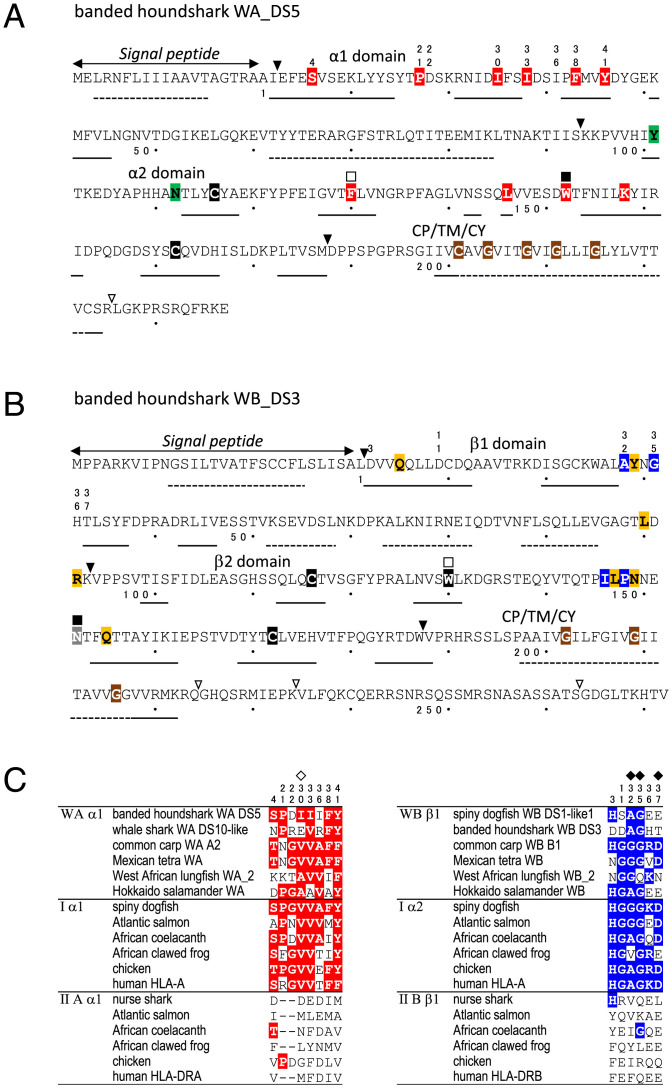
W-category molecules exhibit class II domain architectures together with class I–specific interdomain features. (*A* and *B*) Representative WA (*A*) and WB (*B*) sequences of banded houndshark display class II organization. Triangles refer to corresponding exon/intron borders at the DNA level and open triangles show those within CP/TM/CY. Double-sided arrows indicate signal peptide predictions. The secondary structure predictions are shown by solid lines (β-strand) and dotted lines (α-helix), respectively. (Red) WA/class I–characteristic residues. (Blue) WB/class I–characteristic residues. (Green) β_2_-m/WAα2/IIAα2–characteristic residues. (Orange) Class I/WB/class IIB–characteristic residues. (Gray) Most WB possess a glycine at this position. (Brown) Conserved residues in class II TM region. (Black) Cysteine and tryptophan residues conserved in the Ig superfamily. The numbers above the residues, which are different from those of the mature protein, match those in *C*, the squares above the residues match those in Fig. 3. The numbers with a dot below the residues indicate those of the mature protein, and in every 10 residues, a dot is indicated. (*C*) Selected amino acid residues shared between representative WAα1 and Iα1 (red) and between WBβ1 and Iα2 (blue). The positions of HLA-A2 human class I heavy chain residues which interact with β_2_-m L55 (open diamond) or with β_2_-m W61 (filled diamond) are indicated. The residue numbers in *C* correspond to those in Dataset S1.

**Fig. 3.**
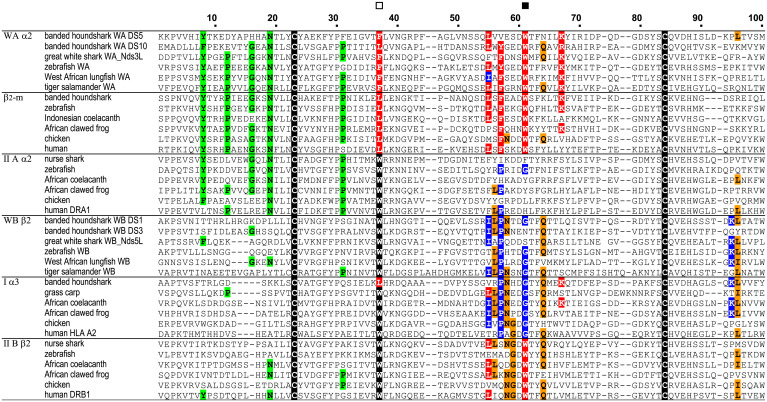
Sequence similarity between W-category and class I in Ig-like, C1-set domains. Color-shading principles are the same as in Fig. 2. An open square indicates position 37 of the highly conserved tryptophan in the Ig superfamily, and a filled square indicates position 61, where especially important interdomain residues are present.

**Fig. 4.**
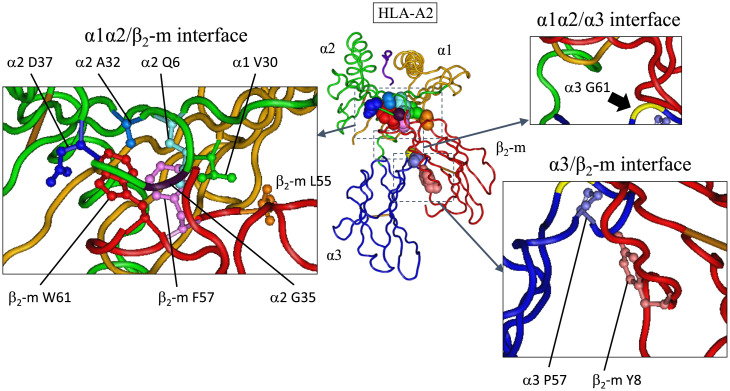
Three class I–specific interdomain interface features in HLA-A2 (human classical MHC class I molecule), which can also be observed in the W-category molecules. In the center, the crystal structure of human MHC class I molecule, HLA-A2, is shown. α1 (orange), α2 (green), and α3 (blue) domains of heavy-chain, β_2_-m (red), and a bound peptide (dark purple) are shown in tube worm. The side chains and main-chain Cα of the interdomain residues, characteristic for W-category and class I molecules, are shown with space-filling models with individual colors (not referring to colors used in other figures). The three interfaces are enlarged and indicated separately in small panels. In these panels, the important amino acid residues are shown in ball and stick model, except glycines. At the α1α2/β_2_-m interface: α1V30 (green), α2A32 (blue), α2G35 (dark violet), α2D37 (dark blue), β_2_-m L55 (orange), β_2_-m F57 (light magenta), and β_2_-m W61 (red). In this interface, Q6 of α2 domain (light blue), which interacts with both F57 and W61 of β_2_-m, is also shown, although Q6 is also conserved to some extent at the corresponding position of MHC class II. β_2_-m W61 forms the conserved hydrogen bonds with α2D37 and α2Q6. At the α1α2/α3 interface: α3G61 (yellow, indicated by thick arrow), which corresponds to invariant W61 of class IIB β2 domain. Compared to class II molecules, W-category and class I molecules impressively share the feature of not possessing tryptophan (W) residue at this position 61 of WB β2 domain or class I α3 domain. At the α3/β_2_-m interface: α3P57 (light purple) and β_2_-m Y8 (coral) form the evolutionarily conserved hydrogen bond. The structure is based on 1QSF of the Protein Data Bank ID, and the interacting αβ TCR situated above HLA-A2 is not depicted. The bound peptide is shown in dark purple, and the disulfide bridges between cysteines are shown in light brown. Amino acid numbers are based on Dataset S1 for the membrane-distal domains and based on Fig. 3 and Dataset S2 for the membrane-proximal domains. For molecular modeling of a W-category molecule, see *SI Appendix*.

In Fig. 2 *A* and *B*, we show representative, deduced amino acid sequences of the W-category isolated from banded houndshark, W-category α-chain (WA) and W-category β-chain (WB), corresponding to an α-chain and a β-chain of class II–type molecules, respectively (*SI Appendix*, Figs. S1 and S2 and Datasets S1–S3 for sequence comparisons of WA and WB; *SI Appendix*, Figs. S3 and S4 for the genomic and expression analyses of the banded houndshark W-category genes, respectively). The domain architectures of both WA and WB are typical for MHC class II chains, each consisting of a signal peptide, a membrane-distal domain, and a membrane-proximal, Ig-like domain plus CP/TM/CY regions. Predicted distributions of WA and WB secondary structures are also characteristic for MHC molecules. The assignments of the W-category sequences as an α-chain or as a β-chain are supported by the presence of respective, chain-specific residues including those in the Ig-like domains and unique glycines in the TM regions [(24); Datasets S1–S3]. Further sequence features highlighted in Fig. 2 *A* and *B* are addressed in Figs. 2*C*, 3, and 4 (see the sections *W-category Possesses Class I Sequence Features*, *Class I Interdomain Sequence Features Shared by W-category*, and *Additional Class I Features of W-category*).

### W-category Possesses Class I Sequence Features.

Amino acid sequence comparisons between W-category and other MHC molecules revealed striking, class I–like sequence features of W-category molecules (Figs. 2*C* and 3). Previous analyses comparing class I and class II molecules (e.g., refs. 4, 5, 7, 8, 10, 11, and 25–28) recognized the following pairs of phylogenetically related domains based on sequences and unique, geometrical, structural positions (*SI Appendix*, Fig. S5): Iα1 with IIAα1, Iα2 with IIBβ1, Iα3 with IIBβ2, and β_2_-m with IIAα2. Within this phylogenetic context, the present study revealed special sequence similarities between W-category and class I molecules that distinguish them from class II in the first half of the membrane-distal domains (red and blue residues in Fig. 2 and Dataset S1) and the membrane-proximal, Ig-like domains (red and blue residues in Fig. 3 and *SI Appendix*, Figs. S5–S7 and Dataset S2). Importantly, many of the residues specifically shared between W-category and class I molecules can be found at the interdomain interfaces of an MHC class I molecule (Fig. 4 and *SI Appendix*, Figs. S5 and S6).

### Class I Interdomain Sequence Features Shared by W-category.

Class I interdomain sequence features at the domain interfaces, which are remarkably shared by W-category, are described here. Except for the β-sheet–forming interface between the two membrane-distal domains (α1 and α2), class I molecules have three interdomain interfaces: α1α2/β_2_-m, α1α2/α3, and α3/β_2_-m [(29); Fig. 4], and in all these three interfaces, W-category shares sequence features with class I as described in this section.

For the α1α2/β_2_-m interface of class I, the most pronounced residue specifically shared by WAα2 and β_2_-m is tryptophan (W)-61 (indicated by a filled square in Fig. 3 and a red residue in Fig. 4 and *SI Appendix*, Fig. S5 and Dataset S2). In an MHC class I molecule, this residue W61 constitutes the central major part of this interface, projecting from β_2_-m into a pleat of the α1α2 β-sheet [(25, 29, 30); Fig. 4 and *SI Appendix*, Fig. S5]. W61 of β_2_-m interacts with as many as six residues of the α2 domain (29), which include W-category/class I–characteristic A32, G35, and D37 (Figs. 2*C*, *Right* and 4) and W-category/class I/class II–shared Q6 (Fig. 4). W61 forms conserved hydrogen bonds with D37 and Q6 of the α2 domain. In addition to W61, at the same interface, the highly conserved L55 and F57 of β_2_-m interact with class I α1 V30 (also found in W-category; Figs. 2*C* and 4) and α2 Q6 (Fig. 4), respectively. At the corresponding positions of these β_2_-m residues, W-category WAα2 possesses L55 and F/Y57, respectively, thus resembling β_2_-m (Figs. 3 and 4 and *SI Appendix*, Fig. S5 and Dataset S2). Compared to class I, conventional (hitherto published classical and nonclassical) class II possesses distinct and not well-conserved features at the corresponding interface (class II α1β1/α2), for example, class II lacks highly conserved residues at the IIAα2 positions corresponding to F57 and W61 of β_2_-m [(31); *SI Appendix*, Fig. S5 and Datasets S1 and S2]. Thus, W-category possesses class I–specific sequence features at this α1α2/β_2_-m interface.

For the α1α2/α3 interface of class I, Iα3 possesses glycine (G)-61 (Figs. 3 and 4 and *SI Appendix*, Fig. S5 and Dataset S2), which is mostly shared by W-category WBβ2, while a conventional class II molecule invariably possesses W61 at the corresponding position of IIBβ2 domain (Fig. 3 and *SI Appendix*, Fig. S5 and Dataset S2). In class II molecules, the invariant W61 of IIBβ2 makes an important contribution at the corresponding class II α1β1/β2 interface (31, 32), interacting with several residues highly conserved in class II but not conserved in the corresponding positions of W-category and class I (Fig. 2*C* and Dataset S1 and *SI Appendix*, Fig. S5). Thus, W-category WBβ2 domain resembles class I α3 domain in mostly possessing G61 (Dataset S2) and in not possessing the W61 residue, which is important for conventional class II molecules.

For the α3/β_2_-m interface of class I, Iα3 possesses P57 which interacts with Y8 of β_2_-m through an evolutionarily conserved hydrogen bond (Figs. 3 and 4 and *SI Appendix*, Fig. S6 and Dataset S2). Like class I, W-category possesses both P57 and Y8 in the corresponding domains, WBβ2 and WAα2, respectively, while class II possesses neither conserved P57 in IIBβ2 nor a well-conserved Y8 in IIAα2.

In short, W-category remarkably possesses class I–specific sequence features in all three corresponding interdomain interfaces described in this section.

### Additional Class I Features of W-category.

In addition to the interdomain features, W-category also exhibits other class I–specific features. One pronounced example is the absence of a tryptophan residue at position 37 of Ig-like domain of WAα2 and of β_2_-m (indicated by an open square in Fig. 3). At this position, a tryptophan (W) is highly conserved among Ig superfamily members, and it is located within the central core of the domain. Instead of a tryptophan, WAα2 and β_2_-m possess F/L37 and L37, respectively (Fig. 3 and Dataset S2 and *SI Appendix*, Fig. S5), whereas the corresponding IIα2 domain in class II invariably possesses a tryptophan (Fig. 3 and Dataset S2). Other than the above-listed residues, W-category and class I share several additional class I–specific residues, and those are included in Figs. 2*C* and 3 and *SI Appendix*, Fig. S7. Besides unique similarities in conserved residues, W-category and class I also exhibit shared class I–specific features in regard to deletions/insertions at three regions of the membrane-distal domains that show apparent class I/class II disparity in sequence length (*SI Appendix*, Fig. S21 and Dataset S1).

### Like MHC Class II, W-category α- and β-chain Genes Exist as a Pair in the Genome and Their Gene Products Appear to Form a Heterodimer.

**Fig. 5.**
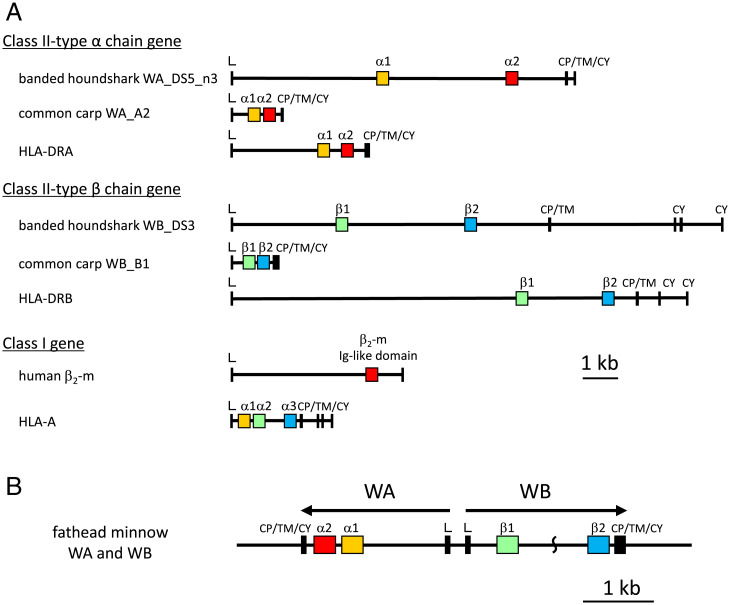
The genomic structures of W-category genes show class II domain architectures. (*A*) The genomic organization of coding exons and introns of the class I– and class II–type genes, the latter including representative W-category genes. Yellow- or green-colored boxes indicate related membrane-distal, domain-encoding exons: *WA* α1, classical class II *HLA-DRA* α1, and classical class I *HLA-A* α1 in yellow and *WB* β1, classical class II *HLA-DRB* β1, and *HLA-A* α2 in green. Red or blue boxes indicate related, Ig-like domain exons: *WA* α2, *HLA-DRA* α2, and *β_2_-m* in red and *WB* β2, *HLA-DRB* β2, and *HLA-A* α3 in blue. A bar indicates 1 kb. (*B*) The genomic organization of a pair of *WA* and *WB* genes in teleost fish, represented by the fathead minnow genes, *WA* and *WB*. The arrows indicate predicted, transcriptional directions. Between the β1 and β2 domain–encoding exons of *WB*, a portion of the genomic sequence is not available. Similar head-to-head genomic organization can be observed for *WA*/*WB* pairs of sharks, *WA*/*WB* pairs of the other teleost fish, and a *WA*/*WB* pair of African coelacanths (*SI Appendix*, Figs. S11 and S12 and *SI Appendix*). A bar indicates 1 kb. The sequences used in the figure are listed in *SI Appendix*, Table S3.

The genomic structures of *WA* and *WB* genes of various animals are similar to those of α- and β-chain genes of MHC class II (Fig. 5*A* and *SI Appendix*, Fig. S8). In banded houndshark, *WA* and *WB* genes are present in the same linkage group in the genome (*SI Appendix*, Figs. S9 and S10), and the recent whole-genome, shotgun-sequencing data of a great white shark revealed a gene cluster of three presumable pairs of *WA*/*WB* genes (*SI Appendix*, Fig. S11). In cases of the teleost fish and the lobe-finned fish coelacanth, *WA* and *WB* genes exist as a single pair closely connected to each other in the genome, suggesting that their gene products form a heterodimer-like conventional MHC class II molecules [(2, 3); Fig. 5*B* and *SI Appendix*, Figs. S11 and S12 and *SI Appendix*].

**Fig. 6.**
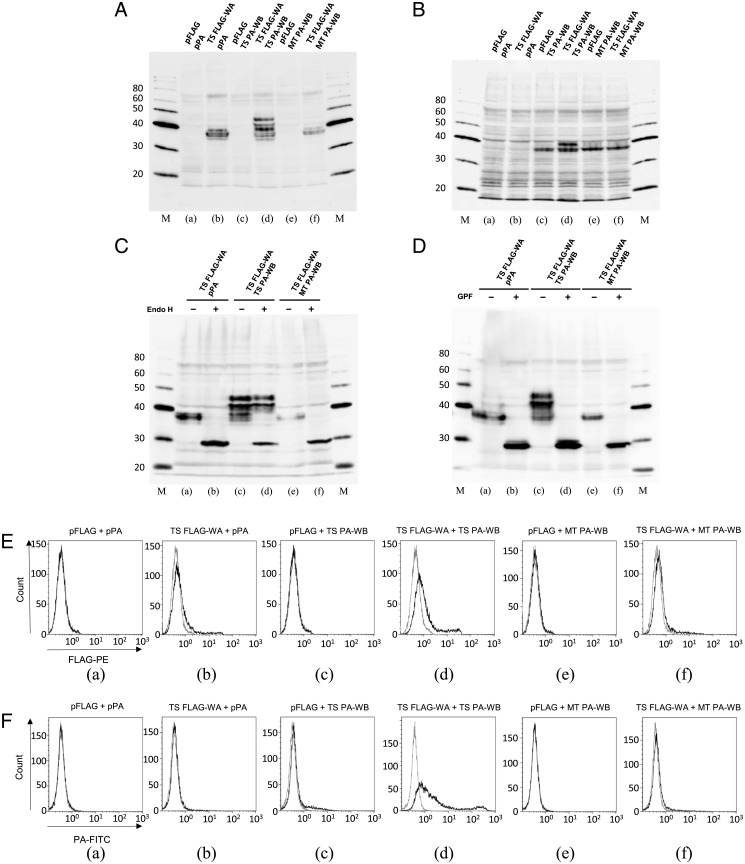
Specific interaction between WA and WB chains of tiger salamander. (*A*–*D*) Production of recombinant WA and WB proteins in transfected cells. Total proteins from Chinese hamster ovary (CHO) K-1–transfected cells used for flow cytometry in *E* and *F* were investigated by Western blot analyses. Above each lane, two kinds of cotransfected DNA are indicated. (*A*) FLAG-tagged WA detected by anti-FLAG antibody. (*B*) PA-tagged WB detected by anti-PA antibody. Apparent molecular weights of protein-size markers are indicated in kilo Dalton. Abbreviations are the following: pFLAG, empty FLAG vector; pPA, empty PA vector; TS FLAG-WA, tiger salamander N-terminal FLAG-tagged WA; TS PA-WB, tiger salamander N-terminal PA-tagged WB; MT PA-WB, Mexican tetra (teleost fish) N-terminal PA-tagged WB. MT PA-WB was used instead of TS PA-WB in some cases as a recombinant WB of a distantly related species (see Fig. 1 for tiger salamander and Mexican tetra). β-actin controls using the membrane in *A* or *B* are shown in *SI Appendix*, Fig. S13. (*C* and *D*) Digestion of recombinant WA protein by Endoglycosidase H (Endo H), which does not cleave highly processed complex oligosaccharides, or Glycopeptidase F (GPF), which broadly cleaves oligosaccharides from *N*-linked glycoproteins. Enzyme-digested total proteins from transfected CHO-K1 cells were investigated by Western blot analyses. (*C*) FLAG-tagged WA with/without (±) Endo H digestion detected by anti-FLAG antibody. (*D*) FLAG-tagged WA with/without (±) GPF digestion detected by anti-FLAG antibody. The results of the PA-tagged WB are shown in *SI Appendix*, Fig. S14. Apparent molecular weights of protein-size markers are indicated in kilo Dalton. See *SI Appendix* for details. (*E* and *F*) Cell surface expression of recombinant WA and WB proteins of tiger salamander in CHO K-1–transfected cells, as determined by flow cytometry. Above each panel, two kinds of cotransfected DNA are indicated. (*E*) Anti-FLAG antibody binding to N-terminal FLAG-tagged WA on the cell surface. Solid lines represent the results with anti-FLAG antibody, and gray lines represent the results with isotype control antibody. (*F*) Anti-PA antibody binding to N-terminal PA-tagged WB on the cell surface. Solid lines represent the results with anti-PA antibody, and gray lines represent the results with isotype control antibody. Abbreviations are the same as those in *A*–*D*. See *SI Appendix* for details.

In accordance with these observations, we found specific interaction between W-category α- (WA) and β- (WB) chains and cell surface expression of these chains using recombinant proteins of tiger salamander (Fig. 6 and *SI Appendix*, Figs. S13 and S14 and *SI Appendix*). We observed specific glycosylation processing for the recombinant WA and WB chains only when both WA and WB chains of tiger salamander were simultaneously introduced into cultured cells (Fig. 6 *A*, *d* and *B*, *d*). In case of WA chain, this Glycopeptidase F–sensitive glycosylation appeared to include Endoglycosidase H–resistant, advanced complex glycan structure (Fig. 6 *C*, *d* and *D*, *d*), suggesting intracellular processing of WA chain through the Golgi system. Furthermore, we conducted cell surface expression analyses of the tagged recombinant W-category chains using flow cytometry (Fig. 6 *E* and *F*) and low but reproducible binding of anti-FLAG antibody to FLAG-tagged WA (Fig. 6 *E*, *d*), and significant binding of anti-PA (representing a dodecapeptide of human podoplanin) antibody to PA-tagged WB (Fig. 6 *F*, *d*) could be observed on the cell surface, only in the presence of both tiger salamander WA and WB.

The *Mhc* region forms an ancient linkage group in which many gene duplications took place, and the classical MHC class I (heavy chain) and class II genes are known to be closely linked even in sharks (33). Likewise, the β_2_-m (class I light chain) gene was found to be closely linked with the *Mhc* region in sharks, in contrast to the situation known for many other species [(28); *SI Appendix*, Fig. S15 with a different shark species; *SI Appendix*]. In the present study, a pair of W-category chain genes were also found in the *Mhc* region (*SI Appendix*, Fig. S12*A* and *SI Appendix*), in the case of coelacanth (34, 35). Even though the W-category gene pairs of other species are situated in diverse genomic environments, presumably because of many genomic changes, including gene translocations and chromosomal rearrangements (*SI Appendix*, Figs. S9–S12), some surrounding sequences can be classified as *Mhc* region related (*SI Appendix*, Figs. S11 and S12). Thus, the *Mhc* region is the presumable place of evolutionary origin of W-category genes.

### W-category α2 Domain Clusters with Class I β_2_-m in Phylogenetic Tree Analyses.

**Fig. 7.**
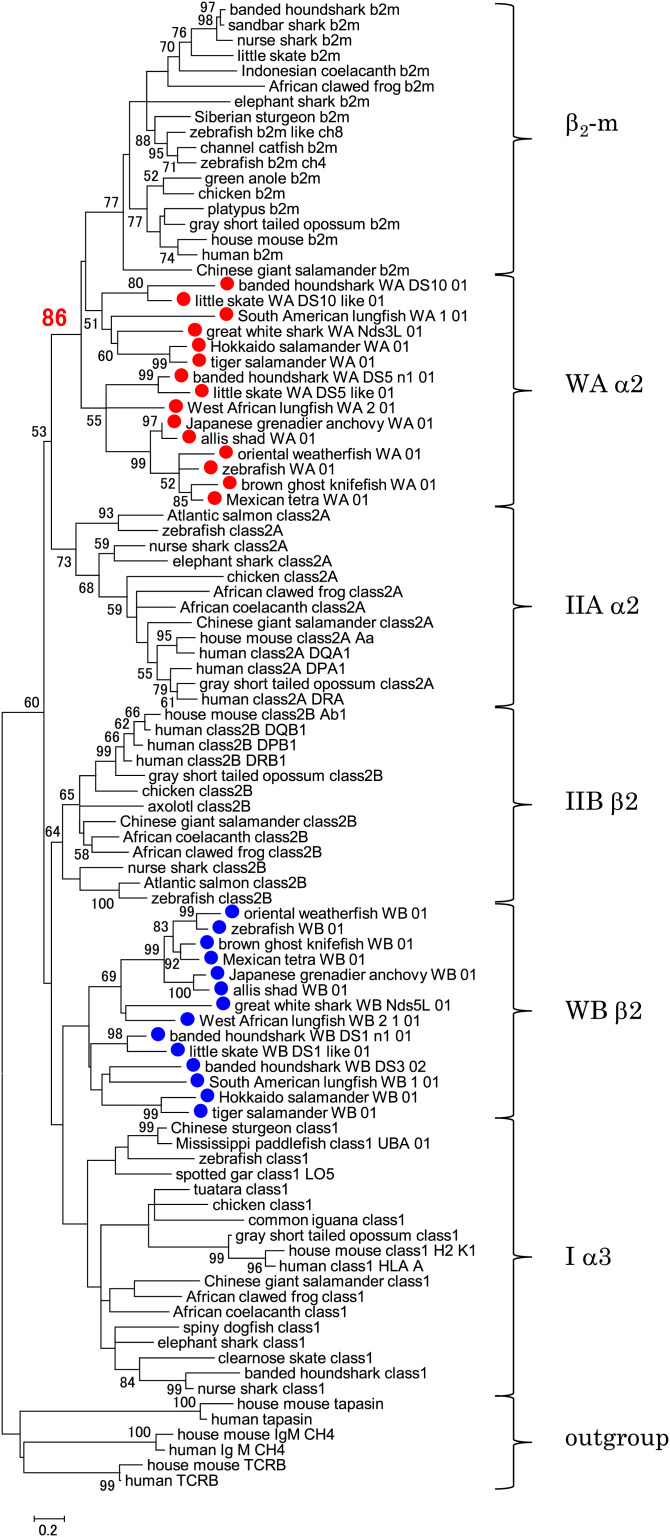
Close relationship between W-category α2 domain and β_2_-m of the MHC class I molecules. The phylogenetic tree was constructed with the amino acid sequences of the membrane-proximal, Ig-like, C1-set domains of selected MHC molecules using the maximum-likelihood method. The percentage of trees (bootstrap value, 50 or greater) in which respective sequences clustered together is shown next to the nodes. The bootstrap value at the WAα2/β_2_-m node is shown in red. The alignment used for this analysis is shown in Dataset S2. Red dots mark WAα2 sequences, and blue dots mark WBβ2 sequences. For outgroup sequences, C1-set domains of Ig M, TCRB (T cell receptor β chain), and tapasin were used. Similar phylogenetic tree analyses, including those with the DNA sequences, those using the neighbor-joining method for estimating phylogenetic trees, and those with more sequences, are shown in *SI Appendix*, Figs. S16–S18.

We conducted a phylogenetic tree estimation for the evolutionary history of MHC molecules using Ig-like domains of representative MHC molecules, including W-category (Fig. 7), because these domains form the best-conserved parts among these molecules. Importantly, WAα2 and β_2_-m sequences cluster together with a significant bootstrap value upon phylogenetic tree analysis (Fig. 7), which is consistent with the observation that WAα2 and β_2_-m share a number of unique amino acid residues (see the sections *W-category Possesses Class I Sequence Features*, *Class I Interdomain Sequence Features Shared by W-category*, and *Additional Class I Features of W-category*). The clustering of WAα2 and β_2_-m, observed with the amino acid sequence data (shown in Fig. 7), was also obtained with the DNA sequence data (*SI Appendix*, Fig. S16) and was obtained using different methods for estimating phylogenetic trees (e.g., maximum likelihood [Fig. 7] and neighbor joining [*SI Appendix*, Fig. S17]). A similar analysis that additionally included the nonclassical class II DM molecules and some nonclassical class I molecules also produced similar clustering of WAα2 and β_2_-m (*SI Appendix*, Fig. S18). On the other hand, for the Iα3/IIBβ2 group to which W-category WB β2 domains belong, we did not obtain results with high-branching resolution (Fig. 7 and *SI Appendix*, Figs. S16–S18), which is similar to the previous observations for this group (e.g., refs. 10 and 28).

### W-category as a Multigene Family.

Like the MHC class I and the conventional MHC class II groups, W-category exhibits the nature of a multigene family, namely, it includes divergent subgroups (Figs. 3 and 7 and *SI Appendix*, Figs. S1, S2, and S16–S18 and Datasets S1–S3). As multigene families, the MHC class I and the conventional MHC class II groups each contain divergent members. Some members do not bind any ligands in their grooves, and some nonclassical class I molecules bind lipids (e.g., in case of CD1) or small metabolites (e.g., in case of MR1) instead of peptides. In humans, the MHC class I molecules, ranging from classical HLA-A, HLA-B, and HLA-C to nonclassical HLA-E, HLA-F, HLA-G, CD1, MR1, HFE, and FcRn, can possess very divergent heavy chains but commonly possess β_2_-m as a light-chain component (2). The human class II molecules include classical HLA-DP, HLA-DQ, and HLA-DR and nonclassical HLA-DO and HLA-DM (2). Like many nonclassical class I and class II, the various W-category subgroups identified in the present study do not possess complete sets of peptide-interacting residues conserved either in the classical MHC class I or in the classical MHC class II molecules and do not show the high, allelic polymorphism characteristic of classical MHC molecules (Dataset S1 and *SI Appendix*, Figs. S19 and S20 and *SI Appendix*). However, the W-category subgroup found in the teleost fish exhibits an amino acid conservation profile at expected groove positions (*SI Appendix*, Figs. S21 and S22 and Table S4 and *SI Appendix*) that resembles that of the MHC-Z molecules, an ancient nonclassical class I group, which shares important, peptide-binding motifs with classical MHC class I (36–38). Thus, probably like class I and class II groups, W-category also includes at least some members which possess grooves with ligand-binding capacity (*SI Appendix*, Fig. S22). Therefore, it is feasible that, as a multigene family, in ancient times W-category may have contained a peptide-binding molecule that played an intermediate role in the MHC class divergence.

### Implications of W-category for the MHC Class Divergence.

**Fig. 8.**
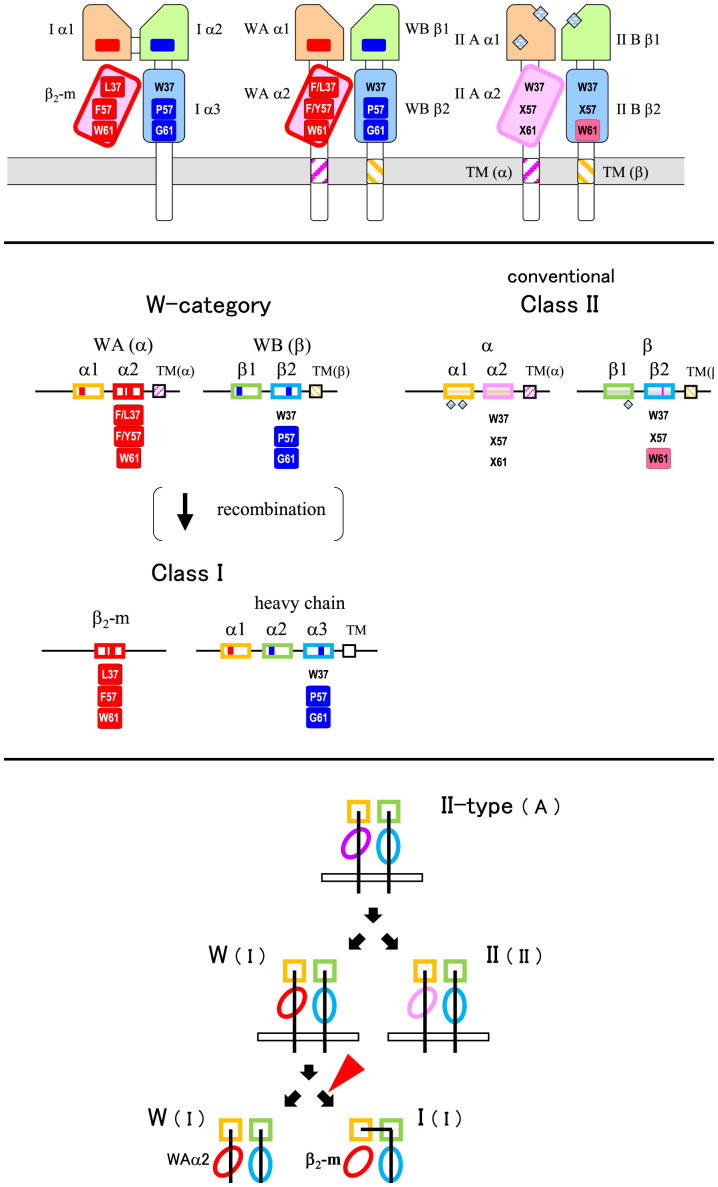
Summary of W-category with features of both class I and class II. (*A*) Schematic drawings of class I, conventional class II, and predicted W-category molecular structures. W-category exhibits class II–like domain architectures and exhibits class I–like interdomain interfaces (indicated by “I-interfaces” in a parenthesis). In the membrane-distal domains (light orange and light green), red and blue boxes indicate class I–characteristic residues of class I α1 (Fig. 2*C*, *Left*) and α2 (Fig. 2*C*, *Right*), respectively, and include residues important at the interdomain interfaces. In the membrane-proximal, Ig-like, C1-set domains (light magenta and light blue), characteristic residues are shown at three representative positions, 37, 57, and 61 of Fig. 3. Residues in red rectangles are WAα2/β_2_-m characteristic, and residues in blue ones are WBβ2/Iα3 characteristic. W37 is highly conserved among the Ig superfamily; however, β_2_-m possesses L37, and WAα2 possesses F/L37 at this position. F57 and W61 of β_2_-m form the major part of the evolutionary conserved Iα1α2/β_2_-m interface, P57 of Iα3 forms part of the evolutionary conserved Iα3/β_2_-m interface, and G61 of Iα3 is located near the Iα1α2/Iα3 interface. W61 in magenta is highly conserved in conventional class IIB and forms the major part of the class II α1β1/β2 interface (corresponding to the class I α1α2/α3 interface). × denotes a nonconserved residue. Pale blue grid diamonds indicate gaps in the sequences compared to MHC class I and W-category in the membrane-distal domains of the conventional MHC class II molecules. Cell membranes are depicted in the gray color. TM regions of MHC molecules with class II α-chain features are shown in magenta stripes, and those with class II β-chain features are shown in light yellow stripes [(24); Dataset S3]. Tilted domains denote asymmetrically paired, Ig-like, C1-set domains both in MHC class I (25) and class II (27) molecules, and the overall structures of the MHC class I and class II molecules are quite similar (27). (*B*) Schematic drawings of class I, conventional class II, and W-category gene structures with selected features. Representative, characteristic features at the protein level (same as those in *A*) are shown under the corresponding exons. Only exons encoding the extracellular domains and an exon for the TM region are shown for simplicity. Although the genes for α- and β-chains of W-category and many conventional class II are present with the opposite transcriptional direction (as shown in Fig. 5*B*), they are depicted with the same direction in this figure to allow easier comparison of domain components. (*C*) Class II first model with W-category for MHC class divergence. The previous model (7, 15) is modified with W-category as an intermediate. The red arrowhead indicates the stage when alterations of domain architectures take place. The class I–type and the class II–type interdomain interfaces are indicated by (I) and (II), respectively, and unknown ancestral state of the interdomain interface is indicated by "(A)."

In the present study, we revealed W-category as an ancient MHC group, in addition to MHC class I and conventional MHC class II. Fig. 8 shows simplified figures of the protein domains (Fig. 8*A*) and the genomic structures (Fig. 8*B*) of these three kinds of MHC groups based on the major findings in this study: namely, 1) W-category exhibits class II–type domain architectures; 2) W-category exhibits class I characteristics, including class I–specific interdomain sequence features; and 3) W-category α2 domain exhibits a clustering with β_2_-m in the phylogenetic tree analyses. The overall characteristics of W-category appear to be remarkably appropriate for those of an intermediate molecule between class I and class II in the MHC class diversification. For decades, we have been searching for such molecules and finally we found W-category.

When W-category is incorporated into a model of the MHC class divergence, logically, both evolutionary directions can be considered, one from class I to W-category that possesses class II domain architecture (class I first model) and another from W-category to class I (class II first model) (*SI Appendix*, Fig. S23). For a class I first model, the formation of a class I–like molecule by transferring a complete peptide-binding region of a heat-shock protein (such as HSP70) to a β_2_-m–like, single, Ig-like domain and the following creation of a class II–type molecule from a class I molecule were previously proposed (9). However, the domain structure of HSP70 and the interactive mode of bound peptides turned out to be quite different from those of the MHC molecules, and a plausible explanation for this evolutionary direction has not been presented (15). Importantly, the phylogenetic topology of the WAα2/β_2_-m/IIAα2 domain group observed in the present study (Fig. 7 and *SI Appendix*, Figs. S16–S18) is not compatible with a class I first model (*SI Appendix*, Fig. S23).

For a class II first model, the creation of a class I heavy-chain gene from a pair of class II α- and β-chain genes was previously proposed (7, 15), based on sequence similarity and considerations of parsimony, and then further discussed by other researchers (8, 10, 11). In this case, a simple recombination event between the genes for the two chains was assumed, which placed the α1 domain exon of class II α-chain into the upstream of β1 plus β2 domain exons of class II β-chain, producing a class I heavy-chain–like domain architecture with three linked extracellular domains. The remaining part of class II α-chain containing α2 domain was speculated to become β_2_-m, losing most of the CP/TM/CY region (7, 8, 10, 15). However, hitherto no special class II–type candidate for an immediate class I precursor has been elucidated for decades (15). W-category possesses both class II domain architectures and class I–specific interdomain interfaces (Figs. 4 and 8*A*), and the phylogenetic analyses of the WAα2/β_2_-m/IIAα2 domain group (Fig. 7 and *SI Appendix*, Figs. S16–S18) support a class II first model.

Based on our results, we propose a model in which the ancestral class I heavy chain and β_2_-m genes were created from a pair of W-category α-chain and β-chain genes (Fig. 8 *B* and *C*). After the recombination at the DNA level, which presumably occurred in the *Mhc* region and transformed the class II–type domain architecture of W-category into the class I type, the interdomain interfaces at the protein level were preserved by the newly formed ancestral class I molecule. Thus, the incorporation of W-category into the class II first model allows the formation of the ancestral class I with a simple, exon-shuffling event, without further requirements for elaborate changes at the interdomain interfaces.

Future studies should include structural and functional investigations of various W-category molecules and additional searches for W-category members. Since classical MHC class I and class II and W-category can be identified in both cartilaginous fish and bony fish/tetrapods, it is concluded that the ancient common ancestor of these animal groups possessed all three MHC groups. The discovery of W-category provides a stunning addition of one fascinating MHC group in jawed vertebrates and casts a light on the understanding of MHC class divergence.

## Materials and Methods

Detailed information is provided in *SI Appendix*, *Materials and Methods*. All animals were handled according to the Guidelines for the Management of Laboratory Animals in Fujita Health University. DNA, RNA, and genomic and complementary DNA (cDNA) libraries were prepared basically using standard protocols. Rapid amplification of cDNA ends (RACE) reactions, PCR sequencing, and Southern blot analyses were conducted basically using standard protocols. Linkage analyses with banded houndshark were performed essentially as described previously (17). Database searches, analyses of DNA and amino acid sequences, and alignments of amino acid sequences were conducted basically using standard methods. Phylogenetic tree analyses were conducted mainly with the maximum-likelihood method. For recombinant protein studies, Chinese hamster ovary–K1 cells were used, and vector construction and transfection, analyses of proteins, and flow cytometry were described in *SI Appendix*, *Materials and Methods*. The references for the structural comparisons and the analysis of the conservation profile of a W-category subgroup were described in *SI Appendix*, *Materials and Methods* and *SI Appendix*.

## Supplementary Material

Supplementary File

Supplementary File

Supplementary File

Supplementary File

## Data Availability

All the DNA sequence data determined in the present study have been deposited and archived, and are available in the DNA DataBank of Japan and GenBank under the accession numbers listed in *SI Appendix*, Table S3. All other data in this study are included in the article and/or supporting information.
