# Supplementary material for: Discovery of an ancient MHC category with both class I and class II features
Source: Proc Natl Acad Sci U S A. 2021 Dec 13;118(51):e2108104118. doi: 10.1073/pnas.2108104118 (PMC8713811; doi:10.1073/pnas.2108104118)
Supplement: Supplementary File [file pnas.2108104118.sapp.pdf]

Supplementary Information

Discovery of an ancient MHC category with both class I and class II features

Kazuhiko Okamura*, Johannes M. Dijkstra*, Kentaro Tsukamoto*, Unni Grimholt, Geert F. Wiegertjes, Akiko Kondow, Hisateru Yamaguchi, Keiichiro Hashimoto§

*These authors contributed equally to this work.
§corresponding author. E-mail: keihashi@fujita-hu.ac.jp (K.H.)

Contents of Supplementary Information

Page
2 Materials and Methods
7 Appendix
1. Overview of W-category genes
10 2. Identification of W-category genes
28 3. Southern blot analyses of the banded houndshark W-category genes
4. Banded houndshark W-category genes are expressed in various tissues
5. Variation of W-category molecules
32 6. Conserved features at the Iα3/β_2-m interface
7. β_2-m K67 can form an intra-domain hydrogen bond
8. Linkage between the banded houndshark *β_2-m* gene and the *Mhc* region
33 9. Specific interaction between WA and WB
34 10. Conservation profile of the teleost fish W-category membrane-distal domains and molecular modeling of the extracellular domains of W-category molecule
71 11. Additional explanations for Fig. S5
73 12. Notes for Datasets
76 13. Detailed Author Contributions
77 Figs. S1 to S23
121 Tables S1 to S4
130 References (1-91)
138 Representative W-category sequences in FASTA format

Materials and Methods

Animals. Banded houndshark (*Triakis scyllium*) individuals were obtained as described in a previous paper (17). Zebrafish (*Danio rerio*) were obtained from the Zebrafish National BioResource Project, Laboratory for Developmental Gene Regulation, RIKEN Brain Science Institute (Wako, Saitama, Japan). Goldfish (*Carassius auratus*), Mexican tetra (*Astyanax mexicanus*), West African lungfish (*Protopterus annectens*) and tiger salamander (*Ambystoma tigrinum)* were all obtained from Meitosuien Co. Ltd. (Nagakute, Aichi, Japan). All animals were handled according to the Guidelines for the Management of Laboratory Animals in Fujita Health University.

DNA and RNA. The isolation of the genomic DNA of banded houndshark was described in a previous paper (17). For banded houndshark, total RNA was isolated from kidney, spleen, liver and blood using ISOGEN (Nippon Gene, Tokyo, Japan) or TRIzol (Invitrogen, Thermo Fisher Scientific, Waltham, MA, USA). For the other animal species, total RNA was isolated from various tissues (gills of goldfish and Mexican tetra; anterior half of zebrafish; kidney of West African lungfish; internal organs of tiger salamander) using TRIzol. Total RNA was used for RACE and RT-PCR experiments.

Genomic and cDNA libraries. The construction of a genomic library of banded houndshark (individual N0) was described previously (23). In the present study, an additional genomic library was constructed by partial digestion of banded houndshark (individual N0) red blood cell DNA with *Sau*3AI. The construction of a cDNA library of banded houndshark (individual N2) was described previously (17). The primers used to amplify and prepare various probes to screen the libraries are listed in Table S2.

RACE reactions. RACE reactions were conducted to obtain sequence information for 5'- and 3'-UTRs of relevant genes using SMART RACE cDNA Amplification Kit (Clontech, Takara Bio USA, Mountain View, CA, USA), SMARTer RACE cDNA Amplification Kit (Clontech) and GeneRacer Kit (Invitrogen). RACE-PCR was conducted to obtain entire coding regions of relevant genes using Advantage 2 Polymerase (Clontech, Takara Bio USA, Mountain View, CA, USA) with SMART RACE cDNA Amplification Kit, Advantage 2 Polymerase or Ex Taq DNA polymerase (Takara Bio Inc., Shiga, Japan) with SMARTer RACE cDNA Amplification Kit, and Ex Taq DNA polymerase with GeneRacer Kit.

PCR. The primer sequences used in PCR and RT-PCR are listed in Table S2. To obtain the coding sequences of banded houndshark *WA_DS5*, *WB_DS1* and *WB_DS3* genes, total kidney RNA (individual N1) was reverse-transcribed into cDNA using ReverTra Ace (TOYOBO, Osaka, Japan). In case of banded houndshark *WA_DS10*, Prime Script II High Fidelity RT-PCR kit (Takara) was used to reverse-transcribe RNA into cDNA. The coding sequences were amplified from cDNA using specific primers designed at 5' and 3' UTRs. PCR reactions were conducted using Ex Taq HS DNA polymerase (Takara) with 35 cycles and PCR products were cloned into pGEM-T Easy vector (Promega, Madison, WI, USA). In case of *WA_DS10*, PCR reactions were conducted using Prime STAR GXL DNA polymerase (Takara) with 32 cycles and PCR products were cloned into Bluescript vectors (Stratagene, Agilent Technologies, CA, USA) as described previously (23).

The genomic sequences of banded houndshark *WA_DS5* and *WA_DS10* genes were amplified from genomic DNA (individual N1) using specific primers designed at 5' and 3' UTRs. A partial genomic sequence containing the β2 domain exon of banded houndshark *WB_DS1* gene (individual N0) was reported previously (23; M85291) whereas part of the upstream intron region was additionally determined in the present study (LC200978). The CP/TM/CY region sequence of the banded houndshark *WB_DS1* gene was amplified from genomic DNA (individual N1) using specific primers derived from β2 domain and 3' UTR regions, and was determined in the present study (LC009542). The genomic sequence of 10.9 kb, which contains the first four exons of the banded houndshark *WB_DS3* gene, was determined using a λ phage clone isolated from a genomic library of banded houndshark individual N0. The genomic sequence of 4.5 kb, which contains the last three exons of the banded houndshark *WB_DS3* gene, was amplified from genomic DNA of banded houndshark individual N0 using specific primers derived from intron 4 and 3' UTR regions.

PCR conditions for amplification of genomic *WA_DS5*, *WA_DS10* and *WB_DS3* were as follows: denaturation at 94°C for 2 min, 5 cycles of denaturation at 98°C for 10 sec, annealing and elongation at 74°C for 10 min, 5 cycles of denaturation at 98°C for 10 sec, annealing and elongation at 72°C for 10 min, 5 cycles of denaturation at 98°C for 10 sec, annealing and elongation at 70°C for 10 min, 40 cycles of denaturation at 98°C for 10 sec, annealing and elongation at 68°C for 10 min and final elongation at 68°C for 7 min with KOD Fx Neo DNA polymerase (TOYOBO). PCR conditions for amplification of genomic *WB_DS1* were as follows: denaturation at 94°C for 2 min, 5 cycles of denaturation at 98°C for 10 sec, annealing and elongation at 74°C for 20 min, 5 cycles of denaturation at 98°C for 10 sec, annealing and elongation at 72°C for 20 min, 5 cycles of denaturation at 98°C for 10 sec, annealing and elongation at 70°C for 20 min, 40 cycles of denaturation at 98°C for 10 sec, annealing and elongation at 68°C for 20 min and final elongation at 68°C for 7 min with KOD Fx Neo DNA polymerase (TOYOBO). The PCR products were cloned into pCR-XL-TOPO vector (Invitrogen) or used as templates for direct sequencing.

For the isolation of the teleost fish and lungfish W-category genes, SuperScript III reverse transcriptase kit (Invitrogen) with random hexamer primers was used to construct cDNA. RT-PCR was conducted using Ex Taq DNA polymerase (Takara) with 40 or less PCR cycles. For isolation of tiger salamander W-category genes, RACE reactions were conducted using GeneRacer Kit and the entire coding sequences were amplified using Prime STAR HS DNA polymerase (Takara) with 35 cycles. PCR products were cloned into Bluescript vectors as described previously (23).

For linkage analyses of the banded houndshark *WB_DS10* gene using *Hha*I digestion, PCR conditions for amplification using DS10-2 and DS10-8 primers were as follows: denaturation of genomic DNA at 94 °C for 1 min, 40 cycles of denaturation at 94 °C for 30 sec, annealing at 60 °C for 30 sec and elongation at 72 °C for 4 min, and final elongation at 72 °C for 5 min with LA-Taq HS DNA polymerase (Takara). For linkage analyses using DS10-6 and DS10-14 primers, PCR conditions were the same as above except for using 35 cycles. For linkage analyses of the banded houndshark β_2-*m* gene, PCR conditions for amplification using β_2-m-F11 and β_2-m-R10 primers were as follows: denaturation of genomic DNA at 98 °C for 10 sec, 50 cycles of denaturation at 98 °C for 10 sec, annealing at 61 °C for 15 sec and elongation at 72 °C for 5 min, and final elongation at 72 °C for 10 min with Prime STAR DNA polymerase (Takara).

Expression of the banded houndshark *WA* and *WB* genes was studied using RT-PCR using RNA from spleen, kidney, liver and blood (individual N1) and two gene-specific primers, using Prime Script II High Fidelity RT-PCR Kit (Takara). RT-PCR conditions were as follows: denaturation at 98 °C for 1 min, 27, 32, 37 or 42 cycles of denaturation at 98 °C for 10 sec, annealing at 60 °C for 15 sec and elongation at 68 °C for 3 min, and final elongation at 68 °C for 5 min with Prime STAR GXL DNA polymerase (Takara). As a control gene, banded houndshark β-actin gene (39) was used under the following RT-PCR conditions: denaturation at 98 °C for 1 min, 16, 19, 22 or 27 cycles of denaturation at 98 °C for 10 sec, annealing at 60 °C for 15 sec and elongation at 68 °C for 3 min, and final elongation at 68 °C for 3 min with Prime STAR GXL (Takara Bio Inc., Shiga, Japan). In some experiments, RT-PCR conditions were slightly different (protocol No. 2) and as follows: denaturation at 98 °C for 30 sec, 35 cycles of denaturation at 98 °C for 10 sec, annealing and elongation at 60 °C for 3 min, and final elongation at 72 °C for 3 min with Ex Taq HS DNA polymerase (Takara).

Variations in the cDNA sequences of zebrafish WA and WB genes were studied with RT-PCR using RNA from anterior halves of adult zebrafish of various strains: AB, India (IND), TL and WIK. PCR reactions were conducted using Ex Taq DNA polymerase (Takara) with 40 or less PCR cycles.

Sequencing. Sequencing reactions were performed with BigDye Terminator v3.1 Sequencing Standard kit (Applied Biosystems), and nucleotide sequences were determined using 3100Avant/3130xl Genetic Analyzer (Applied Biosystems Life Technologies, Foster City, CA, USA). The DNA sequence of one genomic phage clone of *WB_DS3* was also determined using the above sequencing method. For the sequence determination of PCR products, various techniques including independent PCR reactions, analyses of multiple independent clones, PCR with different sets of primers and direct sequencing reactions were used to eliminate possible PCR and sequencing errors.

In the initial phase of our study, radio-labeled methods were used for sequencing PCR-amplified genomic DNA fragments and clones from genomic libraries and from cDNA libraries.

Southern blot analyses. Southern blot analyses for the linkage analyses of banded houndshark *WA_DS5*, *WB_DS1* and *WB_DS3* (Figs. S9 and S10) were performed essentially as described previously (17). Briefly, 5 μg of DNA digested with a restriction enzyme was electrophoresed through an agarose gel (0.8 %) and blotted onto a nylon membrane. The restriction enzymes used for the experiments for the analyses of *WA_DS5*, *WB_DS1* and *WB_DS3* were *Bam*HI, *Stu*I and *Bam*HI, respectively. After hybridization with the specific radio-labeled probes, membranes were finally washed with 0.1 x SSC/0.05 % SDS at 42° C for 30 min and autoradiographs were obtained. Southern blot analyses for banded houndshark individual N1 (Fig. S3 and *SI Appendix*) were performed with the DIG system using the PCR DIG Probe Synthesis Kit and the DIG Luminescent Detection Kit (Roche Applied Science).

Linkage analyses. As described above, the linkage analyses of the banded houndshark *WA_DS5*, *WB_DS1* and *WB_DS3* genes (Figs. S9 *A*, *C* and *D*, respectively) were performed using gene-specific probes (Table S2) for Southern blot hybridization. Restricted by the limited amount of genomic DNA available, PCR-based methods was used for linkage analysis of banded houndshark *WA_DS10* (Fig. S9*B*) with PCR conditions described above. DNA fragments amplified with the primers DS10-2 and DS10-8 (for primer sequences, see Table S2) were digested with the restriction enzyme *Hha*I to look for haplotype differences within the seventeen littermate banded houndshark individuals (Fig. S9*B* left). Amplification of *WA_DS10* gene with another set of primers, DS10-6 and DS10-14, was also conducted (Fig. S9*B* right). For linkage analyses of the banded houndshark *β_2-m* gene, PCR-based amplification was also performed using primers β_2-m-F11 and β_2-m-R10. Obtained DNA fragments were digested with *Mbo*II restriction enzyme (Fig. S15*A*; *SI Appendix* section 8).

Database searches. Database searches with annotation keywords and similarity searches were conducted using programs such as BLASTN, BLASTP and TBLASTN at NCBI (National Center for Biotechnology Information) (40) or Ensembl (41) Web sites. Specifically for sequence information of little skates and salamanders, SkateBase (42) and SalSite (43) were also used, respectively.

Analyses of DNA and amino acid sequences. The nucleotide sequences that were determined by experiments and sequences retrieved from databases were handled and analyzed with Genetyx software ver. 12 (Genetyx Co. Ltd. Tokyo). For prediction of protein-coding regions in genomic sequences, comparisons with matching cDNA sequences were performed and GENSCAN (44) and FGENESH (45) programs were also used. For prediction of signal peptides, SignalP 4.1 Server (46) was used. For prediction of secondary structures of protein sequences, JPred4 (47) was used.

Alignments of amino acid sequences. Alignments of amino acid sequences were performed with MUSCLE program (48) and also with Genetyx software. Alignments were adjusted based on the previously reported studies (22, 23, 25, 49) and on structural comparisons using the UCSF Chimera package (50). For alignment of the membrane-distal domains of W-category molecules, the positions of conserved and variable residues of the teleost fish W-category molecules were used to compare with other MHC molecules.

Phylogenetic tree analyses. Phylogenetic tree analyses of the MHC Ig-like C1-set domains were conducted with Maximum Likelihood, a tree-searching method, in MEGA6 (51). The best combination of evolutionary model and rates was selected from 56 different amino acid substitution models or from 24 nucleotide substitution models using the log likelihood, and models with the lowest BIC (Bayesian Information Criterion) were considered to describe the substitution pattern the best in MEGA6. For the amino acid sequence analyses, the WAG+G+I model (see below for the abbreviations) was selected and for the DNA sequence analyses, the GTR+G+I model was selected using MEGA6. Abbreviations are as follows: WAG, Whelan And Goldman model (52); +G, a discrete Gamma distribution used to model evolutionary rate differences among sites with

5 rate categories; +I, assuming that a certain fraction of sites are evolutionarily invariable; GTR, General Time Reversible model (53). For the analyses shown in Fig. S19, Neighbor Joining (54), which is an algorithmic method, was conducted in MEGA6. With this method, the JTT (Jones-Taylor-Thornton) matrix model (55) or the p-distance model (53) were used.

Partial deletion was used in the analyses and the tree is drawn to scale, with branch lengths measured in the number of substitutions per site. Reliability of a tree was estimated using Bootstrap method (56) with 1000 bootstrap replications. Percentage of replicate trees in the bootstrap test are indicated next to the nodes (Values of 50 or greater are shown).

For the outgroups in the phylogenetic analyses, Ig-like C1-set domains of three molecules were used: Ig M C_H4 domain (previously used in ref. 28), TCR β chain constant domain (previously used in ref. 10) and tapasin Ig-like domain (e. g., 57) of human and house mouse. The alignments of the outgroup sequences with MHC domains (Dataset S2) were based on previous amino acid sequence comparisons (e. g., 57) and on structural comparisons with the reported structures of mouse IgM (PDB ID: 4JVW), human TCR β chain (PDB ID: 1OGA), and human tapasin (PDB ID: 3F8U) using the Chimera program. The regions between the β-strand-forming sequence blocks which show high sequence-length variation were not included for the comparison and the relevant regions were described in *SI Appendix*.

In Fig. 7, phylogenetic tree analyses are shown with amino acid sequences of Ig-like domains of selected W-category and MHC class I and class II molecules using Maximum Likelihood. In Supplementary Information, additional phylogenetic tree analyses of Ig-like domains are shown as follows: analyses with DNA sequences of selected W-category and MHC class I and class II molecules using Maximum Likelihood (Fig. S16); analyses with amino acid sequences of selected W-category and MHC class I and class II molecules using JTT (Fig. S17*A*) or p-distance (Fig. S17*B*) of Neighbor Joining; analyses of the additional W-category and MHC class I and class II molecules including nonclassical MHC class I and class II molecules using Maximum Likelihood (Fig. S18).

Structural comparisons. Comparative structural molecular analyses were conducted using Cn3D (58) and UCSF Chimera package (50). Figures 4 and S5-S7 were prepared with Cn3D.

Recombinant protein studies. *Cell lines:* CHO (Chinese hamster ovary)-K1 cells were obtained from RIKEN BRC (BioResource Research Center) through the National Bio-Resource Project of the MEXT (the Ministry of Education, Culture, Sports, Science and Technology) of Japan, and grown in Ham's F-12 medium (Wako) containing 10% fetal bovine serum (FBS) in 5% CO_2 at 37℃.

Vector construction and transfection: Signal peptides encoded by *WA* and *WB* genes were predicted using the SignalP 4.1 Server (46). DNA encoding a mature protein of tiger salamander (TS) WA_01 (residues 21-250) was cloned into the pFLAG-CMV-3 Expression Vector (Sigma). DNA encoding a mature protein of TS WB_01 (residues 20-273) or Mexican tetra (MT) WB_01 (residues 23-272) was cloned into the pCAG-Hyg PA tag-N Vector (Wako). The pFLAG-CMV-3 Expression Vector and pCAG-Hyg PA tag-N Vector without insert sequences were used as negative controls (designated pFLAG and pPA, respectively). The following combinations were used for co-transfection experiments: N-terminal FLAG tagged WA (TS FLAG-WA) and N-terminal PA tagged WB (TS PA-WB or MT PA-WB); TS FLAG-WA and pPA; pFLAG and TS PA-WB or MT PA-WB; and pFLAG and pPA vectors. CHO-K1 cells were transfected using Lipofectamine 2000 (Invitrogen) according to the manufacturer's instruction. After 24 h, cells were harvested for flow cytometry and protein extraction.

Protein extraction: The co-transfected cells, a portion of which were analyzed for flow cytometry described below, were lysed using ice-cold lysis buffer containing 50 mM Tris-HCl (pH 7.5), 150 mM NaCl, 5 mM EDTA, 1% NP-40, 10 mM iodoacetamide. After placement on ice for 30 min, mixture was centrifugated at 20,000 x *g* for 20 min at 4℃. The supernatants were used as cell lysates for Western blotting to investigate the expression of recombinant proteins.

Endoglycosydase H and Glycopeptidase F treatments: Lysates from co-transfected cells were prepared as described above. Based on the manufacturer's instructions, the cell lysates prepared under protein denaturing conditions were treated with Endoglycosydase H (Endo H, NEB) or Glycopeptidase F (GPF, Takara Bio).

Western blotting: Equal amounts of total proteins of cell lysates were separated using SDS-PAGE (12% Tris-glycine gel), and transferred to polyvinylidene difluoride membranes. The membranes were incubated with 5% skim milk in PBST (0.05% Tween 20 in PBS) for 1 h. The PBST-washed membranes were incubated with mouse anti DYKDDDDK tag monoclonal antibody or rat anti PA tag monoclonal antibody for 1 h, washed with PBST and then incubated with anti-mouse IgG, HRP-linked whole Ab sheep (GE Healthcare) or goat anti-rat IgG(H+L)-HRP conjugate (SouthernBiotech) for 1 h. The HRP-conjugated antibodies were visualized using Pierce ECL Plus Western Blotting Substrate (Thermo Scientific) on ImageQuant LAS 4000mini (GE Healthcare). The MagicMark XP Western Protein Standard (Invitrogen) was used as protein size marker. After HRP-inactivation with 15% H_2O_2 for 1 h, the membranes were incubated with mouse anti β-actin monoclonal antibody (MBL) for 1 h prior to incubation with anti-mouse IgG, HRP-linked whole Ab sheep (GE Healthcare) for 1 h. Visualization of β-actin was performed as described above.

Flow cytometry: Cells were incubated with primary antibody in FCM buffer (2% FBS and 0.1% sodium azide in PBS) for 1 h on ice. Then cells were washed with FCM buffer and incubated with secondary antibody in FCM buffer for 1 h on ice. The washed cells were suspended in PBS containing propidium iodide for staining of dead cells which were excluded from the analyses. The cells were analyzed using Gallios (Beckman Coulter) with Kaluza v1.3 software (Beckman Coulter). The FLAG tag was detected with mouse anti DYKDDDDK tag monoclonal antibody (Wako) and mouse IgG_{2b} (SouthernBiotech) was used as isotype control. The PA tag was detected with rat anti PA tag monoclonal antibody (Wako) and rat IgG_{2a} (SouthernBiotech) was used as isotype control. The mouse and rat IgG were detected using secondary antibodies Goat $F(ab')_2$ Anti-Mouse IgG(H+L) PE-conjugated (Beckman Coulter) and Goat $F(ab')_2$ Anti-Rat IgG(H+L) FITC-conjugated (SouthernBiotech), respectively.

Analysis of the conservation profile of a W-category subgroup. The amino acid sequences of the membrane-distal domains of the teleost fish W-category molecules (Figs. S1 and S2) were compared and, to understand the conservation profile, Wu-Kabat variability values were calculated as described (59; Table S4). Wu-Kabat variability (V) is defined as the number of different amino acids at a given position (Nd) divided by the frequency of the most common amino acid at that position (Nc/N; Nc, the number of the most common amino acid at that position; N, the number of sequences studied at that position) (59).

Appendix

1. Overview of W-category genes

1-1 Various animal groups possess W-category genes

W-category genes were identified in various jawed vertebrates. Table S1 shows the current identification of W-category genes. The animal groups shown to possess W-category genes include cartilaginous fish (sharks and skates), ray-finned fish (the teleost fish group), lobe-finned fish (coelacanth and lungfish) and tetrapods (salamanders). Thus, the ancestral W-category genes should have existed in the common ancestor of cartilaginous fish and ray-finned fish/lobe-finned fish/tetrapods, estimated to have diverged from each other around 457 million years ago (MYA) (posterior mean and 95 % credible interval of 443-464 MYA according to ref. 60). Therefore, W-category genes should be very ancient members of the MHC gene family.

Although W-category genes can be found in various animal groups, their distribution appears to be somewhat restricted within each group. Some examples are as follows. Cartilaginous fish (class Chondrichthyes) include two Subclasses, Holocephali (e.g., elephant shark) and Elasmobranchii (modern sharks and rays), estimated to have diverged around 421 MYA (410-447 MYA) (60). So far, W-category genes have been identified in Elasmobranchii. Among ray-finned fish, W-category genes have thus far been identified in the teleost fish group which include, for example, herring and zebrafish. The ancestors of ray-finned fish and lobe-finned fish/tetrapods are estimated to have diverged from each other around 419 MYA (416-422 MYA) (60). In lobe-finned fish, W-category genes have been identified in coelacanth and lungfish. The ancestors of lungfish and tetrapods are estimated to have diverged from each other around 392 MYA (375-408 MYA) (60). Among tetrapods, W-category genes have thus far been identified in salamanders. Restricted identification within a certain group may be related to genuine differences in gene inactivation but in some cases possibly also to incompleteness of sequence information or to sequence diversification beyond recognition.

1-2 Nomenclature of W-category genes

<u>1-2-1 General description</u>

Like conventional MHC class II molecules, a W-category molecule is expected to be composed of an α chain and a β chain. In the present study, we name W-category α chain WA, and W-category β chain WB. The assignments of the sequences as an α chain-type or as a β chain-type are supported by their possession of amino acid residues specific for the respective chain and also by phylogenetic analyses (Fig. 2*A* and *B*; Datasets S1-S3; Fig. 7; Figs. S16-S18).

Except for W-category genes of cartilaginous fishes, some teleost fishes (described below) and lobe-finned fishes, simple "WA" and "WB" names for W-category genes are used, because information regarding the relationship among W-category genes is still limited and information about the gene locations is also limited. Numbers were added to the gene names like WA _01 to specify each sequence even when only a single member for the relevant gene has been found. In case of cartilaginous fish, multiple loci were found in some cases and locus identifications remain to be studied. In those cases, additional symbols like "n1", "n2" and so on, were temporarily used to discriminate related sequences of which at least some are expected to be from different loci.

<u>1-2-2 Nomenclature of cartilaginous fish W-category genes</u>

In cartilaginous fish, currently six W-category genes have been identified; three *WA* genes and three *WB* genes. *WA_DS5*, *WA_DS10*, *WB_DS1* and *WB_DS3* were found in banded houndshark, and *WA_Nds3L* and *WB_Nds5L* were recently found in various other sharks. The somewhat complicated nomenclature for these shark W-category genes has historical reasons as explained below.

About banded houndshark WA_DS5, WA_DS10, WB_DS1 and WB_DS3

All *WA* and *WB* genes of banded houndshark were found to be linked in the genome (Fig. S9), but relative positions of individual genes of this shark species remain unknown. In the early phase of the present study (including the part described in ref. 23), fragments of MHC-like genes were found from the banded

houndshark genomic DNA or cDNA, and were named sequentially as *DS1*, *DS2*, *DS3* and so on. After some of them were recognized to belong to W-category genes and could be classified into *WA* or *WB*, they were renamed and their original tentative names were incorporated into the new names as *WB_DS1*, *WB_DS3*, *WA_DS5* and *WA_DS10*. That is the reason why these genes have a somewhat peculiar numerical nomenclature.

Probable numbers of gene loci of banded houndshark W-category genes are as follows (indicated in parentheses): *WA_DS5* (three), *WA_DS10* (one), *WB_DS1* (two) and *WB_DS3* (one). While simple names can be used for *WA_DS10* and *WB_DS3*, a more refined nomenclature is necessary for *WA_DS5* and *WB_DS1* as described below.

A banded houndshark presumably possesses three loci for *WA_DS5* based on isolated cDNAs and Southern blot analyses (Figs. S1A, S3, S9A). Four kinds of cDNA sequences for *WA_DS5* from a single individual banded houndshark N1 were identified. These four sequences were named as *WA_DS5_n1_01*, *WA_DS5_n2_01*, *WA_DS5_n3_01* and *WA_DS5_n4_01* (Fig. S1A). At present, relationships among these *WA_DS5* sequences are only partially clarified.

For *WB_DS1*, there presumably exist two loci in a banded houndshark based on the genomic sequencing and Southern blot analyses (Figs. S2A, S3, S9C and *SI Appendix*). However, complete expressed sequences could be identified from only one of these loci. This gene locus was named *WB_DS1_n1* and the other one was named *WB_DS1_n2*. A cDNA sequence of *WB_DS1_n2* could only be identified in an unusual cDNA form in which parts of *WA_DS10* and *WB_DS1_n2* are combined together (LC218721, Table S3).

About W-category genes of other cartilaginous fishes

Similar to banded houndshark, other cartilaginous fishes also possess *WA_DS5-like*, *WA_DS10-like*, *WB_DS1-like* and *WB_DS3-like* sequences. Names with "*-like*" are used for those sequences with respective homology in the present study. In some cases, "L" instead of "-like" is used to save space.

The recent whole genome shotgun sequencing of great white shark revealed two additional W-category genes, namely, *WA_Nds3L* and *WB_Nds5L* and showed that three *WA* genes and three *WB* genes are located closely to each other forming three pairs of *WA*/*WB* genes (Fig. S11A). These pairs are: *WA_Nds3L*/*WB_DS3L*, *WA_DS10L*/*WB_DS1L* and *WA_DS5L*/*WB_Nds5L*. In the names of *WA_Nds3L* and *WB_Nds5L* , "N" represents "next to", and "L" represents "-like". The genes homologous to *WA_Nds3L* or *WB_Nds5L* could be identified in other cartilaginous fish species as shown in Table S1.

1-2-3 Nomenclature of teleost fish W-category genes

In many teleost fishes (61) possessing W-category genes, only a single pair of intact *WA* and *WB* genes appears to exist. Exceptions are members of a group of Cyprinidae, including common carp and Chinese cavefish, which have two pairs of *WA/WB* genes in the genome (Fig. S11) and which are believed to have experienced a relatively recent additional whole genome duplication approximately 8.2 MYA (62). Therefore, the names *WA_A1* and *WB_B1* are used for one pair and *WA_A2* and *WB_B2* for the other. For the zebrafish W-category genes identified in the NCBI genomic sequence database, we use the names *WA_13A* and *WB_13B* referring to their location on zebrafish chromosome 13. This allows distinction from the zebrafish W-category sequences that were amplified from cDNA and were named zebrafish *WA_01* to *05* and zebrafish *WB_01* to *05* (Fig. S19).

1-2-4 Nomenclature of lobe-finned fish W-category genes

As two distinct groups of *WA* genes were identified in lungfish based on similarity in the Ig-like domains, temporarily *WA_1* and *WA_2* were used for those genes. For *WB* genes, two kinds of *WB* genes were also identified in lungfish, and the names *WB_1* and *WB_2* were used for those genes. The *WB_2* genes could be further classified into two groups, and the names *WB_2_1* and *WB_2_2* were used for those genes. Numerical supplements were used to specify the sequences.

1-2-5 Nomenclature of salamander W-category genes

For the W-category genes of salamanders, simply *WA* and *WB* names were used, and numerical supplements were added like *WA_01* and *WB_01* to specify the sequences.

2. Identification of W-category genes

Various W-category genes were identified through our own experiments and also through database searches using various datasets. Below, accession numbers for sequences are provided where relevant. Accession numbers refer to those jointly used by GenBank at NCBI, DNA DataBank of Japan (DDBJ) and European Nucleotide Archive (ENA) unless mentioned otherwise.

2-1 Cartilaginous fish - modern sharks and skates

Extant Elasmobranchii (Neoselachii) contain two subgroups, Selachii (modern sharks) and Batoidea (rays, skates and sawfish). The divergence time of these two groups is estimated to be around 281 MYA (251-318 MYA) (60).

Three pairs of *WA*/*WB* genes were found in sharks. Two *WA* genes (*WA_DS5* and *WA_DS10*) and two *WB* genes (*WB_DS1* and *WB_DS3*) were isolated from banded houndshark. DNA fragments of *WA_DS5*, *WB_DS1* and *WB_DS3* were originally amplified together in a single PCR reaction, and *WA_DS10* was later discovered in a different way as described below. The homologs of these genes in other cartilaginous fishes were identified through searches of cartilaginous fish sequence databases.

WA_Nds3L and *WB_Nds5L* genes were identified in the whole genome shotgun sequence data of great white shark and then also found in other sequence data of cartilaginous fish.

Below, the identification of W-category genes of cartilaginous fish are described per species.

<u>2-1-1 Banded houndshark (*Triakis scyllium*)</u>

Two *WA* genes (*WA_DS5* and *WA_DS10*) and two *WB* genes (*WB_DS1* and *WB_DS3*) were initially discovered experimantally in banded houndshark. Banded houndshark may have genes similar to *WA_Nds3L* and *WB_Nds5L*, but those have not been identified yet.

2-1-1-1 Banded houndshark *WA_DS5*

A short DNA fragment in the banded houndshark *WA_DS5* Ig-like domain exon was found among the mixture of products (*Ban*I/*Pst*I restriction enzyme-resistant fractions) of the same PCR reaction products from which banded houndshark *WB_DS1* (previously called DI) was found (23). Using this DNA fragment as a probe, the genomic library of banded houndshark was screened and positive clones were isolated, and then the genomic sequence of the Ig-like domain exon of banded houndshark *WA_DS5* was determined. As positive clones could not be obtained in the screening of a banded houndshark cDNA library, the "rapid amplification of cDNA ends (RACE)" method was conducted using WA_DS5-specific primers, and then 5'- and 3'-untranslated region (UTR) sequences were obtained. Using 5'- and 3'-UTR primers, four different cDNA sequences (*WA_DS5_n1_01*, *n2_01*, *n3_01*, and *n4_01*) could be amplified from a single individual of the banded houndshark N1 (similarities of these four sequences is described in section 5). The genomic exon/intron organization of banded houndshark *WA_DS5_n3* was determined as described in the Materials and Methods and shown in Figs. 5 and S8.

2-1-1-2 Banded houndshark *WA_DS10*

As stated above, *WA_DS10* was discovered separately from the other banded houndshark W-category genes, and here some details on its discovery are described.

The banded houndshark *WA_DS10* was found in one cDNA sequence (named F02_14, LC218721, Table S3) among the products of the 5'-RACE reactions for *WB_DS1* gene. This cDNA looked like a read-through fusion product and contained an - until then - unknown Ig-like domain exon which was combined with the Ig-like domain exon of *WB_DS1_n2* (a possible pseudogene, see the section of *WB_DS1*) with an apparently normal splicing junction. Then RACE reactions were conducted using primers specific to this novel Ig-like domain exon sequence. After 5'- and 3'-regions of the cDNA were obtained, the complete coding sequence was obtained. It turned out that this new gene had a domain structure of MHC class II and could be classified as a W-category α chain gene. This gene was named the banded houndshark *WA_DS10*. Two kinds of *WA_DS10* cDNA sequences were identified from the single individual banded houndshark N1 (Table S3). At

amino acid position 40 in Fig. 3, one (*WA_DS10_01*) has an "AAC" codon for an asparagine, the other (*WA_DS10_02*) has "AAT". Otherwise they possess the same open reading frame sequences. The genomic exon/intron organization of banded houndshark *WA_DS10* was determined as described in the Materials and Methods and shown in Fig. S8.

2-1-1-3 Banded houndshark *WB_DS1*

In 1992 we published the DNA sequence of the exon encoding the Ig-like domain of, now named, *WB_DS1* (M85291) (23). However, at the time, we had not yet a concept of W-category genes. Later, one incomplete cDNA sequence of *WB_DS1_n1* was isolated by us from a kidney cDNA library of banded houndshark individual N2 and was registered (AY227971). In the present study, two loci for banded houndshark *WB_DS1*, namely, *WB_DS1_n1* and *WB_DS1_n2* were identified. From *WB_DS1_n1* locus, the complete coding regions of two highly similar presumed allelic sequences, *WB_DS1_n1_01* and *WB_DS1_n1_02* were obtained (Fig. S2) using RT-PCR method based on information obtained from RACE studies. Shorter cDNA forms of both sequences lacking the penultimate CP/TM/CY exon were identified in our experiments and these presumably are alternatively spliced sequences. On the other hand, the β2 domain exon of *WB_DS1_n2* was only found as part of an unusual cDNA (described above in the section for *WA_DS10*) and other *WB_DS1_n2* exons were not detected. The β2 domain exons were compared between *WB_DS1_n1* and *WB_DS1_n2* genes and high similarity between these genes was found in the β2 domain exons and surrounding regions. In the β2 domain exons, there are only three nucleotide differences between *WB_DS1_n1* and *WB_DS1_n2* (e.g. M85291 vs. LC200979, Table S3) and thus the probe consisting of the central portion of the β2 domain exon should be able to detect both genes (Figs. S3, S9 and S10). The genomic region of *WB_DS1_n1* containing a partial β2 domain exon and CP/TM/CY exons was determined using PCR method and the genomic DNA of banded houndshark individual N1 (LC009542). The upstream region of *WB_DS1_n1* β2 domain exon was partially investigated using genomic phage library clones of banded houndshark individual N0 (LC200978). These two genomic sequences were preliminarily combined in Fig. S8. Identification of genomic regions containing the leader and β1 domain exons of *WB_DS1_n1* gene of banded houndshark has not been successful thus far and remains to be investigated.

2-1-1-4 Banded houndshark *WB_DS3*

A short DNA fragment within the banded houndshark *WB_DS3* Ig-like domain was found among the mixture of products (*Ban*I/*Pst*I restriction enzyme-resistant fractions) of the same PCR reaction products from which banded houndshark *WB_DS1* (previously called DI) was found (23), just like the case of banded houndshark *WA_DS5*. Using this DNA fragment as a probe, the genomic library of banded houndshark was screened and positive phage clones were isolated, and then the genomic sequence of banded houndshark *WB_DS3* was determined as described in the Materials and Methods. *WB_DS3* cDNA clones could be isolated from the kidney cDNA library of banded houndshark individual N2, and the sequence of one cDNA clone (*WB_DS3_01*) was determined. A complete coding sequence of banded houndshark *WB_DS3* was amplified with two UTR primers and only a single *WB_DS3* sequence (*WB_DS3_02*) was obtained in banded houndshark individual N1. There are only two amino acid differences between the two obtained *WB_DS3* sequences (Fig. S2). Based on these cDNA sequences, exons in the genomic sequence of *WB_DS3* could be assigned. The genomic exon/intron organization is presented in Fig. 5 and Fig. S8.

<u>2-1-2 Blue shark (*Prionace glauca*)</u>

2-1-2-1 Blue shark *WA_DS5-like*

Blue shark *WA_DS5-like* partial sequences were found through tblastn searches with banded houndshark WA_DS5_n1_01 sequence using SRX1823831 (white muscle tissue) of *Prionace glauca* SRA (Sequence Read Archive, NCBI). Representative sequences are as follows: SRR3632063.60991435.2, SRR3632063.65504350.1, and SRR3632063.57575183.1. Blue shark *WA_DS5-like* partial sequences were also found using SRX3298485 (male immature kidney) of *Prionace glauca* SRA. Representative sequences are as follows: SRR6188468.25604256.2, SRR6188468.25604256.1, and SRR6188468.12728785.1.

2-1-2-2 Blue shark *WA_DS10-like*

Blue shark *WA_DS10-like* sequence was found in GFYY01081745.1 (corresponding to the amino acid residues 15-143 of banded houndshark WA_DS10_01) and GFYY01081744.1 (139-243) through tblastn searches with banded houndshark WA_DS10_01 sequence using transcriptome shotgun assembly (TSA) databases of cartilaginous fishes. For Fig. S1, a hypothetical combined sequence was used, which is composed of GFYY01081745.1 for the partial α1 domain and GFYY01081744.1 for the α2 domain and CP/TM/CY region as blue shark WA_DS10_like sequence.

2-1-2-3 Blue shark *WA_Nds3L*

Blue shark *WA_Nds3L* sequence was found in GFYY01021401.1 through tblastn searches with great white shark WA_Nds3L sequence using TSA databases of cartilaginous fishes.

2-1-2-4 Blue shark *WB_DS1-like*

Blue shark *WB_DS1-like* sequence was found in GFYY01012278.1 (corresponding to the amino acid residues 1-282 of banded houndshark WB_DS1_01) through tblastn searches with banded houndshark WB_DS1_01 sequence using TSA databases of cartilaginous fishes.

2-1-2-5 Blue shark *WB_DS3-like*

Blue shark *WB_DS3-like* sequence was found in GFYY01033062.1 (corresponding to the amino acid residues 1-137 of banded houndshark WB_DS3_01) through tlastn searches with banded houndshark WB_DS3_01 sequence using TSA databases of cartilaginous fishes.

2-1-2-6 Blue shark *WB_Nds5L*

Blue shark *WB_Nds5L* sequence was found in GFYY01040305.1 (corresponding to the amino acid residues 12-83 of great white shark WB_Nds5L) through tblastn searches with great white shark WB_Nds5L sequence using TSA databases of cartilaginous fishes.

<u>2-1-3 Cloudy catshark (*Scyliorhinus torazame*)</u>

Two pairs of W-category genes, *WA_DS5-like*/*WB_Nds5L* and *WA_DS10-like*/*WB_DS1-like*, were found in a 159 K contig, and *WA_DS5-like*/*WB_Nds5L* pair was found in a 82 K contig, as described below.

2-1-3-1 Cloudy catshark *WA_DS5-like*

Cloudy catshark *WA_DS5-like* sequence was found in BFAA01007584.1 (159 K contig, 159043 bp) through tblastn searches with banded houndshark WA_DS5_n1_01 sequence using whole genome shotgun (wgs) databases of cartilaginous fishes which include Cloudy catshark WGS Project BFAA01. In this 159 K contig, *WA_DS5-like* and *WB_Nds5L* genes exist as a pair (head to head configuration with respect to the transcriptional orientations; 4185 bp between the two methionine-coding start codons) as shown in Fig. S11B. Cloudy catshark *WA_DS5-like* sequence was also found in BFAA01011934.1 (82 K contig, 82266 bp) through tblastn searches with banded houndshark WA_DS5_n1_01 sequence using wgs databases of cartilaginous fishes. In this 82 K contig, *WA_DS5-like* and *WB_Nds5L* genes exist as a pair (head to head configuration with respect to the transcriptional orientations; 3059 bp between the two methionine-coding start codons) as shown in Fig. S11B.

Amino acid identity % between two WA_DS5-like sequences from two different contigs of cloudy catshark are as follows:

Between cloudy catshark WA_DS5-like_82K vs. cloudy catshark WA_DS5-like_159K, 36 % for α1 domain and 64 % for α2 domain.

Amino acid identity % between WA_DS5-like sequences of cloudy catshark and great white shark are as follows:

Between cloudy catshark WA_DS5-like_82K vs. great white shark WA_DS5-like, 63 % for α1 and 79 % for α2.
Between cloudy catshark WA_DS5-like_159K vs. great white shark WA_DS5-like, 45 % for α1 and 70 % for α2.
WA_DS5-like sequence of cloudy catshark 82 K contig showed higher amino acid identity % with those of other sharks compared to WA_DS5-like sequence of cloudy catshark 159 K contig. Similar relationships were observed in case of WB_Nds5L of two cloudy catshark contigs.

2-1-3-2 Cloudy catshark *WA_DS10-like*
Cloudy catshark *WA_DS10-like* sequence was found in BFAA01007584.1 (159 K, 159043 bp) through tblastn searches with banded houndshark WA_DS10_01 sequence using wgs databases of cartilaginous fishes. In this 159 K contig, *WA_DS10-like* and *WB_DS1-like* genes exist as a pair (head to head configuration with respect to the transcriptional orientations; 272 bp between the two methionine-coding start codons) as shown in Fig. S11B.

2-1-3-3 Cloudy catshark *WB_DS1-like*
Cloudy catshark *WA_DS1-like* sequence was found in BFAA01007584.1 (159 K, 159043 bp) through tblastn searches with banded houndshark WA_DS1_01 sequence using wgs databases of cartilaginous fishes. *WA_DS1-like* and *DS10-like* genes exist as a pair as described above.

2-1-3-4 Cloudy catshark *WB_Nds5L*
Cloudy catshark *WB_Nds5L* sequence was found in BFAA01007584.1 (159 K, 159043 bp) through tblastn searches with banded houndshark WB_DS1_01 sequence using wgs databases of cartilaginous fishes. Cloudy catshark *WB_Nds5L* sequence was also found in BFAA01011934.1 (82 K, 82266 bp; for β2 domain) through tblastn searches with banded houndshark WB_DS3_01 sequence using wgs databases of cartilaginous fishes. *WB_Nds5L* and *WA_DS5-like* genes exist as a pair in both contigs as described above.

Amino acid identity % between two WB_Nds5L sequences from two different contigs of cloudy catshark are as follows:
Between cloudy catshark WB_Nds5L_82K vs. cloudy catshark WB_Nds5L_159K, 39 % for β1 domain and 84 % for β2 domain.

Amino acid identity % between WB_Nds5L sequences of cloudy catshark and great white shark are as follows:
Between cloudy catshark WB_Nds5L_82K vs. great white shark WB_Nds5L, 63 % for β1 and 73 % for β2.
Between cloudy catshark WB_Nds5L_159K vs. great white shark WB_Nds5L, 36 % for β1 and 66 % for β2.
WB_Nds5L sequence of cloudy catshark 82 K contig showed higher amino acid identity % with those of other sharks compared with WB_Nds5L sequence of cloudy catshark 159 K contig. Similar relationships were observed in case of WA_DS5-like of two cloudy catshark contigs as described above.

2-1-4 Great white shark (*Carcharodon carcharias*)
In a 285 K contig of great white shark, three pairs of W-category genes were found: *WA_Nds3L*/*WB_DS3-like*, *WA_DS10-like* /*WB_DS1-like* and *WA_DS5-like* /*WB_Nds5L*.

2-1-4-1 Great white shark *WA_DS5-like*
Great white shark *WA_DS5-like* sequence was found in QUOW01001706.1 (285 K, 285375 bp) through tblastn searches with banded houndshark WA_DS5_n1_01 sequence using wgs databases of cartilaginous fishes. In this 285 K contig, *WA_DS5-like* and *WB_Nds5L* genes exist as a pair (head to head configuration with respect to the transcriptional orientations; 1790 bp between the two methionine-coding start codons) as shown in Fig. S11A.

2-1-4-2 Great white shark *WA_DS10-like*

Great white shark *WA_DS10-like* sequence was found in QUOW01001706.1 (285 K, 285375 bp) through tblastn searches with banded houndshark WA_DS10_01 sequence using wgs databases of cartilaginous fishes. In this 285 K contig, *WA_DS10-like* and *WB_DS1-like* genes exist as a pair (head to head configuration with respect to the transcriptional orientations; 275 bp between the two methionine-coding start codons) as shown in Fig. S11A.

2-1-4-3 Great white shark *WA_Nds3L*
Great white shark *WA_ Nds3L* sequence was found in QUOW01001706.1 (285 K, 285375 bp) through tblastn searches with banded houndshark WA_DS5_n1_01 sequence using wgs databases of cartilaginous fishes. In this 285 K contig, *WA_Nds3L* and *WB_DS3-like* genes exist as a pair (head to head configuration with respect to the transcriptional orientations; 1742 bp between the two methionine-coding start codons) as shown in Fig. S11A. *WA_Nds3L* gene in this contig lacks a single nucleotide (G) near the middle of the α1 domain exon, which produces a frame-shift, compared to the expressed sequences (e.g., SRA: SRR684727.41156647.1 of SRX228421). In Fig. S1 and Dataset S1, the corrected sequence for great white shark WA_Nds3L is presented.

2-1-4-4 Great white shark *WB_DS1-like*
Great white shark *WA_DS1-like* sequence was found in QUOW01001706.1 (285 K, 285375 bp) through tblastn searches with banded houndshark WB_DS1_ 01 sequence using wgs databases of cartilaginous fishes. *WA_DS1-like* and *DS10-like* genes exist as a pair as described above.

2-1-4-5 Great white shark *WB_DS3-like*
Great white shark *WA_DS3-like* sequence was found in QUOW01001706.1 (285 K, 285375 bp) through tblastn searches with banded houndshark WB_DS3_01 sequence using wgs databases of cartilaginous fishes. *WB_DS3-like* and *WA_Nds3L* genes exist as a pair as described above.

2-1-4-6 Great white shark *WB_Nds5L*
Great white shark *WA_Nds5L* sequence was found in QUOW01001706.1 (285 K, 285375 bp) through tblastn searches with banded houndshark WB_DS3_01 sequence using wgs databases of cartilaginous fishes. *WB_Nds5L* and *WA_DS5-like* genes exist as a pair as described above.

2-1-5 Whale shark (*Rhincodon typus*)
2-1-5-1 Whale shark *WA_DS5-like*
Whale shark *WA_DS5-like* sequences were found in LVEK01588988.1 (for α1 domain) and LVEK01625365.1 (for α2 domain) through tblastn searches with banded houndshark WA_DS5_n1_01 sequence using wgs databases of cartilaginous fishes. In Fig. S1, as a whale shark WA_DS5-like sequence, a hypothetical combination of sequences was used, which is composed of LVEK01588988.1 for the α1 domain and LVEK01625365.1 for the α2 domain.

2-1-5-2 Whale shark *WA_DS10-like*
Whale shark *WA_DS10-like* sequences were found in LVEK01757095.1 (for α1 domain) and LVEK01573532.1 (for α2 domain) through tblastn searches with banded houndshark WA_DS10_01 sequence using wgs databases of cartilaginous fishes. In Fig. S1, as a whale shark WA_DS10-like sequence, a hypothetical combination of sequences was used, which is composed of LVEK01757095.1 for the α1 domain and LVEK01573532.1 for the α2 domain.

2-1-5-3 Whale shark *WB_DS1-like*
Whale shark *WB_DS1-like* sequence was found in LVEK01262083.1 (for β2 domain) through tblastn searches with banded houndshark WB_DS1_01 sequence using wgs databases of cartilaginous fishes.

2-1-5-4 Whale shark *WB_DS3-like*

Whale shark *WB_DS3-like* sequence was found in LVEK01746520.1 (for β1 domain) through tblastn searches with banded houndshark WB_DS3_01 sequence using wgs databases of cartilaginous fishes. This β1 domain possesses a cysteine residue highly characteristic for WB_DS3.

<u>2-1-6 Whitespotted bambooshark (*Chiloscyllium plagiosum*)</u>
2-1-6-1 Whitespotted_bambooshark *WA_DS5-like*
Whitespotted bambooshark *WA_DS5-like* sequences were found in QPFF01095956.1 (18K, 18685 bp; for α1 and α2 domains), QPFF01329347.1 (657 bp; for α2 domain), and BEZZ01383420.1 (1297 bp; for α1 domain), through tblastn searches with banded houndshark WA_DS5_n1_01 sequence using wgs databases of cartilaginous fishes.

2-1-6-2 Whitespotted bambooshark *WA_DS10-like*
Whitespotted bambooshark *WA_DS10-like* sequence was found in QPFF01486458.1 (8K, 8625 bp; for α1 and α2 domains, and CP/TM/CY region), in QPFF01329347.1 (657 bp; for α2 domain), and BEZZ01383420.1 (1297 bp; for α1 domain), through tblastn searches with banded houndshark WA_DS5_n1_01 sequence using wgs databases of cartilaginous fishes.

2-1-6-3 Whitespotted bambooshark *WA_Nds3L*
Whitespotted bambooshark *WA_Nds3L* sequence was found in QPFF01560724.1 (1417 bp; for α2 domain) through tblastn searches with great white shark WA_Nds3L sequence using wgs databases of cartilaginous fishes.

2-1-6-4 Whitespotted bambooshark *WB_DS1-like*
Whitespotted bambooshark *WB_DS1-like* sequence was found in QPFF01495858.1 (705 bp; for β2 domain) through tblastn searches with banded houndshark WB_DS1_01 sequence using wgs databases of cartilaginous fishes.

2-1-6-5 Whitespotted bambooshark *WB_DS3-like*
Whitespotted bambooshark *WB_DS3-like* sequence was found in QPFF01327377.1 (543 bp; for β1 domain) and QPFF01162766.1 (4608 bp; for β2 domain) through tblastn searches with banded houndshark WB_DS3_01 sequence using wgs databases of cartilaginous fishes.

2-1-6-6 Whitespotted bambooshark *WB_Nds5L*
Whitespotted bambooshark *WB_Nds5L* sequences were found in QPFF01130085.1 (1921 bp; for β1 domain) and QPFF01212593.1 (1887 bp; for β1 domain) through tblastn searches with great white shark WB_Nds5L sequence using wgs databases of cartilaginous fishes.

<u>2-1-7 Brownbanded bambooshark (*Chiloscyllium punctatum*)</u>
2-1-7-1 Brownbanded bambooshark *WA_DS5-like*
Brownbanded bambooshark *WA_DS5-like* sequences were found in BEZZ01010960.1 (3420 bp; for α1 domain), BEZZ01017577.1 (3066 bp; for α1 domain), and BEZZ01115237.1 (1187 bp; for α1 domain), through tblastn searches with banded houndshark WA_DS5_n1_01 sequence using wgs databases of cartilaginous fishes.

2-1-7-2 Brownbanded bambooshark *WA_DS10-like*
Brownbanded bambooshark *WA_DS10-like* sequence was found in BEZZ01254771.1 (544 bp; for α1 domain), and in BEZZ01004761.1 (22649 bp; for α2 domain and CP/TM/CY region) through tblastn searches with banded houndshark WA_DS10_01 sequence using wgs databases of cartilaginous fishes. This 22 K contig also contains the exons for β1 and β2 domains of *WB_DS3-like* gene.

2-1-7-3 Brownbanded bambooshark *WA_Nds3L*

Brownbanded bambooshark *WA_Nds3L* sequence was found in BEZZ01099794.1 (1357 bp; for α2 domain) through tblastn searches with great white shark WA_Nds3L sequence using wgs databases of cartilaginous fishes.

2-1-7-4 Brownbanded bambooshark *WB_DS1-like*
Brownbanded bambooshark *WB_DS1-like* sequence was found in BEZZ01084999.1 (1551 bp; for β2 domain) through tblastn searches with banded houndshark WB_DS1_01 sequence using wgs databases of cartilaginous fishes.

2-1-7-5 Brownbanded bambooshark *WB_DS3-like*
Brownbanded bambooshark *WB_DS3-like* sequence was found in BEZZ01004761.1 (22649 bp; for β1 and β2 domains) through tblastn searches with banded houndshark WB_DS3_01 sequence using wgs databases of cartilaginous fishes. This 22 K contig also contains partial *WA_DS10-like* sequence as described above.

2-1-7-6 Brownbanded bambooshark *WB_Nds5L*
Brownbanded bambooshark *WB_Nds5L* sequences were found in BEZZ01198256.1 (690 bp; for β1 domain), BEZZ01164644.1 (829 bp; for β1 domain), BEZZ01050759.1 (2168 bp; for β1 domain), BEZZ01176133.1 (775 bp; for β2 domain) and BEZZ01162851.1 (838 bp; for β2 domain) through tblastn searches with great white shark WB_Nds5L sequence using wgs databases of cartilaginous fishes.

2-1-8 Zebra bullhead shark (*Heterodontus zebra*)
2-1-8-1 Zebra bullhead shark *WA_DS5-like*
Zebra bullhead shark *WA_DS5-like* sequence was found in GGGL01449145 through tblastn searches with banded houndshark WA_DS5_n1_01 sequence using TSA databases of catilaginous fishes. This TSA sequence is an apparent readthrough transcript and also contains *WA_DS10-like* sequence (without a starting ATG codon for methionine, but with a stop codon), followed by a complete coding sequence for *WA_DS5-like*.

The sequences with the following accession numbers also contain *WA_DS5-like* sequence, and the numbers with asterisks (*) contain *WA_DS10-like* as well:
GGGL01449115*, GGGL01449117, GGGL01449119*, GGGL01449123, GGGL01449125, GGGL01449130*, GGGL01449131*, GGGL01449132*, GGGL01449136*, GGGL01449137, GGGL01449140, and GGGL01449146.

2-1-8-2 Zebra bullhead shark *WA_DS10-like*
Zebra bullhead shark *WA_DS10-like* sequence was found in GGGL01449113 through tblastn searches with banded houndshark WA_DS10_01 sequence using TSA databases of catilaginous fishes. This TSA sequence as well as the sequences described below do not contain an ATG start codon for *WA_DS10-like*.

The sequences with the following accession numbers also contain *WA_DS10-like* sequence, and the sequences with the numbers with asterisks (*) contain *WA_DS5-like* sequences which were already described in the preceding section:
GGGL01449114, GGGL01449115*, GGGL01449119*, GGGL01449130*, GGGL01449131*, GGGL01449132*, GGGL01449133, GGGL01449135, GGGL01449136*, and GGGL01449145*.

2-1-8-3 Zebra bullhead shark *WB_DS1-like*
Zebra bullhead shark *WB_DS1-like* sequence was found in GGGL01058883 through tblastn searches with banded houndshark WB_DS1_01 sequence using TSA databases of catilaginous fishes.

The sequences with the following accession numbers also contain *WA_DS1-like* sequence:
GGGL01058879, GGGL01058880, GGGL01058881, GGGL01058882, GGGL01058886, and GGGL01058887.

2-1-8-4 Zebra bullhead shark *WB_Nds5L*

Zebra bullhead shark *WB_Nds5L* sequences were found in GGGL01449143, GGGL01449112 and GGGL01449138 as three representatives through tblastn searches with great white shark WB_Nds5L sequence using TSA databases of catilaginous fishes.

<u>2-1-9 Spiny dogfish (*Squalus acanthias*)</u>

2-1-9-1 Spiny dogfish *WA_DS5-like*

Spiny dogfish *WA_DS5-like* sequence was found in HAGW01089906 through tblastn searches with banded houndshark WA_DS5_n1_01 sequence using TSA databases of cartilaginous fishes. This TSA sequence is an apparent readthrough transcript and also contains *WB_DS1-like* sequence, followed by *WA_DS5-like* sequence without a proper sequence for a leader region. In Fig. S1, the predicted amino acid sequence based on HAGW01089906 is shown.

The sequences with the following accession numbers also contain *WA_DS5-like* sequence, and the sequences with the numbers with asterisks (*) contain *WB_DS1-like* sequences as well: HAGW01089902*, HAGW01089904*, HAGW01089907*, HAGW01089908*, HAGW01089910*, HAGT01020445, HAGT01100681, and HAGV01095271.

The predicted amino acid sequence of HAGT01020445 is similar to banded houndshark WA_DS5_n1_01 sequence, and those of the other sequences are similar to banded houndshark WA_DS5_n1_04 sequence.

2-1-9-2 Spiny dogfish *WA_DS10-like*

Spiny dogfish *WA_DS10-like* sequence was found in HAGT01122485 (partial α1, α2 and partial TM) through tblastn searches with banded houndshark WA_DS10_01 sequence using TSA databases of cartilaginous fishes.

2-1-9-3 Spiny dogfish *WA_Nds3L*

Spiny dogfish *WA_Nds3L* sequence was found in HAGT01004242 (311bp; α2 domain) through tblastn searches with great white shark WA_Nds3L sequence using TSA databases of cartilaginous fishes.

2-1-9-4 Spiny dogfish *WB_DS1-like*

Several EST sequences of spiny dogfish were found through tblastn searches with banded houndshark WB_DS1_01 using “expressed sequence tags (est)” databases; these sequences included spiny dogfish *WB_DS1-like1* and *WB_DS1-like2* from pooled multiple tissues of spiny dogfish, and their accession numbers are listed in Table S3. All these sequences lack the 3’ portion of the gene. The longer one (spiny dogfish *WB_DS1-like1*) contains a partial CP/TM/CY region.

Spiny dogfish *WB_DS1*-like sequence (*WB_DS1-like3*) was also found in HAGW01089902 through tblastn searches with banded houndshark WB_DS1_01 sequence using TSA databases of cartilaginous fishes. This longest TSA sequence is an apparent readthrough transcript and also contains *WA_DS5-like* sequence as described in the previous section. In Fig. S2, the predicted amino acid sequence based on HAGW01089902 is shown.

The sequences with the following accession numbers also contain *WB_DS1-like* sequences followed by incomplete *WA_DS5-like* sequences:
HAGW01089904, HAGW01089906, HAGW01089907, HAGW01089908, and HAGW01089910.

2-1-9-5 Spiny dogfish *WB_DS3*-like

Several EST sequences of spiny dogfish were found through tblastn searches with banded houndshark *WB_DS3_01* sequence using est databases; these sequences included spiny dogfish *WB_DS3-like1*, *WB_DS3-like2* and *WB_DS3-like3* as representatives from pooled multiple tissues of spiny dogfish, and their accession numbers are listed in Table S3. All these sequences lack most of the 3’ portion of the gene. The longest one (spiny dogfish *WB_DS3-like1*) contains a partial β2 domain sequence as shown in Fig. S2.

2-1-9-6 Spiny dogfish *WB_Nds5L*

Spiny dogfish *WB_Nds5L* sequence was found in HAGT01000336 (329 bp; β1 domain) through tblastn searches with great white shark WB_Nds5L sequence using TSA databases of cartilaginous fishes.

<u>2-1-10 Little skate (*Leucoraja erinacea*)</u>

2-1-10-1 Little skate *WA_DS5-like*

Little skate *WA_DS5-like* sequences were found in AESE012173146 (for the α1 domain) and AESE011681578 (for the α2 domain) through tblastn search with banded houndshark WA_DS5_n1_01 sequence using wgs databases of cartilaginous fishes. One cDNA sequence (Sequence ID: gnl|SRA|SRR088619.19134478.1) was found through tblastn searches with the translated sequence of AESE012173146 using "sequence read archive (SRA)" dataset SRX036536, and this cDNA sequence contains a short overlap region between the α1 and α2 domain-exons as described above. A tentative leader sequence was obtained through tblastn searches with AESE012173146 (α1 domain) using SRX036536 mentioned above. In Fig. S1, a hypothetical combination of sequences was used based on genomic sequence information of a tentative leader sequence, the α1 domain (AESE012173146) and the α2 domain (AESE011681578).

2-1-10-2 Little skate *WA_DS10-like*

Little skate *WA_DS10-like* gene sequences corresponding to the α1 domain, the α2 domain and the CP/TM/CY region were found as follows.

Little skate *WA_DS10-like* sequence was found in AESE011710435 (for the α2 domain) through tblastn searches with banded houndshark WA_DS10_01 sequence using wgs databases of cartilaginous fishes. A partial α1 and α2 domain-corresponding cDNA sequence (LittleSkate_ TranscriptomeContig95319) was found through tblastn searches with banded houndshark WA_DS-10_01 sequence using Little Skate Transcriptomic Contigs-Build 2 at the SkateBase (42) (http://skatebase.org/skateBLAST). A complete sequence for the α1 domain of little skate *WA_DS10*-like gene was found in AESE011594527 (corresponding to LittleSkate_Consensusfrom Contig1715029 at the SkateBase) through tblastn searches with the partial α1 domain sequence using wgs database of little skate. A candidate sequence for CP/TM/CY region was found in AESE012658604 (LittleSkate_ConsensusfromContig2822493 at the SkateBase) through tblastn searches with LittleSkate_TranscriptomeContig99288 which contains a partial sequence of α2 domain and CP/TM/CY region using Little Skate Transcriptomic Contigs-Build 2 at the SkateBase. In Fig. S1, a hypothetical combination of sequences was used, which is composed of three genomic regions, namely, the α1 domain (AESE011594527), the α2 domain (AESE011710435) and CP/TM/CY region (AESE012658604).

2-1-10-3 Little skate *WB_DS1-like*

Little skate *WB_DS1-like* sequence was found in AESE011933439 (for the β2 domain) through tblastn searches with banded houndshark WB_DS1_n1_01 sequenc using wgs databases of cartilaginous fishes. A cDNA sequence corresponding to a partial β1 and β2 domain (LittleSkate_ TranscriptomeContig44319) was found through tblastn searches with banded houndshark WB_DS1_n1_01 sequence using Little Skate Transcriptomic Contigs-Build 2 at the SkateBase. In Fig. S2, a hypothetical combined sequence was used, which is composed of a cDNA sequence (LittleSkate_TranscriptomeContig44319, for the partial β1 domain) and a genomic sequence (AESE011933439, for the β2 domain).

2-1-10-4 Little skate *WB_Nds5L*

Little skate *WB_Nds5L* sequence was found in AESE011737676.1 (328 bp; for β2 domain) through tblastn search with great white shark WB_Nds5L sequence using wgs databases of cartilaginous fishes.

2-2 Ray-finned fish - teleost fish group

<u>2-2-1 Japanese grenadier anchovy (*Coilia nasus*)</u>

Japanese grenadier anchovy *WA* and *WB* sequences were found in GFON01059655.1 (encoding α1 and partial α2 domain of WA) and GFON01085854.1 (encoding WB) through tblastn searches with Atlantic herring WA_01 and WB_01 sequences, respectively, using TSA databases of bony fishes.

2-2-2 Atlantic herring (*Clupea harengus*)
Atlantic herring *WA* and *WB* sequences were initially found in 34 K contig and further recently identified in an extended 344 K contig as described below.

Atlantic herring *WA* and *WB* sequences were found in JZKK01005006 (34 K contig, 34278 bp) and also in OOIJ01000439 ("344 K contig", 344569 bp, Fig. S11C) through tblastn searches with zebrafish WA_13A and WB_13B sequences, respectively, using wgs databases of bony fishes. 344 K contig completely covers 34 K contig. *WA* and *WB* genes exist as a pair (head to head configuration with respect to the transcriptional orientations; 118 bp between the two methionine-coding start codons) as shown in Fig. S11C. Other than this *WA/WB* pair, an exon fragment of WA α2 is also detected (Fig. S11C). Notable about Atlantic herring WA molecule is that the otherwise highly conserved canonical second cysteine of the α2 domain is replaced with a phenylalanine. This replacement was also observed in the WA α2 domain fragment and observed in SRA genomic sequences (e. g., gnl|SRA|SRR611608.17870700.1 of SRX203066). cDNA sequence information of Atlantic herring W-category genes is not available at present.

2-2-3 Sardine (*Sardina pilchardus*)
Sardine *WA* sequence was found in GGSC01089055, GGSC01089060, GGSC01089061 and GGSC01089054, GGSC01089059 (corresponding to WA α2 domain) and GGSC01089058 (also corresponding to WA α2 domain) through tblastn searches with Atlantic herring WA_01 sequence using TSA databases of bony fishes.

Sardine WB partial sequences were found through tblastn searches with Atlantic herring WB_01 sequence using SRX3593722 (liver RNA) of sardine SRA. Representative sequences are as follows: SRR6505116.71659865.1, SRR6505116.16871074.2, and SRR6505116.16871074.1.

2-2-4 Allis shad (*Alosa alosa*)
Allis shad *WA* and *WB* sequences were found in GETY01010370 (for *WA*), GETY01018251 (for *WB*) and GETY01036995 (for *WB*) through tblastn searches with zebrafish WA_13A for WA and WB_13B for WB, respectively, using TSA databases of bony fishes. Allis shad WA sequence possesses the canonical second cysteine in the α2 domain, which is different from the case of Atlantic herring, although both allis shad and Atlantic herring belong to the family Clupeidae.

2-2-5 Alewife (*Alosa pseudoharengus*)
Alewife *WA* and *WB* sequences were found in GFCK01040569.1 (encoding partial α2 and CP/TM/CY of WA) and GFCK01026404.1 (encoding WB) through tblastn searches with Atlantic herring WA_01 and WB_01 sequences, respectively, using TSA databases of bony fishes.

2-2-6 Hilsa shad (*Tenualosa ilisha*)
Hilsa shad WA and WB sequences were found in QYSC01123695 ("325 K contig", 325703 bp, Fig. S11C) through tblastn searches with Atlantic herring WA_01 and WB_01 sequences using wgs databases of bony fishes. *WA* and *WB* genes exist as a pair (head to head configuration with respect to the transcriptional orientations; 11593 bp between the two methionine-coding start codons) as shown in Fig. S11C. Hilsa shad WA sequence also possesses the canonical second cysteine in the α2 domain like allis shad WA, which is different from the case of Atlantic herring,

2-2-7 Oriental weatherfish (weather loach, mud loach) (*Misgurnus anguillicaudatus*)
Oriental weatherfish *WA* and *WB* sequences were found in GAAD01002023 (encoding a partial α1 domain, a complete α2 domain and a partial CP/TM/CY region of WA) and GAAD01004277 (encoding a partial β2 domain and a complete CP/TM/CY region of WB) through tblastn searches with zebrafish WA_13A and WB_13B, respectively, using TSA databases of bony fishes. Since GAAD01004277 lacks sequences corresponding to the amino-terminal portion (eleven amino acid residues) of the β2 domain, we assembled a presumed complete β2 domain coding sequence using SRA transcriptomic database information (e. g., SRA:SRR3064024.68986258.1 of SRX1479505) and used this sequence in the analyses.

2-2-8 Spined loach (*Cobitis taenia*)
Spined loach *WA* sequence was found in GGJF01002240 (encoding partial α1, α2 and CP/TM/CY of WA) through tblastn searches with zebrafish WA_13A sequence using TSA databases of bony fishes. Spined loach *WB* sequence has not been obtained yet.

2-2-9 White sucker (*Catostomus commersonii*)
White sucker *WA* and *WB* sequences were found in GECX01063527 (for *WA*) and GECX01114064 (for *WB*) through tblastn searches with zebrafish WA_13A and WB_13B sequences, respectively, using TSA databases of bony fishes. The white sucker *WB* sequence of GECX01114064 lacks the beginning of the leader sequence and the last part of the CP/TM/CY portion.

2-2-10 Common carp (*Cyprinus carpio*)
For genomic sequence information of common carp, the NCBI database contains datasets of two whole genome sequencing projects, BioProject PRJEB7241 and BioProject PRJNA73579. The two projects sequenced the genome of common carp using different individuals and the two sources showed similar but not identical results. In the common carp genome, we could identify two *WA/WB* pairs, namely, *WA_A1/WB_B1* and *WA_A2/WB_B2*, in both datasets. Between common carp WA_A1 and WA_A2, amino acid sequence identity is 93 % in the α2 domain, and between common carp WB_B1 and WB_B2, amino acid sequence identity is 81 % in the β2 domain. In addition, fragments of *WA* and *WB* genes were identified. In Figs. S11E and S11F, the genomic locations of these common carp W-category genes and the fragments are summarized.

Below, identifications of W-category sequences in common carp are described separately for the two different BioProject sources.

Within BioProject PRJEB7241 (62), common carp *WA* and/or *WB* sequences were found in LN590696 ("26 M contig" in Fig. S11E, LG32, 26100072 bp), LN598268 ("890 K contig", 890226 bp, 26 M contig includes this 890 K contig region), LN594648 ("138 K contig" in Fig. S11E, 138244 bp) and LN590685 ("20 M contig" in Fig. S11F, LG16, 20606094 bp) through tblastn searches with zebrafish WA_13A and WB_13B sequences using the database of nucleotide collection. The 26 M contig and 890 K contig each contain a *WA_A1/WB_B1* gene pair (head to head configuration with respect to the transcriptional orientations; 294 bp between the two methionine-coding start codons), while the 138 K contig contains a *WA_A2/WB_B2* gene pair (head to head configuration with respect to the transcriptional orientations; 331 bp between the two methionine-coding start codons) (Fig. S11E). The 138 K contig additionally contains a *WB_B2* CP/TM/CY fragment (Fig. S11E) and the 20 M contig contains a *WA_A1* α2 domain exon fragment (Fig. S11F). Compared with presumed intact genes, *WA_A1* of the 26 M/890 K contigs lacks a single nucleotide in the α1 domain exon, and *WB_B1* of these contigs has an extra nucleotide in the leader exon. *WA_A2* of the 138 K contig appears intact while *WB_B2* of this contig has two extra nucleotides in the β2 domain exon. These deviations from presumed intact sequences may either represent actual gene inactivations or sequencing/assembly errors.

Within BioProject PRJNA73579 (63, 64), common carp *WA/WB* sequences were found in LHQP01015169 ("107 K contig", 107664 bp) and LHQP01028415 ("82 K contig" in Fig. S11E, 82519 bp) through tblastn searches with zebrafish WA_13A and WB_13B sequences using wgs databases of bony fishes. The 107 K contig contains the *WA_A1/WB_B1* gene pair (head to head configuration with respect to the transcriptional orientations; 294 bp between the two methionine-coding start codons) while the 82 K contig contains the *WA_A2/WB_B2* gene pair (head to head configuration with respect to the transcriptional orientations; 333 bp between the two putative start codon positions). In the middle of the α2 domain exon of *WA_A1* of the 107 K contig, inversion and other changes of DNA sequences are observed, while *WB_B1* of this contig appears intact. *WA_A2* of the 82 K contig appears to be intact while *WB_B2* of this contig has a single nucleotide change for the methionine-coding start codon. Again, these deviations from presumed intact sequences may either represent actual gene inactivations or sequencing/assembly errors.

In the present study, for comparisons with sequences of other species, apparently intact *WA_A2* of the 82 K contig was used for the common carp *WA_A2_01*, and *WB_B1* of the 107 K contig was used for the common

carp *WB_B1_01*. For the common carp *WA_A1* and *WB_B2*, putatively correct sequences were assembled as information about short genomic sequences of *WA_A1* and *WB_B2* genes are available from SRA of the common carp strain Songpu in BioProject PRJEB7241, which is the same strain as that used to make the draft genome in the same project (ref. 62) and, based on those SRA sequences, the portions of available W-category sequences of the common carp draft genome that were suggested defective appeared to be intact instead. The corrected version of *WA_A1* of the 26 M/890 K contigs was made for the common carp *WA_A1* after the confirmation with the genomic DNA sequences of the common carp strain Songpu (e. g., SRA:SRR924320.37964842.2 of SRX316954, Sp1). Similarly the corrected version of *WB_B2* of the 138 K contig was made for the common carp *WB_B2* after the confirmation with the genomic DNA sequences (e. g., SRA:SRR924320.1454377.1 of SRX316954, Sp1). There are four different SRA datasets from strain Songpu Sp1, Sp2, Sp3 and Sp4. SRA sequence information supported intactness of *WA_A1* and *WB_B2*.

2-2-11 Chinese cavefish (*Sinocyclocheilus grahami (Sg), S. rhinocerous (Sr), S.anshuiensis (Sa)*)

Chinese cavefish from which W-category genes were identified include three closely related species, *Sinocyclocheilus grahami (Sg), S. rhinocerous (Sr)* and *S. anshuiensis (Sa)*. In our study, the same abbreviations of Sg, Sr and Sa are used as in a published report (65). In Chinese cavefish, we could identify two *WA/WB* gene pairs, namely, *WA_A1/WB_B1* and *WA_A2/WB_B2*, just like common carp; the assignments of 1 or 2 were in accordance with the names of the orthologous common carp genes (Fig. S20). Because both *WA_A1* and *WA_A2* were obtained only from *S. rhinocerous (Sr)*, W-category sequences of *S. rhinocerous (Sr)* were used as the representative sequences of Chinese cavefish in the analyses.

Chinese cavefish *WA* and *WB* sequences were found to correspond to the following predicted sequences through tblastn searches with zebrafish WA_13A and WB_13B sequences using the database of nucleotide collection; XM_016288790.1 (*WA_A1* of Sg), XM_016571073.1 (*WA_A1* of Sr), XM_016474588.1 (*WA_A1* of Sa), XM_016544921.1 (*WA_A2* of Sr), XM_016288788.1 (*WB_B1* of Sg), XM_016288789.1 (*WB_B1* of Sg, partial), XM_016571072.1 (*WB_B1* of Sr), XM_016474614.1 (*WB_B1* of Sa), XM_016263664.1 (*WB_B2* of Sg), XM_016544920.1 (*WB_B2* of Sr), and XM_016486958.1 (*WB_B2* of Sa). Chinese cavefish *WA* and *WB* genomic sequences were also detected in the following contigs through tblastn searches with zebrafish WA_13A and WB_13B sequences using wgs databases of bony fishes: LCYQ01031371.1 (including a complete pair of *WA_A1/WB_B1* of Sg, 102 K contig, 102623 bp; head to head configuration with respect to the transcriptional orientations; 341 bp between the two opposing nearest methionine-coding start codons, as there are two possible WB leader exons), LCYQ01012883.1 (including a partial *WA_A2* of Sg, 29 K contig, 29450 bp), LCYQ01125997.1 (including a partial *WA_A2* and a complete *WB_B2* of Sg, 2 K genomic sequence, 2712 bp), LAVF01191476.1 (including a complete pair of *WA_A2/WB_B2* of Sr, 50 K contig, 50091 bp; head to head configuration with respect to the transcriptional orientations; 329 bp between the two methionine-coding start codons), LAVF01079966.1 (including a partial *WA_A1* and a complete *WB_B1* of Sr, 64 K contig, 64734 bp), LAVF01079967.1 (including a partial *WA_A1* of Sr, 37 K contig, 37291 bp), LAVE01180287.1 (including a partial *WA_A1* of Sa, 36 K contig, 36160 bp), LAVE01000366.1 (including a partial *WA_A2* and a complete *WB_B2* of Sa, 31 K contig, 31461 bp) and LAVE01180285.1 (including a partial *WA_A1* and a complete *WB_B1* of Sa, 36 K contig, 36027 bp). Based on those shorter contigs, longer genomic scaffolds containing Chinese cavefish W-category genes are available as follows: NW_015505413 (*WA_A1/WB_B1* of Sg, 2.6 M, 2662682 bp), NW_015641068 (*WA_A1/WB_B1* of Sr, 124 K, 124120 bp), NW_015557379 (*WA_A1/WB_B1* of Sa, 3.3 M, 3351518 bp), NW_015666971 (*WA_A2/WB_B2* of Sr, 1.2 M, 1242612 bp), NW_015536465 (pseudo [?] *WA_A2/WB_B2* of Sa, 413 K, 413061 bp). Basically, the region containing W-category genes and the surrounding genes of Chinese cavefish is similar to the relevant region of zebrafish chromosome 13 (Fig. S11E).

2-2-12 Goldfish (*Carassius auratus*)

Goldfish *WA* and *WB* sequences were isolated based on the sequence information of zebrafish and/or common carp W-category genes. At first, to isolate goldfish W-category gene fragments, RT-PCR reaction was conducted using goldfish RNA and primers based on zebrafish and/or common carp W-category sequences. Then, 5' and 3' RACE reactions were conducted using goldfish *WA*- and *WB*-specific primers. After obtaining

goldfish partial sequences for *WA* and *WB*, complete coding regions of goldfish *WA* and *WB* genes were amplified (the primers are listed in Table S2) and the sequences were determined. Because the goldfish genome experienced the same additional whole genome duplication as the common carp genome did, a duplication of *WA/WB* pair in goldfish would be expected in comparison with the zebrafish genome (66). The isolated goldfish *WA* and *WB* sequences were compared with common carp *WA_A1*, *WA_A2*, *WB_B1* and *WB_B2* and, judging from sequence similarity levels, identified goldfish *WA* and *WB* sequences probably correspond to *WA_A1* and *WB_B1*, respectively (Fig. S20). The possible existence of additional *WA* and *WB* genes in goldfish remains to be studied.

2-2-13 Catla (*Gibelion catla* or *Catla catla*)
Catla *WA* and *WB* sequences were found in GEAE01049160.1 (for *WA*) and GEAE01019557.1 (for *WB*) through tblastn searches with zebrafish WA_13A and WB_13B sequences, respectively, using TSA databases of bony fishes.

2-2-14 Tench (*Tinca tinca*)
Tench *WA* and *WB* sequences were found in GFZX01071246.1 (for *WA*) and GFZX01039606.1 (for *WB*) through tblastn searches with zebrafish WA_13A and WB_13B sequences, respectively using TSA databases of bony fishes. The beginning of WB sequence is highly divergent compared to those of other closely related species and can be genomic, therefore, this part is not included in Fig. S2D.

2-2-15 Grass carp (*Ctenopharyngodon idella*)
Grass carp *WA* and *WB* sequences were found in GEUQ01023638 (for WA), GBKA01001259 (for WB) and GEUQ01052457 (for WB) through tblastn searches with zebrafish WA_13A and WB_13B using TSA databases of bony fishes. This grass carp *WA* cDNA sequence contains intron 2 sequence (between the α1 and α2 domain-coding exons) while all the other introns were properly spliced out. The SRA transcriptome databases contain properly spliced sequences of this grass carp *WA* gene (e. g., SRX1634319). Therefore, a complete transcript for grass carp *WA* can be assumed. Accessions GBKA01001259 and GEUQ01052457 contain the same WB-coding sequence.

2-2-16 Fathead minnow (*Pimephales promelas*)
Fathead minnow *WA* and *WB* sequences were found in JNCD01006382 (for *WA/WB* pair, “111 K contig” in Fig. S11E, 111553 bp; head to head configuration with respect to the transcriptional orientations; 173 bp between the two methionine-coding start codons; Fig. 5B) and JNCE01056294 (for *WA/WB* pair, “23 K contig”, 23124 bp) through tblastn searches with zebrafish WA_13A and WB_13B using wgs databases of bony fishes. The 111 K contig sequence includes the 23 K contig sequence fragment region.

2-2-17 Roach minnow (*Rutilus rutilus*)
Roach minnow *WA* and *WB* sequences were found in GEBE01046214.1 (encoding leader, α1 and partial α2 of WA), GEBE01046216.1 (encoding partial α2 and CP/TM/CY of WA), GEBE01052260.1 (encoding β1 and partial β2 domain of WB), and GEBE01052261.1 (encoding partial β2 and partial CP/TM of WB) through tblastn searches with zebrafish WA_13A (for the first two) and WB_13B (for the last two) sequences using TSA databases of bony fishes. The beginning of WB sequence is highly divergent compared to those of other closely related species and can be genomic, therefore, this part is not included in Fig. S2D.

2-2-18 Amur ide (*Leuciscus waleckii*)
Amur ide *WA* and *WB* sequences were found in FLSR01004870 (for *WA/WB* pair, “28 M contig” in Fig. S11E, LG15, 28600511 bp; head to head configuration with respect to the transcriptional orientations; 164 bp between the two methionine-coding start codons) through tblastn searches with zebrafish WA_13A and WB_13B using wgs databases of bony fishes. Compared with presumed intact genes, *WA* of this contig lacks a single nucleotide in the α1 domain exon, and *WB* of this contig lacks two nucleotides in the β1 domain exon. Presumably correct sequences were preliminarily constituted as information about short genomic sequences

of *WA* and *WB* genes are available from SRA of Amur ide in BioProject PRJEB12292, which also produced contig FLSR01004870 and, based on those sequences, the portions of available W-category sequences of Amur ide that were suggested defective appeared to be intact instead. The corrected *WA* of 28 M contigs for Amur ide *WA* was made after the confirmation with genomic SRA sequences of Amur ide (e.g., SRA:ERR1341435.99541468.2 of ERX1413017). Similarly the corrected *WB* of the same contig for Amur ide *WB* was made after the confirmation with genomic SRA sequences (e.g., SRA:ERR1341435.114433469.1 of ERX1413017). Basically, the region containing W-category genes and the surrounding genes of Amur ide is similar to the relevant region of zebrafish chromosome 13 (Fig. S11E).

2-2-19 Zebrafish (*Danio rerio*)

Zebrafish *WA/WB* genes (Fig. S11E) were the first pair of W-category genes that we recognized outside cartilaginous fish. During extensive searches for MHC genes in various teleost fish, we noticed two puzzling MHC genes on chromosome 13 of the zebrafish genome that had not been discussed in article format yet. These turned out to share characteristic features with the banded houndshark W-category genes. The sequence of zebrafish *WA* gene is NM_001098262 (for mRNA of zebrafish *WA_13A*; gene, zmp:0000001138; GeneID:100002901) and that of zebrafish *WB* gene is NM_001328089 (for mRNA of zebrafish *WB_13B*; gene, zmp:0000001006; GeneID:100002840). The zebrafish *WA/WB* gene pair displays head to head configuration with respect to the transcriptional orientations with 227 bp between the two methionine-coding start codons. In this very short region between two start codons, the promoter sequence motifs highly conserved between the classical MHC class I and class II genes (67) were not identified.

RT-PCR reactions were conducted using zebrafish RNA isolated from the anterior half of a zebrafish and primers based on the zebrafish genomic *WA* and *WB* sequences (Table S2) to obtain cDNA sequences of zebrafish W-category genes. Polymorphism of zebrafish W-category genes were investigated using different individuals (see *SI Appendix* section 5 and Fig. S19).

2-2-20 Brown ghost knifefish (*Apteronotus leptorhynchus*)

Brown ghost knifefish *WA* and *WB* sequences were found in GBKR010337083 (for *WA*) and GBKR010051454 (for *WB*) through tblastn searches with zebrafish WA_13A and WB_13B sequences, respectively, using TSA databases of bony fishes. There are twelve *WA*-containing sequences of brown ghost knifefish (GBKR010337075~ GBKR010337086) and GBKR010337083 described above contains the longest and complete WA-coding sequence. There are two *WB*-containing sequences and GBKR010051454 described above contains complete WB-coding sequence.

2-2-21 Black ghost (*Apteronotus albifrons*)

Black ghost *WA* and *WB* sequences were found in GFID01018756.1 (for *WA*) and GFID01018754.1 (for *WB*) through tblastn searches with zebrafish WA_13A and WB_13B sequences, respectively, using TSA databases of bony fishes.

2-2-22 Duckbill knifefish (*Parapteronotus hasemani*)

Duckbill knifefish *WA* and *WB* sequences were found in GGHK01230896.1 (for partial WA), GGHK01278630.1 (for partial WA) and GGHK01160239.1 (for partial WB) through tblastn searches with zebrafish WA_13A (for the first two) and WB_13B (for the last one) sequences using TSA databases of bony fishes. Two *WA* clones have an overlapping region with 11 nucleotides. In Fig. S1D, the combined sequence is shown.

2-2-23 Glass knifefish (*Eigenmannia virescens*)

Glass knifefish *WA* sequence was found in GGJF01002240 through tblastn searches with zebrafish WA_13A sequence using TSA databases of bony fishes. The beginning of this sequence appears to be in the genomic configuration with the presumed splicing signal of AG for *WA* $\alpha 1$ domain exon. Glass knifefish *WB* sequence has not been obtained yet.

2-2-24 Mexican tetra (*Astyanax mexicanus*)

Mexican tetra *WA* and *WB* sequences were found to correspond to XM_007254575 (Gene ID: 103042725) (*WA*) and XM_007254556 (Gene ID: 103034283) (*WB*) through tblastn searches with zebrafish WA_13A and WB_13B sequences using the database of nucleotide collection. These Mexican tetra *WA* and *WB* genes were identified in NW_006749376.1 ("2433 K contig", 2433507 bp; head to head configuration with respect to the transcriptional orientations; 176 bp between the two methionine-coding start codons) and this contig was later integrated into chromosome 22 (Fig. S11D). Based on these genomic sequences, we amplified *WA* and *WB* cDNAs (for primers, Table S2). After polishing the results, we obtained a single cDNA sequence of *WA* gene and also of *WB* gene, which matched the genomic sequences of *WA* and of *WB* genes, respectively.

2-2-25 Tambaqui (*Colossoma macropomum*)

Tambaqui *WA* and *WB* sequences were found in GGHL01010311 (for partial *WA*) and GGHL01049603 (for *WB*) through tblastn searches with zebrafish WA_13A and WB_13B sequences, respectively using TSA databases of bony fishes. The last part of the obtained WA sequence corresponding to ten amino acid residues is highly divergent compared to those of other closely related species and can be genomic, therefore, this part is not included in Fig. S1D.

2-3 Lobe-finned fish – coelacanth and lungfish

2-3-1 African coelacanth (*Latimeria chalumnae*)

At first, *WB* gene was identified in the genome of African coelacanth (34, 35), and then *WA* gene was identified as a gene paired with *WB* gene (Fig. S12A).

African coelacanth *WB* sequence was found to correspond to XM_014491414.1 (Gene ID: 102352955) through tblastn searches with banded houndshark WB_DS3 using the database of nucleotide collection of coelacanth. The sequence of XM_014491414.1 corresponds to a partial β1 domain plus complete β2 domain and CP/TM/CY region. Previously, this β2 domain exon was recognized as MHC class II β chain sequence in the *Mhc* region of African coelacanth (in JH127214.1 as reported in ref. 34). In the present study, complete β1 domain exon was identified in the genomic sequence of NW_005819663.1 (1088 K contig, 1088405 bp, same as JH127214.1 mentioned above). Further, the leader-encoding exon of *WB* was identified in BAHO01266725 (28764 bp).

Next to this *WB* gene in the genome, there exists another incompletely described MHC gene (XM_006000942.2, Gene ID: 102353475, annotated as beta-2-microglobulin-like). Our analyses revealed that this gene has some similarity with *WA* genes of other animals. The sequence of XM_006000942.2 corresponds to the leader, α2 domain and CP/TM/CY regions of an α chain gene of class II-type, *WA*. *WA* α1 domain exon was found between the leader and α2 domain exons through tblastn searches with zebrafish WA_13A using the relevant region within the sequence of NW_005819663.1. African coelacanth *WA* and *WB* genes are present in head to head configuration with respect to the transcriptional orientations and with 2418 bp between the two facing methionine start codons (Fig. S12A).

The expected spliced sequences of African coelacanth *WA* and *WB* genes were found in various SRA sequences, and some of them contain exon-junctions as follows; SRA:DRR002312.90231472.1 of DRX001723 (*WA*, containing the junction between the leader and α1 domain exons), SRA:DRR002317.11828674.2 of DRX001727 (*WA*, α2 domain and CP/TM/CY exons), SRA:DRR002312.69542290.1 of DRX001723 (*WB*, leader and β1 domain exons), SRA:DRR002312.69542290.2 of DRX001723 (*WB*, β1 and β2 domain exons), SRA: DRR002312.139051261.2 of DRX001723 (*WB*, β2 domain and CP/TM/CY exons), SRA:DRR002310.108065773.1 of DRX001721 (*WB*, the first and second junctions within CP/TM/CY exons). Only sequences containing the junction between *WA* α1 and α2 domain exons remain to be found.

2-3-2 Indonesian coelacanth (*Latimeria menadoensis*)

Indonesian coelacanth W-category gene sequences were found in SRA databases as described below.

Indonesian coelacanth *WA* partial sequences were found through tblastn searches with African coelacanth WA_1_01 sequence using SRX189185 (liver transcriptome) of Indonesian coelacanth SRA. Representative sequences are as follows: SRR576100.10484394.2, SRR576100.10484394.1, SRR576100.28293161.2. Indonesian coelacanth *WA* partial sequences were also found using SRX189186 (testis transcriptome). Representative sequences are as follows: SRR576101.33151112.1, SRR576101.9789453.1, and SRR576101.33928550.1.

Indonesian coelacanth *WB* partial sequences were found through tblastn searches with African coelacanth WB_1_01 sequence using SRX189185 (liver transcriptome) of Indonesian coelacanth SRA. Representative sequences are as follows: SRR576100.12524173.1, and SRR576100.12524173.2. Indonesian coelacanth *WB* partial sequences were also found using SRX189186 (testis transcriptome). Representative sequences are as follows: SRR576101.10092538.1, SRR576101.2943170.2, and SRR576101.12703814.2.

2-3-3 West African lungfish (*Protopterus annectens*)

For this animal, SRA datasets were utilized for the initial detection of W-category genes, and then the full-length coding sequence was amplified from cDNA by PCR experiments. Candidate *WA* and *WB* short cDNA sequences of West African lungfish could be obtained through tblastn searches with zebrafish WA_13A and WB_13B using SRA datasets of West African lungfish. We constructed presumed full-length cDNAs of *WA* and *WB* using SRA datasets, starting with short candidate cDNAs (e. g., SRA:SRR2028017.36937414.2 of SRX1016238 for *WA* and SRA:SRR2028017.1854076.1 of SRX1016238 for *WB*). *WA_2_01* and *WB_2_1_01* sequences were amplified from cDNA using presumed gene-specific primers (Table S2) and their complete coding sequences were determined.

West African lungfish *WA_1* partial sequences were found through tblastn searches with South American lungfish WA_1_01 sequence using SRX1016236 (liver RNA) of West African lungfish SRA. Representative sequences are as follows:
SRR2028021.6417444.1, SRR2028021.24594841.1, and SRR2028021.5460576.1.

West African lungfish *WB_1* partial sequences were found through tblastn searches with South American lungfish WB_1_01 sequence using SRX1016236 (liver RNA) of West African lungfish SRA. Representative sequences are as follows:
SRR2028021.33310257.1, SRR2028021.24798137.1, and SRR2028021.30422763.1.

2-3-4 Slender lungfish (*Protopterus dolloi*)

Slender lungfish W-category gene sequences were found in SRA databases as described below.

Slender lungfish *WA_1* partial sequences were found through tblastn searches with South American lungfish WA_1_01 sequence using SRX895335 (pelvic fin RNA) of slender lungfish SRA. Representative sequences are as follows: SRR1823814.10055282.2, SRR1823814.5075596.2, and SRR1823814.13757613.1.

Slender lungfish *WA_2* partial sequences were found through tblastn searches with West African lungfish WA_2_01 sequence using SRX895335 (pelvic fin RNA) of slender lungfish SRA. Representative sequences are as follows: SRR1823814.5579530.2, SRR1823814.9013780.1, and SRR1823814.18055363.1.

Slender lungfish *WB_1* partial sequences were found through tblastn searches with South American lungfish WB_1_01 sequence using SRX895335 (pelvic fin RNA) of slender lungfish SRA. Representative sequences are as follows: SRR1823814.11266724.1, SRR1823814.9260428.1, and SRR1823814.1607136.2.

Slender lungfish *WB_2* partial sequences were found through tblastn searches with West African lungfish WB_2_01 sequence using SRX895335 (pelvic fin RNA) of slender lungfish SRA. Representative sequences are as follows: SRR1823814.20167303.2, SRR1823814.2617696.2, and SRR1823814.20167303.1.

2-3-5 South American lungfish (*Lepidosiren paradoxa*)

South American lungfish *WA* and *WB* sequences were found in GEHZ01032426.1 (for WA_1_01) and GEHZ01040013.1 (for WB_1_01) through tblastn searches with African coelacanth WA_1_01 and WB_1_01 sequences, respectively, using TSA databases of lungfishes.

South American lungfish WA_2 and WB_2 were not found through tblastn searches with West African lungfish WA_2_01 and WB_2_01 sequences, respectively, using TSA databases of lungfishes.

2-3-6 Australian lungfish (*Neoceratodus forsteri*)
The sequence information of Australian lungfish *WA_1*, *WB_1*, *WA_2* and *WB_2* were obtained through the studies using SRA databases and *WA_1* and *WB_2_2* presumed sequences were constructed using SRA sequences as described below.

Australian lungfish *WA_1* partial sequences were found through tblastn searches with South American lungfish WA_1_01 sequence using SRX1823846 (pectoral fin RNA) of Australian lungfish SRA. Representative sequences are as follows:
SRR3632078.33699611.1, SRR3632078.19590582.1, and SRR3632078.19723085.1.
Australian lungfish *WA_1* partial sequences were also found through tblastn searches with South American lungfish WA_1_01 sequence using SRX4952748 (dorsal fin RNA) of Australian lungfish SRA. Representative sequences are as follows:
SRR8131642.15072763.1, SRR8131642.13478427.2, and SRR8131642.2822953.1.
A presumed complete Australian lungfish *WA_1* sequence was constructed using many short partial sequences like those described above and the presumed complete amino acid sequence is included in Fig. S1E.
Australian lungfish *WA_2* partial sequences were found through tblastn searches with West African lungfish WA_2_1_01 sequence using SRX1823846 (pectoral fin RNA) of Australian lungfish SRA. Representative sequences are as follows:
SRR3632078.17728174.2, SRR3632078.16709568.1, and SRR3632078.18029890.1.

Australian lungfish *WB_1* partial sequences were found through tblastn searches with South American lungfish WB_1_01 sequence using SRX1823846 (pectoral fin RNA) of Australian lungfish SRA. Representative sequences are as follows:
SRR3632078.34014254.1, SRR3632078.36687266.1, and SRR3632078.33111091.1.
Australian lungfish *WB_1* partial sequences were also found through tblastn searches with South American lungfish WB_1_01 sequence using SRX4952748 (dorsal fin RNA) of Australian lungfish SRA. Representative sequences are as follows:
SRR8131642.6755488.1, SRR8131642.3977539.2, and SRR8131642.15778735.2.
Australian lungfish *WB_2_1* partial sequences were found through tblastn searches with West African lungfish WB_2_1_01 sequence using SRX1823846 (pectoral fin RNA) of Australian lungfish SRA. Representative sequences are as follows:
SRR3632078.37409092.1, SRR3632078.15581747.2, and SRR3632078.24092841.2.
Australian lungfish *WB_2_2* partial sequences were found through tblastn searches with West African lungfish WB_2_1_01 sequence using SRX1823846 (pectoral fin RNA) of Australian lungfish SRA. Representative sequences are as follows:
SRR3632078.38307413.1, SRR3632078.35894717.1, and SRR3632078.35767306.1.
A presumed complete Australian lungfish *WB_2_2* sequence was constructed using many short partial sequences like those described above and the presumed complete amino acid sequence is included in Fig. S2E.

2-4 Tetrapods – salamanders
Extant amphibians belong to the subclass Lissamphibia which contains the order Anura (e.g., frogs), the order Urodela (e.g., salamanders) and the order Gymnophiona (e.g., caecilians). Thus far, W-category genes were identified in salamanders. Within the order Urodela, two suborders, Cryptobranchoidea and Salamandroidea, diverged from each other earlier than 157 MYA (68). Both Chinese salamander and Hokkaido salamander belong to the same genus *Hynobius* of the family Hynobiidae within the suborder Cryptobranchoidea (which also includes giant salamander). Tiger salamander belongs to Salamandroidea (which also includes axolotl and cynops).

2-4-1 Chinese salamander (*Hynobius chinensis*)

Chinese salamander *WA* sequences were found in GAQK01112360 (for the leader and partial α1 domain) and GAQK01026695 (for the partial α2 domain and CP/TM/CY region) through tblastn searches with tiger salamander WA_01 sequence using TSA databases of amphibians. GAQK01112360 and GAQK01026695 are derived from mRNA of whole body of Chinese salamander larvae. Candidate sequences of the missing last part of the α1 domain and the beginning of the α2 domain were obtained from SRA sequence reports (e. g., SRA:SRR1042328.36645099.2 of SRX386518 and SRA:SRR1042328.25098594.1 of SRX386518). For Fig. S1F, we combined two TSA sequences GAQK01112360 and GAQK01026695, and the sequence between these two came from the SRA sequences.

Chinese salamander *WB* sequences were found in GAQK01101790 (for the leader and the beginning of the β1 domain) and GAQK01119707 (for the partial β1 domain plus β2 domain and partial CP/TM/CY region) through tblastn searches with tiger salamander WB_01 sequence using TSA databases of amphibians. A region corresponding to four amino acid residues is overlapped by these two sequences. Sequences corresponding to the expected last five amino acids are missing in GAQK01119707. For Fig. S2F, we combined the two TSA sequences, GAQK01101790 for the leader plus the beginning of the β1 domain, and GAQK01119707 for the rest of the molecule except the last part.

2-4-2 Hokkaido salamander (*Hynobius retardatus*)

Hokkaido salamander *WA* and *WB* sequences were found in LE079167.1 (including a complete coding sequence of WA) and LE079819.1 (including a complete coding sequence of WB) through tblastn searches with tiger salamander WA_01 and WB_01 sequence, respectively, using TSA databases of amphibians. LE079167.1 and LE079819.1 are derived from mRNA of mixture of brain, gill, head and tail of Hokkaido salamander larvae.

2-4-3 Tiger salamander (*Ambystoma tigrinum*)

Identifications of tiger salamander *WA* and *WB* genes are described below.

A candidate sequence of partial tiger salamander *WA* gene was found in CN053477 through tblastn searches with zebrafish WA_13A using est database of amphibians. 5'- and 3'-RACE reactions were conducted with the primers based on this sequence and with RNA of tiger salamander. Using 5'- and 3'-UTR gene-specific primers, two complete coding sequences (*WA_01* and *WA_02*) were amplified from a single individual, which may be alleles (Fig. S1F).

A candidate sequence of salamander *WB* gene was found in Isotig_96288 through tblastn searches with zebrafish WB_13B sequence using the transcriptome of *Ambystoma mexicanum* (assembly V4.0-Isotigs + Singletons) at the Sal-Site (*Ambystoma* research resource development: R24OD010435). 5'- and 3'-RACE reactions were conducted with primers based on the central part of the genomic sequences of a presumable WB β2 domain exon of tiger salamander, which had been isolated with the information of Isotig_96288. Using 5'- and 3'-UTR gene-specific primers, two complete coding sequences (*WB_01* and *WB_02*) could be amplified from a single tiger salamander individual, which may be alleles (Fig. S2F).

2-4-4 Axolotl (*Ambystoma mexicanum*)

Axolotl *WA* and *WB* sequences were found in GFZP01140229 (including α2 domain and CP/TM/CY region of WA) and GFZP01045381 (including a complete coding sequence of WB) through tblastn searches with tiger salamander WA_01 and WB_01 sequence, respectively, using TSA databases of amphibians.

Axolotl *WA* and *WB* sequences were also found in PGSH01013846 ("5397 K contig", 5397334 bp DNA; head to head configuration with respect to the transcriptional orientations; 536 bp between the two methionine-coding start codons) through tblastn searches with tiger salamander WA_01 and WB_01 sequences using wgs databases of *Ambystoma mexicanum* (Fig. S12B). In this sequence, the leader, the α2 domain exon and the CP/TM/CY region of *WA*, and the leader, the β1 and β2 domain exons and the partial CP/TM/CY region of *WB* could be identified. In this *WA* gene sequence, no α1 domain exon, deletions of four nucleotides in the α2 domain exon, and a deletion of a single nucleotide in the second exon of CP/TM/CY region, were recognized. In *WB* gene sequence, the sequences of the last part corresponding to twenty amino acid residues, are missing. A complete α1 domain exon of *WA* was found in PGSH01122872 (57652 bp) by tblastn searches using wgs

databases of *Ambystoma mexicanum* with tiger salamander WA_01 sequence. The DNA sequences of the surrounding regions of *WA* α1 domain exon found in PGSH01122872 partially correspond to the sequences present in PGSH01013846.1 and some repetitive sequences were recognized in these contigs, which may have produced sequencing difficulty.

For WA sequence in Fig. S1F, the leader sequence of PGSH01013846, the α1 domain sequence from PGSH01122872 and the α2 domain and CP/TM/CY region from GFZP01140229 were combined. For WB sequence in Fig. S2F, WB_01 sequence from GFZP01045381 was used.

Based on the latest information, the two genomic sequences mentioned above (PGSH01013846 and PGSH01122872) were replaced by PGSH00000000.2. However, W-category sequences could not be found in the newer axolotl wgs databases (JXRH01 and PGSH02), possibly because they may be still included in the unplaced regions and therefore not included in the chromosomal regions. As axolotl *WA* sequences were found in GFBM010788916.1 (length 1045, *WA*α1 and first half of α2, second half of α2 and CP/TM/CY portion) and GFBM010763839.1 (length 3323, *WA* leader portion) through tblastn searches using TSA databases of *Ambystoma mexicanum*, we present Fig. S12B as a useful reference.

3. Southern blot analyses of the banded houndshark *W-category* genes

Because many banded houndshark W-category gene sequences were determined using the banded houndshark individual N1, the Southern blot analyses using DNA of the banded houndshark individual N1 is shown in Fig. S3. In the linkage experiments of Fig. S9, the results of Southern blot analyses were shown with the probes of banded houndshark *WA_DS5*, *WB_DS1* and *WB_DS3* using banded houndshark individuals different from N1 and also using different restriction enzymes. The following numbers of loci (in parentheses) are consistent with the results of Southern blot analyses presented in Figs. S3 and S9: banded houndshark *WA_DS5* (3), *WA_DS10* (1), *WB_DS1* (2), *WB_DS3* (1) and β_2-*m* (1).

In Fig. S10, the uncropped versions of the Southern blots used in Fig. S9 are shown to indicate that there are no additional positive bands in the blots.

4. Banded houndshark *W-category* genes are expressed in various tissues

The expression of the banded houndshark *W-category* genes was studied using RT-PCR.

Essentially, both *WA* (*WA_DS5* and *WA_DS10*) and *WB* genes (*WB_DS1_n1* and *WB_DS3*) are expressed in spleen, liver and kidney as shown in Fig. S4. The expression of *WA_DS5* was also detected in blood.

As described in the section 2, cDNA of W-category genes could be isolated from various animals; banded houndshark (kidney, spleen and liver), goldfish (gill), zebrafish (an anterior half of the body), Mexican tetra (gill), West African lungfish (kidney) and tiger salamander (internal organs). Further we could detect W-category cDNA sequences in various transcriptomic databases. Therefore, currently identified W-category genes may be expressed in multiple tissues although W-category subgroup-specific expression patterns might not be excluded and could be observed after further analyses.

5. Variation of W-category molecules

In the following sections, general observations about variation of W-category molecules are described. When appropriate data are available, the extent of presumable allelic polymorphism is discussed, since the classical MHC molecules are known to possess extremely high allelic polymorphism in their peptide-binding domains (i.e. membrane-distal domains) and it should be important to know whether W-category molecules also possess such property. Although based on limited information, the available results suggest that the membrane-distal domains of W-category molecules do not possess high allelic polymorphism.

5-1 Variation among W-category molecules in sharks

Banded houndshark possesses two kinds of WA, namely WA_DS5 and WA_DS10, and two kinds of WB, namely WB_DS1 and WB_DS3. In some other cartilaginous fishes, in addition to these, WA_Nds3L and WB_Nds5L are also identified.

Selected amino acid identity % are as follows:
Between banded houndshark WA_DS5_n1 and banded houndshark WA_DS10, 16 % for α1 domain and 31 % for α2 domain.
Between banded houndshark WA_DS5_n4 and banded houndshark WA_DS10, 17 % for α1 and 33 % for α2.
Between banded houndshark WA_DS5_n1 and great white shark WA_Nds3L, 10 % for α1 and 36 % for α2.
Between banded houndshark WA_DS10 and great white shark WA_Nds3L, 12 % for α1 and 33 % for α2.

Between banded houndshark WA_DS5_n1 and great white shark WA_DS5-like, 84 % for α1 and 79 % for α2.
Between banded houndshark WA_DS5_n4 and great white shark WA_DS5-like, 52 % for α1 and 78 % for α2.
Between banded houndshark WA_DS10 and great white shark WA_DS10-like, 56 % for α1 and 64 % for α2.
Between blue shark WA_Nds3L and great white shark WA_Nds3L, 47 % for α1 and 56 % for α2.

Between banded houndshark WB_DS1_n1 and banded houndshark WB_DS3, 13 % for β1 and 40 % for β2.
Between banded houndshark WB_DS1 and great white shark WB_Nds5L, 14 % for β1 and 31 % for β2.
Between banded houndshark WB_DS3 and great white shark WB_Nds5L, 15 % for β1 and 26 % for β2.

Between banded houndshark WB_DS1_n1 and great white shark WB_DS1-like, 48 % for β1 and 78 % for β2.
Between banded houndshark WA_DS3 and great white shark WA_DS3-like, 64 % for β1 and 66 % for β2.
Between blue shark WB_Nds5L and great white shark WB_Nds5L, 63 % for β1 and 73 % for β2.

Variation observed in banded houndshark WA_DS5
As shown in Fig. S1A, four kinds of *WA_DS5* cDNA could be identified from the banded houndshark individual N1, namely *WA_DS5_n1_01*, *n2_01*, *n3_01* and *n4_01*. WA_DS5_n1_01, n2_01 and n3_01 possess α1 domains highly similar to each other while WA_DS5_n4_01 possesses a highly distinct one (amino acid identity % = 47 ~ 48 % between n1_01/n2_01/n3_01 and n4_01). Regarding the leader, α2 domain and CP/TM/CY region, all four possess highly similar sequences (identity = 95 ~ 99 %). Based on our preliminary genomic analyses, *WA_DS5_n4_01* may be situated at a locus (loci) distinct from those of *WA_DS5_n1_01*, *n2_01* and *n3_01*. Therefore, currently it is assumed that the variation observed between WA_DS5_n4_01 and WA_DS5_n1_01/n2_01/n3_01 is not allelic polymorphism but variation observed between distinct loci, although further studies should be necessary to clarify relationships among various WA_DS5 members.

Variation observed in banded houndshark WA_DS10
As described in section 2-1-1-2, two kinds of cDNA of *WA_DS10* were identified from the banded houndshark individual N1. The difference between the two sequences is ascribed to a single nucleotide (C or T) of the third nucleotide position of a codon in the α2 domain exon, which does not alter the encoded amino acid. This difference was confirmed with multiple clones of independent PCR reactions and also with direct sequencing of the PCR products as described in the Materials and Methods. From the simple pattern of Southern blot results (Figs. S3 and S9), and also from the specific amplification of these cDNAs using 5'- and 3'-UTR primers, it follows that these cDNAs presumably represent allelic sequences.

Variation observed in banded houndshark WB_DS1
As shown in Fig. S2A, two kinds of cDNA of banded houndshark *WB_DS1_n1* were identified from the banded houndshark individual N1. These presumably constitute allelic sequences of the *WB_DS1_n1* locus. The two sequences show disparity at only two amino acid positions in the membrane-distal β1 domain. The

expression of another *WB_DS1* gene, *WB_DS1_n2*, seems very much limited, as it was only found in an unusual transcript described in the section (2-1-1-2) of banded houndshark *WA_DS10*.

Variation observed in banded houndshark WB_DS3

As shown in Fig. S2B, thus far two kinds of sequences of banded houndshark *WB_DS3* were obtained from different individuals, *WB_DS3_01* from banded houndshark individual N2 and *WB_DS3_02* from individual N1. The two sequences show disparity at only two amino acid positions in the Ig-like domain and the CY region and no difference is observed in their membrane-distal β1 domains.

5-2 Variation among W-category molecules in teleost fish

5-2-1 Variation and similarity among W-category molecules in teleost fish

Thus far, in ray-finned fish, W-category genes can be identified in the teleost fish group. Generally, W-category molecules of the teleost fish group share pronounced features as shown in the phylogenetic tree (Fig. 7) and amino acid sequence comparisons (Figs. S1 and S2). Within the teleost fish group, the order Clupeiformes (e.g., Atlantic herring and allis shad) is estimated to have diverged from the superorder Ostariophysi (e.g., zebrafish and Mexican tetra) around 261 MYA (240-282 MYA; ref. 61). Teleost fish WA and WB sequences can be relatively well aligned within each group and amino acid identity percentages between the W-category molecules of Atlantic herring and Mexican tetra are obtained as follows: WA α1, 59 %; WA α2 60 %; WB β1 63 %; WB β2 57 %. As an example of comparison between closely related species, amino acid percentages between the W-category molecules of zebrafish and fathead minnow, both belonging to the family Cyprinidae, are as follows: WA α1, 79 %; WA α2 80 %; WB β1 77 %; WB β2 73 %.

Below, variations of W-category molecules in zebrafish and Chinese cavefish (both belonging to the family Cyprinidae) are described .

5-2-2 Inspection of allelic polymorphism of W-category molecules in zebrafish

To investigate possible presence of high polymorphism in the membrane-distal domains of the W-category molecules of zebrafish, variation of those domains was studied using four different strains of zebrafish with unknown relationships. Using RT-PCR method, the expressed W-category sequences were amplified from an individual zebrafish of four different strains (AB, India [IND], TL and WIK) (zebrafish WA_01~05 and zebrafish WB_01~05; Fig. S19, A and B). Analyzing five or more clones of amplified fragment per gene per zebrafish individual, two sequences were obtained from a single individual at most (Fig. S19C). Because only a single pair of *WA/WB* genes can be detected in the genome of zebrafish (Fig. S11E), each obtained sequence can be assumed to be derived from a single locus, *WA* or *WB*. Very limited variation both in the membrane-distal domain of WA (Fig. S19A) and in the membrane-distal domain of WB (Fig. S19B) was observed. It is concluded that both zebrafish WA and WB molecules exhibit very limited allelic polymorphism in the membrane-distal domains (WA α1 and WB β1).

5-2-3 Variation of W-category molecules among closely related species of Chinese cavefish

In the Chinese cavefish, *Sinocyclocheilus*, several W-category sequences can be detected. Chinese cavefish WA sequences can be classified into two, A1 and A2, based on similarity with the common carp WA_A1 and WA_A2 sequences, respectively (Fig. S20A). Similarly, Chinese cavefish WB sequences can be classified into two, B1 and B2, based on similarity with the common carp WB_B1 and WB_B2 sequences, respectively (Fig. S20B). In the common carp contig, *WA_A1* and *WB_B1* exist as a pair and are situated very close to each other. In another common carp contig, *WA_A2* and *WB_B2* similarly exist as a pair. Inspecting the Chinese cavefish contigs, we confirmed that Chinese cavefish *WA_A1* and *WB_B1* constitute a pair, and *WA_A2* and *WB_B2* constitute another pair. With these Chinese cavefish sequences, sequences of different but closely related three species of Chinese cavefish could be compared at the same locus. Even between different species of *Sinocyclocheilus*, only a limited amount of variation could be observed in the membrane-distal domains of WA and WB sequences as shown (Fig. S20).

5-3 Variation among W-category molecules in lobe-finned fish

In lungfish, currently two major subgroups of WA molecules were identified: WA_1 and WA_2.
Amino acid identity % are as follows:
Between South American lungfish WA_1 and Australian lungfish WA_1, 40 % for α1 domain and 70 % for α2 domain.
Between South American lungfish WA_1 and West African lungfish WA_2, 19 % for α1 and 35 % for α2.
Between Australian lungfish WA_1 and West African lungfish WA_2, 19 % for α1 and 33 % for α2.

As African coelacanth WA molecule shares some specific amino acid residues with South American lungfish and Australian lungfish WA_1 molecules (Fig. S1E), African coelacanth WA molecule is temporarily classified as WA_1 subgroup.
Amino acid identity % are as follows:
Between South American lungfish WA_1 and African coelacanth WA_1, 22 % for α1 domain and 39 % for α2 domain.
Between Australian lungfish WA_1 and African coelacanth WA_1, 33 % for α1 and 43 % for α2.
Between African coelacanth WA_1 and West African lungfish WA_2, 13 % for α1 and 32 % for α2.

In lungfish, currently two major subgroups of WB molecules were identified: WB_1 and WB_2. Temporarily, WB_2 can be further classified into two groups: WB_2_1 and WB_2_2.
Amino acid identity % are as follows:
Between South American lungfish WB_1 and West African lungfish WB_2_1, 18 % for β1 domain and 32 % for β2 domain.
Between South American lungfish WB_1 and Australian lungfish WB_2_2, 18 % for β1 and 29 % for β2.
Between West African lungfish WB_2_1 and Australian lungfish WB_2_2, 37 % for β1 and 58 % for β2.

As African coelacanth WB molecule shares some specific amino acid residues with South American lungfish WB_1 molecule (Fig. S2E), African coelacanth WB molecule is temporarily classified as WB_1 subgroup.
Amino acid identity % are as follows:
Between South American lungfish WB_1 and African coelacanth WB_1, 22 % for β1 domain and 47 % for β2 domain.
Between West African lungfish WB_2_1 and African coelacanth WB_1, 15 % for β1 and 32 % for β2.
Between Australian lungfish WB_2_2 and African coelacanth WB_1, 18 % for β1 and 32 % for β2.

As already described in the previous section 2-3 and summarized in Table S1, WA_1 sequence could be identified in West African lungfish, and WA_2 sequence could be identified in Australian lungfish based on the investigation using SRA databases. Similarly, WB_1 sequence could be identified in West African lungfish and also in Australian lungfish, WB_2_1 sequence could be identified in Australian lungfish, and WB_2_2 sequence could be identified in West African lungfish.

5-4 Variation among W-category molecules in salamanders

Between the Chinese salamander and the Hokkaido salamander, which both belong to the same genus *Hynobius*, only a small number of amino acid differences could be observed in the membrane-distal domains (96 % amino acid identity for WA and 97 % for WB).

Between the two suborders, Salamandroidea and Cryptobranchoidea, W-category molecules exhibit a relatively divergent nature in their membrane-distal domains while they exhibit more conserved nature in the Ig-like domains as shown in Figs. S1 and S2. For example, the amino acid identity percentages between tiger salamander WA_01 (Salamandroidea) and Hokkaido salamander (Cryptobranchoidea) WA_01 are as follows: 52 % in WA α1 domain and 76 % in WA α2 domain. Those between tiger salamander WB_01 and Hokkaido salamander (Cryptobranchoidea) WB_01 are as follows: 35 % in WB β1 domain and 64 % in WB β2 domain.

From a single individual tiger salamander, presumed alleles of two WA and also presumed alleles of two WB sequences could be isolated as shown in Figs. S1 and S2. In the membrane-distal domains, they show a small number of amino acid differences (95 % amino acid identity for WA and 98 % for WB).

6. Conserved features at the Iα3/β_2-m interface

Iα3 P57 and β_2-m Y8 are highly conserved in class I molecules at the Iα3/β_2-m interface (e. g., human, 1DLH of PDB ID; mouse, 2VAA; chicken, 3BEV; and grass carp, 5Y91) and these residues are highly conserved at the corresponding positions of W-category molecules (in the WB β2 domain and WA α2 domain). In Fig. S6A, the positions of Iα3 P57 and β_2-m Y8 in HLA-A2, which interact through a hydrogen bond, are shown at the Iα3/β_2-m interface. In Fig. S6B, the corresponding interface in the MHC class II molecule, namely, the IIBβ2/IIAα2 interface is shown. Although some class II molecules possess Y8 in IIAα2, there is no conserved P57 in IIBβ2 (Fig. 3; Dataset S2).

7. β_2-m K67 can form an intra-domain hydrogen bond

β_2-m K67 is one of the amino acid residues shared between the WA α2 domain and β_2-m, which are shaded in red color in Fig. 3 and Dataset S2. Because phylogenetically relatively primitive jawed vertebrates such as grass carp possess a lysine (K) at this position in their β_2-m molecules, the structural features of β_2-m K67 can be studied for grass carp β_2-m (69) (PDB: 3GBL and 5Y91; Fig. S7). As reported previously (69), a hydrogen atom from the ε-amino group of grass carp β_2-m K67 forms hydrogen bonds with a main chain carbonyl oxygen of β_2-m I35 [Fig. S7A, length of hydrogen bond = 2.75 Å] and also with a γ-carboxyl oxygen of β_2-m E36 [Fig. S7B, length of hydrogen bond = 3.54 Å]. At position 35 of the WA α2 domain and also of β_2-m, an isoleucine (I) is often replaced by other amino acid residues. Because a main chain carbonyl oxygen is involved in the hydrogen bonds between β_2-m I35 and β_2-m K67, the conservation of this hydrogen bond is assumed in the WA α2 domain as well as in β_2-m of other phylogenetically relatively primitive vertebrates. In addition, as seen in Fig. S7, grass carp β_2-m L37, which also corresponds with residues shaded in red color in the β_2-m and WA α2 domain sequences (Fig. 3 and Dataset S2), is positioned close to β_2-m K67 in the three-dimensional structure (69). It is notable that the classical class I α3 domain of all cartilaginous fish (Dataset S2) possess both I α3L37 and I α3K/R68, and independent acquisitions of L37 plus K/R68 might have occurred in β_2-m and in the classical class I α3 domain of cartilaginous fish.

8. Linkage between the banded houndshark *β_2-m* gene and the *Mhc* region

The banded houndshark *β_2-m* gene was isolated using PCR based on information of cartilaginous *β_2-m* genes, and has been deposited in GenBank (HQ630063 for the *Triakis scyllium* β_2-m mRNA and HQ634972 for the *Triakis scyllium β_2-m* gene). The primer sequences for the amplification of the transcript of *β_2-m* are listed in Table S2.

Linkage analysis was conducted using a previously described panel of seventeen littermate sharks (17). The PCR products (~3.8 kb) of banded houndshark *β_2-m* gene, amplified with two primers (F11 and R10, Table S2), were digested with the restriction enzyme, *Mbo*II, to detect distribution of allelic variation among the littermates and their mother.

Figure S15A shows an additional band in six samples, indicated with an arrow. Classification with or without the additional band is in complete concordance with the haplotype classification obtained for the classical MHC genes (Fig. S9; 17). This pattern also matches those reported for complement *C4* and *Bf* genes of banded houndshark which were concluded to be linked with the classical MHC genes (70). Using sample No. 12, the region of interest was sequenced (Fig. S15B) and seven nucleotides were revealed as responsible for the additional band.

In conclusion, the results indicate that the banded houndshark *β_2-m* gene is linked with the *Mhc* region, as reported previously for nurse shark (28).

9. Specific interaction between WA and WB

As W-category *WA* and *WB* genes are present close to each other as a pair in the genome of sharks, teleost fish, coelacanth and salamander like MHC class II genes (Figs. S11 and S12; *SI Appendix*), the protein products of these genes are expected to form a heterodimer (2, 3). Both classical MHC class I and class II molecules form a heterodimer in the endoplasmic reticulum, and after they bind appropriate peptide ligands at their respective intracellular locations, they are transported and expressed on the cell surface. Although it is not clear whether W-category molecules bind any ligands, which might be necessary for stabilization and efficient transport, possible intracellular processing and cell surface expression of recombinant W-category proteins of tiger salamander was investigated using Western blotting and flow cytometry. Total protein lysates of the cells used for the flow cytometry experiments were also analyzed by Western blotting. The results shown in Fig. 6 and Figs. S13 and S14 are representatives of several independent experiments with similar results.

Processing of recombinant α and β chain proteins of a tiger salamander W-category molecule

Figure 6 confirmed the production of recombinant WA (Fig. 6A) and WB (Fig. 6B) proteins by transfected cells. Further, apparent higher molecular weight species were observed only when both WA and WB of tiger salamander were simultaneously introduced into cells [Fig. S6A (d) for WA; Fig. S6B (d) for WB], compared to the controls [Fig. S6A (b) and (f) for WA; Fig. S6B (c) for WB]. The apparent size differences can be ascribed to different N-glycosylations of proteins as shown by the digestion of the samples with Endo H [Fig. 6C (b) and (d)] or GPF [Fig. 6D (b) and (d)]. In case of the transfectants with both WA and WB of tiger salamander, the WA protein fractions of relatively lower molecular weight could be digested with Endo H while the protein fractions of higher molecular weight could not be [Fig. 6C (d)], suggesting that those undigested proteins possess an Endo H-resistant complex glycan structure. This was confirmed by the experiments which showed that those proteins of higher molecular weight actually could be digested with GPF [Fig. 6D (d)]. In contrast, in the controls in which tiger salamander WA plus empty PA vector [Fig. 6C (b)] or tiger salamander WA plus Mexican tetra WB [Fig. 6C (f)] were present, all WA proteins could be digested with Endo H. Regarding the additional band of higher molecular weight of WB protein detected in Fig. 6B (d), this particular band became obscure (possibly due to protein aggregation) with a digestion protocol without addition of enzymes (mock treatment) (Fig. S14). Therefore, in case of WB proteins, a conclusion could not be drawn about its detailed glycosylation state.

Thus, when both WA and WB of a tiger salamander were introduced into cells, the molecular species of higher molecular weight were observed for both WA and WB. In case of WA, these molecular species were apparently produced by the advanced glycosylation processing.

For the possible N-glycosylation sites, the following multiple asparagines could be mentioned solely based on the sequence information of tiger salamander WA_01 and WB_01:
WAα1, N26, N119 (numbers are based on Dataset S1); WAα2, N60, N97 (based on Dataset S2); WA CP region, N14 (based on Dataset S3); WBβ2, N34, N60, N97 (based on Dataset S2).

Cell surface expression of recombinant α and β chain proteins of a tiger salamander W-category molecule

Figure 6E and F shows that the recombinant WA (α chain) and WB (β chain) of a tiger salamander W-category molecule could be observed on the cell surface only when these two chains were simultaneously introduced into cells. Namely, for the detection of α chains, low but reproducible binding of anti-FLAG antibody to FLAG-tagged WA on the cell surface could be observed only in the presence of both tiger salamander WA and WB [Fig. 6E (d)] in contrast to the controls in which tiger salamander WA plus empty PA vector [Fig. 6E (b)] or tiger salamander WA plus Mexican tetra WB [Fig. 6E (f)] were introduced. For the detection of β chains, significant binding of anti-PA antibody to PA-tagged WB on the cell surface could be observed only in the presence of both tiger salamander WA and WB [Fig. 6F (d)] in contrast to the controls in which empty FLAG vector plus tiger salamander WB [Fig. 6F (c)] or tiger salamander WA plus Mexican tetra WB [Fig. 6F (f)] were introduced.

Taken together, both the recombinant WA and WB proteins of a tiger salamander W-category molecule were processed differently from controls and could be detected on the cell surface, only when these two tiger

salamander chains were simultaneously introduced into cells. These results indicate a specific interaction between recombinant WA and WB chains of a tiger salamander W-category molecule.

10. Conservation profile of the teleost fish W-category membrane-distal domains and molecular modeling of the extracellular domains of W-category molecule

10-1 The teleost fish W-category membrane-distal domains show a unique conservation profile

To gain insight into the nature of W-category molecules, the conservation profile was investigated in the membrane-distal domains of teleost fish W-category molecules. The membrane-distal domains of the classical MHC class I and class II molecules are known to be highly polymorphic and critically responsible for peptide ligand-binding. The present investigation became possible because a large number of sequences of the teleost fish W-category genes could be identified from various fish species.

More than twenty sequences of WA and WB were compared (Figs. S1 and S2) and the Wu-Kabat variability (described in the Materials and Methods) was calculated (Table S4). Originally Wu-Kabat variability was used to study the variable nature of immunoglobulins, but later also used to study the polymorphic nature of the membrane-distal domains of MHC molecules (e. g., 17, 71, 72). The conservation profile, i. e., the patterns of positions of highly conserved and variable residues were found to be remarkably similar between the teleost fish W-category molecules and MHC-Z molecules (Fig. S21). MHC-Z molecules are ancient nonclassical class I molecules which possess most of the peptide-termini-binding amino acid residues of the classical MHC class I molecules; compared to most classical MHC class I molecules in non-mammalian jawed vertebrates, they possess seven out of eight such residues, although their possible (peptide) ligand(s) has not been clarified yet (36-38). In contrast, these eight amino acid residues are not conserved in the teleost fish W-category molecules (Fig. S21). Previously, the positions of the conserved residues among MHC-Z molecules have been revealed to correspond to the peptide-binding positions of the classical MHC class I molecules (38). In the present study, the positions of conserved residues among the teleost fish W-category molecules (Table S4) were found to correspond to those of peptide-binding residues of the classical MHC class I molecules and also of the classical MHC class II molecules (Fig. S21). Based on Fig. S21 and the structure of HLA-A2, the hypothetical membrane-distal domains of the teleost fish W-category molecule are shown in Fig. S22. More than 80 % of the peptide binding positions of the HLA-A2 molecule correspond to highly conserved positions of the teleost fish W-category molecules (Figs. S21 and S22). In the α-helical regions, many positions of the HLA-A2 residues that point away from the binding groove (25) correspond to highly variable residues in the teleost fish W-category molecules (Fig. S22 and Table S4).

The present study suggests that the teleost fish W-category subgroup molecules may bind some conserved ligands in their presumed groove, although in the teleost fish W-category molecules, neither the eight amino acid residues of the classical MHC class I molecules which interact with the peptide termini of a bound peptide nor the four amino acid residues of the classical MHC class II molecules which interact with the main chain residues of a bound peptide are conserved (Fig. S21).

10-2 Molecular modeling of the extracellular domains of W-category molecule

For the prediction of the protein structure of the W-category molecules, we used protein modeling tools such as Phyre2 (90) and SWISS-MODEL (91), although there exist various limitations for such analyses. We briefly show here some selected results obtained with Phyre2 and SWISS-MODEL, and compared to our predicted alignment and structure (Figs. S21 and S22) of the membrane-distal domains of the teleost fish W-category molecule which was obtained based on the conservation profile.

For both protein modeling softwares, we used five query sequences.

1. allis shad WA α1 (a single domain)
2. allis shad WA α2 (a single domain)
3. allis shad WA all (a whole sequence including signal peptide and CP/TM/CY regions)
4. allis shad WB all (a whole sequence including signal peptide and CP/TM/CY regions)

5. allis shad WA α1 + WB β1β2 (artificially combined three-domains sequence)

Further, for SWISS-MODEL, we used the following sequences for hetero oligomeric protein analyses.
6. allis shad WA all / WB all (two sequences, four extracellular domains, class II-type model)
7. allis shad (WA α1 + WB β1β2) / WA α2 (two sequences, four extracellular domains, class I-type model)

In the last part of this section, we present the following results obtained with the modeling programs in this order.

Representative results of the modeling analyses using Phyre2 or SWISS-MODEL
Representative results of the 3D modeling in the Phyre2 analyses
Representative results of the alignments in the Phyre2 analyses
Representative results of the 3D modeling in the SWISS-MODEL analyses
Representative results of the alignments in the SWISS-MODEL analyses

10-2-1 Overall comparison

Phyre2 and SWISS-MODEL produced multiple predicted structural models of the teleost fish W-category molecule. The models of the teleost fish W-category molecule are basically similar to those of the classical MHC class I and also to those of the classical MHC class II molecules. In the two membrane-proximal Ig-like domains, namely, WA α2 and WB β2 domains, especially high similarity and highly probable alignments were obtained in various model cases. In the membrane-distal domains, highly probable alignment in WB β1 domain were obtained whereas especially the second half of the WA α1 domain (namely, α-helix region) showed variations.

With the [allis shad WB all] sequence or with the [allis shad WAα1WBβ1β2] sequence as a query sequence, the α2 and α3 domains of various class I sequences were obtained as templates for the corresponding WBβ1β2 sequence. The alignment for the WB β1 sequence including the α-helix region is highly similar between those obtained with the models (both Phyre2 and SWISS-MODEL) and that of Fig. S21. Therefore, the proposed alignment shown in Fig. S21 was basically similar to the models except for the α-helix region of WA α1 domain. Further, even for WA α1 domain, some models showed the alignments similar to that of Fig. S21 in the α-helix region to some extent. One example was obtained in the following model, in which the consecutive 28 amino acid positions of the last part of the WA α1 domain are consistent with the alignment of Fig. S21: allis shad WA all / WB all, SWISS-MODEL No. 1 template 3pdo.1 Human MHC class I HLA-DR1 (class II-type model).

If the two presumed α-helix structures are reasonably apart from each other and there exists a "groove", some conserved ligand may be expected. As described above, W-category molecule possesses neither the eight amino acids highly conserved in the classical MHC class I for the binding to the bound peptide termini nor the four amino acids highly conserved in the classical MHC class II for the binding to the main chain of a bound peptide. However, W-category could bind a peptide with the principle different from the classical MHC class I and also different from the classical MHC class II. Clarification of possible ligand(s) including peptides remains to be investigated.

10-2-2 Molecular modeling of the inter-domain interfaces of W-category molecule

In the model of [allis shad WA all/WB all] and [allis shad (WAα1 +WBβ1β2/WAα2)], amino acid residues like WAα2W61 and Y57 can be found in the interface between WAα1/WBβ1 and WAα2 domains (in the pictures shown below). This has been expected because α1 and α2 domains of class II molecule superimpose closely on the corresponding α1 domain and the β_2-m, respectively, of class I, and β1 and β 2 domains of class II superimpose on the α2 and less closely on the α3, respectively, of class I, as described by Brown et al. (27), and in our analyses, [allis shad WA all/WB all] sequence has class II-type domain architecture and [allis shad (WAα1 + WBβ1β2/WAα2)] sequence has class I-type domain architecture. However, the existence

of those amino acid residues like W61 in the relevant interface, should be checked with the model, which can consider structural distortions, unfavorable interactions and clashes.

In the class I molecules, β_2-m W61 interacts with α2Q6 and α2D37 through hydrogen bonds. Although in the class II-type model of [allis shad WA all/WB all], the oxygen of WBβ1D37 is not close to the nitrogen of WAα2W61, they are very closely located to each other in the class I-type model of [allis shad (WAα1 +WBβ1β2/WAα2)]. In both models, however, the nitrogen of WB β1Q6 is very close to the oxygen of main chain of WAα2W61. For the modeling of W-category molecule, class I-type modeling appear to produce a better model with respect to the domain-interfaces.

In the α3/ β_2-m interface of a class I molecule, β_2-m Y8 interacts with α3P57 through hydrogen-bonding. In the corresponding WBβ2/ WAα2 interface of the model, WBβ2P57 can also be found in the positions very close to WAα2Y8 in case of class I-type model and not class II-type model.

Apparently, currently there is no highly appropriate template for the modeling which fulfills requirements for both domain architecture and domain-interfaces.

Future structural determination of W-category molecules should reveal the details of the inter-domain interfaces and the structure of the membrane-distal domains, and further possible ligand(s).

Representative results of the modeling analyses using Phyre2 or SWISS-MODEL

Some explanations in the softwares:

"Rank" in Phyre2 means that "models are ranked according to raw alignment score".

"Confidence" in Phyre2 is "the probability that the query sequence and the template are homologous".

"GMQE" in SWISS-MODEL means "Global Model Quality Estimate".

Numbers in red, shown in the columns of "Rank" or "No.", indicate the modeling results with the alignments and 3D models shown later.

1. allis shad WA α1

Phyre2 allis shad WA α1

Rank	Confidence	Template information	Template	Sequence Identity %
1	37.7	Rum1-related protein	c4idiA	40
2	23.0	Alpha-amylases	d1j0ha2	32
3	19.7	Alpha-amylases	d1m53a1	33
4	17.8	Alpha-amylases	d1uoka1	38
5	14.6	Mouse idothyronine deidodinase 3	c4tr3A	25
6	12.8	Human nonclassical class I ZAG α1	c1t7wA	15
7	11.1	Alanine racemase	d1d7ka1	34
8	9.9	Alpha amylases	d1wzla2	32
9	9.6	Alpha amylases	d1jl1a2	26
10	8.6	Marsupial MHC class I α1	c7edoA	17

SWISS-MODEL allis shad WA α1

No.	GMQE	Template information	Template	Sequence Identity %
1	0.19	Mouse MHC class II IA α1	6blr.1.A	10.00
2	0.19	Mouse MHC class II A-D α1	6dfs.1.C	10.00
3	0.17	Human MHC class II HLA-DQ8 α1	1jk8.1.A	5.88
4	0.09	Beta-glucosidase 1	4iib.1.A	12.2
5	0.09	Beta-glucosidase	5ju6.1.A	4.88
6	0.09	Beta-glucosidase	5fjj.1.A	12.50
7	0.06	Alpha-amylase	1ob0.1.A	20.83
8	0.06	Alpha-1, 4-glucan-4-glucanohydrolase	1bpl.1.B	21.74
9	0.05	Amylase	6toy.1.A	20.00

2. allis shad WA α2

Phyre2　allis shad WA α2

Rank	Confidence	Template information	Template	Sequence Identity %
1	99.9	Human MHC nonclassical class II HLA-DO	c3usaG	29
2	99.9	Human MHC class II HLA-DR	c4aenA	31
3	99.9	Chicken MHC class II	c6kvmA	28
4	99.9	Human MHC class II HLA-DR	c2q6wD	31
5	99.9	Human MHC class II HLA-DP	c3lqzA	29
6	99.9	Antibody constant domain-like	d1klub1	35
7	99.9	Antibody constant domain-like	d1klua1	31
8	99.9	Antibody constant domain-like	d1es0a1	24
9	99.9	Antibody constant domain-like	d1fnga1	32
10	99.9	Antibody constant domain-like	d1iaka1	24
21	99.9	Human nonclassical class I MR1 β_2-m	c4gupB	42

SWISS-MODEL　allis shad WA α2

No.	GMQE	Template information	Template	Sequence Identity %
1	0.79	Mouse MHC class I H-2Kb β_2-m	2qri.2.A	39.13
2	0.79	HLA-A with H-2K α3 domain β_2-m	6e1i.1.A	41.30
3	0.79	Mouse MHC class I H-2Kb β_2-m	5oqg.2.A	39.13
4	0.79	Mouse MHC class I H-2Db β_2-m	6mp1.1.A	39.13
5	0.79	Rat MHC class I β_2-m	1kjv.1.B	39.13
6	0.79	Mouse MHC class I H-2Kb β_2-m	2qrt.1.A	39.13
7	0.78	Mouse MHC class I H-2Kb β_2-m	2qri.1.A	39.13
8	0.78	Chicken MHC class I BF β_2-m	6lhf.1.B	39.78
9	0.78	Chicken MHC class I BF β_2-m	6lhg.1.B	39.78
10	0.78	Mouse MHC class I H-2Kb β_2-m	5oqg.1.A	39.13

3. allis shad WA all

Phyre2 allis shad WA all

Rank	Confidence	Template information	Template	Sequence Identity %
1	100.0	Chicken MHC class II α1α2	c6kvmA	19
2	100.0	Human MHC class II HLA-DM α1α2	c2bc4C	16
3	100.0	Human MHC class II HLA-DO α1α2	c3usaG	20
4	100.0	Mouse MHC class II H-2 I-A α1α2	c1f3jD	18
5	100.0	Human MHC class II HLA-DR α1α2	c1sebB	22
6	100.0	Mouse MHC class I H-2K α2α3	c3tidA	20
7	100.0	Chicken MHC class I Rfp-Y α2α3	c3p73A	22
8	100.0	Mouse MHC class I H-2K α2α3	c2clvA	20
9	100.0	Human MHC class II HLA-DQ α2α3	c1s9vA	20
10	100.0	Mouse MHC class I H-2K α2α3	c1kj2I	20

SWISS-MODEL allis shad WA all

No.	GMQE	Template information	Template	Sequence Identity %
1	0.47	Human MHC class II HLA-DR1	1aqd.1.B	21.39
2	0.47	Human MHC class II HLA-DR1	1aqd.4.B	21.39
3	0.47	Human MHC class II HLA-DQB1	5ksa.1.B	20.21
4	0.46	Human MHC class II HLA-DRB1	3pgd.1.B	21.16
5	0.45	Human MHC class II HLA-DRB1	3pdo.1.B	21.16
6	0.44	Mouse MHC class I H-2 D-B	6h6d.1.A	19.91
7	0.44	Mouse MHC class I H-2 D-B	1s7u.1.A	19.91
8	0.44	Mouse MHC class I H-2 D-B	1s7v.1.A	19.91
9	0.43	Mouse MHC class I H-2 D-B	1s7u.3.A	19.91
10	0.43	Mouse MHC class I H-2 D-B	1s7u.2.A	19.91

4. allis shad WB all

Phyre2 allis shad WB all

Rank	Confidence	Template information	Template	Sequence Identity %
1	100.0	Chicken MHC class I B21 α2α3	c2yf6A	19
2	100.0	Chicken MHC class I Rfp-Y α2α3	c3p73A	19
3	100.0	Mouse MHC class I H-2 K α2α3	c1kj3H	19
4	100.0	Mouse MHC class I H-2 K α2α3	c2clvA	19
5	100.0	Mouse MHC class I H-2 K α2α3	c3tidA	19
6	100.0	Mouse MHC class I H-2 K α2α3	c1nanH	19
7	100.0	Mouse MHC class I H-2 K α2α3	c1kj2I	19
8	100.0	Mouse MHC class I H-2 K α2α3	c2zswA	19
9	100.0	Mouse MHC class I H-2 K α2α3	c2zswC	19
10	100.0	Mouse MHC class I H-2 K α2α3	c2zswG	19

SWISS-MODEL allis shad WB all

No.	GMQE	Template information	Template	Sequence Identity %
1	0.43	Chicken MHC class I Rfp-Y α2α3	3p77.1.A	20.32
2	0.41	Human nonclassical class I MR1 α2α3	6w9u.2.A	24.04
3	0.41	Chicken MHC class I B2 α2α3	4cvx.1.A	20.32
4	0.41	Human nonclassical class I ZAG α2α3	1zag.1.A	21.51
5	0.40	Chicken MHC class I B2α2α3	4d0d.1.A	20.32
6	0.40	Human nonclassical class I FcRn α2α3	6fgb.1.A	19.82
7	0.40	Human nonclassical class I ZAG α2α3	1zag.3.A	21.51
8	0.40	Human nonclassical class I ZAG α2α3	1t7v.1.A	20.97
9	0.39	Human nonclassical class I ZAG α2α3	3es6.1.A	20.97
10	0.39	Human MHC class I HLA-B7 α2α3	6at5.1.A	18.60

5. allis shad (WA α1 + WB β1β2)

Phyre2 allis shad (WA α1 + WB β1β2)

Rank	Confidence	Template information	Template	Sequence Identity %
1	100.0	Mouse nonclassical class I CD1 α1α2α3	c1cd1C	17
2	100.0	Chicken MHC class I B21 α1α2α3	c2yf6A	18
3	100.0	Chicken MHC class I B21 α1α2α3	c3bewD	16
4	100.0	Chicken MHC class I Rfp-Y α1α2α3	c3p73A	17
5	100.0	Mouse nonclassical class I CD1d1 α1α2α3	c2akrA	16
6	100.0	Human nonclassical class I CD1d	c1zt4C	17
7	100.0	Human nonclassical class I CD1a α1α2α3	c1xz0A	15
8	100.0	Nonclassical class I HFE α1α2α3	c1de4A	16
9	100.0	Xenopus laevis MHC class I α1α2α3	c6a2bA	17
10	100.0	Chicken nonclassical class I CD1	c3dbxA	16

SWISS-MODEL allis shad (WA α1 + WB β1β2)

No.	GMQE	Template information	Template	Sequence Identity %
1	0.58	Anolis MHC class I α1α2α3	7cpo.1.A	18.33
2	0.57	Chicken MHC class I B2 α1α2α3	4cvx.1.A	19.69
3	0.57	Cattle MHC class I α1α2α3	3pwv.1.A	17/65
4	0.57	Chicken MHC class I B2 α1α2α3	4d0d.1.A	19.69
5	0.57	Bat MHC class I α1α2α3	6ilf.1.A	17.05
6	0.57	Bat MHC class I α1α2α3	6ilc.1.A	17.05
7	0.56	Cattle MHC class I α1α2α3	3pwu.1.A	17.65
8	0.56	Human MHC class I HLA-B57 α1α2α3	5vvp.1.A	17.65
9	0.56	Chicken MHC class I Rfp-Y α1α2α3	3p77.1.A	18.95
10	0.56	Xenopus MHC class I α1α2α3	6a2b.1.A	18.04

6. allis shad WA all / WB all

SWISS-MODEL allis shad WA all / WB all

No.	GMQE	Template information	Template	Sequence Identity %
1	0.46	Human MHC class II HLA-DR1	3pdo.1	22.25
2	0.46	Human MHC class II HLA-DR1	4x5w.1	22.25
3	0.46	Human MHC class II HLA-DQ	4may.1	22.93
4	0.46	Mouse MHC class II H2-I-EK	1fne.1	23.08
5	0.46	Human MHC class II HLA-DQA	6dig.1	22.99
6	0.46	Human MHC class II HLA-DQ1	3pl6.1	22.93
7	0.46	Human MHC class II HLA-DQ	1uvq.1	23.06
8	0.46	Mouse MHC class II H2-E	1kt2.1	23.64
9	0.46	Mouse MHC class II H2-I-Au	2p24.1	20.72
10	0.46	Human MHC class II HLA-DR	2wbj.1	22.31

7. allis shad (WA α1 + WB β1β2) / WA α2

SWISS-MODEL allis shad (WA α1 + WB β1β2) / WA α2

No.	GMQE	Template information	Template	Sequence Identity %
1	0.66	Chicken MHC class I BF2	6lhf.1	25.15
2	0.66	Chicken MHC class I BF2	6lhg.1	23.98
3	0.65	Duck MHC class I	6kyu.1	23.17
4	0.65	Duck MHC class I	5gjjx.1	24.12
5	0.65	Chicken MHC class I B2	4cvx.1	25.07
6	0.65	Chicken MHC class I B21	4cvz.1	25.22
7	0.65	Anolis MHC class I	7cpo.1	23.62
8	0.64	Bat MHC class I	6ilc.1	24.36
9	0.64	Anolis MHC class I	7cpo.1	23.82
10	0.64	Human MHC class I HLA-B57	3wuw.1	23.92

Representative results of the 3D modeling in the Phyre2 analyses

1. allis shad WA α1

Phyre2 Rank 10 template c7edoA Marsupial class1 α1

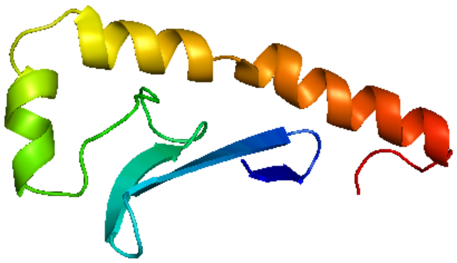

2. allis shad WA α2

Phyre2 Rank 2 template c4aenA Human MHC class II HLA-DR α2

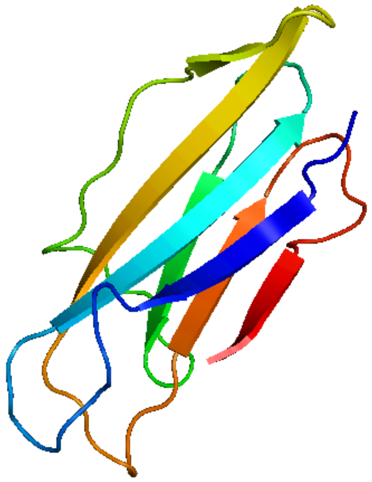

3. allis shad WA all

Phyre2 Rank 1 template c6kvmA   Chicken MHC class II α1α2

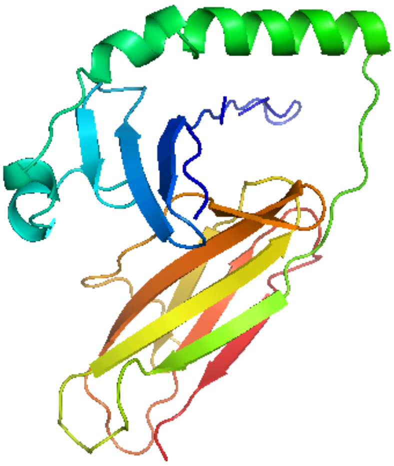

4. allis shad WB all

Phyre2 Rank 1 template c2yf6A   Chicken MHC class1 α2α3

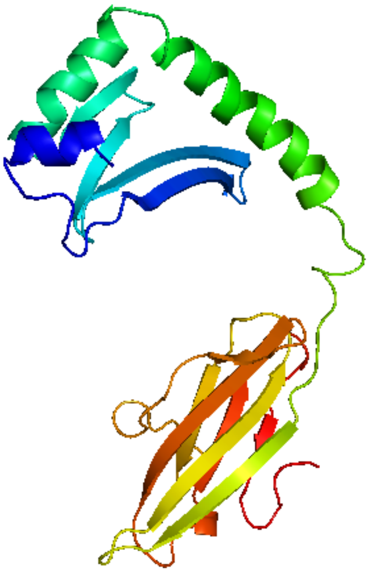

5. allis shad (WA α1 + WB β1β2)

Phyre2 Rank 2 template c2yf6A   Chicken MHC class1 α1α2α3

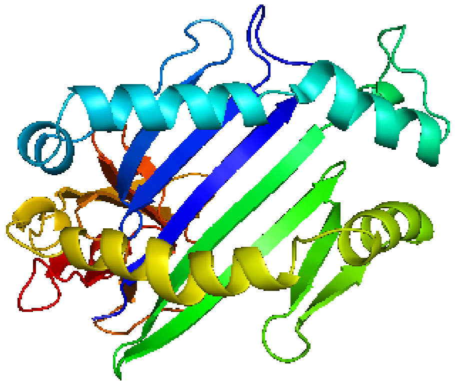

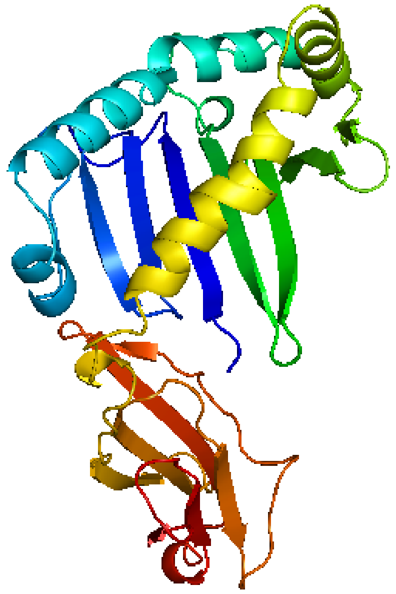

Representative results of the alignments in the Phyre2 analyses

1. allis shad WA α1
Phyre2 Rank 10 template c7edoA Marsupial class I α1

```
10 c7edoA_ Probab=8.56    E-value=3.8e+02  Score=13.42  Aligned_columns=82  Identities=17%

Q ss_pred              EEEEECCCC-C-CEEEEEEEEHHHHHHHHHCCCEEEECHHHHHHHHHH-HHHHHHHHHH
Q ss_conf              654204786-2-104887871167764430054575540212235877-42114141798
Q allis_shad_WA_   10 YTRLTSRDS-V-EQGVLVLVNEAVFAYFNATEKTFLLRPTAMAGFSVL-EAKERMYCVSE   66 (92)
Q Consensus        10 ytr~~~~~s-i-~q~v~vlvn~aifa~f~~~~~tf~l~pta~agfsvl-e~~e~~~c~~e   66 (92)
                       ||-+.+.++ .  +-.+++.|+|-.|.+||...+++-..|.+.---.++ |..+-.=...+
T Consensus         9 ~T~~s~~~~g~p~f~~v~~vDd~~~~~Yds~~~~~r~~p~~~W~~~~~~~~~~~~we~~t~   68 (278)
T c7edoA_          9 YTAVSGPELREPRFLSVGYVDEQQFVRFDSASESPREEPRAKWIERVGEEDPEYWERQTG   68 (278)
T ss_dssp              EEEEECSSCSSCEEEEEEEETTEEEEEEETTSSSCCCEECSGGGGGGGGGSTTHHHHHHH
T ss_pred              EEEEECCCCCCCEEEEEEEECCEEEEEEEECCCCCCEEEECHHHHHHHCCCCHHHHHHHHH
T ss_conf              9998167898845999999999899998278754234204045543103682789999999


Q ss_pred              HHHCCCCH-HHHHHHHHHHHCCCCCC
Q ss_conf              74105012-79999999631257889
Q allis_shad_WA_   67 VINAFPRQ-QDYLDKLIKQTNGAKPP   91 (92)
Q Consensus        67 v~~~f~rq-~~yl~kl~k~t~~~kpp   91 (92)
                       ..+...+.  ++.+..++.+.|.++.+
T Consensus        69 ~~~~~~~~~~~~~~~l~~i~~~~n~s~~g   94 (278)
T c7edoA_         69 ILRRNTQVFRVGLETLRGYFNQSAGG   94 (278)
T ss_dssp              HHHHHHHHHHHHHHHHHHHHHTTCCTTC
T ss_pred              HHHHHHHHHHHHHHHHHHHHHHCCCCCC
T ss_conf              99889999999999999860478776
```

2. allis shad WA α2
Phyre2 Rank 2 template c4aenA Human MHC class II HLA-DR α2

```
2 c4aenA_ Probab=99.93   E-value=5.1e-25  Score=163.41  Aligned_columns=94  Identities=31%

Q ss_pred              CCCEEEEEECCCCEECCCCEEEEEEEEECCCCCCCEEEEEEECCEECCCCCEEEEEEEECCCCE
Q ss_conf              96589980567433698489999997677985316886277372777635578864897 0
Q allis_shad_WA_    1 LSPSVNVYSHFPAMPGSPNYLYCYATGFYPGDIEISFVLNGRPFPGPTESSDLVYGEDWT   60 (94)
Q Consensus         1 V~P~v~v~~~~~~~~~~g~~~tL~C~a~g~~P~~~~v~W~~~g~~l~~~~~~~~~~~~~~~~~~~   60 (94)
                       |+|+|+|+++.++..|+.++|+|.|.||||+.++|+|++||+++..++..+..++++||+
T Consensus        83 ~pP~v~v~~~~~~~~~~~~~~~~L~C~a~gFyP~~i~v~W~~ng~~~~~~~~~~~~~~~~~~DgT  142 (178)
T c4aenA_         83 VPPEVTVLTNSPVELREPNVLICFIDKFTPPVVNVTWLRNGKPVTTGVSETVFLPREDHL  142 (178)
T ss_dssp              BCCEEEEEESSCCCTTSCEEEEEEEEEEBSSCCEEEEEEETTEECCSSCEECCCEECTTSC
T ss_pred              CCCCEEEEECCCCCCCCCEEEEEEEEECCCCCCEEEEEEEECCEECCCCCEECCEEECCCCE
T ss_conf              78612897158565677358999991300783599998768104477400201643896 0


Q ss_pred              EEEEEEEEEECCCCCEEEEEEEECCCCCCCEEEEC
Q ss_conf              67999999952327889999990878886797 39
Q allis_shad_WA_   61 FRVFKYISILPLPGEVYECFVNHSSLAQPKITVW   94 (94)
Q Consensus        61 ~~~~s~l~v~~~d~~~ytC~V~H~~l~~p~~~~~~   94 (94)
                       |++.+.|.+.+++++.|+|+|+|++|.+|++++|
T Consensus       143 f~~~s~L~v~p~~~~~~ytC~V~H~sL~~p~~~~~w  176 (178)
T c4aenA_        143 FRKFHYLPFLPSTEDVYDCRVEHWGLDEPLLKHW  176 (178)
T ss_dssp              EEEEEEEEECCCSSCCEEEEEEECTTSSSCEEEEE
T ss_pred              EEEEEEEEEECCCCCEEEEEEEEECCCCCCEEEEE
T ss_conf              99999999489988799999907788976787 3
```

3. allis shad WA all

Phyre2 Rank 1 template c6kvmA Chicken MHC class II α1α2

```
1 c6kvmA_ Probab=100.00  E-value=0  Score=308.98  Aligned_columns=180  Identities=19%

Q ss_pred              EEEEEE-EEEEECCCCCCCEEEEEEEECCEEEEEEEECCCCEEEEEECCCCCCCEEECCCCCC
Q ss_conf              044466-676651699823079999837059999847975997125665410000012 31
Q allis_shad_WA_   26 EAVSIL-AYTRLTSRDSVEQGVLVLVNEAVFAYFNATEKTFLLRPTAMAGFSVLEAKERM   84 (251)
Q Consensus       26 h~~~~~-~~c~~~~~gs~~~~~~~~~~~d~~~f~~fd~~~~~~~~~~~p~a~~~~~~~~~~~~~~~~~   84 (251)
                       |++++.  ++|+..|++...|.++++|||+||++||++.++|+       +..+........
T Consensus        4 h~~~~~~f~~~~~gp~~~~G~~~ygyDGee~~~~D~~~~~~v------~~~p~~~~~~~~~   57 (184)
T c6kvmA_          4 HVLLQAEFYQRSEGPDKAWAQFGFHFDADELFHVELDAAQTV------WRLPEFGRFASF   57 (184)
T ss_dssp             CCEEEEEEEEEEETTTTEEEEEEEEEEETTEEEEEECTTSCCEE------ESSGGGGGTCCC
T ss_pred             CEEEEEEEEEEECCCCCCCCEEEEEECCCEEEEEEECCCCEEE------EECCCCCCHHHH
T ss_conf             258889987750688777621568888670699998257558 7------307532212233


Q ss_pred              HHHHHHHHHHHHHHHHHHHHHHHHHHCCCCCCCCCCEEEEEECCCCCCCCCEEEEEEEEECEE
Q ss_conf              0233210112335789999997401233443796489999538877887049999993303
Q allis_shad_WA_   85 YCVSEVINAFPRQQDYLDKLIKQTNGAKPPKLSPSVNVYSHFPAMPGSPNYLYCYATGFY  144 (251)
Q Consensus       85 ~~~~~~~~~~~~~~~~~~~~~~~~~~~~L~~~~~~~~~~~~~~~~~~~~~~v~P~V~V~~~~p~~~g~~~~L~C~v~gFY  144 (251)
                       +|+..... ..+|.+||++++++++.+++++++|+|.|+++++++.|++++|+|+|+|||
T Consensus       58 ~~~~~~~~-l~~c~~~L~~~l~~~~~~~~~~~~~~~pP~V~v~~~~~~~~~~~~~~~~~tL~C~a~gFy  116 (184)
T c6kvmA_         58 EAQGALQN-MAVGKQNLEVMISNSNRSQQDFVTPELALFPAEAVSLEEPNVLICYADKFW  116 (184)
T ss_dssp             CGGGHHHH-HHHHHHHHHHHHHHHTTSCCCCCBCCEEEEEEESSCCCTTCCEEEEEEEEEEB
T ss_pred             HHCCCHHH-HHHHHHHHHHHHHHHCCCCCCCCCCEEEEEEECCCCCCCCCEEEEEEEEECCC
T ss_conf             31001688-998799999999972453211257516999815877667743899999 2401


Q ss_pred              CCCEEEEEEEECCEECCCCCEECCEEECCCCEEEEEEEEEEEEECCCCCEEEEEEEEECCCCCC
Q ss_conf              674399999999898126620020153299719999999998089988899999936 67886
Q allis_shad_WA_  145 PGDIEISFVLNGRPFPGPTESSDLVYGEDWTFRVFKYISILPLPGEVYECFVNHSSLAQP  204 (251)
Q Consensus      145 P~~I~vtW~knG~~~~~~~~v~~s~~~pn~DgTyq~~s~L~v~p~~~~~~YtC~V~H~sL~~p  204 (251)
                       |++|+|+|+|||++++++++.++++||+|||||++++|+++|++++.|+|+|+|+||.+|
T Consensus      117 P~~I~v~W~~ng~~~~~~~~~~~~~~~~~pn~Dgtf~~~s~L~~~p~~~~~~ytC~V~H~sL~~p  176 (184)
T c6kvmA_        117 PPVATMEWRRNGAVVSEGVYDSVYYGRPDLLFRKFSYLPFVPQRGDVYSCAVRHWGAEGP  176 (184)
T ss_dssp             SSCCEEEEEEETTEEECTTCEECCCEECGGGCEEEEEEEEEECCCTTCCEEEEEEECTTSSSC
T ss_pred             CCCEEEEEEEECCEECCCCCCCCCCEEECCCCEEEEEEEEEEEEECCCCCEEEEEEEEECCCCCC
T ss_conf             682699999788322366311421653996099999999981886887999999 0678887


Q ss_pred              EEEEEECC
Q ss_conf              68997236
Q allis_shad_WA_  205 KITVWRPE  212 (251)
Q Consensus      205 ~~~~W~p~  212 (251)
                       +++.|+|+
T Consensus      177 ~~~~W~pe  184 (184)
T c6kvmA_        177 VQRMWEPE  184 (184)
T ss_dssp             EEEEECCC
T ss_pred             EEEEEECC
T ss_conf             58997229
```

4. allis shad WB all

Phyre2 Rank 1 template c2yf6A   Chicken MHC class I α2α3

```
1 c2yf6A_ Probab=100.00  E-value=0  Score=345.82  Aligned_columns=199  Identities=19%

Q ss_pred             HHHHHHCCCCCCCCCEEEEEEEEEEEECCCCCCEEEEEEEECCEEEEEEEECCCCEEEEECCC
Q ss_conf             268664034789997589999888983599801279998858057999768778997880
Q allis_shad_WB_    6 CAILLGLSSLNARDEFTYQQYIGCAFNRQGPVGRFWRYGFNTKDIMQVDLKNEAVVAVSD   65 (273)
Q Consensus        6 ~~~l~~~~~~~~~~~~ht~Q~~~GCel~~~g~~~g~~~~gyDGed~i~fD~d~~~wva~~~   65 (273)
                     ...++...... ++.||+|+++|||+.+||...+|++|||||+||++||.++.+|++..+
T Consensus       77 l~~~~~~~n~t-~G~htlQ~~~GCel~~dg~~~~~~~~~aydG~d~l~fD~~~~~w~~~~~  135 (276)
T c2yf6A_        77 LDILRRRYNQT-GGSHTVQWMSGCDILEDGTIRGYHQAAYDGRDFVAFDKGTMTLTAAVP  135 (276)
T ss_dssp             HHHHHHHTTCC-SSSCEEEEEEEEEEECSSCCEEEEEEEEEETTEEEEEEEETTTTEEEECSG
T ss_pred             HHHHHHHCCCC-CCCEEEEEEEEEEEEECCCCEEEEEEEEEECCCEEEEEEECCCCEEEECCH
T ss_conf             99999742577-676179998878861897043689999759069999268518995482


Q ss_pred             CCCHHHHHHHHHHHHHHHHHHHHCCCCHHHHHHHHHCCCC-CCCCCCCCCEEEEECCCCCCE
Q ss_conf             01403344311346789887411555566554211332-21234455302442167774 3
Q allis_shad_WB_   66 EGNFMAEERQSKVYFKDKEYKLMRICSAVNTVFLQSNN-SLSKDAKPTVRVSLEESEGKE  124 (273)
Q Consensus       66 ~a~~~~~~~~~~~~~~~~~~~~~~~~k~yle~~C~~~L~~~l~~~~-~~~~~~~~p~v~~~~~~~~~~~~~~  124 (273)
                     .|...++.++...++++.+.|++..|+.||++|+++++ .+.+++||.|.++++.+++.+
T Consensus      136 ~a~~~k~~w~~~~~~~~~~~k~yl~~~C~~~L~~yL~~~k~~l~~~~~~P~v~v~~~~~~~~~~~  195 (276)
T c2yf6A_       136 EAVPTKRKWEEGGYAEGLKQYLEETCVEWLRRYVEYGKAELGRRERPEVRVWGKEADGIL  195 (276)
T ss_dssp             GGHHHHHHHHHSSSHHHHHHHHTTHHHHHHHHHHHHTHHHHHCCBCCEEEEEEEEESSSCE
T ss_pred             HHHHHHHHHCCHHHHHHHHHHHHHHHHHHHHHHHHHHHHHHCCCCCCCCCCCEEEEECCCCCCE
T ss_conf             688888872766789999987343438998887620223344566625999814898825


Q ss_pred             EEEEEEECCCCCCEEEEEEECCCCCCCCCCCCCCEEECCCCEEEEEEEEEEEECCCCCCEEEE
Q ss_conf             899999042271129999418847157731143174289729999999982665886999
Q allis_shad_WB_  125 YIMCSVQGFSPNTISVRWVYKGKIVHFARTTTGLLPRKDGTFQITSYLTLGNKTLQDIVC  184 (273)
Q Consensus      125 ~l~C~~~gFyP~~i~v~W~~~g~~~~~~~~~~~~~~~~~~~~~pn~dgtf~~~~~~~l~v~~~~~~~~~~~ytC  184 (273)
                     +|+|+|+||||++|+|+|++||+++..++..++++||+|||||++++|++++++++.|+|
T Consensus      196 ~L~C~~~gFyP~~i~v~W~~~g~~~~~~~~~~~~~~~~~~~~~pn~dgtf~~~~~~~l~v~~~~~~~~~~~ytC  255 (276)
T c2yf6A_       196 TLSCRAHGFYPRPIVVSWLKDGAVRGQDAQSGGIVPNGDGTYHTWVTIDAQPGDGDKYQC  255 (276)
T ss_dssp             EEEEEEEEEBSSCCEEEEEEEESSCBCTTTCEECCCEECTTSCEEEEEEEEEEECTTCSTTEEE
T ss_pred             EEEEEEECEECCCCEEEEEEECCEECCCCEEECCCEECCCCCEEEEEEEEEEEECCCCCEEEE
T ss_conf             999999320478519999998899567513043076499758999999992021886999


Q ss_pred             EEEECCCCCCEEEEECCCCCCC
Q ss_conf             999326898489981587665
Q allis_shad_WB_  185 ETEHISIEGTLQATLDDKYSM  205 (273)
Q Consensus      185 ~V~H~~l~~p~~~~~~~~~~~~~~~~~~  205 (273)
                     +|+|+||++|+++.|+|.+..
T Consensus      256 ~V~H~sl~~p~~~~~w~~~~~~~~  276 (276)
T c2yf6A_       256 RVEHASLPQPGLYSWRSGGGL  276 (276)
T ss_dssp             EEECTTCSSCEEECTTTTSCC
T ss_pred             EEEECCCCCCEEEEEEECCCCC
T ss_conf             999178987768995027889
```

5. allis shad (WA α1 + WB β1β2)
Phyre2 Rank 2 template c2yf6A   Chicken MHC class I α1α2α3

```
2 c2yf6A_ Probab=100.00  E-value=0  Score=481.63  Aligned_columns=266  Identities=18%

Q ss_pred             CCCEEEEEEEEECCCCCCC-CEEEEEEECCEEEEEEECCCCEEEECCCCCCCCCHHHHH
Q ss_conf             950488999740268877-44799999998999995899778972632244332036 78
Q allis_shad_WA_    1 DYEAVSILAYTRLTSRDSV-EQGVLVLVNEAVFAYFNATEKTFLLRPTAMAGFSVLEAKE   59 (273)
Q Consensus         1 ~~~~~~~~~~~~~~~~~~~~~-~~~vv~yvDd~~~~~YDS~~~~~~pr~~W~~e~~~~~~we   59 (273)
                      .+|+|..+......+.++. +|.+++||||++|++|||++++++|+++||++++++++||
T Consensus         1 e~HSL~y~~T~~s~~~~~~~p~f~~~g~lDd~~~~~Yds~~~~~~p~~~W~~~~~~~~e~we   60 (276)
T c2yf6A_         1 ELHTLRYIRTAMTDPGPGLPWFVDVGYVDGELFMHYNSTARRAVPRTEWIAANTDQQYWD   60 (276)
T ss_dssp            CCEEEEEEEEEESCCCTTCCSEEEEEEETTEEEEEECTTTCCCEECSHHHHTTSCHHHHH
T ss_pred             CCEEEEEEEEEECCCCCCCCEEEEEEEECCEEEEEEECCCCEEEECCHHHCCCCCHHHHH
T ss_conf             963599999996388898725999999999899999789986998433530128816789


Q ss_pred             HHHHHHHHHHH-HHHHHHHHHHHHHHHHCCCCCCCCEEEEEEEEEEEEECCCCEEEEEEEE
Q ss_conf             77777777754-46779999999999851135788855899999899727985 4589997
Q allis_shad_WA_   60 RMYCVSEVINA-FPRQQDYLDKLIKQTNGAKPPKDEFTYQQYIGCAFNRQGPVGRFWRYG  118 (273)
Q Consensus        60 ~~t~~~~~~~~~~~-~~~~~~~~~~~~~~~~~~~~~t~~~~~~~~~~g~HtlQ~~~GCel~~dg~~~~~~~~~~~~~  118 (273)
                      ++++.....+. |+.++..++..+|++      +|+||+|+++|||+.+||.++||++||
T Consensus        61 ~~t~~~~~~~~~~~~~~~~~~l~~~~~~~~~n~t------~G~htlQ~~~GCel~~dg~~~~~~~~~a  114 (276)
T c2yf6A_        61 RETQIVQGSEQINRENLDILRRRYNQT------GGSHTVQWMSGCDILEDGTIRGYHQAA  114 (276)
T ss_dssp            HHHHHHHHHHHHHHHHHHHHHHHTTCC------SSSCEEEEEEEEEEECSSCCEEEEEEEE
T ss_pred             HHHHHHHHHHHHHHHHHHHHHHHCCCC------CCCEEEEEEEEEEEEECCCCEEEEEEEE
T ss_conf             999887548999999999999742577------6761799988788618970436899 99


Q ss_pred             ECCCEEEEEEECCCCEEEECCHHHHHHHHHHCCHHHHHHHHHHHHHHHHHHHHHHHHHCCCC-
Q ss_conf             757227898624434874254554566641085788999998633578888987 40354-
Q allis_shad_WA_  119 FNTKDIMQVDLKNEAVVAVSDEGNFMAEERQSKVYFKDKEYKLMRICSAVNTVFLQSNN-  177 (273)
Q Consensus       119 yDGed~l~fD~~~~~~~w~a~~~~~~a~~~k~~w~~~~~~~~~~~~~k~yl~~~Ci~~L~kyL~~~~~-  177 (273)
                      |||+|||+||.++++|+++.|+|+.+|++|++...+++.+.||+..|++||++||++++
T Consensus       115 ydG~d~l~fD~~~~~~~w~~~~~~~~a~~~k~~w~~~~~~~~~~~~~k~yl~~~C~~~L~~yL~~~k~  174 (276)
T c2yf6A_       115 YDGRDFVAFDKGTMTLTAAVPEAVPTKRKWEEGGYAEGLKQYLEETCVEWLRRYVEYGKA  174 (276)
T ss_dssp            ETTEEEEEEETTTTEEEECSGGGHHHHHHHHHSSSHHHHHHHHHTTHHHHHHHHHHHHHTHH
T ss_pred             ECCCEEEEEEECCCCEEEECCHHHHHHHHHHCCHHHHHHHHHHHHHHHHHHHHHHHHHHHCCC
T ss_conf             759069999268518995482688888872766789999987343438998887620223


Q ss_pred             CCCCCCCCCEEEEEECCCCCEEEEEEEEECEECCCEEEEEEEECCEECCCCCCCCCEEECCC
Q ss_conf             33202555316786068886489999942017732999989878812663035406 5499
Q allis_shad_WA_  178 SLSKDAKPTVRVSLEESEGKEYIMCSVQGFSPNTISVRWVYKGKIVHFARTTTGLLPRKD  237 (273)
Q Consensus       178 ~~~~~~~~~~~p~v~v~~~~~~~~~~~~~~~~~~L~C~a~gFyP~~i~v~W~~~g~~~~~~~~~~~~~~~~~~pn~d  237 (273)
                      .+.+++||.|.|+++++++.++|+|+|+|||++|.|+|++||++++.++.+++++||+|
T Consensus       175 ~l~~~~~~P~v~v~~~~~~~~~~~~~~L~C~~~gFyP~~i~v~W~~~g~~~~~~~~~~~~~~~~pn~d  234 (276)
T c2yf6A_       175 ELGRRERPEVRVWGKEADGILTLSCRAHGFYPRPIVVSWLKDGAVRGQDAQSGGIVPNGD  234 (276)
T ss_dssp            HHHCCBCCEEEEEEEEESSSCEEEEEEEEEBSSCCEEEEEEESSCBCTTTCEECCCEECTT
T ss_pred             CCCCCCCCEEEEECCCCCCCEEEEEEEECEECCCCEEEEEEECCEECCCCEEECCCEECCC
T ss_conf             344566625999814898825999999320478519999998899567513043076499


Q ss_pred             CCEEEEEEEEEECCCCCCEEEEEEEEECCCCCCEEEE
Q ss_conf             74899999998356588699999932688858877
Q allis_shad_WA_  238 GTFQITSYLTLGNKTLQDIVCETEHISIEGTLQAT  272 (273)
Q Consensus       238 gty~~~~~~~l~v~~~~~~~~~~ytC~V~H~sl~~~l~v~  272 (273)
                      ||||++++|+|+++++++++|+|+|+|+|++||++.
T Consensus       235 gtf~~~~~~l~v~~~~~~~~~ytC~V~H~sl~~p~~~~~  269 (276)
T c2yf6A_       235 GTYHTWVTIDAQPGDGDKYQCRVEHASLPQPGLYS  269 (276)
T ss_dssp            SCEEEEEEEEECTTCSTTEEEEEECTTCSSCEEEC
T ss_pred             CCEEEEEEEEEECCCCCEEEEEEEEECCCCCCEEEE
T ss_conf             75899999999202188699999917898776899
```

Representative results of the 3D modeling in the SWISS-MODEL analyses

1. allis shad WA α1

SWISS-MODEL No. 1 template 6blr.1.A Mouse MHC class II IA α1

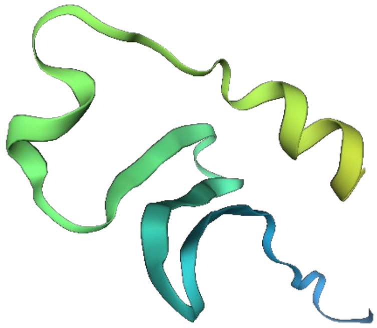

2. allis shad WA α2

SWISS-MODEL No. 1 template 2qri.2.A Mouse MHC class I H-2 Kb β_2-m

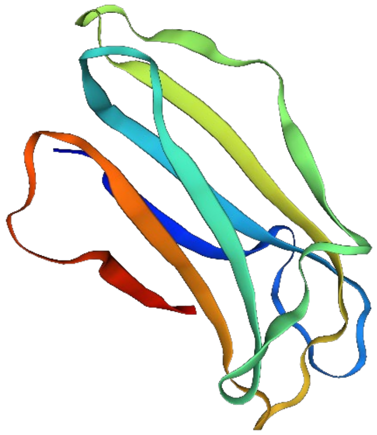

3. allis shad WA all

SWISS-MODEL No.1 template 1aqd.1.B Human MHC class II HLA-DR1

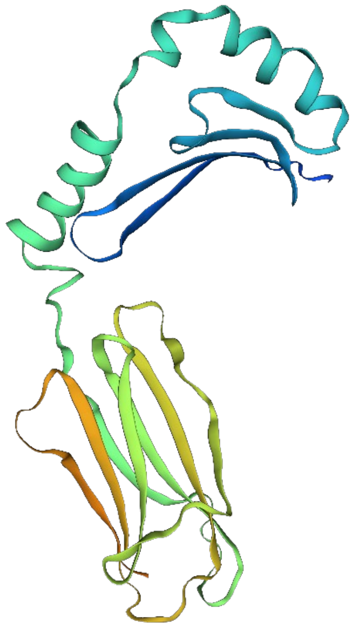

SWISS-MODEL No.6 template 6h6d.1.A Mouse MHC class I H-2 D-B

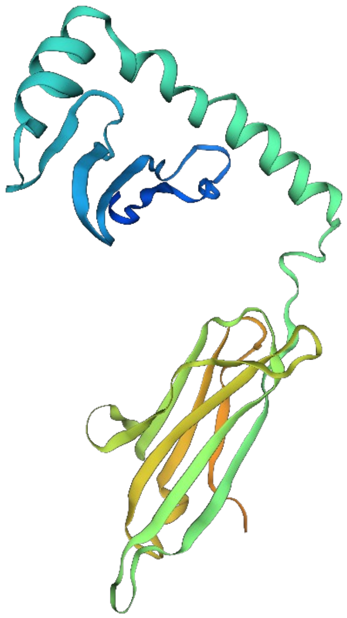

4. allis shad WB all

SWISS-MODEL No. 1 template 3p77.1.A Chicken MHC class I Rfp-Y α2α3

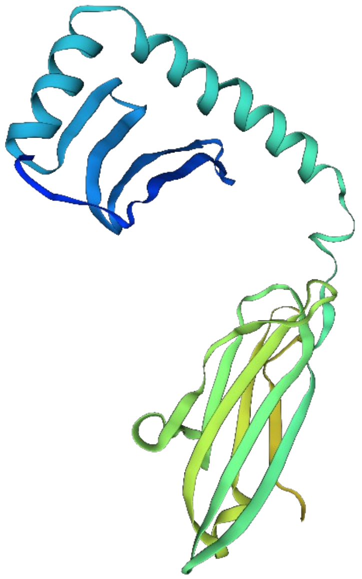

5. allis shad (WA α1 + WB β1β2)

SWISS-MODEL No. 1 template 7cpo.1.A Anolis MHC class I α1α2α3

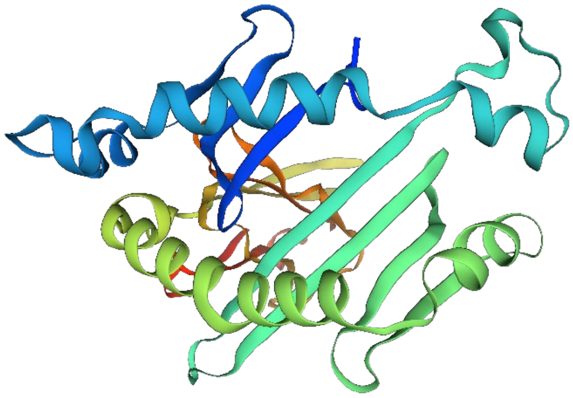

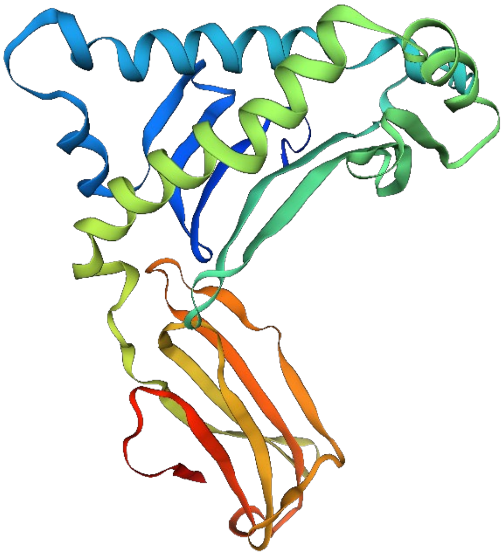

6. allis shad WA / WB

SWISS-MODEL No. 1 template 3pdo.1   Human MHC class I HLA-DR1   (class II-type model)

Within the first half of the α-helix of the WA α1 domain is not formed well.
The template MHC class II molecules generally possess deletions of residues at the corresponding region (Fig. S21).

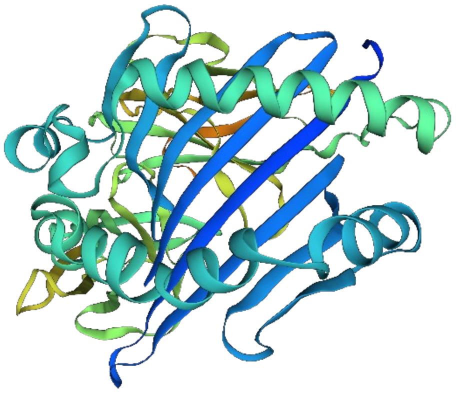

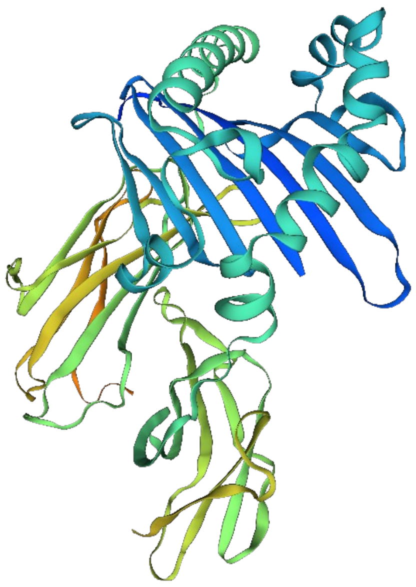

SWISS-MODEL No. 1 template 3pdo.1   Human MHC class I HLA-DR1   (class II-type model)

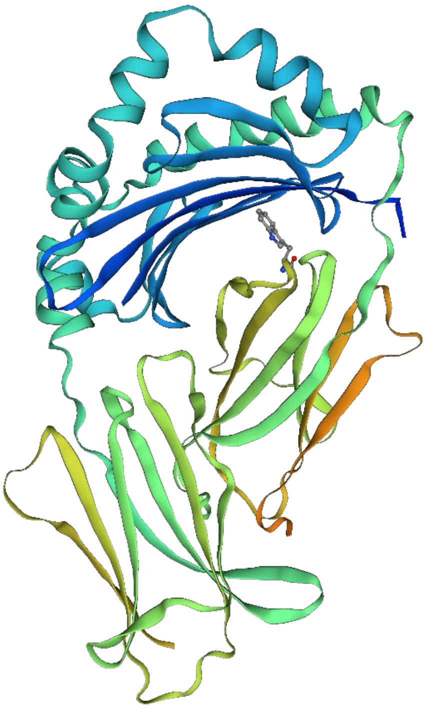

WA α2 W61

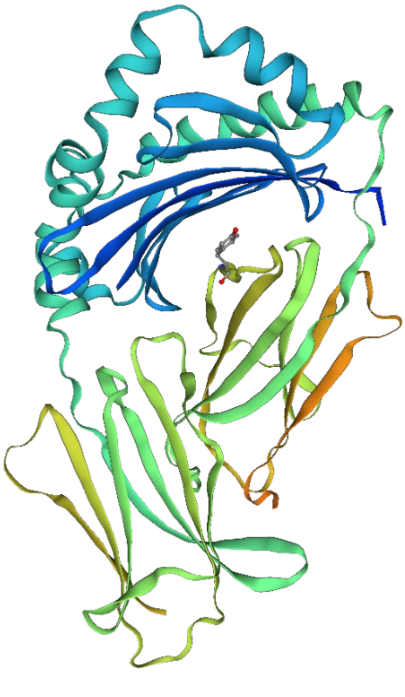

WA α2 Y57

SWISS-MODEL No. 1 template 3pdo.1   Human MHC class I HLA-DR1   (class II-type model)

showing Tryptophan (W)-61 and Tyrosine (Y)-57 of WA α2 domain in the model
together with glutamine (Q)-6 and aspartic acid (D)-37 of WB β1 domain.
β_2-m possesses conserved phenylalanine (F)-57 instead of tyrosine.
β_2-m W61 interacts with Q6 and D37 of the class I α2 domain through hydrogen-bonding.
β_2-m F57 also interacts with Q6 of the class I α2 domain.

Even in the model of the class II-type molecule composed of WA α chain (WA α1α2) and WB β chain (WB β1β2), WB β1 Q6 and WB β1 D37 are situated close to WA α2 W61.

Oxygen: light red
Nitrogen: blue

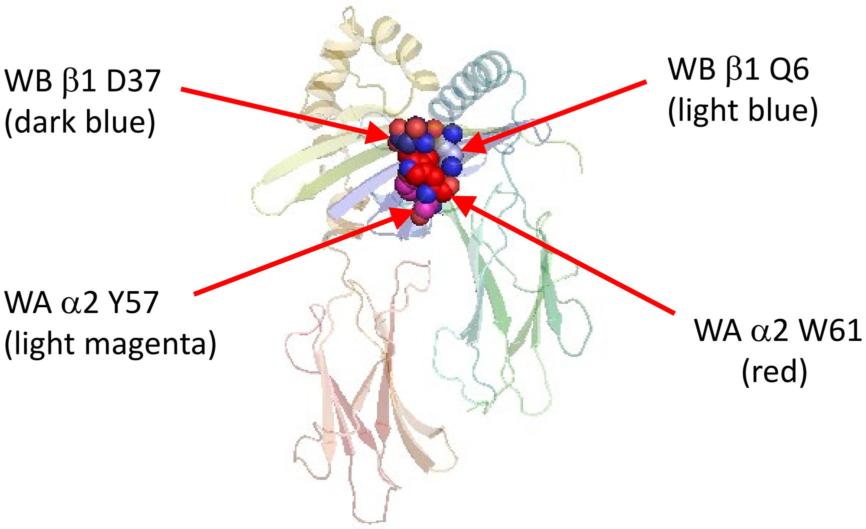

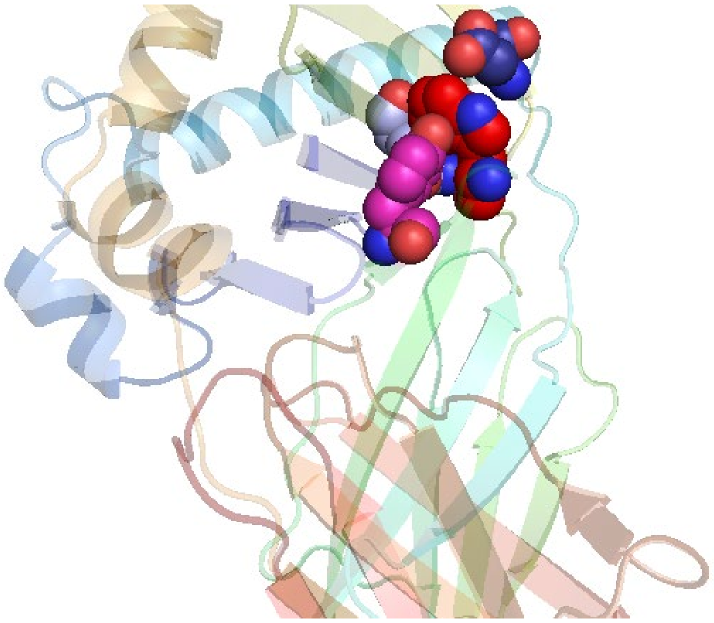

The nitrogen of WAα2 W61 is not so close to the oxygen of WB β1 D37.

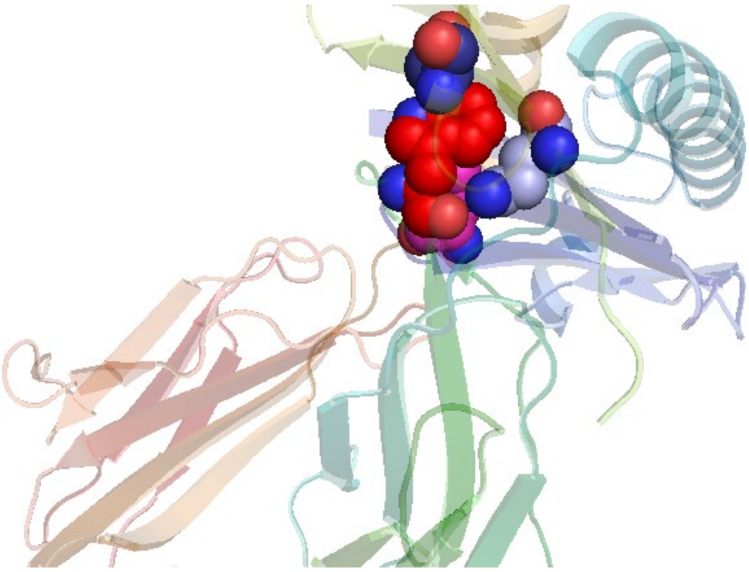

The oxygen of main chain of WAα2 W61 is very close to the nitrogen of WB β1 Q6.

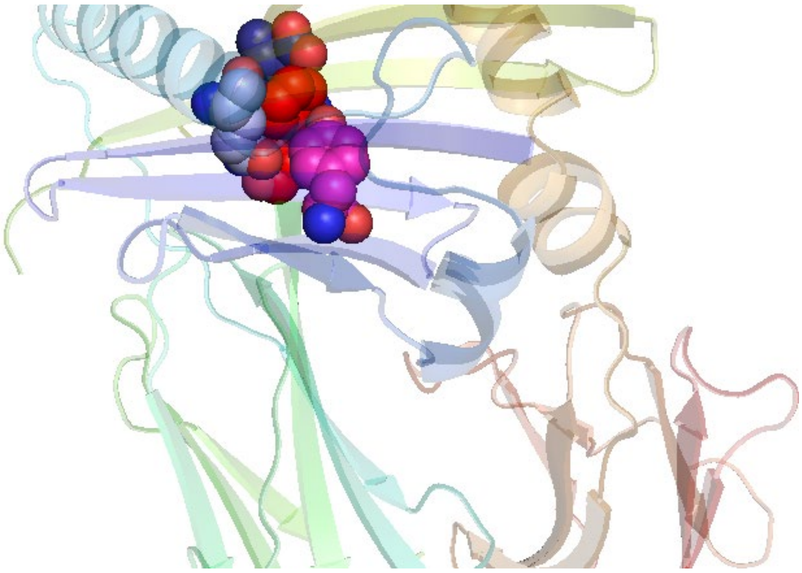

WAα2 Y57 and WB β1 Q6.

SWISS-MODEL No. 1 template 3pdo.1 Human MHC class I HLA-DR1 (class II-type model)

In $\alpha 3/\beta_2$-m interface of MHC class I molecule, the oxygen of main chain of α3P57 and the hydroxy group of β_2-m Y8 form the conserved hydrogen bond.
In this model of W-category molecule, the oxygen of main chain of WBβ2 P57 and the hydroxy group of WAα2 Y8 are distantly located.
Oxygen: light red, Nitrogen: blue

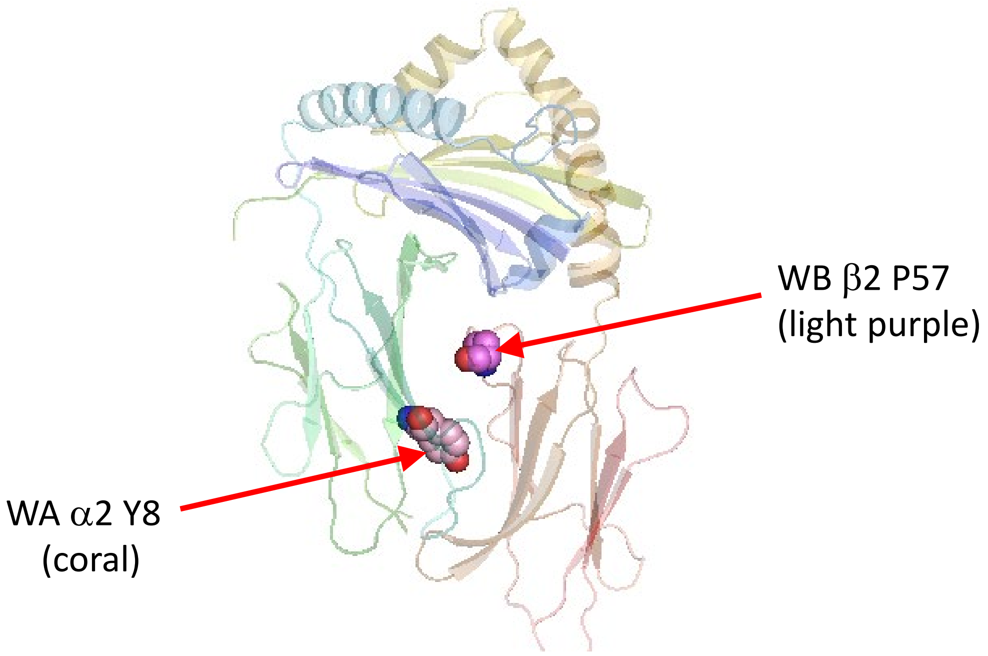

7. allis shad (WA $\alpha 1$ + WB $\beta 1 \beta 2$) / WA $\alpha 2$

SWISS-MODEL No. 1 template 6lhf.1 Chicken MHC class I BF2 (class I-type model)

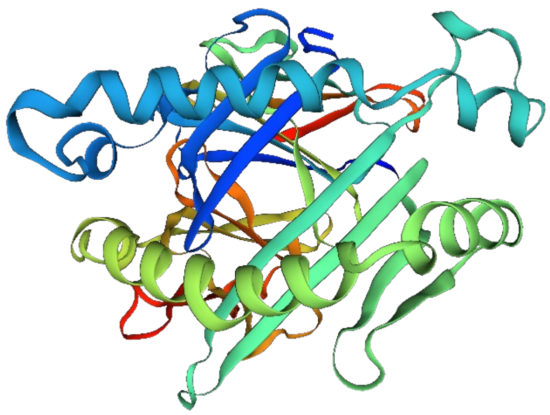

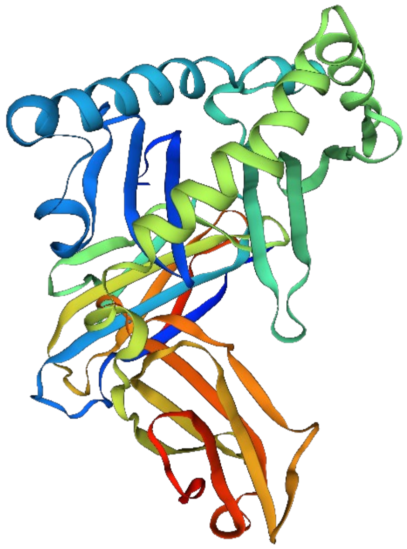

SWISS-MODEL No. 10 template 3wuw.1 Human MHC class I HLA-B57 (class I-type model)

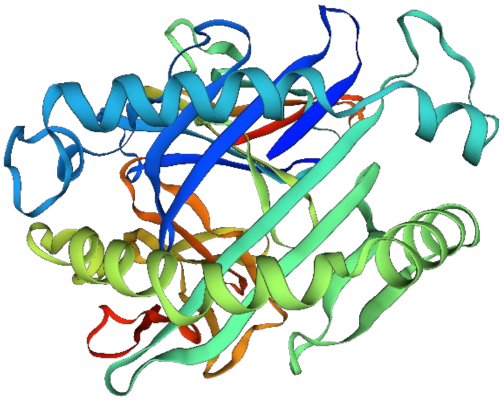

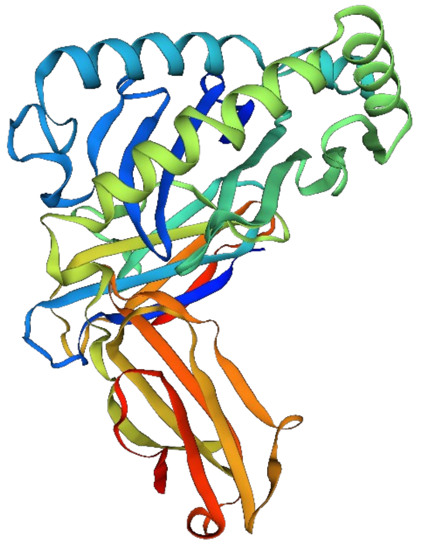

SWISS-MODEL No. 10 template 3wuw.1 Human MHC class I HLA-B57 (class I-type model)

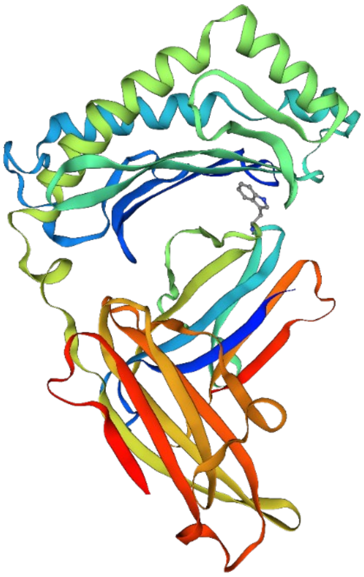

WA α2 W61

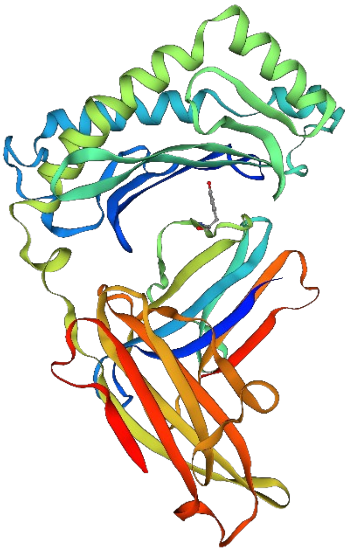

WA α2 Y57

SWISS-MODEL No. 10 template 3wuw.1 Human MHC class I HLA-B57 (class I-type model)

showing Tryptophan (W)-61 and Tyrosine (Y)-57 of WA α2 domain in the model together with glutamine (Q)-6 and aspartic acid (D)-37 of WB β1 domain.
β_2-m possesses conserved phenylalanine (F)-57 instead of tyrosine.
β_2-m W61 interacts with Q6 and D37 of the class I α2 domain through hydrogen-bonding.
β_2-m F57 also interacts with Q6 of the class I α2 domain.
Oxygen: light red, Nitrogen: blue

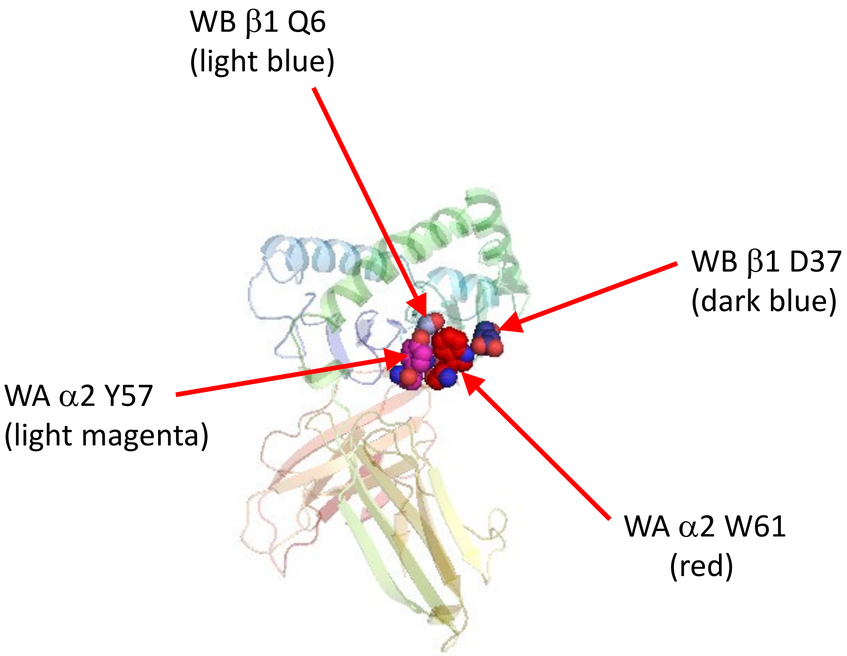

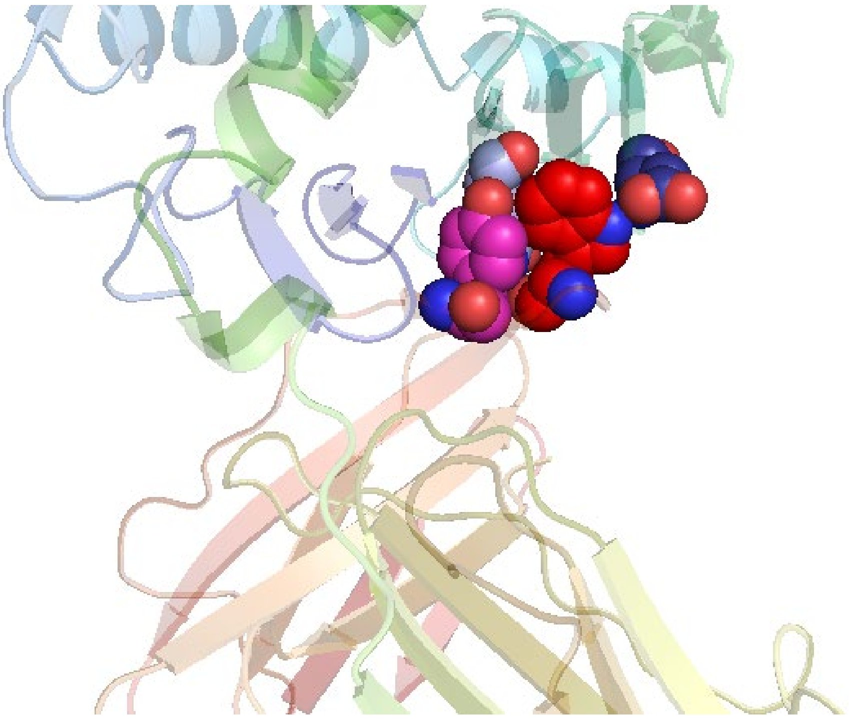

The nitrogen of WAα2 W61 is very close to the oxygen of WB β1 D37.

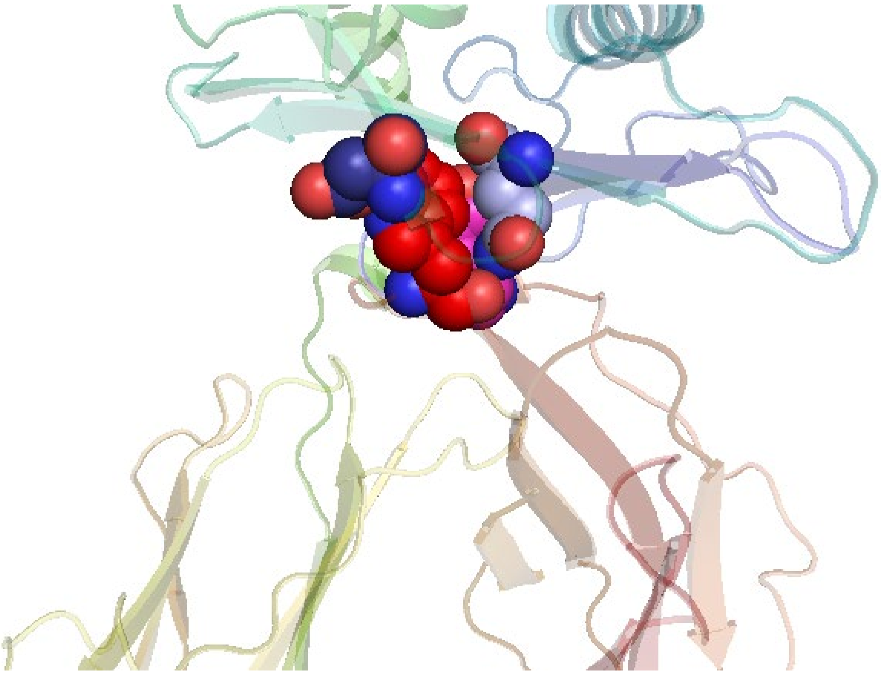

The oxygen of main chain of WAα2 W61 is very close to the nitrogen of WB β1 Q6.

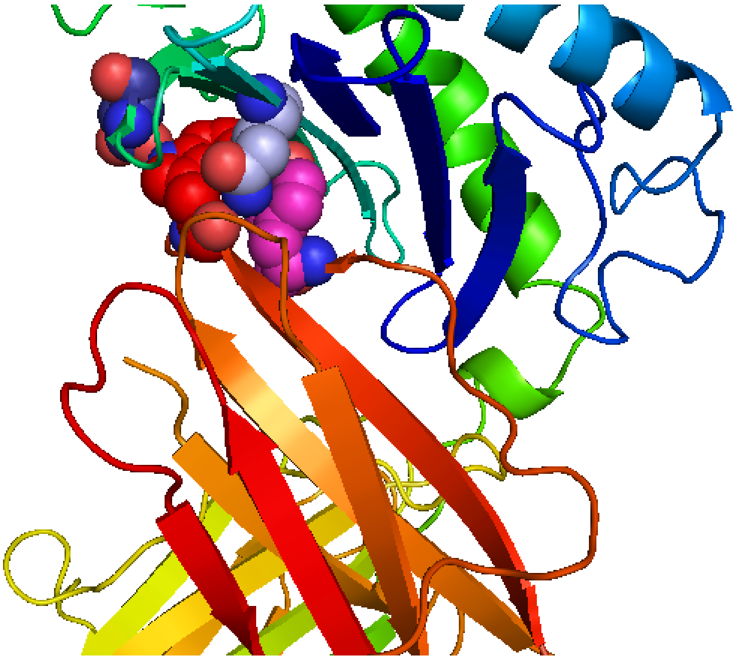

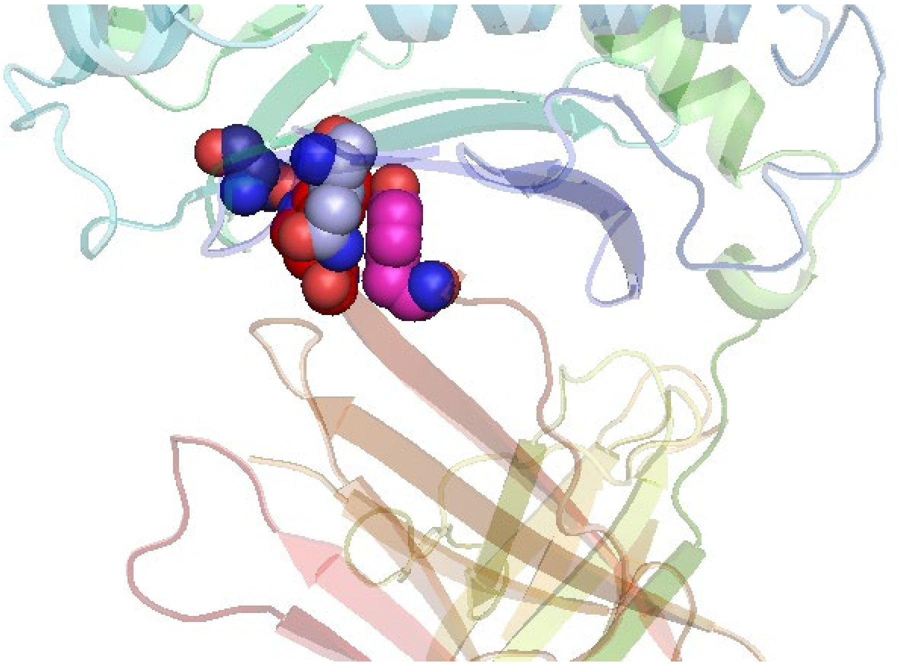

WAα2 Y57 is also close to WB β1 Q6.

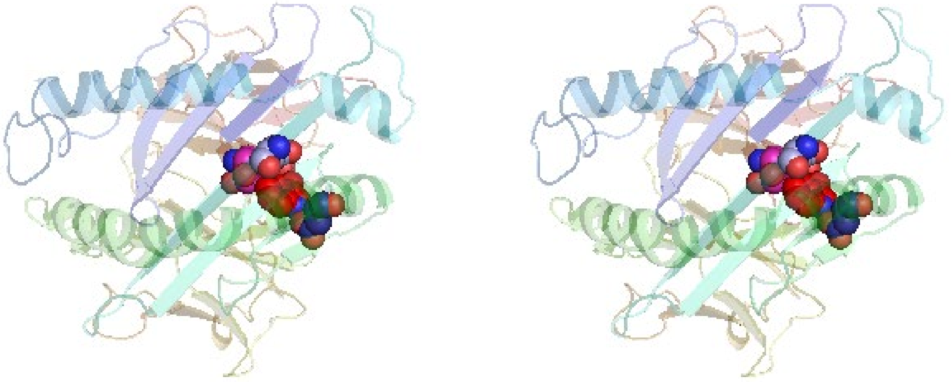

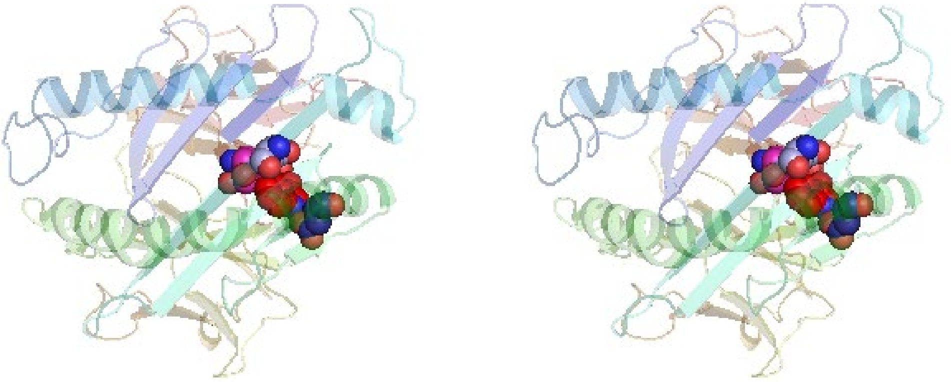

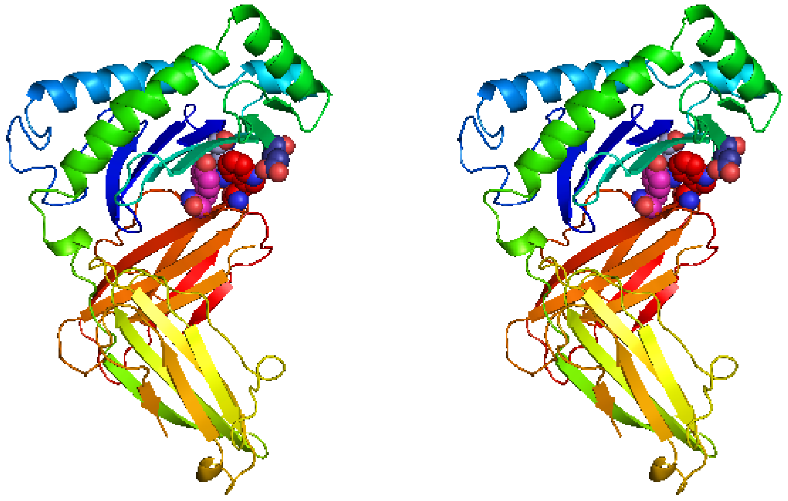

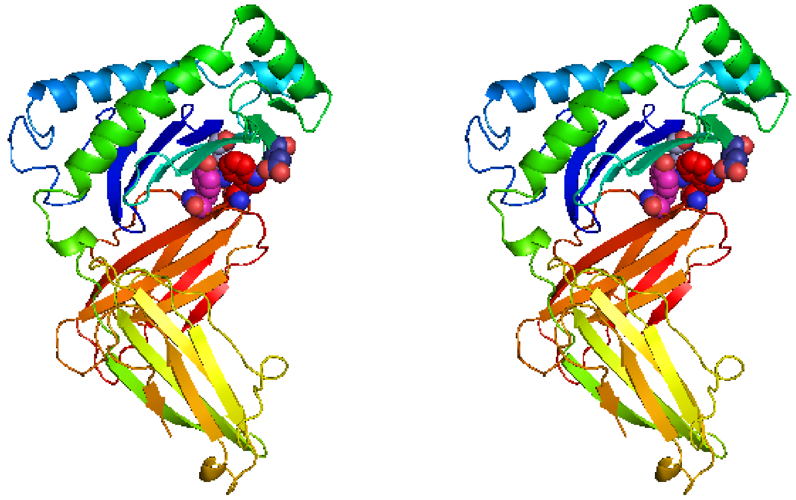

SWISS-MODEL No. 10 template 3wuw.1 Human MHC class I HLA-B57 (class I-type model)

In α3/β_2-m interface of MHC class I molecule, the oxygen of main chain of α3P57 and the hydroxy group of β_2-m Y8 form the conserved hydrogen bond.
In this model of W-category molecule, the oxygen of main chain of WBβ2 P57 and the hydroxy group of WAα2 Y8 are very close to each other.
Oxygen: light red, Nitrogen: blue

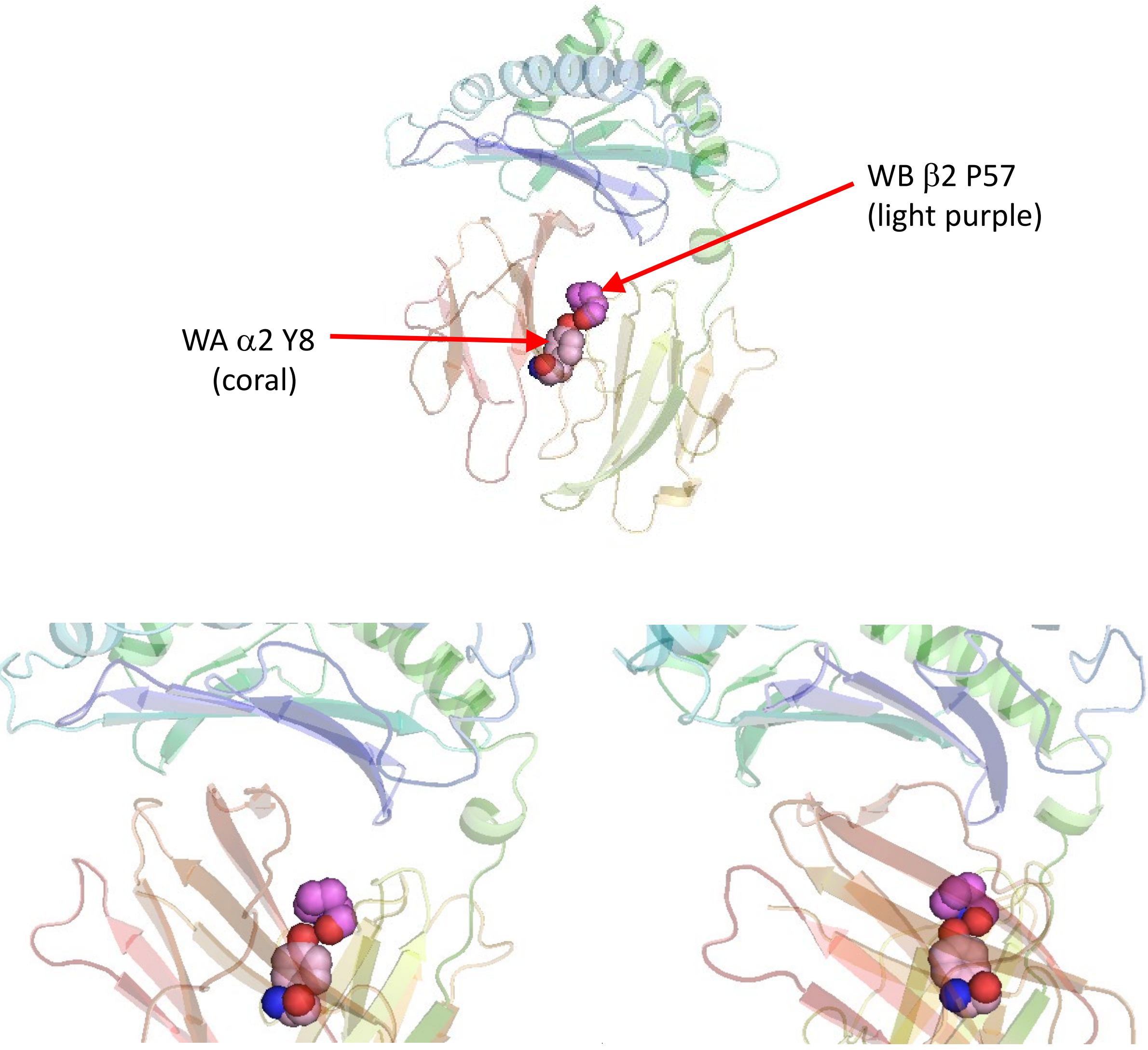

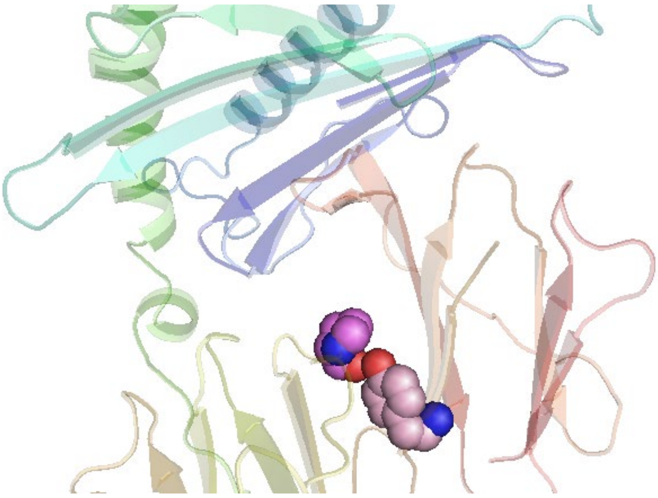

Representative results of the alignments in the SWISS-MODEL analyses

Arrow: β-strand, Ellipse: α-helix

1. allis shad WA α1

SWISS-MODEL No. 1 template 6blr.1.A Mouse MHC class II IA α1

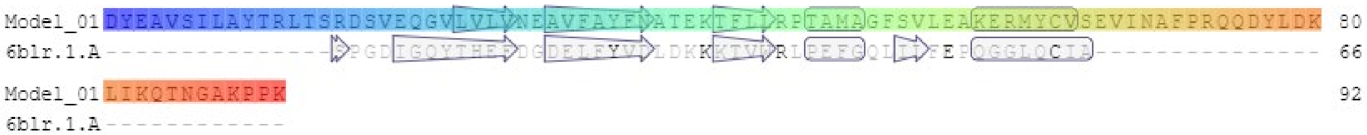

2. allis shad WA α2

SWISS-MODEL No. 1 template 2qri.2.A Mouse MHC class I H-2 K-B β_2-m

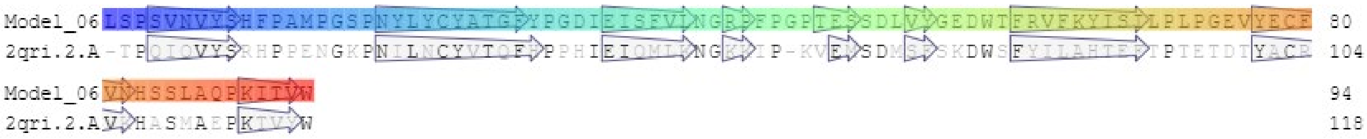

3. allis shad WA all

SWISS-MODEL No.1 template 1aqd.1.B Human MHC class II HLA-DR1 β1β2

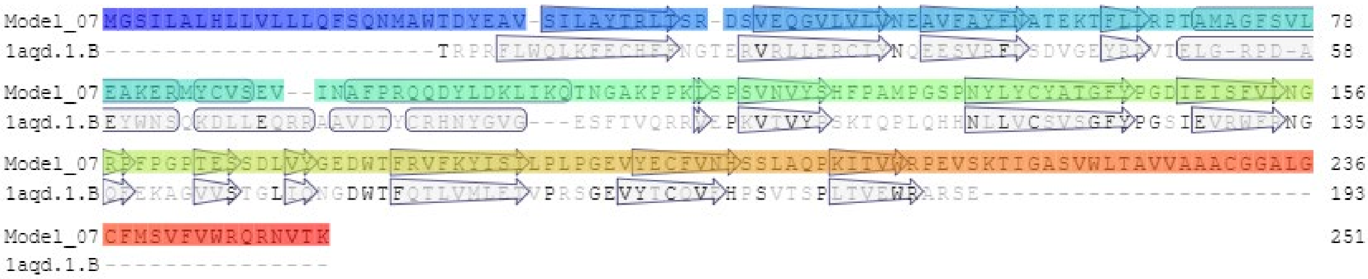

SWISS-MODEL No.6 template 6h6d.1.A Mouse MHC class I H-2 D-B α2α3

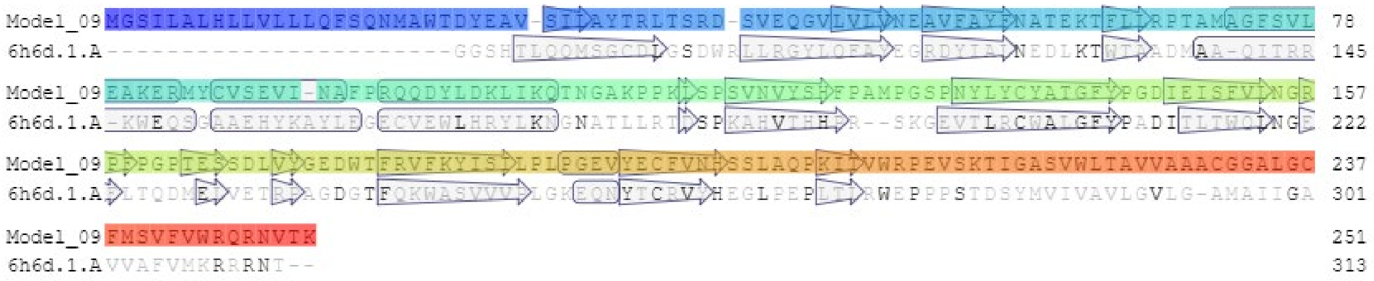

4. allis shad WB all

SWISS-MODEL No. 1 template 3p77.1.A Chicken MHC class I Rfp-Y α2α3

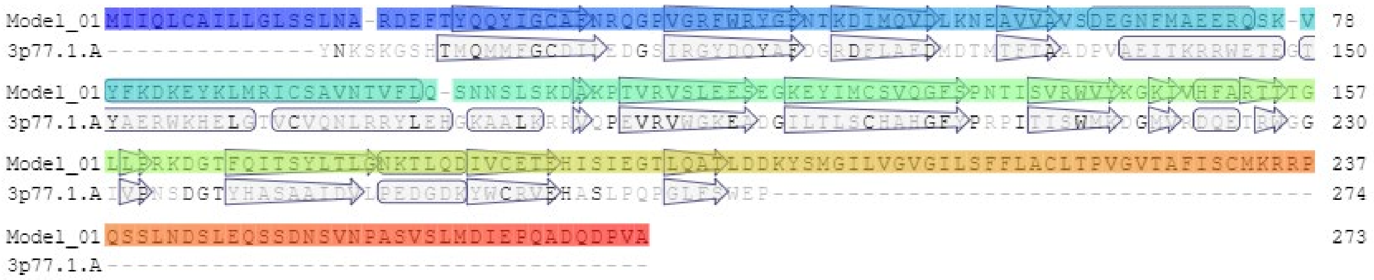

5. allis shad (WA α1 + WB β1β2)

SWISS-MODEL No. 2 template 7cpo.1.A Anolis MHC class I α1α2α3

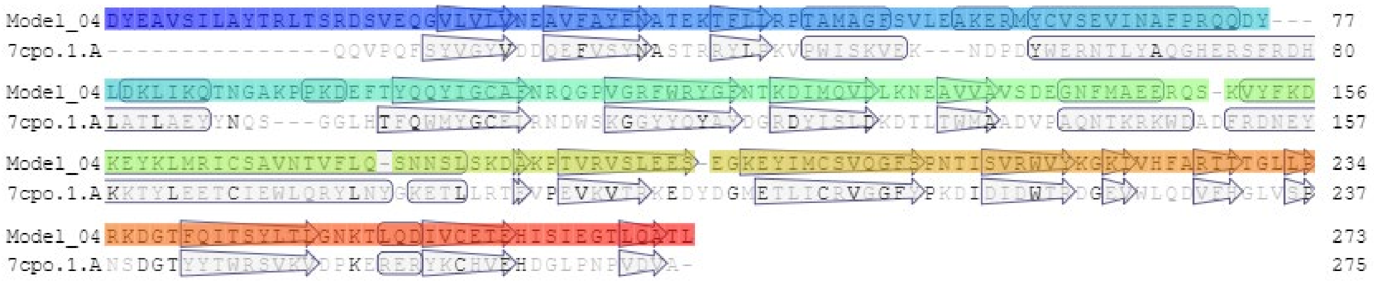

6. allis shad WA / WB

SWISS-MODEL No. 2 template 3pdo.1   Human MHC class II HLA-DR1

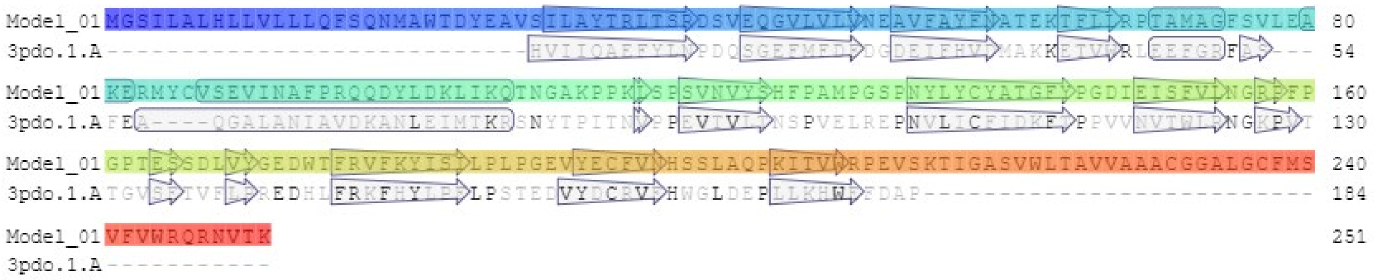

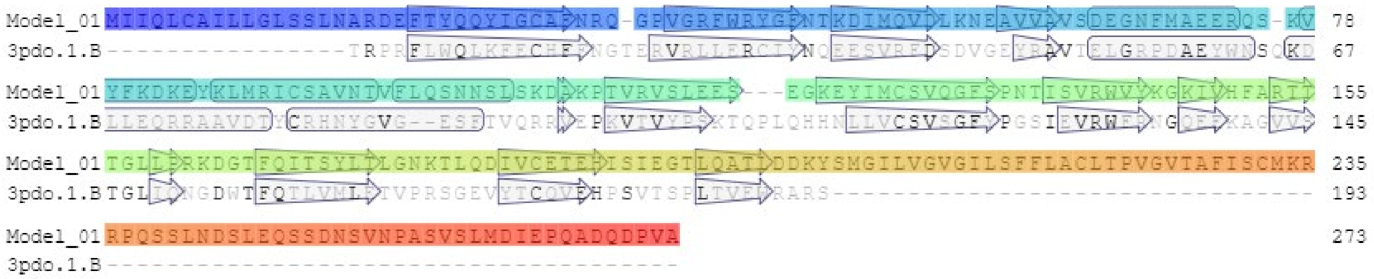

7. allis shad (WA α1 + WB β1β2) / WA α2

SWISS-MODEL No. 1 template   6lhf.1   Chicken MHC class I BF2

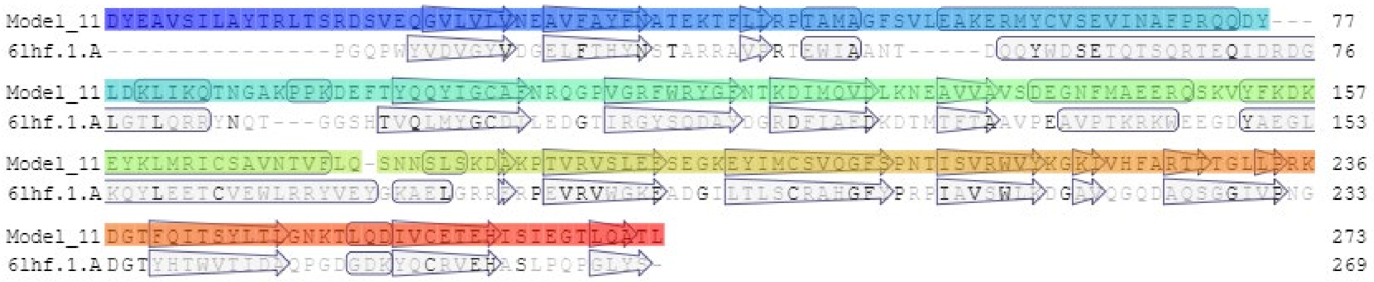

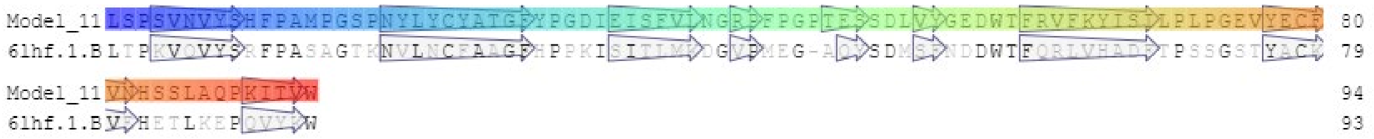

SWISS-MODEL No. 10 template 3wuw.1 Human MHC class I HLA-B57

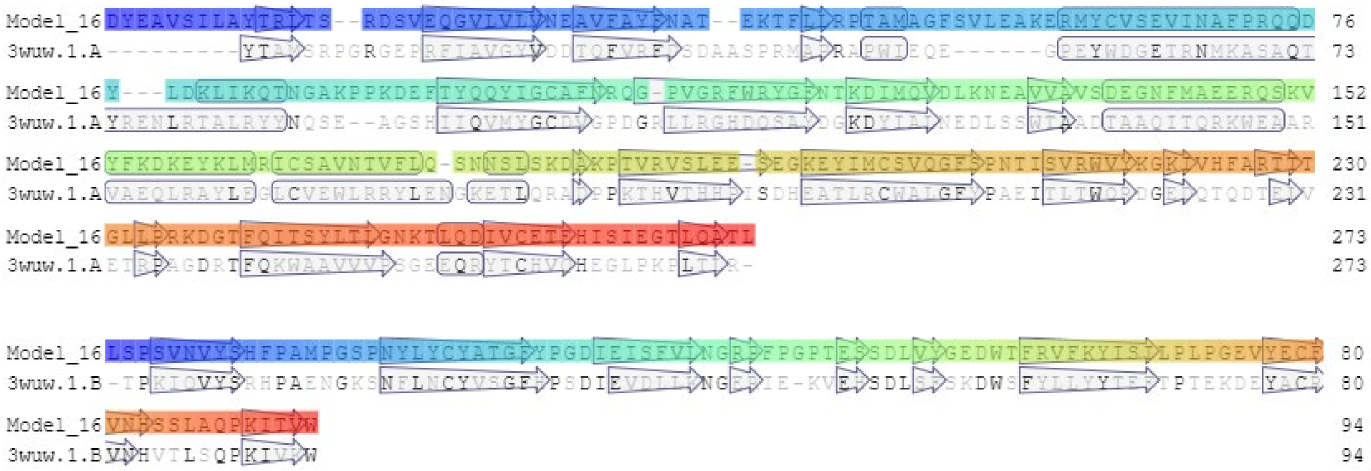

11. Additional explanations for Fig. S5

Figure S5

A

At the corresponding position of β_2-m [Y]67, WA α2 and β_2-m molecules of phylogenetically more primitive animals often possess a lysine or an arginine, and indeed a lysine can be seen at this position in the structure of grass carp β_2-m (PDB ID: 3GBL and 5Y91) forming intra-domain hydrogen bonds (69) (*SI Appendix* and Fig. S7). At the corresponding position of I α3[T]55, many WB β2 and several class I α3 domains possess an isoleucine, and at the corresponding position of I α3[P]95, many WB β2 and several class I α3 possess a lysine.

B

The class II α1 residues described in the preceding part of this legend are not conserved in the corresponding positions of W-category and class I molecules (Dataset S1). However, for inter-domain interactions between the membrane-distal and the membrane-proximal domains, MHC class II molecules use regions roughly corresponding to those used by MHC class I molecules (31). The amino acid residues of HLA-DR1 described above are located in these regions. All the residues of HLA-DR1 involved in the interface interaction between the membrane-distal and the membrane-proximal domains according to ref. 31 are described in the legends of Datasets S1 and S2.

C

Among the residues described hitherto in this legend, several (color-shaded) are involved in inter-domain interactions as follows (29): I α2Q6, I α2A32, I α2G35 and I α2D37 interact with β_2-m W61, I α2Q6 also interacts with β_2-m F57, I α1V30 interacts with the main chain of β_2-m L55 and I α1 R60 interacts with β_2-m D54. I α2K36 interacts with the first residue (an isoleucine) of the mature β_2-m peptide, which is not included in Fig. 3 and Dataset S2 and not conserved among β_2-m. This figure does not show all the residues involved in inter-domain interaction between β_2-m and the membrane-distal domains. In HLA-A2, β_2-m W61 interacts with two more residues of the α2 domain (I α2Q30 and I α2Y31) in addition to those described above. β_2-m W61 appears to play an important role at the interface with such many interactions (two of them are hydrogen bonds, β_2-m W61 to I α2Q6 and β_2-m W61 to I α2D37). All the residues of HLA-A2 involved in the interface interaction between the membrane-distal and the membrane-proximal domains according to ref. 29 are described in the legends of Datasets S1 and S2.

In the MHC class I structure, the β_2-m and class I α3 domains, which are classified as C1-set Ig-like domains, unexpectedly possess asymmetrical structural arrangements (25) as shown in A, namely, they are not related by a dyad (180°) symmetry, whereas several other molecules such as antibodies and T cell receptors show a dyad symmetry in the arrangements of their dimeric C1-set Ig-like domains [e.g., C_H1 and C_L in an Fab, and C_H3 dimer in Fc in case of antibodies (73); Cα and Cβ in case of T cell receptors (74)]. This special asymmetrical structural organization of MHC class I Ig-like domains is essentially also observed for the two Ig-like domains (class II α2 and β2 domains) of an MHC class II molecule (27) (see B). An MHC class I molecule can superimpose onto an MHC class II molecule although there is a small difference in angle for the arrangements between the class I α3 and class II β2 domains (27).

In this figure, human HLA-A2 (PDB ID: 1AO7) and human HLA-DR1 (PDB ID: 1DLH) are used as representative MHC class I and conventional class II molecules, respectively. For class I molecules, overall structures largely similar to that of HLA-A2 have been reported for other classical class I molecules, e.g., mouse (30) (PDB ID: 2VAA), chicken (75) (PDB ID: 3BEV) and grass carp (PDB ID: 5Y91). Topologically similar overall structures also have been found for highly divergent class I molecules including, for example, mouse CD1 (76) (PDB ID: 1CD1), chicken CD1 (77) (PDB ID: 3DBX) and rat FcRn (78) (PDB ID: 1EXU). In these class I structures, several conserved residues can be observed at the interface between the membrane-distal and the membrane-proximal domains described in this legend including β_2-m W61. Also for class II molecules, largely similar overall structures with highly conserved W61 in the β2 domain have been reported

for the DM molecules of human (PDB ID: 1HDM) and mouse (PDB ID: 1K8I), whose sequences are highly divergent from the classical MHC class II molecules (Datasets S1 and S2).

In the figure, only for the residues relevant for the discussion, their side chains are shown as a ball-and-stick representation. The MHC class I domains are colored as follows: yellow, green, blue and red for α1, α2, α3 and β_2-m, respectively. The MHC class II domains are colored as follows: salmon pink, magenta, light green and light blue for II A α1, II A α2, II B β1 and II B β2, respectively. Each pair of corresponding domains of MHC class I and class II molecules (class I α1/class II α1, β_2-m/class II α2, class I α2/class II β1 and class I α3/class II β2, see Fig. 8) possesses related colors. The highlighted amino acid residues with a ball-and-stick representation have colors similar to those of the domain to which the relevant residue belongs. Bound peptides are shown in the color purple. Disulfide bridges formed by two cysteine residues are shown in dark brown color.

The numbers of amino acid positions are based on Dataset S1 for the membrane-distal domains, and Fig. 3/ Dataset S2 for the Ig-like domains. The corresponding amino acid numbers in HLA-A2 (ref. 29) or the respective chain of HLA-DR1 (ref. 31) for the residues used in the figure are as follows: the numbers in HLA-A2 are shown in parentheses, I α1S4 (4), I α1R21 (17), I α1G22 (18), I α1V30 (25), I α1V33 (28), I α1T36 (31), I α1F38 (33), I α1F41 (36), I α1R60 (48), I α2H3 (93), I α2Q6 (96), I α2G11 (100), I α2A32 (117), I α2G35 (120), I α2K36 (121), I α2D37 (122), I α3T55 (233), I α3P57 (235), I α3G61 (239), I α3P95 (269), β_2-m L37 (39), β_2-m L55 (54), β_2-m F57 (56), β_2-m W61 (60), β_2-mY67 (66); the numbers in HLA-DR1 are shown in parentheses, IIA α1F34 (26), IIA α1L72 (45), IIA α1F75 (48), IIA α2H61 (143). IIB β2W61 (153).

12. Notes for Datasets

Dataset S1: Amino acid comparison of the membrane-distal domains of W-category and other MHC molecules.

The classification of molecules is shown in the first column. The following classification is used: WA α1 (W-category α chain α1 domain), I α1 (class I α1 domain), I nc α1 (nonclassical class I α1 domain), IIA α1 (class II α chain α1 domain), DOA α1 (α1 domain of HLA-DO α chain related), DMA α1 (α1 domain of HLA-DM α chain related), IIA nc α1 teleost (nonclassical class II α chain α1 domain of teleost fish), WB β1 (W-category β chain β1 domain), I α2 (class I α2 domain), I nc α2 (nonclassical class I α2 domain), IIB β1 (class II β chain β1 domain), DOB β1 (β1 domain of HLA-DO β chain related), DMB β1 (β1 domain of HLA-DM β chain related), IIB nc β1teleost (nonclassical class II β chain β1 domain of teleost fish).

The shading principles are as follows:
Red, residues which are shared between WA α1 and class I α1 domains but very uncommon in classical class II α1 domains;
Blue, residues which are shared between WB β1 and class I α2 domains but very uncommon in classical class II β1 domains;
Purple, conserved residues interacting with bound peptide termini in classical class I (the same residues at the same position in most of the other aligned domain types are also shaded with purple color for comparison, but in case of irrelevant class I domains, those are shaded with pale orange color);
Moss green, conserved residues interacting with a bound peptide in classical class II (the same residues at the same position in most of the other aligned domain types are also shaded with moss green color for comparison, but in case of irrelevant class II domains, those are shaded with pale orange color);
Dark gray, conserved tryptophans in class I α1 domains (position 73) and class I α2 domains (position 57);
Light green, residues found in many teleost WB and classical class II β1 domains;
Pale gray, N-glycosylation motif NX(S/T) found in the last part of domains, all residues of N and/or (S/T) at the same positions are shaded irrespective of the completeness of the motif;
Orange, residues frequently observed in WB β1, class I α2 and class II β1 but not in WA α1, class I α1 and also not in class II α1 except position 33;
Pink and magenta, specific for WA α1 domain (F57) and WB β1 domain (Q7, C27 and W29), respectively;
Black, highly conserved cysteines in class I α2 and class II β1 domains, and also in DM α1 domains and bony fish and some cartilaginous fish class II α1 domains;
Yellow, commonly observed in various MHC membrane-distal domains;
Bright purple, "LN" motif observed in banded houndshark WB_DS1 and WB_DS3;
Dark brown, conserved tryptophan in class II α1 domain (position 59), which is important for the interaction with DM molecules (79);
Pale blue, three residues with this color-shading in the last part of domains are highly conserved in class I α2 domains.

The amino acid residues in the membrane-distal domains of the human classical MHC class I HLA-A2 molecule which participate in the inter-domain interactions between the membrane-distal domains and the Ig-like domains are as follows (ref. 29) (for convenience, in the following listing, not only the residue numbering of this figure but also, in parentheses, the residue numbering of HLA-A2 in ref. 29 is shown):
I α1/α2 with β_2-m, I α1F8 (8), I α1F10 (9), I α1T11 (10), I α1V13 (12), I α1I28 (23), I α1V30 (25), I α1Y32 (27), I α1Q37 (32), I α1R40 (35), I α1R60 (48), I α2T4 (94), I α2Q6 (96), I α2Q30 (115), I α2Y31 (116), I α2A32 (117), I α2D34 (119), I α2G35 (120), I α2K36 (121), I α2D37(122); I α1/α2 with I α3, I α1D34 (29), I α1D35 (30), I α1T36 (31), I α2L124 (179), I α2R126 (181), I α2T127 (182).

In case of human classical MHC class II HLA-DR1 molecule, the amino acid residues in the membrane-distal domains which participate in the inter-domain interactions between the membrane-distal domains and the Ig-like domains are as follows (ref. 31) (for convenience, in the following listing, not only the residue numbering of this figure but also, in parentheses, the residue numbering of the respective chain of HLA-DR1 in ref. 31 is shown):

II α1/β1 with II α2/II β2, II α1I6 (8), II α1A8 (10), II α1F11(12), II α1L13 (14), II α1D16 (17), II α1E28 (21), II α1M30 (23), II α1F33 (26), II α1D34 (27), II α1G35 (28), II α1D36 (29), II α1E37 (30), II α1I38 (31), II α1H40 (33), II α1R60 (44), II α1L71 (45), II α1F74 (48), II β1R2 (6), II β1L4 (8), II β1Q6 (10), II β1K8 (12), II β1R30 (29), II β1F32 (31), II β1Q35 (34).

The sequences used in this figure are listed in Table S3.

Dataset S2: Amino acid comparison of the membrane-proximal, Ig-like domains of W-category and other MHC molecules.

The classification of molecules is shown in the first column. The following classification is used: WA α2 (W-category α chain α2 domain), β_2-m (β_2-microglobulin), IIA α2 (class II α chain α2 domain), DOA α2 (α2 domain of HLA-DO α chain related), DMA α2 (α2 domain of HLA-DM α chain related), IIA nc α2 teleost (nonclassical class II α chain α2 domain of teleost fish), WB β2 (W-category β chain β2 domain), I α3 (class I α3 domain), I nc α3 (nonclassical class I α3 domain), IIB β2 (class II β chain β2 domain), DOB β2 (β2 domain of HLA-DO β chain related), DMB β2 (β2 domain of HLA-DM β chain related), IIB nc β2 teleost (nonclassical class II β chain β2 domain of teleost fish).

The shading principles are as follows:

Red, residues which are shared between WA α2 and β_2-m domains but very uncommon in classical class II α2 domains;

Blue, residues which are shared between WB β2 and class I α3 domains but very uncommon in classical class II β2 domains;

Green, often found in WA α2, β_2-m and class IIA α2;

Light blue, a tryptophan (position 37) conserved in many Ig-superfamily members and observed except WA α2, β_2-m, and some class I α3;

Orange, observed in class I α3 and class IIB β2;

Purple, a leucine residue (position 70) conserved in conventional class II α2 and β2 domains;

Black, highly conserved cysteines in Ig-like domains;

For the co-receptor CD8 binding to classical MHC class I molecule, I α3Q48 [226 in HLA-A2 (ref. 29)] is the most important residue (80, 81). This glutamine residue is generally not conserved in I α3-corresponding WB sequences. Only banded houndshark WB_DS1 and WB_DS3 possess a glutamine residue at this position.

For the co-receptor CD4 binding to a classical MHC class II molecule, the following residues of the class II molecule have been reported to interact with CD4 (82) (for convenience, in the following listing, not only the residue numbering of this figure but also, in parentheses, the residue numbering of the respective chain of HLA-DR1 in ref. 31 is shown): II α2E4(88), II α2T6(90), II α2K99(176), II β2S10(104), II β2L20(114), II β2V22(116), II β2M50(142), II β2V51(143), II β2S52(144), II β2T54(145), II β2I57(148), II β2L67(158), II β2M69(160), II β2E71(162).

The amino acid residues in the Ig-like domains of the human classical MHC class I HLA-A2 molecule which participate in the inter-domain interactions between the membrane-distal and the membrane-proximal Ig-like domains are as follows (ref. 29) (for convenience, in the following listing, not only the residue numbering of this figure but also, in parentheses, the residue numbering of HLA-A2 heavy chain and β_2-m in ref. 29 is shown): β_2-m with I α1/α2, β_2-m I (not included) (1), β_2-m R1 (3), β_2-m H29 (31), β_2-m S31 (33), β_2-m D54 (53), β_2-m L55 (54), β_2-m S56 (55), β_2-m F57 (56), β_2-m W61 (60), β_2-m F63 (62), β_2-m Y64 (63); α3 with I α1/α2, I α3D1 (183), I α3Y29 (209), I α3P30 (210), I α3A31 (211), I α3F63 (241), I α3E89 (264).

In case of human classical MHC class II HLA-DR1 molecule, the amino acid residues in the Ig-like domains which participate in the inter-domain interactions between the membrane-distal and the membrane-proximal Ig-like domains are as follows (ref. 31) (for convenience, in the following listing, not only the residue numbering of this figure but also, in parentheses, the residue numbering of the respective chain of HLA-DR1 in ref. 31 is shown): II α2 with II α1/β1, II α2T29 (113), II α2P30 (114), II α2P31 (115), II α2V32 (116), II

α2T53 (135), II α2V54 (136), II α2F55 (137), II α2L56 (138), II α2P57 (139), II α2R58 (140), II α2E59 (141), II α2D60 (142), II α2H61 (143), II α2L62 (144), II α2F63 (145), II α2R64 (146), II α2Y68 (150), II α2W89 (168); II β2 with II α1/β1, II β2Y29 (123), II β2Q57 (149), II β2G59 (151), II β2D60 (152), II β2W61 (153).

At position 61, several C1-set Ig-like domains of other molecules, such as tapasin (57), some B7 members (83), antibodies (e.g., 57, 78) and signal-regulatory protein (SIRP) α (84), possess a glycine. Thus, a glycine is one of the candidate residues at position 61 in the primordial class II molecule.

In the oriental weatherfish WB_01 sequence, two amino acid residues (SW) between positions 37 and 38 are not included.

In the outgroup sequences used in the phylogenetic analyses, the following amino acid residue(s) are not included after the amino acid position numbers indicated. In the house mouse IgM sequence: 15, N; 39, R; 59, GA; 77, N. In the human IgM sequence: 15, N; 39, R; 59, QA; 77, N. In the house mouse TCRB sequence: 11, KA; 55, A; 74, FW; 75, N; 91, SEEDKWPEGSPKPV. In the human TCRB sequence: 11, EA; 55, P; 59, PALN; 74, FW; 75, N; 91, SENDEWTQDRAKPV. In the house mouse tapasin: 15, AP; 32, G; 40, GGPGGSS; 77, KQ. In the human tapasin: 15, AP; 32, G; 40, GGPGGRS; 77, EQ.

For the phylogenetic tree analyses, the following three regions in which the three outgroup molecules exhibit high structural variations compared to the MHC molecules were not included: the amino acid position numbers 11-18, 73-80 and 92-93.

The sequences used in this figure are listed in Table S3. The numbers of amino acid positions shown at the top equal those in Fig. 3 and the positions with asterisks are not included for numbering.

Dataset S3: Conservation of α chain or β chain-specific motifs in the TM region of the W-category molecules.

CP/TM/CY region sequences of W-category and other class II molecules are compared. The classifications of molecules are indicated in the first column. In the TM region, W-category sequences possess heptad repeats of glycine residues reported previously for conventional class II sequences (24, 79, 85, 86). MHC class I molecules do not display these features. Other small amino acid residues, e.g., an alanine (A) or a serine (S), can often replace a glycine. Notable differences between class II α and β chains are those at positions 21 and 31. Three residues (F, L, A) between positions 27 and 28 of fat head minnow WB_01 are not included. The shading colors are used as follows: purple, cysteine (C); brown, glycine (G); green, alanine (A) or serine (S).

13. Detailed Author Contributions

K.O. contributed to experiments including isolation of cDNA library clones of banded houndshark *WB_DS1* and *WB_DS3*, isolation of *WB_DS3* genomic clones and their initial sequence determination, isolation of *WA_DS10*, isolation of salamander W-category cDNAs, Southern blot analyses, linkage and expression experiments for banded houndshark W-category genes, and experiments regarding β_2-*m*. J.M.D. contributed to the planning for the expansion of the study with identification of W-category genes from various animals including little skate, various teleost fish, coelacanth, lungfish and salamander, isolated cDNAs from lungfish, goldfish, Mexican tetra, salamander and zebrafish, determined their sequences, and performed the study on polymorphism of zebrafish W-category genes. K.T. contributed to the identification and isolation of cDNAs of banded houndshark *WA_DS5*, isolated cDNAs of banded houndshark *WB_DS1* and *WB_DS3* using RT-PCR, performed intensive study of determination of the genomic organizations of banded houndshark *WA_DS5*, *WA_DS10* and *WB_DS3*, determined the partial genomic organization of *WB_DS1_n1*, and conducted experiments using recombinant proteins of W-category molecules. U.G. performed extensive searches for MHC sequences in the teleost fish genome databases and identified unclassified MHC sequences from the zebrafish database. G.F.W. performed the study of the sequences of the common carp genome and identified the duplicated common carp W-category genes. A.K. performed the experiments of the expression of the zebrafish W-category genes and studied salamander class I sequences for comparison. H.Y. performed comparative structural analyses of MHC sequences using Chimera program for the basis of sequence alignments. K.H. conceived the study, performed the initial experiments including isolation of DNA fragments of banded houndshark *WA_DS5* and *WB_DS3*, and banded houndshark genomic library screening, contributed to the identification of banded houndshark *WA_DS10*, identified *WA_Nds3L* and *WB_Nds5L*, analyzed conservation profile of teleost fish W-category molecules, conducted database searches, phylogenetic estimation, and molecular modeling, analyzed the results, and wrote the manuscript with great helps from J.M.D., K.T., K.O., U. G. and G. F. W.

Fig. S1

A Shark/skate WA_DS5

```
                                    Leader              α1
banded_houndshark_WA_DS5_n1_01        1 MELRNFLIIIAAVTAGTRAAI-EF-ESVSEKLYYSYTP-DSKRNIDIFSIDSIPFMVYDYGEKMFVLNGNVTDGIKELGQKEVTYYTERA 87
banded_houndshark_WA_DS5_n2_01        1 MELGNFLIIIAAVTAGTRAAI-EF-ESISAMLYYSYTP-DSKRNIDIFSIDSIPFMVYDYREKMFVLNGNVTDGIKELGQKEVTYYTERA 87
banded_houndshark_WA_DS5_n3_01        1 MELRNFLIIIAAVTAGTRAAI-EF-ESVSAMLYYSYTP-DSKRNIDIFSIDSIPFMVYDYREKMFVLNGNVTDGIKELGQKEVTYYTERA 87
banded_houndshark_WA_DS5_n4_01        1 MELRNFLIIIAAVTAGTRAAI-EY-KSVSERLYYSHTE-DEETNGHIFTANAFPFLYYDNKKLKFVIHGQETDGIQELGQEEVTHFKERA 87
cloudy_catshark_WA_DS5_like_82K       1 MELWSFLIMISSLTAVTRAEIGEYVESISARIYYRSAP-DSTRNIYVFTINYIPFLLYDFETQKFVLNGKMTEGLKELGQEEVIYYKAMA 89
cloudy_catshark_WA_DS5_like_159K      1 MELWSFLIIISSLTAVTRAEI-DV-ESISIYQYYGYTP-DSENSLSSFAINAFPFMFMHHGLKNFIINGNVTNGKNVLGEDEATFFREIT 87
great_white_shark_WA_DS5_like         1 MELCSSLIIIASLTTVTWAEI-EV-ESVSARLYYSYTP-DSKRNVDIFNINSIPFLFYDYGAQMFVLNGNVTDGIKELGQEEVTYYTERA 87
whale_shark_WA_DS5_like               1 ----------------------V-SAVTESVYYVHTA-HSENILDIFSINYVPFLFYNYGAQTFVINGQVTDGIRELGQEEVDYYHERA 65
zebra_bullhead_shark_WA_DS5_like      1 MELCGFLIIIASLTTVTRAAI-EF-ESVSARLYYSNTL-DLERNIDIFNINSIPFLFYDYGALMFVLNGKMTDGVEELGQEEVTYFKERA 87
spiny_dogfish_WA_DS5_like             1 ----------------------EF-KTISERLYYSYSS-DSETNGHIFDINASPFLFYDYKMHTFVIHGNVTNGIEELGQEEVTHFKERA 66
little_skate_WA_DS5_like              1 MGLRGLLTALLSLAAAV-AEI-EI-ETVSEHFVYGRSTSGLEKTLDILSVNSVPFLAYDHEARAFSINGQATDGREELGEEEVTYFVQRA 87

                                                                        α2
banded_houndshark_WA_DS5_n1_01       88 RGFSTRLQTITEEMIKLTNA------KTIISKKPVVHIYTKEDYAPHHANTLYCYAEKFYPFEIGVTFLVNGRPFAGLVNSSQLVVESDW 171
banded_houndshark_WA_DS5_n2_01       88 RGFSTRLQTITEEMIKLTNA------KTIISKKPVVHIYTKEDYAPHHANTLYCYAEKFYPFEIGVTFLVNGRPFAGLVNSSQLVVELDW 171
banded_houndshark_WA_DS5_n3_01       88 RGFSTRLQTITEEMIKLTNA------KTIISKKPVVHIYTKEDYAPHHANTLYCYAEKFYPFEIGVTFLVNGRPFAGLVNSSQLVVELDW 171
banded_houndshark_WA_DS5_n4_01       88 SGIQAGLQGIAKEITRLTKG------SSVLKEKPVVHIYTEEDYAPHHANTLYCYAEKFYPFEIGVTFLVNGRPFTGLVNSSQLVVESDW 171
cloudy_catshark_WA_DS5_like_82K      90 RGLNVRLQKYTNEMIKLTSASSTITSKSLIYKKPLVHIYTKKIYAPHQSNILFCYAENFYPFEIDITFLINGRPFTGLVNSSQLMIEMDW 179
cloudy_catshark_WA_DS5_like_159K     88 RRFQLRLQRLTKELVKQTGT-RIVHSKLLIYKKPVVHIYAEENYTPGQPNTLYCYAEKFHPYEIEVTFLVNGRPFTGPSESSETMVAADW 176
great_white_shark_WA_DS5_like        88 RGFNTRLQTITEEIIKLTNA------KSIIHKKPVVHIYTKENYTPRESNTLFCYAEKFYPFEIDITLLINGKSFTGPVNSSQLMVETDW 171
whale_shark_WA_DS5_like              66 RGFQARLRGTTREMIHLTNF------TAITKKKPSVHIYMEQNGGPGHSDILYCYAEKFYPFGIELNFLVNGRPFRGQVNSSQLVVEPDW 149
zebra_bullhead_shark_WA_DS5_like     88 RGFQARLQIITEEMIKLTNA------KRIISKKPAVHIYTKENYTPHQSNTLYCYAEKFYPFEIDITFLINGRRFTGLVNSSQTVVEQDW 171
spiny_dogfish_WA_DS5_like            67 SGIQAWQQGITKEMTKLTNG------TSVLKRKPSVHIYTQESYTPRPSNTLYCYAEKFYPFEIDINFLINGRRFTGPVHSSQLVVEADW 150
little_skate_WA_DS5_like             88 EGFQNQIRSDMSELAAGNDN------QPIGSKKPIAHIYTEEDYVLQRANTLYCFAERFYPFEIELQFLVNGQPFTGPVHSSPLLVERDW 171

                                                                          CP/TM/CY
banded_houndshark_WA_DS5_n1_01      172 TFNILKYIRIDPQDGDSYSCQVDHISLDKPLTVSMDPPSPGPRSGIIVCAVGVITGVIGLLIGLYLVTTVCSRLGKPRSRQFRKE       256
banded_houndshark_WA_DS5_n2_01      172 TFNILKYIRIDPQDGDSYSCQVDHISLDKPLTVSMDPPSPGPRSGIIVCAVGVITGVIGLLIGLYLVTTVCSRLGKPRSRQFRKE       256
banded_houndshark_WA_DS5_n3_01      172 TFNILKYIRIDPQDGDSYSCQVDHISLDKPLTVSMDPPSPGPRSGIIVCAVGVITGVIGLLIGLYLVTTVCSRLGKPRSRQFRKE       256
banded_houndshark_WA_DS5_n4_01      172 TFNILKYIRIDPQDGDSYSCQVDHISLDKPLTVSMDPPSPGPRSGIIVCAVGVITGVIGLLIGLYLVTTVCSRLGKPRSRQFRKE       256
cloudy_catshark_WA_DS5_like_82K     180 TFNILKYISIDPQDRDTYSCQVNHMSLDEPMTVLMDQSYSQPHTGTIVCAVGVIAGALGLMVGLYLVTKLCSRQGKPCSRQFCK-       263
cloudy_catshark_WA_DS5_like_159K    177 TFHVLKYIRIDPQEGDTYSCQVDHVSLDKPMFVSM-----------------------------------------------------       211
great_white_shark_WA_DS5_like       172 TFNILKYIRIDPQDGDTYSCQVDHMSLDKPLTVLLDQPPAQPHSGTIVCAVGVIAGVLGLLIGLYLVTKIHNRQGKPCSRPFCKE       256
whale_shark_WA_DS5_like             150 TFNILKYIRMERGSGDTYTCRVDHISLQQPLTVSL-----------------------------------------------------       184
zebra_bullhead_shark_WA_DS5_like    172 TFNILKYIRIDPHDGDTYSCEVAHMSLEKPLTVLLDQPPALPYTGTIVCAVGVIAGILGLVITLYLVTKIYKRRGKLCSDQLCKQ       256
spiny_dogfish_WA_DS5_like           151 TFNILKYIRIDPQDGDTYSCQAAHISLDKPLTVLLDQPPLMPYSGTIVCAVGVVAGVIGLAITLYLMTKIYSRRGKFCTGQFCKQ       235
little_skate_WA_DS5_like            172 TFNILKYIRIEPQRGDTFSCLVAHVSLDKALTLSL-----------------------------------------------------       206
```

B Shark/skate WA_DS10

```
                                  Leader              α1
banded_houndshark_WA_DS10_01        1 --MSRESFLLLLLLSPGTGLSATDTWRVLVACYITDDPNPLEMMCEVTADSRIFIYYDTS---IHLLEGGLEEYRGVLVELA-RDRNRAI 84
blue_shark_WA_DS10_like             1 MKSHPGNSLLILLLLTAGTGLPATDTWRVVVTCYVTDNPSPTEMMCELTADGRVLIYYDTS---IHLLEGNLEEYHGLLDELA-RDRNRTI 86
cloudy_catshark_WA_DS10_like        1 MSRDSFLFFLLLCPGTGS--SESDPWRVLVACFVTDNPSLQPLMCEVTVDDRMFIYYDSTLPSVQVLDSGLEEYRWILAKLV-SDRERTI 87
great_white_shark_WA_DS10_like      1 MSRDFFLFFLLLCPCPGTGNSGLDRWKVSVACFVTDNPSLLEMACELTTNDRILLYYDSTTPSVQFLVSGFEDYRGIFEELV-QDRDRAL 89
whale_shark_WA_DS10_like            1 ------------------------RWNMQFGCFAADDPRFPELVCELQVEGRPFIYYDSSLPDIQFLAPGFEDYRAILTEFT-QDRDRTV 65
spiny_dogfish_WA_DS10_like          0 ------------------------------------------------------------------------------------------ 0
little_skate_WA_DS10_like           1 ------------------------KTTLALTFFATDDPSLPDGVLEAKADGRSLGYYDTTLSKAQFHLSNLESLQPLVEELIERNIDSTL 66

                                                               α2
banded_houndshark_WA_DS10_01       85 ASLRQTMALAAIATNFSSPPNEMADLLLFPEKEVTYGEANILSCLVSGAFPPTITITLQVNGAPLHTDANSSRLWYGEDWRFQAVRRAHI 174
blue_shark_WA_DS10_like            87 ASLRQTMALAAVATKFSSPPNEIADLLLFPEKEVTYGKANILSCLVSGVFPPTITITLQVNGVPLHTDANSSRLWHGEDWRFQVVRRAHI 176
cloudy_catshark_WA_DS10_like       88 ASLRREMAFIATISN-SSPPNDFGELFLYPESEVTYGEMNVLTCLVNGAFPPTITVTLQMNGVPIDAGVNSSRLSFGEDWRFRVTRHAQI 176
great_white_shark_WA_DS10_like     90 ISLRRTMALTAAVTNHSSPPNEIAQLFLYPEKDVVYGQESILICLVSGLFPPTIDITLQKNGAPLDAHVNSSKLSFGEDWRFQVLHYAQV 179
whale_shark_WA_DS10_like           66 ASLRRALAIAAVVTNHSNSPNEVGEIFLYPERLVEWGQPNVLVCLVTGLFPPSVAVSLHLDGAPLGSDVNSSRIFFGEGWRFQALWYAHI 155
spiny_dogfish_WA_DS10_like          1 ------MHDMIALTSNSQPPNEIGEVFLYSEKPVVFGQTNVLTCWVTGLFPPAVNATLYRNGEALASEVNSSRLSFGEDWRFQTLRYAEI 84
little_skate_WA_DS10_like          67 GLVHSLLTQVMALTNNSLPSNEIGEALLYSEKPDVLGEENTLICFVSGFFPPAIEVALQKNGKPIVGEVNSSHLSFSEDWRFYTLQYTHI 156

                                                                   CP/TM/CY
banded_houndshark_WA_DS10_01      175 RPEAGDMYSCQVQHTVSKEVKMVYWEPEMPDSWEESILDSSQLGVLVGGVAVGALGMALGIGSCVWPKALSRNQSLLRIRSSLRSSSSV  263
blue_shark_WA_DS10_like           177 QPEAGDMYSCQVLHTVSKEMKMVYWEPEMPDSWEENILDSSQLGVLVGGVALGVLGMALGIGSCVWPKALNQNLSLLQMRSSLRSTSSA  265
cloudy_catshark_WA_DS10_like      177 RPAAGDLYSCEVLHTISNEVKVVYW-----------------------------------------------------------------  201
great_white_shark_WA_DS10_like    180 RPEAGDTYSCKALHTISREEKIVYWEPELPNPSEESALDSSQFAVLVLGLIVGLLGTATGICFCLWPILVNRNRLRSRMNSSVRSSSSA  268
whale_shark_WA_DS10_like          156 RPGPGHLYSCVVRHNISREEKVVFW-----------------------------------------------------------------  180
spiny_dogfish_WA_DS10_like         85 QPAAGDIYSCKVVHGTGKEEMVTYWELDAQ------------------------------------------------------------  114
little_skate_WA_DS10_like         157 QPTEGDIYSCKVFHNISKEERVTYWEPEVQEG-KEAELDMSQLIVLICGVIIGILGAAMGFSLCFWLRFTRQDSWNIRINPFVGVSHSS  244
```

C Shark WA_Nds3L

```
                              Leader              α1
blue_shark_WA_Nds3L_01        1 MDTERKKNRIAFLRTLITAWVLLPATECGESRVYGTTYSTSAEQTSVFTFATSDMTFAYYNSSSPGFEFLIKALYTLSNASQHLVQKYRN 90
great_white_shark_WA_Nds3L    1 MGCPRQNNKVVFFCALPLAWLLGSAIECAEQRVRGMIFSVSDSRTSIVTSTTDDMTLAYHNSTESNVVFLIEALNALHAESNNLIQKYQS 90

                                                α2
blue_shark_WA_Nds3L_01       91 DLTILNKDILNEMVHRIPGKRTEVPTVLLYPTEPNILGRRTVLNCFVFELFPPAAQVTFLKNDQPLVTKVNSSLLTFQTSWMFHVLNSVP 180
great_white_shark_WA_Nds3L   91 YLT-FNEILLNEMANRIPEKQNDDPTVLLYPGEPFTLGGKNTLNCFVSHLFPPVAHVSFLKNDQPLSGQVRSSQFTFDNSWMFQILKYVQ 179

                                                       CP/TM/CY
blue_shark_WA_Nds3L_01      181 IQPVPGDIYTCVVKLQNGEEIQVHWEAATSDTQRDAAQIAICTIGFLIGAVGMMIGLWLIFYKRGNTQVDSRDQRTVIVEERQ------- 263
great_white_shark_WA_Nds3L  180 IEPAVGDKYSCVVELVTKEQFRAYWEATTSGNHKNPVQIAICAVGFVIGGLGMMTGLCLIFYKWGNE-VGSRDQQTIPAEEPQDSMLNGE 268

blue_shark_WA_Nds3L_01      263 --------------------                                                                       263
great_white_shark_WA_Nds3L  269 GAQDEGTRVDRSDPNQPAEC                                                                       288
```

Fig. S1

D Teleost fish WA

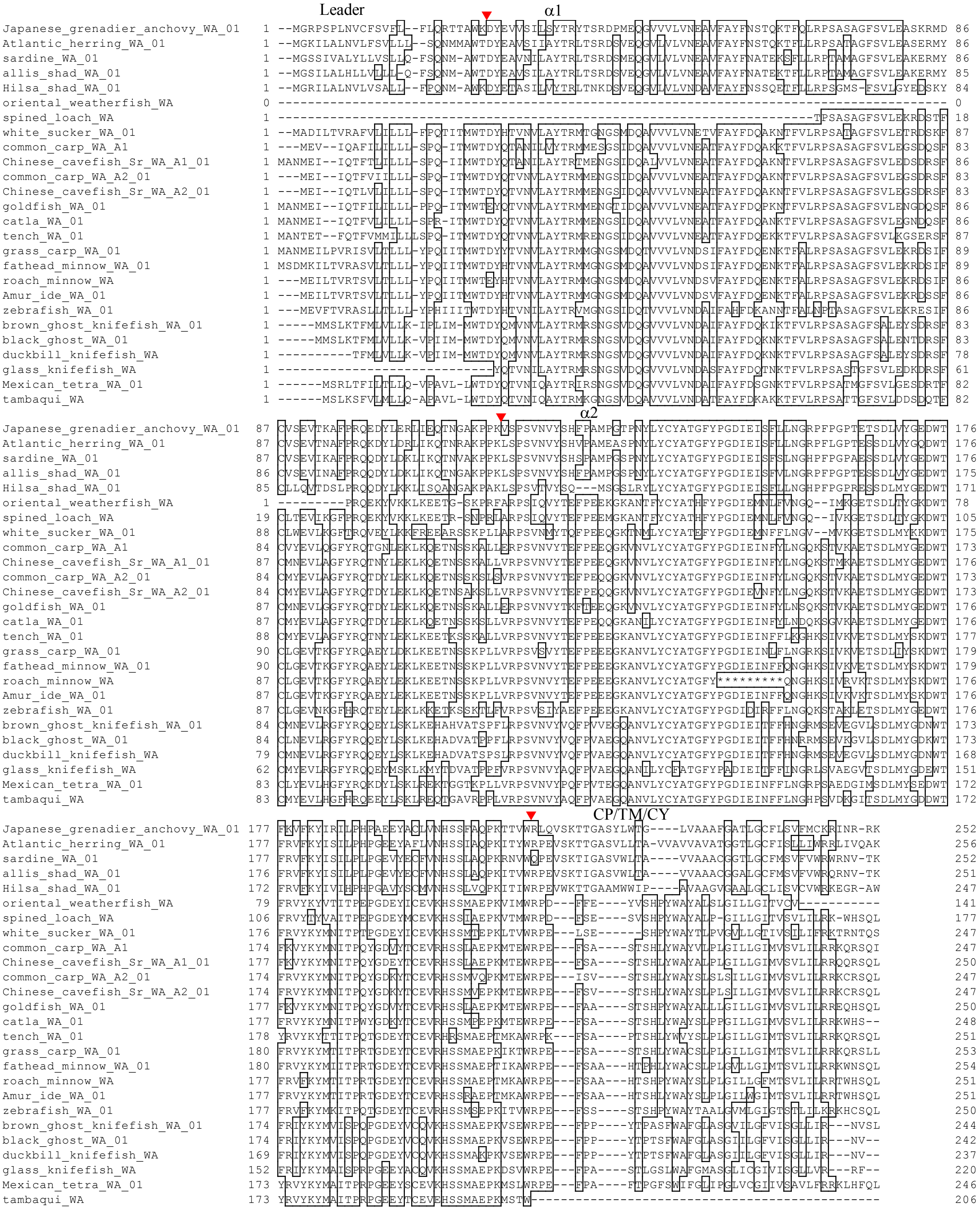

Fig. S1

E Lobe-finned fish WA_1 and WA_2

Leader α1

```
African coelacanth_WA_1_01           1 -MELLRTWPFWGFLWGPLLVTGVEE--DGDFFGMHVLTADMDSVSFGVAVVVSYNDESIAHWDQEKEDTVMEIPGIQHH---MTELFNLL 84
South_American_lungfish_WA_1_01      1 MCGKILNSLSAIILWSSALLLLAEK--DTFFFDAYLFSSDNDVSKNNFQLSVSVNGLELMQWDVTNQTFLEQIGGMEEV---MPFLNSVV 85
Australian_lungfish_WA_1             1 ------MRSALCVVWSLVLMSVAEE--DGYVFDVYFITADNNSNAPVIHSSVAVNGIEIIRWDEKKQKVLQQIGGLEEL---MPFVNNLL 79
West_African_lungfish_WA_2_01        1 ---MATFQGFGLLLWLLFWINMADATFRFRKLEVLPFTTLDMKTIQMQSLAILVNDVPIGLFDQHENKFLIRLNISEEACNKMKQLAVLY 87
```

α2

```
African coelacanth_WA_1_01          85 INNRERAYHFHEEV-KTFVYP-HVPKPPNVMPDIYLYMENPITFGKKNRLNCYAMNFFPPNLRAVLLENENPLLVKKVAIWDLTFNLGGN 172
South_American_lungfish_WA_1_01     86 RLNIKGTMALHQHIFQVMSEETHSAPAPNELPDIYSYFEKPIKFGEKNNLSCFAVRFYPPSIQINILKNNVVIPSEIKP-SGAMFDINWK 174
Australian_lungfish_WA_1            80 YGNIRGALKEHNEVVKLMTEATHNSVPPNELPDIYLYFEKPIKFGEKNNLSCFIIRFFPLAVQIEILRNNISIPGEIKS-FRLMFDDDWK 168
West_African_lungfish_WA_2_01       88 MREAKGNMDWQKVLIR-MMETKQKVHPPLEPPSVSMYSEKPYVPGQNNTIYCYATGFYPPEIDIVFFENGNHFAGKVYA-SDIAFSEDWR 175
```

CP/TM/CY

```
African coelacanth_WA_1_01         173 LQLLLSVEVVPKLRDSYSCTMEHLKLRQNRTIVLDAQEPYKPAADHSETAALAIGIIVGIIGMLVGIVLIAKH-------        245
South_American_lungfish_WA_1_01    175 FQMLKSAEVTPMKGDIYSCSVEHLILKQTRNVTIEPKEPYIPQVDHSETIVCAAGTIVGITGLLLGIFLIRKQHTNRDVM        254
Australian_lungfish_WA_1           169 FQILKSMEVVPRAGDIYSCSVEHLILRQKKLVTLGKLIWAYNYV-------------------------------------        212
West_African_lungfish_WA_2_01      176 FKIMKFIHVVPQQTTLYSCKVNHISFKQPRELTLGNSMDHHARV----PVLFIVSVPCALIGIPLGIYFLKDSRLSSFR-        250
```

F Salamander WA

Leader α1

```
Chinese_salamander_WA         1 MNSLRTIAWMCLLAANSCQQGIKDLFLEAFTSTGSPGASNLTVALLADTVVAAYYSGTNGTFQFPPSQLEALGRFAANQIITNQTVAFHQ 90
Hokkaido_salamander_WA_01     1 MNSLRTIAWICLLAANSCQQGIKDLFLEAFTSTGSPGASNLTVALLADTVVAAYYSGTNGTFQFPPSQLEALGRLAANQIITNKTVAFHQ 90
tiger_salamander_WA_01        1 MNSLPASLCMCILTVVRCQGGSKNIQLAAFTSTDSPAASNLTLALLADTVVAAYYDGSTDTFQIPDTGLKDTVQTFAHFFPMESVSNFHQ 90
tiger_salamander_WA_02        1 MNSLPAYLCMCILTVVRCQEGSKNIQLAAFTSTDSPAASNLTLDLLADTVVAAYYDGRTNTFQIPDTGLKDTVQTFAHFFPMERVSNFHQ 90
axolotl_WA                    1 MNRLPASLCMCILTVVRCQEGSKNIQLAAFTSTDSPAASNLALALLADTVVAAYYDGSTDTFQIPDTGLKDTVQTFANFFPMERVSNFHQ 90
```

α2

```
Chinese_salamander_WA        91 HSMDTLCQRSNCSDPVFVFPDVQFYPEIPVVLGQENRLLCFLKEFFPPEVSVAFLKNGKPFRGQIQSSELTFGRNWTFQVLKSITVEPGA 180
Hokkaido_salamander_WA_01    91 HSMETLCQRSNCSDPVFVFPDVQFYPEIPVVLGQENRLLCFLKEFFPPEVSVAFLKNGKPFMGQIQSSELTFGRNWTFQVLKSITVEPGA 180
tiger_salamander_WA_01       91 MVMKDMCMKINCSDPVSVFPEVQFYIEAPVVLGQENRLLCFLKGFFPPEVRVSFLKNEQPFPGQMQSSELIFGRNWTFQVLKYILVKPQA 180
tiger_salamander_WA_02       91 MVMKDMCMKINCSDPVSVFPEVQFYIEAPVVLGQENRLLCFLKGFFPPEARVSFLKNGQPFPGQMQSSELIFGRNWTFQVLKYILVKPQD 180
axolotl_WA                   91 MVMKDMCMKINCSDPVSVFPEVQFYIEAPVVLGQENRLLCFLKGFFPPEVRVSFLKNEQPFPGQMQSSELIFGRNWTFQVLKYILVKPQA 180
```

CP/TM/CY

```
Chinese_salamander_WA       181 GDTYSCTVEHGNRQSHRDLDFEKLPMTVENNAHIVVLAVGLAVGILGFAMGLLLFLQYSTRNPYMQWLNRNSVSS       255
Hokkaido_salamander_WA_01   181 GDTYSCTVEHGNRQSRRDLACGKLPMTVENNAHIVVLAVGLAVGILGFAMGLLLFLQYSTRNPYMQWLNRNSVSS       255
tiger_salamander_WA_01      181 EDTYSCIVEHGYLQSRQNLTWGR-PVSLENKTHVTVLIVGLTVGFLGFVVGLILCIHGKNLKCLARNPYQR----       250
tiger_salamander_WA_02      181 EDTYSCIVEHGYLQSRQNLTWGR-PVSLENKTHVTVLIVGLTVGFLGFVVGLILCIHGKNLKCLASNPYQR----       250
axolotl_WA                  181 EDTYSCIVEHGYLQSRQNLTWGR-PVSLENKTHITVLIVGLTVGFLGFVVGLILCIHGKNLKCLASNRYQR----       250
```

Fig. S1. Representative W-category α chain (WA) sequences from cartilaginous fish, teleost fish, lobe-finned fish and salamanders.

A, banded houndshark WA_DS5 and closely-related sequences of other cartilaginous fish. From a single individual banded houndshark, four kinds of WA_DS5 cDNA sequences were obtained. Currently, the allelic relationship of these sequences is not clear. **B**, banded houndshark WA_DS10 and closely-related sequences of other cartilaginous fish. **C**, blue shark and great white shark WA_Nds3L sequences. **D**, WA sequences of teleost fish. Information of the oriental weatherfish WA sequence is currently incomplete. The additional twenty-two residues in the last part of sardine WA_01 and the additional two residues in the last part of Hilsa shad WA_01 are not included. **E**, WA sequences of lobe-finned fish including African coelacanth and three kinds of lungfish. **F**, WA sequences of salamanders. The positions where the two tiger salamander WA sequences (obtained from a single individual and presumable alleles) show disparity are shown by black circles above the sequences.

The borders of the corresponding exons are shown by red arrowheads. Additional borders within the CP/TM/CY region are shown by open arrowheads. All sequences used in this figure are listed in Table S3 and composite or corrected sequences are described in *SI Appendix*.

Fig. S2

A Shark/skate WB_DS1

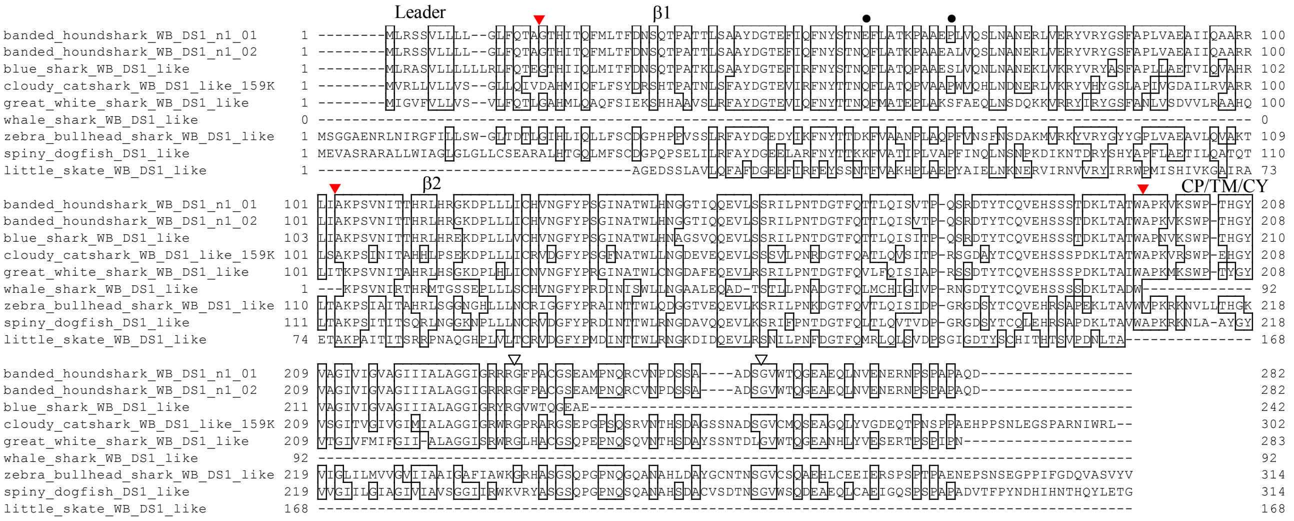

B Shark/skate WB_DS3

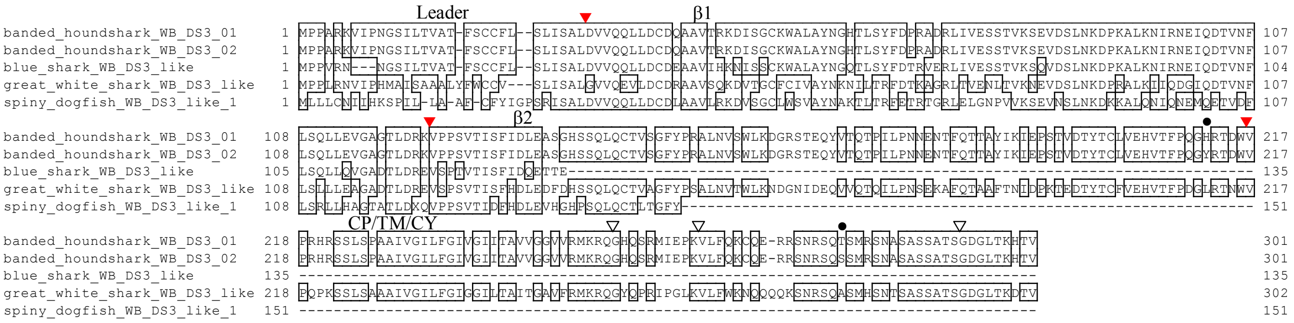

C Shark WB_Nds5L

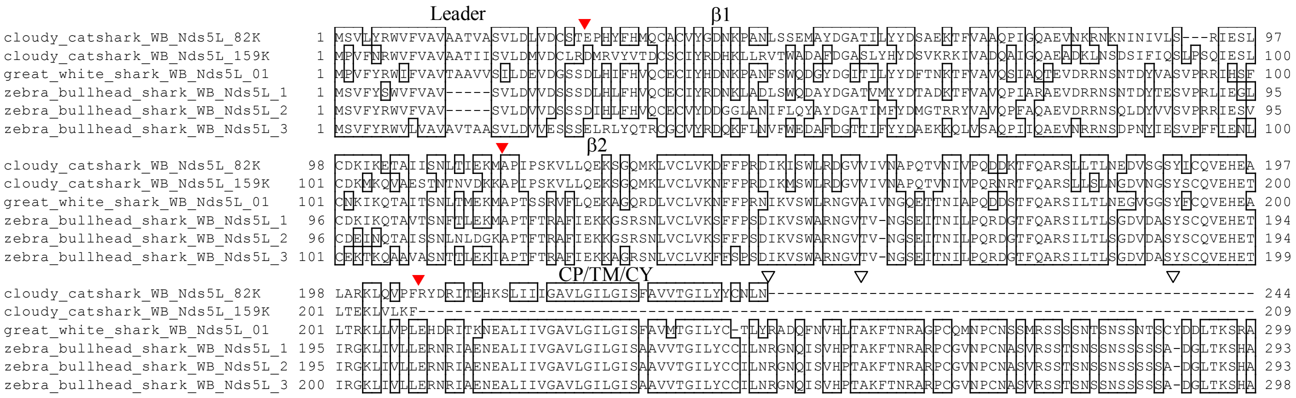

Fig. S2

D Teleost fish WB

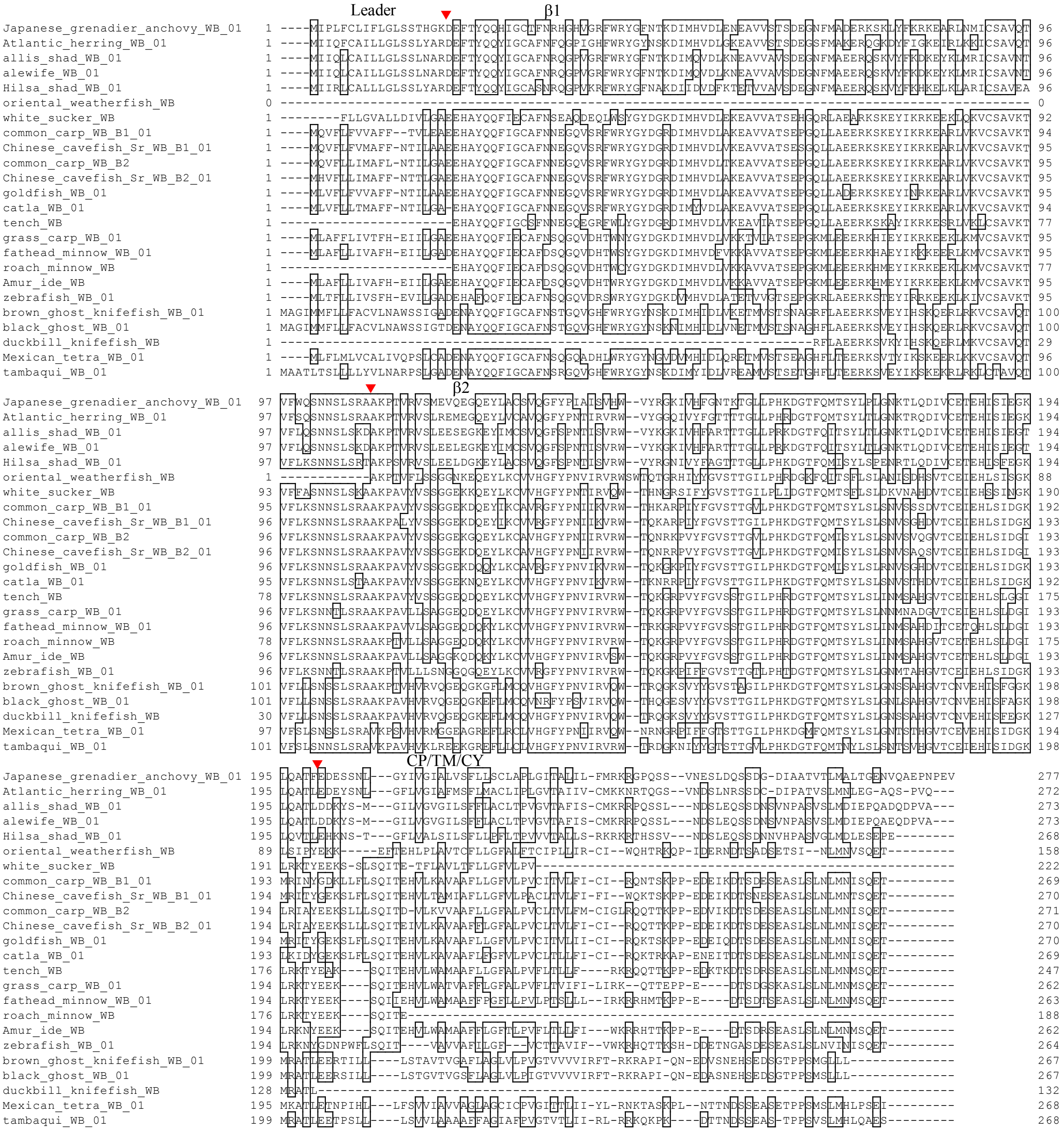

Fig. S2

E Lobe-finned fish WB_1 and WB_2

```
                                       Leader                 β1
African_coelacanth_WB_1_01        1 --MPLAGKGLVLWVVLLSVLFFSQLPNVQGVFNYLQHQFFCAEDSSTNASFCRWLVSYDSRKLFYLDSRVNRFVPLQQFLEKKMEEKNSN 88
South_American_lungfish_WB_1_01   1 ---MFTNRYACQWQKFYLSFLIILIFAFQCELHFLHHHVHCTSFNTGNKTL-SWEVAYDSLTIAHVDNENVGIAATSPYVHEEIESFNRR 86
West_African_lungfish_WB_2_1      1 MLCSCCGIALGLVLFSLLQNNACGGDHVREAMNTLQFRMGCSMD--QGKMKYFWQRGYNQKNIKRYDYTEQRAIMEHECVKDEVEADNRN 88
Australian_lungfish_WB_2_2        1 --MILHKLVLSFLCILLHRKDACG-----EATSTFQYRAGCVLE--KGRNTYFWRSSYNNKDIEHYDYERQCLVADYPSAKDRAEEYNKS 81

                                                                          β2
African_coelacanth_WB_1_01       89 KLTFQQYNSICQLACEYKEKVLASAKATLNRKVPPMVNIGIMKDEEIQTDTLSLICSIRGFYPQAISTTWLRNGEDIKSETFTTGLLPNV 178
South_American_lungfish_WB_1_01  87 KDLIKRYNNIMHDVWPFLDLIASLGNSTLDRKVAPSVTIVVDPV---DHGILNLACYIHGFYPQNIEVFWLQNGEDITEKMATTDILPNT 173
West_African_lungfish_WB_2_1     89 PILRKSRQETAEKFCQKARSIFDELPVHLGELGNNSVSISLENQ----GKENYLVCFVRGFHPSNIKVTWLKDGKEELYGVTTTGVLPLR 174
Australian_lungfish_WB_2_2       82 PSFPMYWNNLITDLCSSAKKLFDELPAELGRLERSSVSVSLETK----EKDKYLECLVHGFHPSYINVTWLRDGKEQFYGVTSSGILPLK 167

                                                                          CP/TM/CY
African_coelacanth_WB_1_01      179 DKTFSLDLYVDIVESDWNEYTCQVEHSGLTEPLHLTLDRKQPGKS--GIIIGAVFAAVGVIVMVTGLI--VRIRRNGSDAAETISINSIS 264
South_American_lungfish_WB_1_01 174 DNTFSLITYVDAEENNKHKYTCMVKHSSVTNGISATVDFNQGFGI--GYYVGGVLALVGIIAGMTGII--LRYRLKALRVCMQRRNLGPA 259
West_African_lungfish_WB_2_1    175 DGTFVMKMYLFLADTTKGLYICQVEHESLKGKIRVLWEQKSEVQSFPDSIIWITFILALLLILLLACI--SKIQKDHQTATTKIPEDRKE 262
Australian_lungfish_WB_2_2      168 DGTFHIRKYLPIGDSVGGSYACQVEHESLVEAILVHWDPVRKT-SFLEKILGIPVLIIGVCFPIFGYIYKRKFPKRGRKETSSISTSSLE 256

African_coelacanth_WB_1_01      265 EMVPMS---------------------------------------------------------                            270
South_American_lungfish_WB_1_01 260 ETVHRTQPTRQELEQLDGSPDSLHENVFSSSNYEAAYLTIQDIPVIGLTSDIPLDQSTSTTVE                            322
West_African_lungfish_WB_2_1    263 MDKLCSSSMGVGNEEE-----------------------------------------------                            278
Australian_lungfish_WB_2_2      257 GNPQAAPLNPEENTD------------------------------------------------                            271
```

F Salamander WB

```
                              Leader                   β1
Chinese_salamander_WB       1 MFSHTWIPLLHIISSISTADAHIFQQTLGCSTRSASFPEITECWWRAAYNGEEVWKFDLLNSTSVYFSPLMVEDERLFLHHFRNSILPSGVDIVASLQSF 100
Hokkaido_salamander_WB_01   1 MFSLTWIPLLHIISSRSTADAHIFQQTLGCSTRSASFPEITECWWRAAYNGEEVWKFDLLNSTSVYFSPLMVEDERLLLRHFRNSILPPGVDIVASLQSF 100
tiger_salamander_WB_01      1 MPPLAWITILLALCCQFTAGSQIFQQLIECS-KSPSSPATPQCWWRAAYNREQLWDFNLLNSNIDLLLLDLNRRFGLPLEYLLRELQYPLQNTPFAANVF 99
tiger_salamander_WB_02      1 MPPLAWITILLALSCPFTAGSQIFQQLIECS-KSPSSPATPQCWWRAAYNREQLWDFNLLNSNIDLLLLDLNRRFGLPLVYLLRELEYPLQNTPFAANVF 99
axolotl_WB_01               1 MPPLAWITILLALSCPFTAGSQIFQQLIECS-KSPSSPATPQCWWRAAYNREQLWDFNLLNSNIDLLLLDLNRRFGLPLEYLLRELQYPLQNTPFAANVF 99

                                                  β2
Chinese_salamander_WB     101 MNQPARETVTPNVTVSLADTEVGAPLKSLCCRVSEFYPPDLNVTWSLDGSTLALGNGLKEPVILPNSDGTFQTTSCIPFNSSHPQGQHYSCTVQHLSTLL 200
Hokkaido_salamander_WB_01 101 MNQPARETVTPNVTVSLVNTEVGAPQKSLCCRVSEFYPPDLNVTWSLDGSTLALGNGLKEPVILPNSDGTFQTTSCIPFNSSHPQGKRYSCTVQHLSTPL 200
tiger_salamander_WB_01    100 MSLTAKEIVAPRVTINAEETEVGAPLYTLCCRATGFYPPNINVTWFLDGSPLAHDHGMKELVILPNSNGTFQTTSCMPFSISHTQAKNYLCAVQHISTPE 199
tiger_salamander_WB_02    100 MSLTAKEIVAPHVTINAEETEVGAPLYTLCCRATGFYPPNINVTWFMDGSPLAHDHGMKELVILPNSNGTFQTTSCMPFSISHTQAKNYLCAVQHISTPE 199
axolotl_WB_01             100 MSLTAKEIVAPHVTINAEETEVGAPLYTLCCRATGFYPPNINVTWFLDGSPLAHDHGMKELVILPNSNGTFQTTSYMPFSISHTQAKNYLCAVQHISTPE 199

                                                CP/TM/CY
Chinese_salamander_WB     201 GLVAKWEAPPLDNRMVKAEMVIGILAGLVGIVFLSSALIYHGCIREGRISSCCKSEDRGMEISEIDTVSEG-----                          271
Hokkaido_salamander_WB_01 201 GLVAKWELPPSENRMVKAEMVIGILAGLAGIIFLTSALIYHGCIRKGRISSCCKSEDRGMEISEIDTASEGASASA                          276
tiger_salamander_WB_01    200 GLNATWEAPGLEEH--KAELAIGILAGLAGVFFASSALVWHGCCKKGRITSCWKHEGLMVEISEMAAASEAASPSV                          273
tiger_salamander_WB_02    200 GLKATWEAPGLEEH--KAELAIGILAGLAGIFFSSSALVWHGCCKKGRVTSCWKHEGLMVEISEMAAASEAASPSV                          273
axolotl_WB_01             200 GLNATWEAPGLEEH--KAELAIGILAGLAGIFFASSALVWHGCCKKGRITSCWKHEGLMVEISEMAAASEAASPSV                          273
```

Fig. S2. Representative W-category β chain (WB) sequences from cartilaginous fish, teleost fish, lobe-finned fish and salamanders.
A, banded houndshark WB_DS1 and closely-related sequences of other cartilaginous fish. The positions where the two banded houndshark WB_DS1 sequences (presumable alleles) show disparity are shown by filled circles above the sequences. The additional twenty-two residues in the last part of zebra bullhead shark WB_DS1-like are not included. **B**, banded houndshark WB_DS3 and closely-related sequences of other cartilaginous fish. The positions where the two banded houndshark WB_DS3 sequences (found in different individuals) show disparity are shown by filled circles above the sequences. **C**, cloudy catshark, great white shark and zebra bullhead shark WB_Nds5L sequences. **D**, WB sequences of teleost fish. Information of the oriental weatherfish and white sucker WB sequences is currently incomplete. **E**, WB sequences of lobe-finned fish including African coelacanth and three kinds of lungfish. **F**, WB sequences of salamanders. The positions where the two tiger salamander WB sequences (obtained from a single individual and presumable alleles) show disparity are shown by black circles above the sequences. Information on the last residues of the Chinese salamander WB sequence is currently incomplete.

The borders of the corresponding exons are shown by red arrowheads. Additional borders within the CP/TM/CY region are shown by open arrowheads. All sequences used in this figure are listed in Table S3 and composite sequences are described in *SI Appendix*.

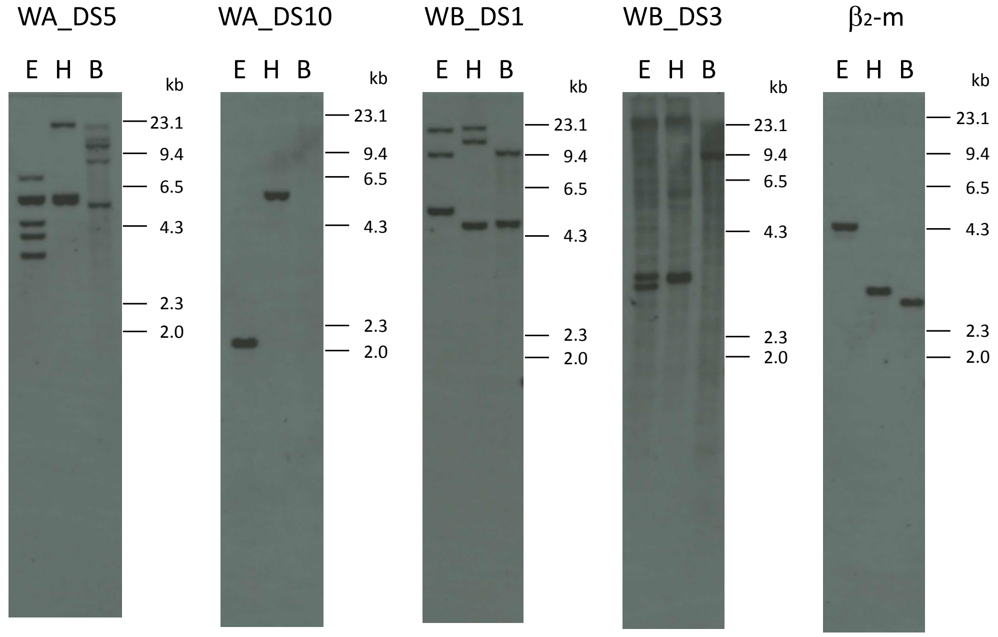

Fig. S3. Southern blot analyses for the banded houndshark *W-category* and *β_2-m* genes. Banded houndshark genomic DNA was digested with either *Eco*RI (E), *Hin*dIII (H) or *Bam*HI (B) as described in the Materials and Methods. The primer sequences for the probes in the analyses are listed in Table S2. Clearly-positive bands could not be observed in *Bam*HI-digests probed with *WA_DS10* in several independent experiments. Background bands in the blot of *WB_DS3* were not observed in other independent experiments (e. g., Fig. S10). Agarose gels of 1.2 % were used.

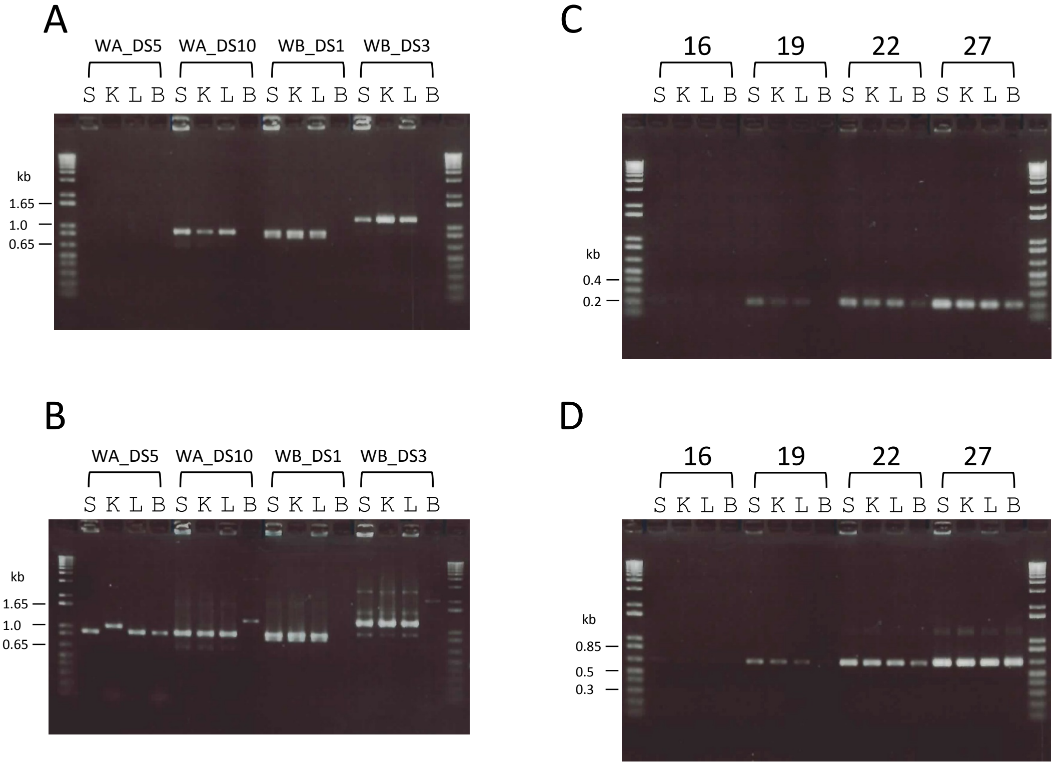

Fig. S4. Expression of various WA and WB genes of a banded houndshark individual (N1).
A, Amplification of various W-category genes with 32 cycles of RT-PCR. S, K, L and B are abbreviations of spleen, kidney, liver and blood, respectively. Using RNA of spleen, kidney and liver, cDNA fragments of expected lengths were amplified in case of WA_DS10, WB_DS1 and WB_DS3. The primers used are described in Table S2. The primers for *WA_DS5* used in this experiment did not discriminate among *WA_DS5_n1~n4*. **B**, Amplification of various W-category genes with 42 cycles of RT-PCR. Compared to the results of 32 cycles in A, some irrelevant bands became apparent and the amount of amplified cDNA of *WA_DS5* increased. In the amplified products of *WA_DS5* using the kidney sample (K), a fragment with a length longer than expected was noticed. Sequencing of this fragment revealed it to be an alternatively spliced product which includes an extra region found in the intron 1 of *WA_DS5* gene and produces a frame-shift in the open reading frame. In a separate experiment, using a different RT-PCR protocol (protocol No. 2) described in the Materials and Methods, we could amplify cDNA fragments of *WA_DS5* with lengths expected from canonical type sequences using the same kidney sample. cDNA fragments with lengths longer than expected in case of WA_DS10 with blood sample (B) have not been investigated yet. **C**, RT-PCR amplification of β-actin cDNA (a short fragment) with various cycles (16, 19, 22 and 27 cycles as indicated on top). **D**, RT-PCR amplification of β-actin cDNA (a longer fragment) with various cycles (16, 19, 22 and 27 cycles as indicated on top). With more PCR cycles, the number of irrelevant bands increased. The primers used for C and D are listed in Table S2. Agarose gels of 1.5 % were used for all experiments in this figure.

Fig. S5

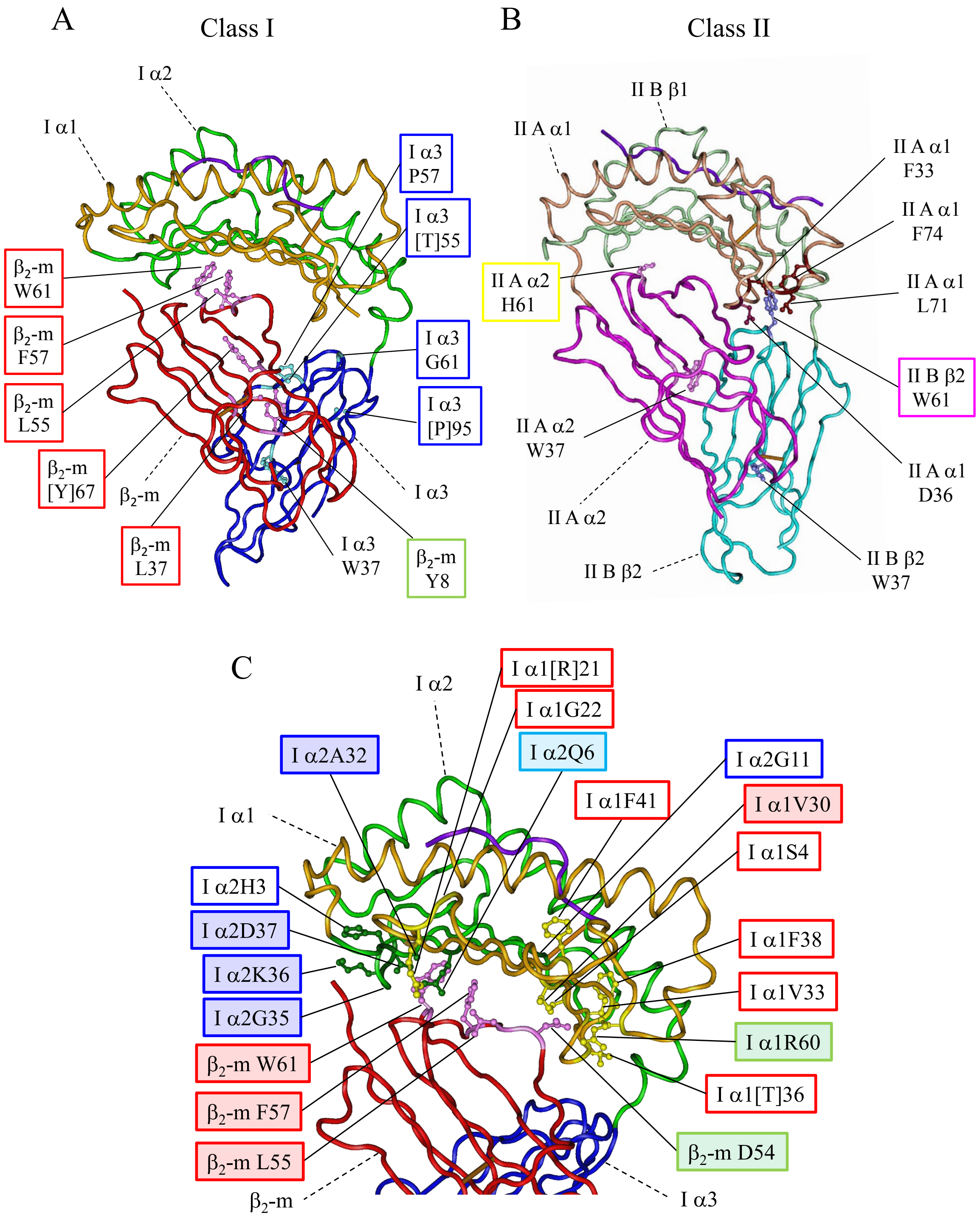
A
Class I
I α2
I α1
I α3 P57
I α3 [T]55
β_2-m W61
β_2-m F57
β_2-m L55
I α3 G61
I α3 [P]95
β_2-m [Y]67
β_2-m
I α3
β_2-m L37
I α3 W37
β_2-m Y8
B
Class II
II B β1
II A α1
II A α1 F33
II A α1 F74
II A α2 H61
II A α1 L71
II B β2 W61
II A α2 W37
II A α1 D36
II A α2
II B β2
II B β2 W37
C
I α2
I α1[R]21
I α1G22
I α2A32
I α2Q6
I α2G11
I α1F41
I α1V30
I α1
I α1S4
I α2H3
I α2D37
I α1F38
I α2K36
I α1V33
I α2G35
I α1R60
β_2-m W61
I α1[T]36
β_2-m F57
β_2-m D54
β_2-m L55
β_2-m
I α3

Fig. S5. The structural positions of amino acid residues which are shared between W-category and class I molecules.

In A, an overall structure of an MHC class I molecule, HLA-A2, is shown. The positions of amino acid residues often shared between W-category and class I molecules in the Ig-like domains are indicated. In B, an overall structure of an MHC class II molecule, HLA-DR1, is shown for comparison. In C, the interface between β_2-m and the membrane-distal domains of HLA-A2 is shown highlighting positions of several residues shared between W-category and class I molecules.

A, Five amino acid positions of β_2-m (with red frames) and four positions of the class I α3 domain (with blue frames) highlighted in Fig. 3 with red and blue shadings, respectively, are indicated. The residues in brackets are different from those shared between W-category and other class I molecules. In the figure, the position of I α3W37, which corresponds to the highly conserved tryptophan in the Ig superfamily and is indicated with an open square in Fig. 3, is shown. The position of β_2-m Y8 (with a light green frame), which interacts with I α3P57 (Fig. S6), is also shown.

B, Two amino acid positions, H61 of the class IIA α2 domain (IIA α2H61, with a yellow frame) and IIB β2W61 (with a magenta frame), are indicated. These residues possess the same position 61 in the sequence alignment (Fig. 3) and correspond to β_2-m W61 and I α3G61 (shown in A), respectively. IIA α2H61 is not conserved among classical MHC class II molecules. Highly conserved IIB β2W61 forms a hydrogen bond with the main chain carbonyl oxygen of IIA α1E37 and further interacts with the three conserved hydrophobic amino acid residues of the class II α1 domain (IIA α1F33, IIA α1L71 and IIA α1F74) (*31*) as shown. In the figure, the positions of IIA α2W37 and IIB β2W37, which correspond to the highly conserved tryptophan in the Ig superfamily and is indicated with an open square in Fig. 3, are also shown.

C, The positions of amino acid residues frequently shared between W-category and class I molecules but not shared with the classical class II molecule at the interface between the membrane-distal and the membrane-proximal domains of HLA-A2. These comprise eight positions of the class I α1 domain (with red frames) and six positions of the class I α2 domain (with blue frames). I α2Q6 (with light blue frame), I α1R60 and β_2-m D54 (both with light green frames) are also indicated, although these can be observed in the corresponding positions of some classical class II molecules (Datasets S1 and S2). A glutamine corresponding to I α2Q6 is highly conserved in WB β1 domains, and an arginine corresponding to I α1R60 plus an aspartic acid corresponding to β_2-m D54 can be found in teleost fish and lungfish WA. β_2-m L55, β_2-m F57 and β_2-m W61, three residues of β_2-m at the interface shared between W-category and class I molecules are also indicated.

Additional explanations for this figure are described in the section 11 of *SI Appendix*.

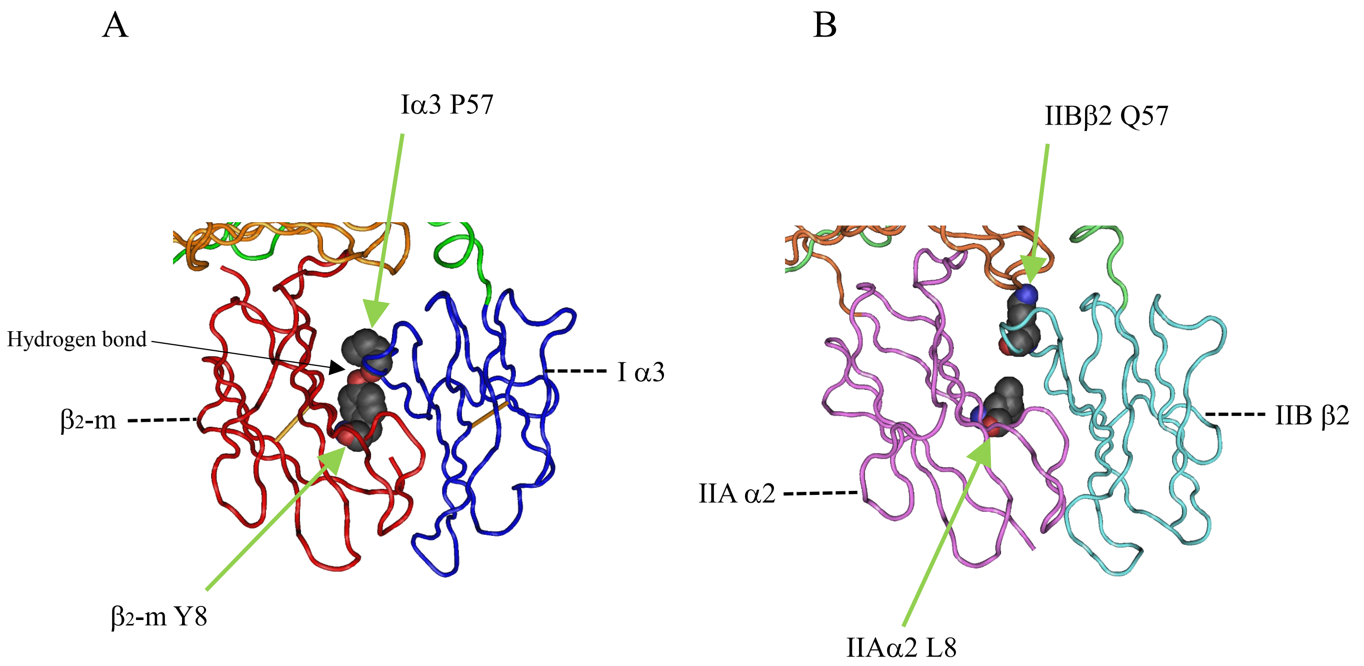

Fig. S6. MHC class I α3 domain P57 interacts with Y8 of β_2-m.
A. The main chain carbonyl oxygen of MHC class I α3 P57 interacts with a hydrogen atom of a hydroxyl group of β_2-m Y8 through a hydrogen bond (29).
B. The residues of the classical MHC class II molecule whose positions are corresponding to class I α3 P57 and Y8 of β_2-m are shown.
HLA-A2 (PDB ID: 1QSF) and HLA-DR1 (PDB ID: 1DLH) were used for MHC class I and class II molecules, respectively. The relevant amino acid residues with their side- and main-chains are presented using the space-filling model representation. Oxygen: red, nitrogen: blue, and carbon: black.

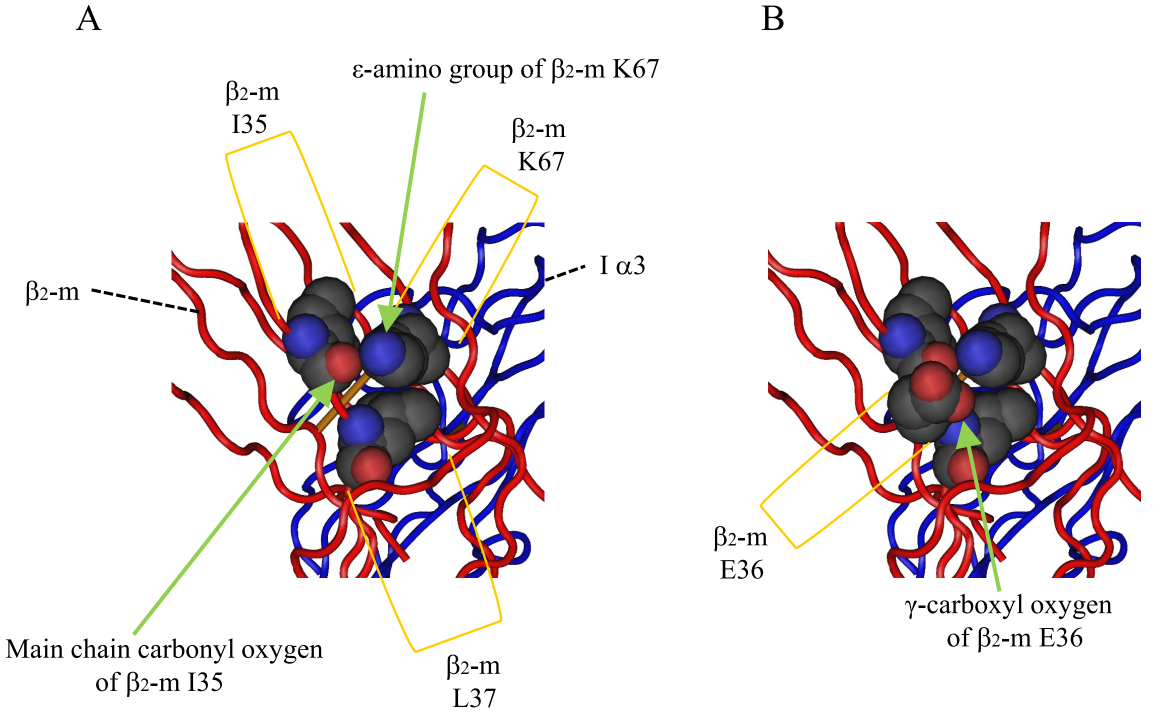

Fig. S7. β2-m K67 can form intra-domain hydrogen bonds.
A, A structural drawing centered on grass carp β2-m K67 is shown. A hydrogen atom from ε-amino group of grass carp β2-m K67 forms a hydrogen bond with a main chain carbonyl oxygen of β2-m I35 (69). The ε-amino group of grass carp β2-m K67 and the main chain carbonyl oxygen of β2-m I35 are indicated with green arrows. In addition to β2-m K67 and β2-m I35, β2-m L37 whose sequence position is corresponding to that of the conserved W37 of the Ig-superfamily, is also shown. The relevant three amino acid residues with their side- and main-chains are shown as the space-filling model and their approximate locations are indicated with yellow brackets. β2-m (red) and class I α3 domain (blue) are indicated. **B,** The same structure from the same angle as shown in A, but also showing the space-filling model of β2-m E36 (indicated with a yellow bracket). β2-m K67 forms a hydrogen bond to a γ-carboxyl oxygen of β2-m E36 (69).

Grass carp class I molecule (PDB ID: 5Y91) was used for the drawing. Oxygen: red, nitrogen: blue, and carbon: black.

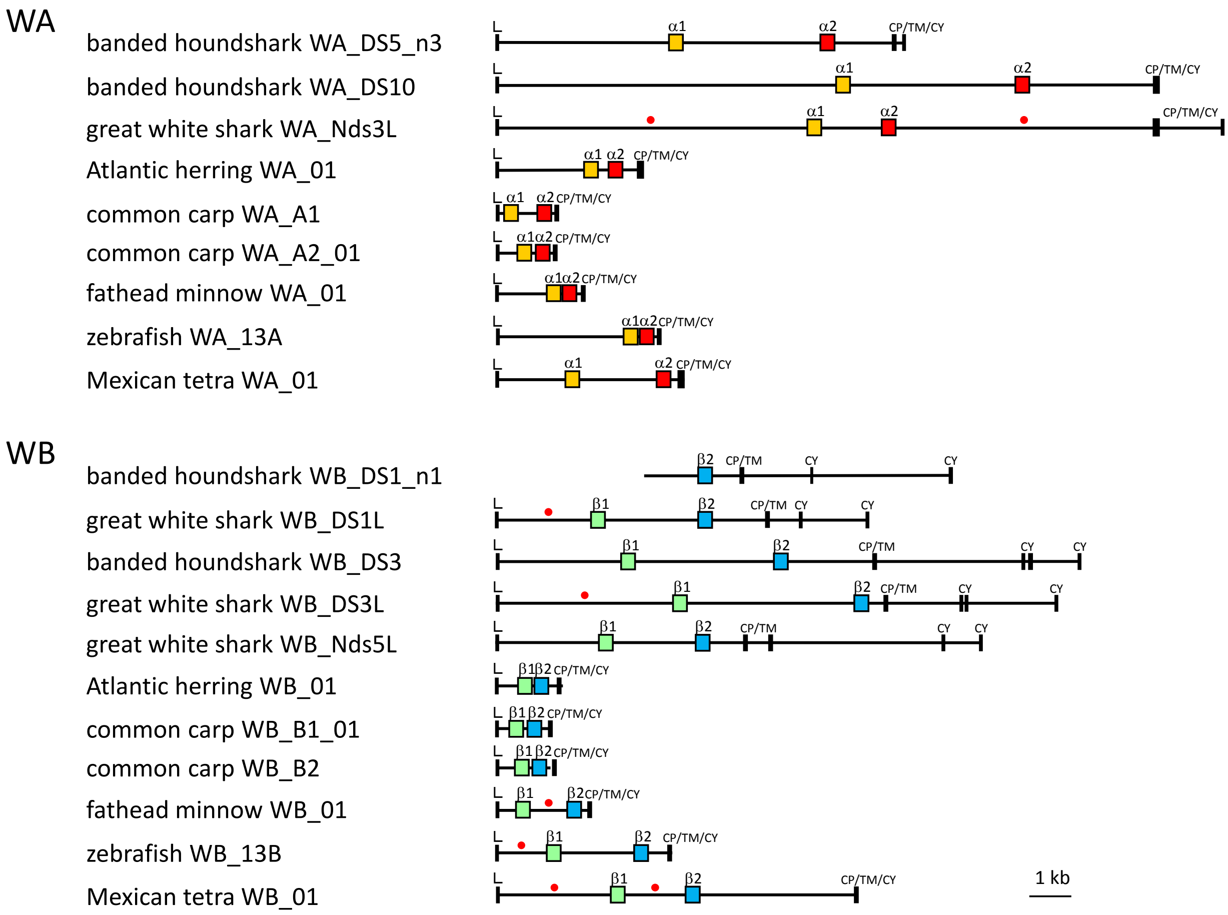

Fig. S8. The genomic structures of additional W-category genes with class II domain architectures. The genomic structures of coding exons and introns of additional *WA* and *WB* genes are shown. The genomic sequence of banded houndshark *WB_DS1_n1* was partially determined and its 5' side has not yet been clarified. The genomic sequence of banded houndshark *WB_DS3* was determined using two parts which contain the first four and the last three exons, respectively. Colored boxes are explained in the legend to Fig. 5. Within some intron sequences, undetermined regions in the database information exist and those introns are indicated by red filled circles.

Fig. S9

A banded houndshark WA_DS5

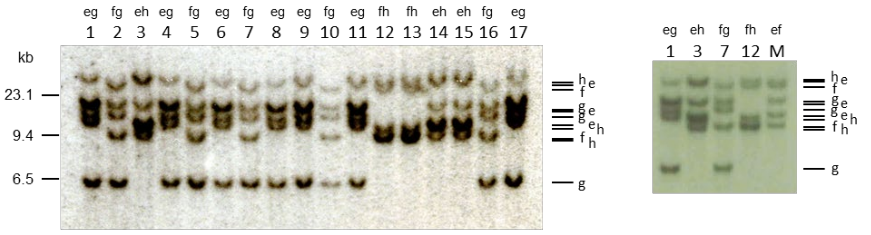

B banded houndshark WA_DS10

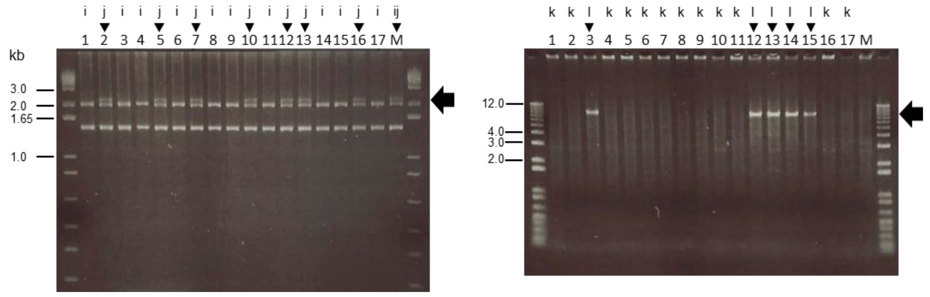

C banded houndshark WB_DS1

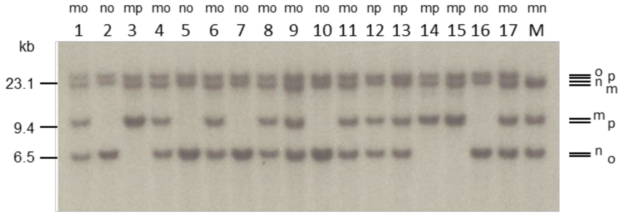

D banded houndshark WB_DS3

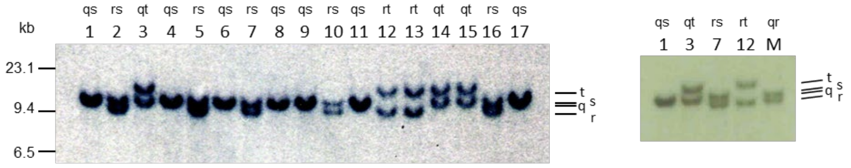

E Haplotype classification

		1	2	3	4	5	6	7	8	9	10	11	12	13	14	15	16	17	M	F
classical MHC	maternal	*a*	*a*	*b*	*b*	*a*	*b*	*b*	*b*	*b*	*a*	*a*	*b*	*a*	*a*	*b*	*b*	*b*	*ab*	
	paternal	*c*	*c*	*c*	*c*	*c*	*c*	*d*	*d*	*c*	*c*	*d*	*d*	*d*	*c*	*c*	*c*	*d*		*cd*
WA_DS5	maternal	e	f	e	e	f	e	f	e	e	f	e	f	f	e	e	f	e	ef	
	paternal	g	g	h	g	g	g	g	g	g	g	g	h	h	h	h	g	g		gh
WA_DS10	HhaI-group	i	j	i	i	j	i	j	i	i	j	i	j	j	i	i	j	i	ij	
	Amp-group	k	k	l	k	k	k	k	k	k	k	k	l	l	l	l	k	k		kl
WB_DS1	maternal	m	n	m	m	n	m	n	m	m	n	m	n	n	m	m	n	m	mn	
	paternal	o	o	p	o	o	o	o	o	o	o	o	p	p	p	p	o	o		op
WB_DS3	maternal	q	r	q	q	r	q	r	q	q	r	q	r	r	q	q	r	q	qr	
	paternal	s	s	t	s	s	s	s	s	s	s	s	t	t	t	t	s	s		st

Fig. S9. All four investigated types of banded houndshark W-category genes belong to the same linkage group. Panel A, C and D show Southern blots for linkage analyses of DNA samples from seventeen littermates and their mother using *WA_DS5*, *WB_DS1* and *WB_DS3* probes, respectively. Panel B shows PCR amplification of *WA_DS10* for linkage analyses because of a limitation of the shark DNA samples. Panel E shows the summary of the linkage analyses.

A, Southern blot analysis using banded houndshark *WA_DS5* as probe. Left: Positive bands of seventeen littermates exhibit four patterns, which are shown with the represented haplotype symbol combination (e-h, indicated on top) with lane numbers as follows: (1, 4, 6, 8, 9, 11, 17; eg), (2, 5, 7, 10, 16; fg), (3, 14, 15; eh) and (12, 13; fh). Based on similar arguments as in the previous studies (17, 33), these four patterns could represent four distinct haplotype patterns produced by total of four different haplotypes of their parents (maternal, e and f; paternal, g and h) through Mendelian inheritance. Positive bands were assigned to one of the haplotypes (indicated on the right), in some cases with difficulties because of limited resolution. The DNA lengths of the size markers in kilo-bases (kb) are indicated on the left side. Right: Southern blot analysis of mothers's DNA along with representative littermates. The experiment described in the left picture did not contain mother's DNA.

B, PCR amplification of banded houndshark *WA_DS10*. Left: DNA fragments amplified from the seventeen littermates and their mother's DNA with two primers (forward: HhaI-group F1, reverse: HhaI-group R1 described in Table S2) were digested with the restriction enzyme *Hha*I and the products analyzed with agarose gel (3 %) electrophoresis. All samples with an additional band (as indicated with an arrow on the right hand side) are indicated by arrowheads (on top). The seventeen littermates could be classified into two groups; an HhaI-group "i" and an HhaI-group "j" with the latter showing an additional band. Right: DNA fragments amplified from seventeen littermates and their mother's DNA with two primers (forward: Amp-group F1, reverse: Amp-group R1), analyzed with agarose gel (1 %) electrophoresis. All samples with amplification of DNA fragments of approximately 8 kb in size (as indicated with an arrow on the right hand side) are indicated by arrowheads (on top). The seventeen littermates could be classified into two groups; an Amp-group "k" and an Amp-group "l" with the latter showing an 8 kb band. Variations observed in the results of the left and right experiments are considered to represent haplotype variations. Combining these results indicates four patterns, shown with the represented group (haplotype) symbol combinations along with lane numbers as follows: (1, 4, 6, 8, 9, 11, 17; ik), (2, 5, 7, 10, 16; jk), (3, 14, 15; il) and (12, 13; jl).

C, Southern blot analysis using banded houndshark *WB_DS1* as probe. Like in A, positive bands of seventeen littermates exhibit four patterns, which are shown with the represented haplotype symbol combination with lane numbers as follows: (1, 4, 6, 8, 9, 11, 17; mo), (2, 5, 7, 10, 16; no), (3, 14, 15; mp) and (12, 13; np).

D, Southern blot analysis using banded houndshark *WB_DS3* as probe. Like in A, positive bands of seventeen littermates exhibit four patterns, which are shown with the represented haplotype symbol combination with lane numbers as follows: (1, 4, 6, 8, 9, 11, 17; qs), (2, 5, 7, 10, 16; rs), (3, 14, 15; qt) and (12, 13; rt).

E, Summary of the haplotype classification of W-category and classical MHC genes. The number of the banded houndshark individual littermate, mother (M) and father (F) are shown on top. In the first row, previously published haplotype classifications of classical MHC genes (17, 33) are shown with four different colors (purple, light blue, orange and pale yellow). In the following rows, haplotype classifications of banded houndshark *W-category* genes are shown in addition to the deduced haplotype of the father. Identical patterns are observed for *WA_DS5*, *WA_DS10*, *WB_DS1* and *WB_DS3* as shown with four different colors (red, green, yellow and blue). This indicates that in banded houndshark all four investigated types of W-category genes belong to the same linkage group. The distribution of the four W-category haplotypes (red, green, yellow and blue) over the littermates is completely different from the distribution of the classical MHC genes haplotypes (purple, light blue, orange and pale yellow), indicating that the four investigated W-category genes are not linked with the classical MHC genes. Linkage analysis of banded houndshark *β_2-m* gene was conducted

separately and shown to be linked with the classical MHC genes (*SI Appendix* and Fig. S15) as also reported for another shark species (28).

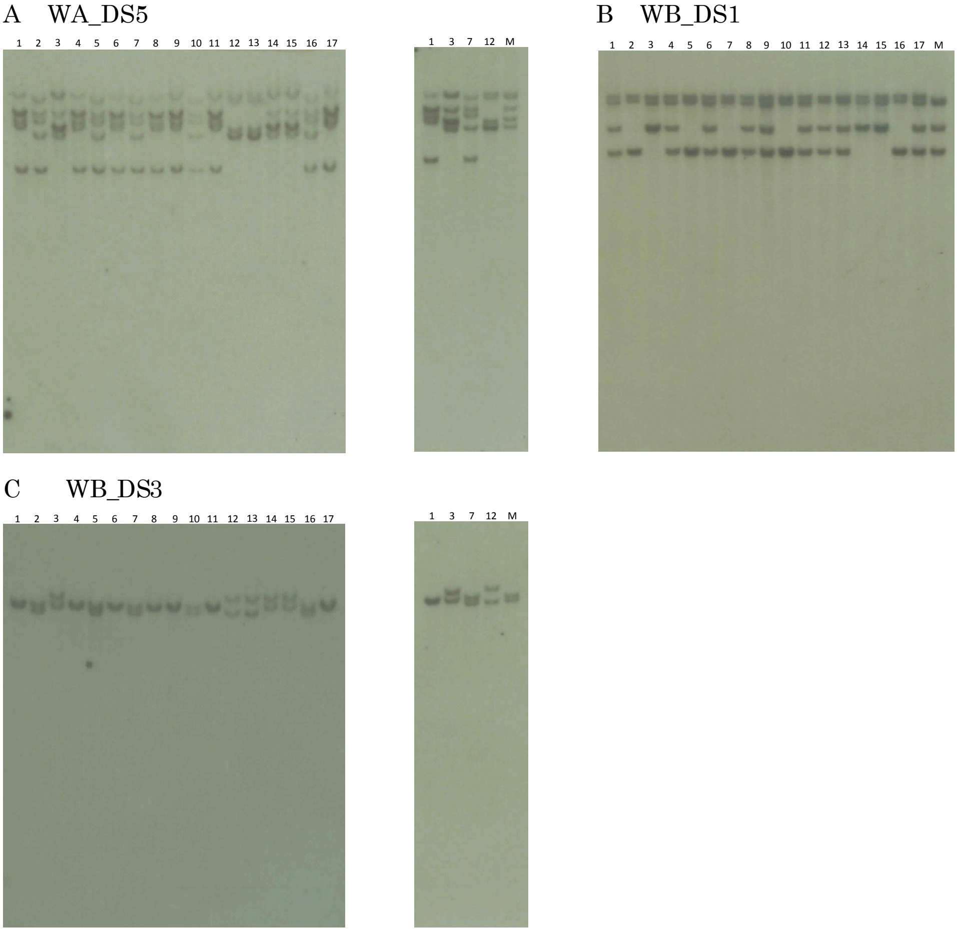

Fig. S10. Full pictures of the Southern blots used for linkage analyses in Fig. S9.
A, probe: banded houndshark *WA_DS5*, **B**, probe: banded houndshark *WB_DS1*, **C**, probe: banded houndshark *WB_DS3*. The numbers indicate individual sharks and "M" indicates their mother as in Fig. S9. There are no positive bands in the blots other than those shown in Fig. S9. Agarose gels of 0.8 % were used.

Fig. S11

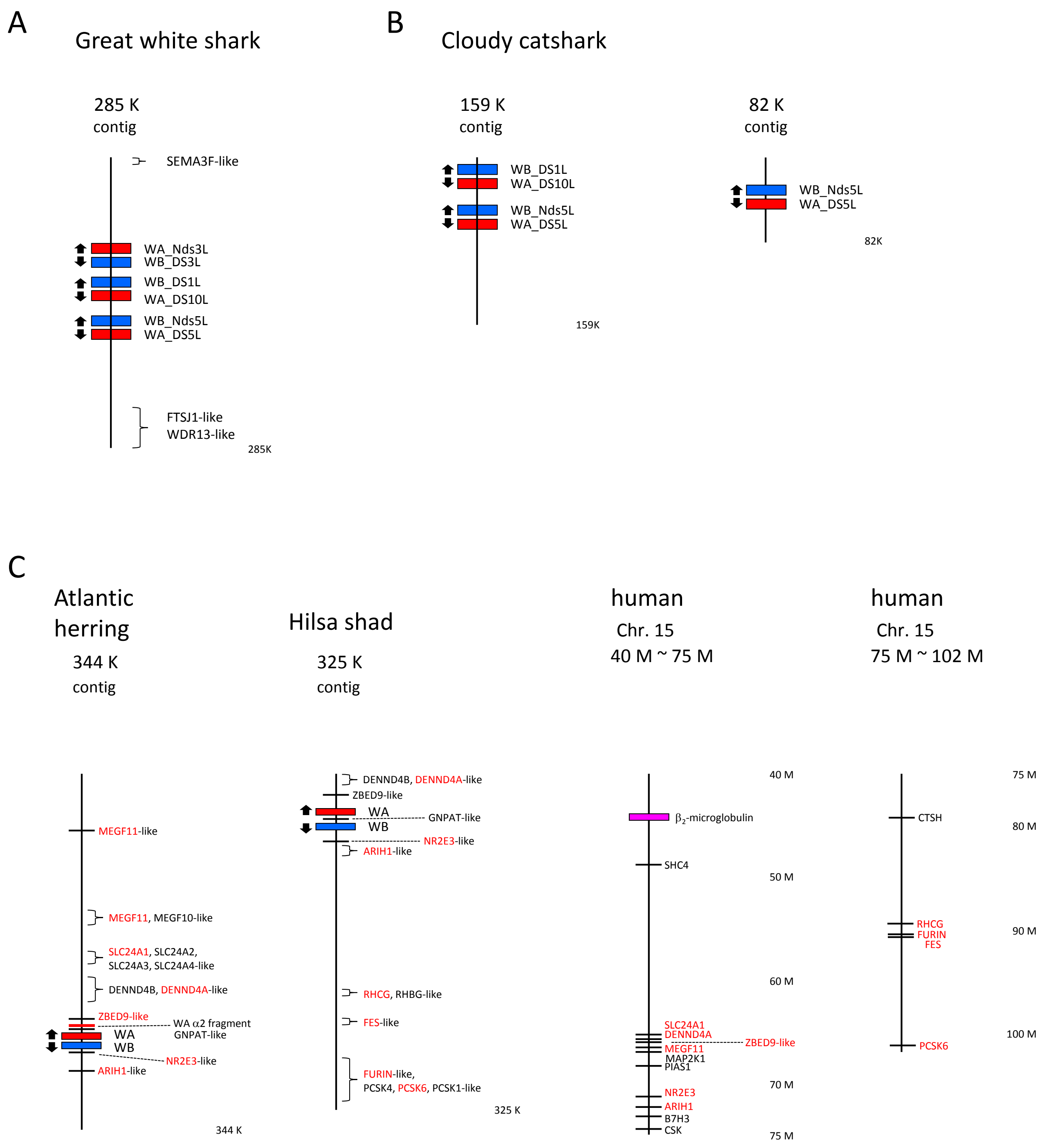
A
Great white shark
285 K
contig
SEMA3F-like
WA_Nds3L
WB_DS3L
WB_DS1L
WA_DS10L
WB_Nds5L
WA_DS5L
FTSJ1-like
WDR13-like
285K
B
Cloudy catshark
159 K
contig
WB_DS1L
WA_DS10L
WB_Nds5L
WA_DS5L
159K
82 K
contig
WB_Nds5L
WA_DS5L
82K
C
Atlantic herring
344 K
contig
MEGF11-like
MEGF11, MEGF10-like
SLC24A1, SLC24A2,
SLC24A3, SLC24A4-like
DENND4B, DENND4A-like
ZBED9-like
WA α2 fragment
GNPAT-like
WA
WB
NR2E3-like
ARIH1-like
344 K
Hilsa shad
325 K
contig
DENND4B, DENND4A-like
ZBED9-like
WA
GNPAT-like
WB
NR2E3-like
ARIH1-like
RHCG, RHBG-like
FES-like
FURIN-like,
PCSK4, PCSK6, PCSK1-like
325 K
human
Chr. 15
40 M ~ 75 M
40 M
β2-microglobulin
SHC4
50 M
60 M
SLC24A1
DENND4A
ZBED9-like
MEGF11
MAP2K1
PIAS1
70 M
NR2E3
ARIH1
B7H3
CSK
75 M
human
Chr. 15
75 M ~ 102 M
75 M
CTSH
80 M
RHCG
FURIN
FES
90 M
100 M
PCSK6

Fig. S11

D

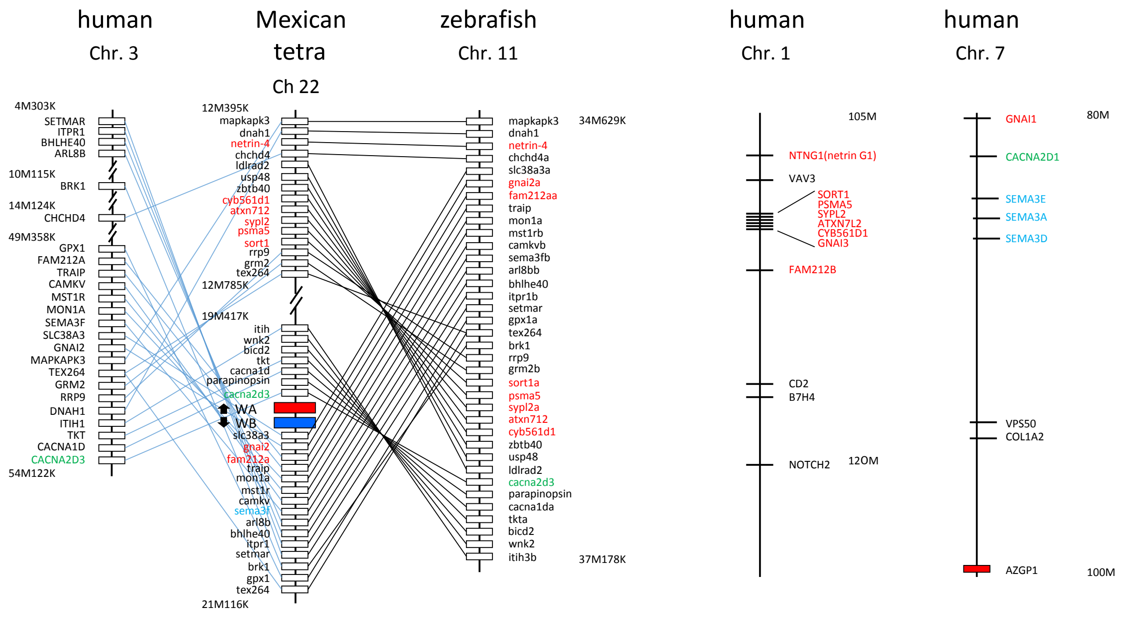

human
Chr. 3
Mexican
tetra
Ch 22
zebrafish
Chr. 11
human
Chr. 1
human
Chr. 7
4M303K
SETMAR
ITPR1
BHLHE40
ARL8B
10M115K
BRK1
14M124K
CHCHD4
49M358K
GPX1
FAM212A
TRAIP
CAMKV
MST1R
MON1A
SEMA3F
SLC38A3
GNAI2
MAPKAPK3
TEX264
GRM2
RRP9
DNAH1
ITIH1
TKT
CACNA1D
CACNA2D3
54M122K
12M395K
mapkapk3
dnah1
netrin-4
chchd4
ldlrad2
usp48
zbtb40
cyb561d1
atxn712
sypl2
psma5
sort1
rrp9
grm2
tex264
12M785K
19M417K
itih
wnk2
bicd2
tkt
cacna1d
parapinopsin
cacna2d3
WA
WB
slc38a3
gnai2
fam212a
traip
mon1a
mst1r
camkv
sema3f
arl8b
bhlhe40
itpr1
setmar
brk1
gpx1
tex264
21M116K
mapkapk3
dnah1
netrin-4
chchd4a
slc38a3a
gnai2a
fam212aa
traip
mon1a
mst1rb
camkvb
sema3fb
arl8bb
bhlhe40
itpr1b
setmar
gpx1a
tex264
brk1
rrp9
grm2b
sort1a
psma5
sypl2a
atxn712
cyb561d1
zbtb40
usp48
ldlrad2
cacna2d3
parapinopsin
cacna1da
tkta
bicd2
wnk2
itih3b
34M629K
37M178K
105M
NTNG1(netrin G1)
VAV3
SORT1
PSMA5
SYPL2
ATXN7L2
CYB561D1
GNAI3
FAM212B
CD2
B7H4
NOTCH2
120M
80M
GNAI1
CACNA2D1
SEMA3E
SEMA3A
SEMA3D
VPS50
COL1A2
AZGP1
100M

E

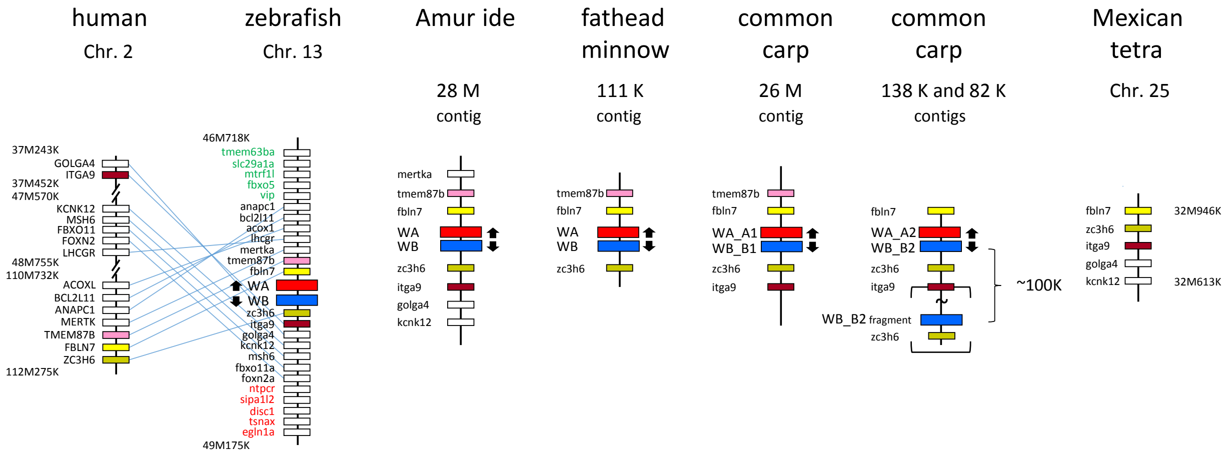

human
Chr. 2
zebrafish
Chr. 13
Amur ide
28 M
contig
fathead
minnow
111 K
contig
common
carp
26 M
contig
common
carp
138 K and 82 K
contigs
Mexican
tetra
Chr. 25
37M243K
GOLGA4
ITGA9
37M452K
47M570K
KCNK12
MSH6
FBXO11
FOXN2
LHCGR
48M755K
110M732K
ACOXL
BCL2L11
ANAPC1
MERTK
TMEM87B
FBLN7
ZC3H6
112M275K
46M718K
tmem63ba
slc29a1a
mtrf1l
fbxo5
vip
anapc1
bcl2l11
acox1
lhcgr
mertka
tmem87b
fbln7
WA
WB
zc3h6
itga9
golga4
kcnk12
msh6
fbxo11a
foxn2a
ntpcr
sipa1l2
disc1
tsnax
egln1a
49M175K
mertka
tmem87b
fbln7
WA
WB
zc3h6
itga9
golga4
kcnk12
tmem87b
fbln7
WA
WB
zc3h6
tmem87b
fbln7
WA_A1
WB_B1
zc3h6
itga9
fbln7
WA_A2
WB_B2
zc3h6
itga9
WB_B2 fragment
zc3h6
~100K
fbln7
zc3h6
itga9
golga4
kcnk12
32M946K
32M613K

Fig. S11

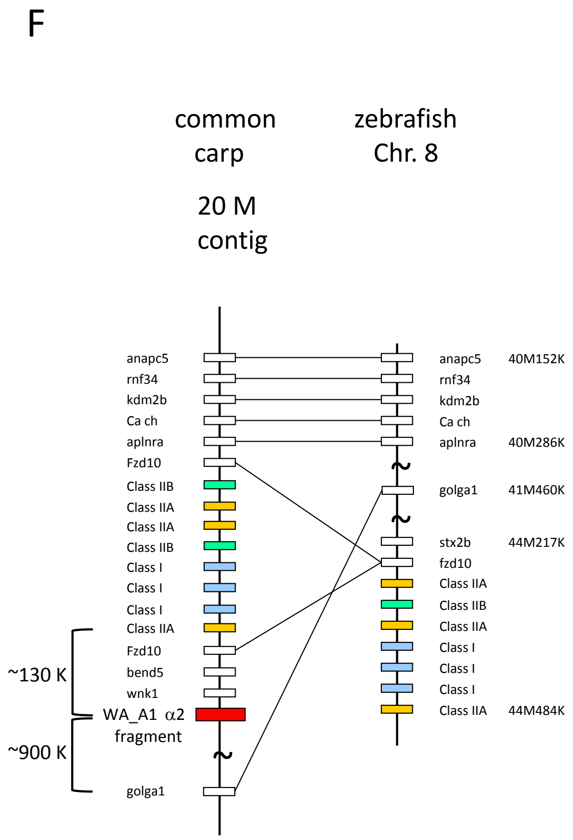

F
common carp
20 M contig
zebrafish Chr. 8
anapc5
rnf34
kdm2b
Ca ch
aplnra
Fzd10
Class IIB
Class IIA
Class IIA
Class IIB
Class I
Class I
Class I
Class IIA
Fzd10
bend5
wnk1
WA_A1 α2 fragment
golga1
~130 K
~900 K
anapc5 40M152K
rnf34
kdm2b
Ca ch
aplnra 40M286K
golga1 41M460K
stx2b 44M217K
fzd10
Class IIA
Class IIB
Class IIA
Class I
Class I
Class I
Class IIA 44M484K

Fig. S11. Genomic locations of W-category genes of cartilaginous fish and of teleost fish.
A, Great white shark 285 K contig containing three *WA* (red) and three *WB* (blue) genes. There are three *WA*/*WB* pairs with head-to-head configurations, although the pairing remains to be studied at the protein level. The three pairs are: *WA_Nds3L*/*WB_DS3L*, *WA_DS10L*/*WB_DS1L*, and *WA_DS5L*/*WB_Nds5L*. In the same contig, in addition to the *WA* and *WB* genes, some gene fragment sequences were identified in the surrounding regions of *WA*/*WB* genes as described below.

SEMA3F-like sequence (~144 bp), corresponding to the coding sixteenth exon (total 19 exons) sequence of human *SEMA3F* (chromosome 3p21.31, 50 M 155 K) transcript variant 1 (NM_004186). A blast search of the Mexican tetra genome with this *SEMA3F*-like sequence revealed the *sema3f* gene situated near the W-category genes (see D) as the top match.

FTSJ1-like sequences of ~69 bp and ~93 bp corresponding to the fourth and the eleventh coding exon sequences, respectively, of human *FTSJ1* (chromosome Xp11.23, 48 M 476 K) transcript variant 1 (NM_012280).

WDR13-like sequences (length: ~94 to ~316 bp with mixed orientations) corresponding to six coding exon sequences including the exons 4 and 6-10 of human *WDR13* (chromosome Xp11.23, 48 M 590 K) transcript variant 4 (NM_001347217). The closest relative of human *WDR13* gene, *WDR5*, is present at the human chromosome 9q34.2 that was previously regarded as an *Mhc* paralogous region (87, 88). *WDR5B*, a sequence homologous to *WDR5*, is located on human chromosome 1 (~122 M) close to the *CASR* gene (see the axolotl W-category contig; Fig. S12B) and close to other *Mhc* region-related genes.

B, Cloudy catshark 159 K and 82 K contigs containing *WA* (red) and *WB* (blue) genes. The order of the genes is the same as that of great white shark in A. *WA_DS5L*/*WB_Nds5L* gene pairs of the two contigs are distinct from each other (*SI Appendix*).

C, Atlantic herring 344 K contig and Hilsa shad 325 K contig containing *WA* (red) and *WB* (blue) genes.

Genes which are homologous to the sequences (depicted with red color) in the surrounding regions of the *WA*/*WB* gene pair in these contigs can be found in the human chromosome 15 (shown on the right), which is closely related to the *Mhc* region (28, 87, 88) and where the β_2-microglobulin (β_2-m) gene and many other *Mhc* related genes are present. Some selected sequences in the surrounding regions of the *WA*/*WB* gene pair and their homologies with the human genes are described below.

In the Atlantic herring 344 K contig:

MEGF11-like sequences correspond to a part of the coding sixth exon of human *MEGF11* (chromosome 15q22.31, 66 M 253 K) mRNA sequence (NM_032445) with 73 % identity (176/240), to a part of the coding twenty-second exon with 86 % identity (95/110), and to a part of the coding eighth and ninth exons with 83 % identity (87/105). These sequences also show similarity to a part of human *MEGF10* gene (chromosome 5q23.2, 127 M 229 K).

A *SLC24A1*-like sequence corresponds to a part of the coding fourth exon of human *SLC24A1* (chromosome 15q22.31, 65 M 611 K) transcript variant 5 (NM_001301033.2) with 77 % identity (414/539). Another *SLC24A1*-like sequence corresponds to the coding tenth exon with 84 % identity (148/177). These sequences also show similarity with a part of human *SLC24A2* (chromosome 9p22.1-p21.3), *SLC24A3* (chromosome 20p11.23) and *SLC24A4* (chromosome 14q32.12) sequences.

In both Atlantic herring and Hilsa shad contigs (described using the results of Atlantic herring):

DENND4B-like sequence stretch corresponds to a part of the coding sixth exon of human *DENND4B* (chromosome 1q21.3, 153 M 946 K) transcript variant 2 (NM_001367466) with 84 % identity (118/140 or 108/129), to the coding twelfth exon with 76 % identity (139/184), to the coding seventeenth and eighteenth exon with 72 % identity (156/217), and to the coding twenty-fourth exon with 76 % identity (108/142).

Some *DENND4B* -like sequences are also *DENND4A*-like, and *DENND4A*-like sequences correspond to various portions of human *DENND4A* (chromosome 15q22.31, 65 M 792 K).

ZBED9-like sequence corresponds to the non-coding sequence of human *ZBED9* (chromosome 6p22.1, 28M 616K, in the *Mhc* region) transcript vatiant X2 mRNA sequence (XM_011514285) with 90 % identity (65/72). A blastn search of the human genome with the Atlantic herring *ZBED9*-like noncoding 72 bases revealed the sequences of chromosome 6, 15 and 17 as the top matches: chromosome 6, 18 M 836 K, 27 M 519 K, 28 M 941 K; chromosome 15, 65 M 869 K; chromosome 17, 8 M 119 K. On human chromosome 6, the locations of some MHC class I genes are as follows: HFE (26 M 87 K), HLA-F (29 M 723 K), HLA-G (29 M 826 K), HLA-A (29 M 942 K), HLA-E (30 M 489 K), HLA-C (31 M 272 K), HLA-B (31 M 357 K), MICA (31 M 400 K), MICB (31 M 494 K). The location of the homologous sequence of *ZBED9*-like noncoding sequence on human chromosome 15 (65 M 869 K) is indicated in the figure.

GNPAT-like sequence corresponds to the coding seventh exon of human *GNPAT* (chromosome 1q42.2, 231 M 241 K) transcript variant 1 mRNA sequence with 69 % identity (88/127). Near the W-category genes of zebrafish, fourteen genes that are homologous to the genes found in the human chromosome 1 (~231 M) exist.

A *NR2E3*-like sequence corresponds to the coding second exon of human *NR2E3* (chromosome 15q23, 71 M 810 K) transcript variant 1 mRNA sequence (NM_016346) with 82 % identity (106/129). Another *NR2E3*-like sequence corresponds to a part of the coding fifth exon with 90 % identity (77/86).

ARIH1-like sequences correspond to the coding tenth exon of human *ARIH1* (chromosome 15q24.1, 72 M 474 K) mRNA sequence (NM_005744) with 81 % identity (113/139) and to the twelfth exon with 87 % identity (228/263).

In the Hilsa shad 325 K contig:

RHCG-like sequences correspond to the coding second exon of human *RHCG* (chromosome 15q26.1, 89 M 496 K) transcript variant 3 mRNA sequence (NM_001321041) with 88 % identity (137/155), to the coding sixth exon with 79 % identity (97/123), to the coding fifth exon with 74 % identity (120/175), to the coding fourth exon with 73 % identity (108/147), and to the coding seventh exon with 70 % identity (84/120).

Some *RHCG*-like sequences are also *RHBG*-like, and these *RHBG*-like sequences correspond to the coding third exon of human RHBG (chromosome 1q22, 156 M 366 K) transcript variant 3 mRNA sequence (NM_001256396) with 78 % identity (124/159), to the coding fifth exon with 71 % identity (103/146), to a part of the coding sixth exon with 75 % identity (63/84), and to a part of the coding eighth exon with 81 % identity (44/54).

FES-like sequences correspond to the coding fifteenth exon of human FES (chromosome 15q26.1, 90 M 884 K) transcript variant 4 mRNA sequence (NM_001143785) with 78 % identity (123/158), to the coding sixteenth exon with 78 % identity (102/131), to the coding thirteenth exon with 83 % identity (81/98), to the coding twelfth exon with 72 % identity (89/124), and to a part of the coding fourteenth exon with 84 % identity (38/45).

FURIN-like sequences correspond to the coding ninth exon of human FURIN (chromosome 15q26.1, 90 M 868 K) transcript variant 1 mRNA sequence (NM_002569) with 85 % identity (185/217), to the coding eighth exon with 77 % identity (137/179), to a part of the coding thirteenth exon with 81 % identity (88/108), to the coding tenth exon with 81 % identity (82/101), to the coding fourteenth exon with 81 % identity (80/99), to the coding twelfth exon with 80 % identity (80/100), to the coding sixth exon with 82 % identity (67/82), to the coding seventh exon with 79 % identity (73/92), to the coding fourth exon with 78 % identity (69/89), and to the coding eleventh exon with 74 % identity (79/107).

PCSK4-like sequences correspond to the coding eighth exon of human *PCSK4* (chromosome 19p13.3, 1 M 490 K) transcript variant X22 mRNA sequence (XM_024451556) with 81 % identity (152/188), to the coding seventh exon with 76 % identity (134/176), to a part of the coding twelfth exon with 76 % identity (90/118), and to the coding tenth exon with 74 % identity (72/97). Identification of exons are based on *PCSK4* mRNA sequence (NM_017573).

Some *PCSK4*-like sequences are also *PCSK6*-like, and these *PCSK6*-like sequences correspond to the coding eighth exon of human *PCSK6* (chromosome 15q26.3, 101 M 489 K) transcript variant 3 mRNA

sequence (NM_138322) with 77 % identity (137/177), to the coding seventh exon with 77 % identity (115/150), and to the coding fourth exon with 76 % identity (71/94).

D, Mexican tetra chromosome 22 containing *WA* (red) and *WB* (blue) genes. The pair of *WA*/*WB* genes is sandwiched in between *slc38a3* and *cacna1d3* genes.

On human chromosome 3 (shown on the left of the Mexican tetra's), homologous genes for many surrounding genes of the Mexican tetra *WA*/*WB* gene pair can be found (indicated by blue lines).

In the corresponding region of zebrafish chromosome 11 (shown on the right of the Mexican tetra's), *WA* and *WB* genes are not present, but present on zebrafish chromosome 13 (see E).

In the region of the human chromosome 1 previously regarded as the *Mhc* paralogous regions (87, 88) (second from the right), homologous genes or closely related genes for some surrounding genes of the Mexican tetra *WA*/*WB* gene pair can be found (shown in red color), and these are also indicated in the figure of the Mexican tetra and zebrafish chromosomal regions.

In the region of 80 M~100 M of human chromosome 7 which includes MHC nonclassical MHC class I gene (*AZGP1*) (shown on the far right), closely related genes for some surrounding genes of the Mexican tetra *WA*/*WB* gene pair can be found (shown in red or green color). VPS50 and COL1A2 genes are present near *AZGP1*. In the human *Mhc* region, VPS52 (chromosome 6p21.32, 33 M 271 K) and COL11A2 (chromosome 6p21.32, 33 M 193 K) are located close to each other like VPS50 and COL1A2 shown in the figure.

In the surrounding region of the Mexican tetra *WA*/*WB* gene pair, *sema3f* gene, whose related gene fragment can be found near the great white shark W-category genes (shown in A), is highlighted in light blue color. On human chromosome 7 (on the far right), closely related genes for this *sema3f* gene are also highlighted in light blue color.

E, Zebrafish chromosome 13 containing *WA* (red) and *WB* (blue) genes. Genomic regions of zebrafish relatives, e. g., Amur ide, fathead minnow and common carp, also containing a *WA*/*WB* gene pair.
The *WA/WB* pair of zebrafish and its relatives is located between *fibulin* 7 gene (*fbln7*) and *zinc finger CCCH-type containing 6* gene (*zc3h6*).

On human chromosome 2 (shown on the left of the zebrafish's), homologous genes for many surrounding genes of the zebrafish *WA*/*WB* gene pair can be found (indicated by blue lines).

In the region of zebrafish chromosome 13 shown, homologous genes for several genes in green color are located on human chromosome 6: *TMEM63B* (6p21.1, 44 M 126 K), *SLC29A1* (6p21.1, 44 M 219 K), *MTRF1L* (6q25.2, 153 M 3 K), *FBXO5* (6q25.2, 152 M 983 K), and *VIP* (6q25.2, 152 M 750 K). Near human chromosome 6q25.2, several MHC related genes are present, including *ULBP1~3*, e. g., *ULBP3* (6q25.1, 150 M 69 K). Homologous genes for several genes in red color are located on human chromosome 1: *NTPCR* (1q42.2, 232 M 950 K), *SIPA1L2* (1q42.2, 232 M 630 K), *DISC1* (1q42.2, 231 M 626 K), *TSNAX* (1q42.2, 231 M 528 K), and *EGLN1* (1q42.2, 231 M 425 K). Near human chromosome 1q42.2, some *Mhc* region-related genes are present, including *AKT3* (1q43-q44, 243 M 851 K).

Amur ide 28 M contig and fathead minnow 111 K contig contain a *WA*/*WB* pair at the genomic region corresponding to the zebrafish chromosome 13.

In case of common carp, two pairs of *WA* and *WB* genes exist, namely, a pair of *WA_A1* and *WB_B1* and another pair of *WA_A2* and *WB_B2*. The common carp contig containing *WA_A1* and *WB_B1* comes in two forms with different genomic configuration, depending on the genomic source: in one form (common carp 107 K contig, *SI Appendix*), an inversion including a partial *WA_A1* was observed. The common carp contigs containing *WA_A2* and *WB_B2* also comes in two forms with different genomic configuration, depending on the genomic source (the difference indicated by a bracket): in one form (common carp 138 K contig), there exists a *WB_B2* gene fragment at around 100 kb distant from the full-length *WB_B2* gene, as shown in the figure.

Mexican tetra chromosome 25, containing *fibulin-7* gene and corresponding to the zebrafish chromosome 13 region, is shown (on the far right).

F, Common carp W-category gene fragment maps close to the nonclassical class I and class II genes. *WA* α2 domain gene fragment (red) exists near MHC class I (light blue) and class II (orange for IIA and green for IIB) genes in the common carp genome. Left, a portion of a common carp 20 M contig on which *WA_A1* α2 fragment is present. Close to this sequence, nonclassical lineage class I and nonclassical lineage class II genes are present. The genomic environment of this region partly resembles the zebrafish chromosome 8 (right) where several nonclassical class I (L-lineage), nonclassical class II genes and some other genes are located (89). Right, the region of 40M~45M of the zebrafish chromosome 8 with selected genes. "Ca ch" stands for "calcium release-activated calcium channel protein 1-like".

Additional information is described in Supplementary Information. Information on the contigs in this figure is listed in Table S3. Only selected genes are shown, and the genomic maps are schematic and not proportional to the actual distances of DNA.

Fig. S12

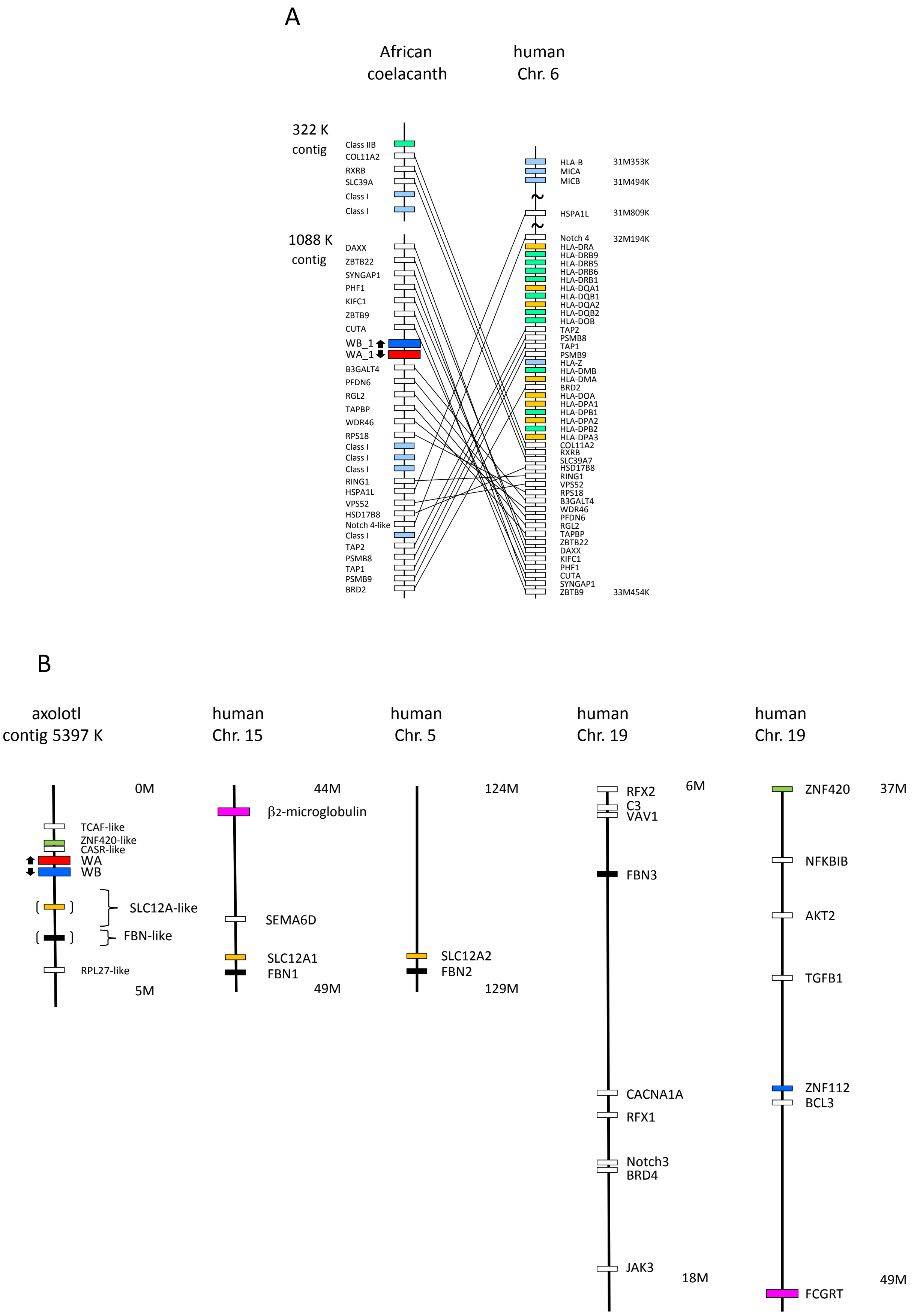
A
African coelacanth
human Chr. 6
322 K contig
1088 K contig
Class IIB
COL11A2
RXRB
SLC39A
Class I
Class I
DAXX
ZBTB22
SYNGAP1
PHF1
KIFC1
ZBTB9
CUTA
WB_1
WA_1
B3GALT4
PFDN6
RGL2
TAPBP
WDR46
RPS18
Class I
Class I
Class I
RING1
HSPA1L
VPS52
HSD17B8
Notch 4-like
Class I
TAP2
PSMB8
TAP1
PSMB9
BRD2
HLA-B
MICA
MICB
HSPA1L
Notch 4
HLA-DRA
HLA-DRB9
HLA-DRB5
HLA-DRB6
HLA-DRB1
HLA-DQA1
HLA-DQB1
HLA-DQA2
HLA-DQB2
HLA-DOB
TAP2
PSMB8
TAP1
PSMB9
HLA-Z
HLA-DMB
HLA-DMA
BRD2
HLA-DOA
HLA-DPA1
HLA-DPB1
HLA-DPA2
HLA-DPB2
HLA-DPA3
COL11A2
RXRB
SLC39A7
HSD17B8
RING1
VPS52
RPS18
B3GALT4
WDR46
PFDN6
RGL2
TAPBP
ZBTB22
DAXX
KIFC1
PHF1
CUTA
SYNGAP1
ZBTB9
31M353K
31M494K
31M809K
32M194K
33M454K
B
axolotl contig 5397 K
human Chr. 15
human Chr. 5
human Chr. 19
human Chr. 19
0M
TCAF-like
ZNF420-like
CASR-like
WA
WB
SLC12A-like
FBN-like
RPL27-like
5M
44M
β2-microglobulin
SEMA6D
SLC12A1
FBN1
49M
124M
SLC12A2
FBN2
129M
RFX2
C3
VAV1
6M
FBN3
CACNA1A
RFX1
Notch3
BRD4
JAK3
18M
ZNF420
37M
NFKBIB
AKT2
TGFB1
ZNF112
BCL3
FCGRT
49M

Fig. S12. Genomic locations of W-category genes of coelacanth and of salamander axolotl.
A, Coelacanth *WA/WB* gene pair maps within the *Mhc* region.
In the *Mhc* region of the African coelacanth, *WA_1* (red) and *WB_1* (blue) genes were identified (*SI Appendix*). The locations of various genes present in this region (34, 35) were compared with those of the corresponding human genes situated at the human *Mhc* region (chromosome 6p21.3) as shown on the right-hand side. Colors indications are as follows: MHC class I, light blue; MHC class II α chain, orange; MHC class II β chain, green.

B, Salamander axolotl *WA/WB* gene pair exists together with genes related to the *Mhc* regions.
Axolotl *WA* (red) and *WB* (blue) gene sequences were identified in one contig. The genes are not intact which may be due to some difficulties in sequencing this region (Supplementary Information).
In addition to the W-category sequences, in the surrounding regions there are notable sequences related to the *Mhc* region as described below:

ZNF420-like sequences (length ~1628 bp) covering partial coding sequences (666-2294) of the fifth exon (399-3639) of human *ZNF420* transcript variant 2 (NM_144689) with around 65 % identity. The human *ZNF420* gene exists on chromosome 19q13.12 (37 M 7 K, far right in the figure) in a region previously regarded as *Mhc* paralogous (28). In this region, the nonclassical MHC class I gene *FCGRT* (encoding FcRn protein) is located as shown. A gene homologous to the *ZNF112* gene (near the center of the region shown) was reported to exist next to the β_2-microglobulin gene in the nurse shark (28).

CASR-like sequence (length ~157 bp) corresponding to partial coding sequences (~2837-2994) of the seventh exon (2208-10150) of human *CASR* transcript variant 1 (NM_001178065) with 71 % identity (113/159). The human *CASR* gene exists on chromosome 3q13.33-q21.1 (122 M 183 K) near some *Mhc* region-related genes, e. g., *CD86* (122 M 55 K), *CD80* (119 M 559 K), *SEMA5B* (123 M 28 K) and *WDR5B* (122 M 416 K) genes. CD80 and CD86 belong to B7 family, whose members are found in the *Mhc*-related regions. *WDR5B* is closely related with *WDR13* (chromosome Xp11.23, 48 M 590 K). *WDR13*-like sequences were found near W-category genes of great white shark (Fig. S11A). The human *WDR5* gene, the homolog of *WDR5B*, exists on chromosome 9q34.2 (134 M 135 K) which was previously regarded as the *Mhc* paralogous region (87, 88).

SLC12A-like sequences are present in the region of 2.5 ~3.5 M of this contig. Examples are as follows. One *SLC12A*-like sequence corresponds to a partial coding sequence (~804-940) of the first exon (1-945) of human *SLC12A2* (chromosome 5q23.3, 128 M 83 K) transcript variant 1 (NM_001046) with 75 % identity (104/139). Another *SLC12A*-like sequence corresponds to a coding sequence (~1900-2023) including the fourteenth exon (1901-2002) of human *SLC12A1* (chromosome 15q21.1, 48 M 206 K) transcript variant 1 (NM_000338) with 80 % identity (100/125). The human *SLC12A1* and *FBN1* genes exist near β_2-microglobulin (β_2-m) gene on chromosome 15.

FBN-like sequences are present in the region of 3.5 ~ 3.9 M of this contig. *FBN*-like sequences in this contig correspond to many coding exon sequences of the human *FBN2*, *FBN3* and *FBN1* sequences. Examples are as follows. One *FBN*-like sequence corresponds to a coding sequence (~1269-1468) including the sixth exon (1271-1468) of human *FBN2* mRNA (NM_001999) with 85 % identity (170/200). Another *FBN*-like sequence corresponds to a coding sequence (~8087-8218) including the sixty-third exon (8095-8214) of human *FBN1* mRNA (NM_000138) with 82 % identity (108/132). The homologs of *FBN* genes exist on human chromosome 15q21.1, 48 M 645 K (*FBN1*), 5q23.3, 128 M 538 K (*FBN2*) and 19p13.2, 8 M 149 K (*FBN3*) which are previously regarded as *Mhc* related regions (87, 88). Both the human *SLC12A* and *FBN* homologs are present on human chromosomes 5 and 15 as shown.

The axolotl contig also contains human *TCAF1*/*TCAF2*-like and *RPL27*-like sequences as shown. The locations of these human genes on human chromosomes are: *TCAF1* and *TCAF2*, 7q35 (~143 M); *RPL27*, 17q21.31 (~43 M).

Information on the contigs in this figure is listed in Table S3. Only selected genes are shown, and the genomic maps are schematic and not proportional to the actual distances of DNA. Some annotated pseudogenes are included. Within the African coelacanth 1088 K contig, additional class I gene (fragment) sequences are present, but not indicated.

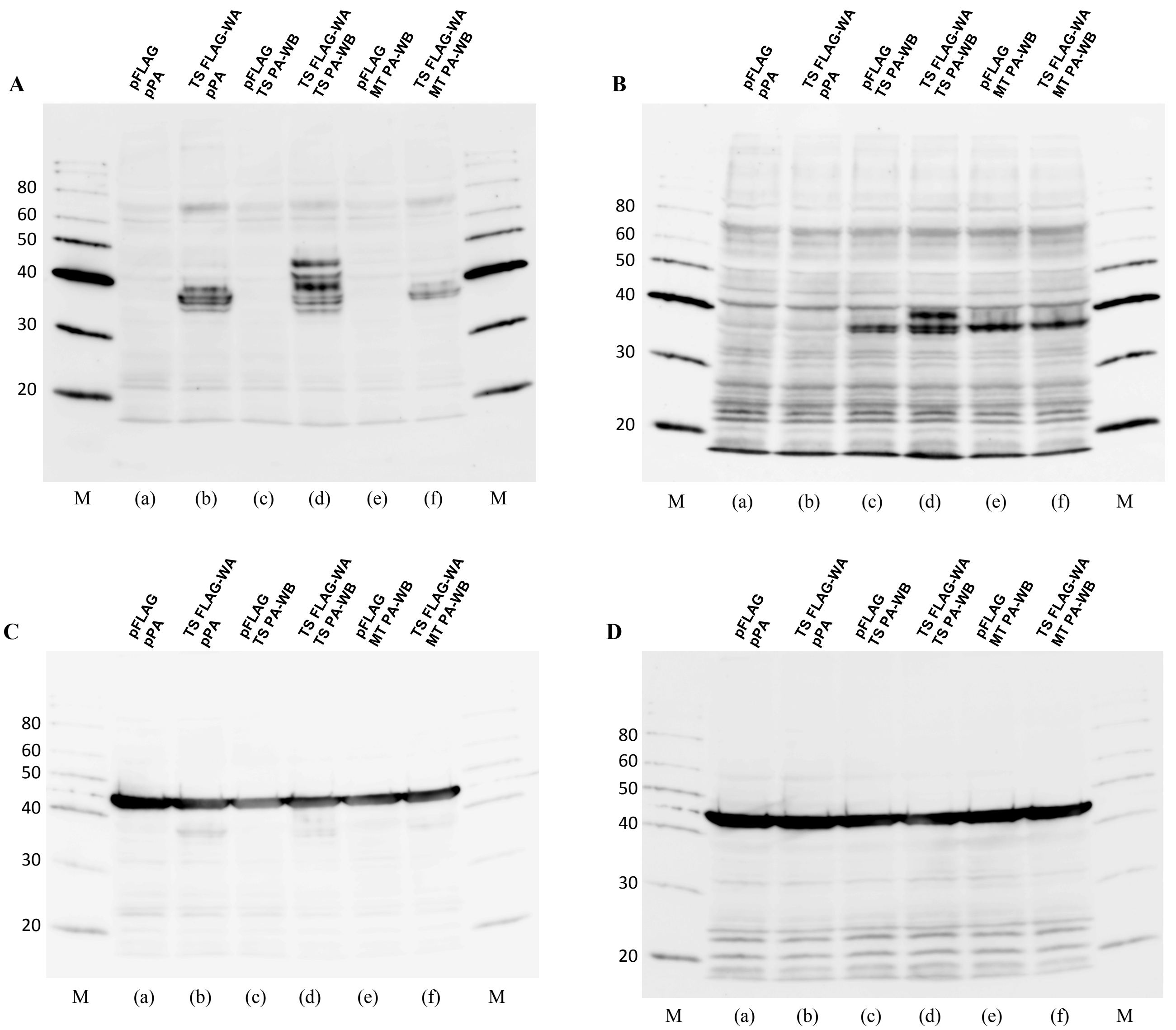

Fig. S13. Production of recombinant WA and WB proteins in transfected cells with β-actin control. Total proteins from CHO K-1 transfected cells were investigated by Western blot analyses. The panels (A) and (B) are the same as those in Fig. 6. (*A*) FLAG-tagged WA detected by anti-FLAG antibody. (*B*) PA-tagged WB detected by anti-PA antibody. (*C*) *β* -actin controls using the membrane in *A*. (*D*) *β* -actin controls using the membrane in *B*. Apparent molecular weights of protein size markers are indicated in kilo Dalton. Abbreviations are the same as those in Fig. 6. See *SI Appendix* for details.

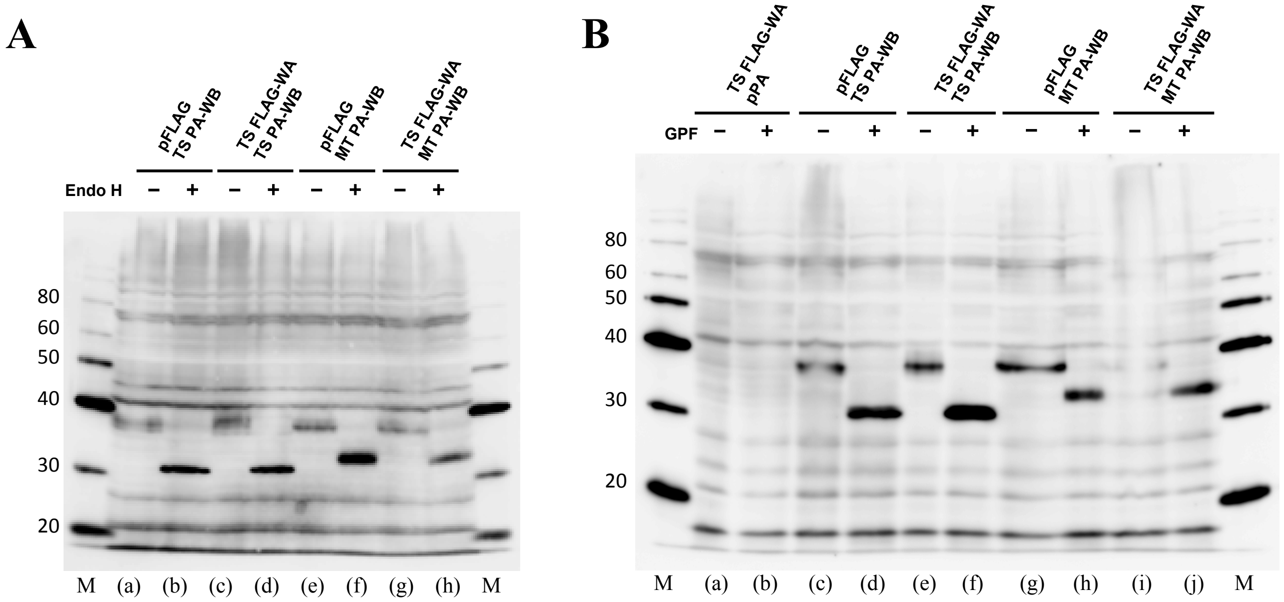

Fig. S14. Digestion of recombinant WB protein by Endoglycosidase H (Endo H) or Glycopeptidase F (GPF). Enzyme-digested total protein from transfected CHO-K1 cells were investigated by Western blot analyses. (A) PA-tagged WB with/without (+/-) Endo H digestion detected by anti-PA antibody. (B) PA-tagged WB with/without (+/-) GPF digestion detected by anti-PA antibody.

Apparent molecular weights of protein size markers are indicated in kilo Dalton. Abbreviations are the same as those in Fig. 6. See *SI Appendix* for details.

A

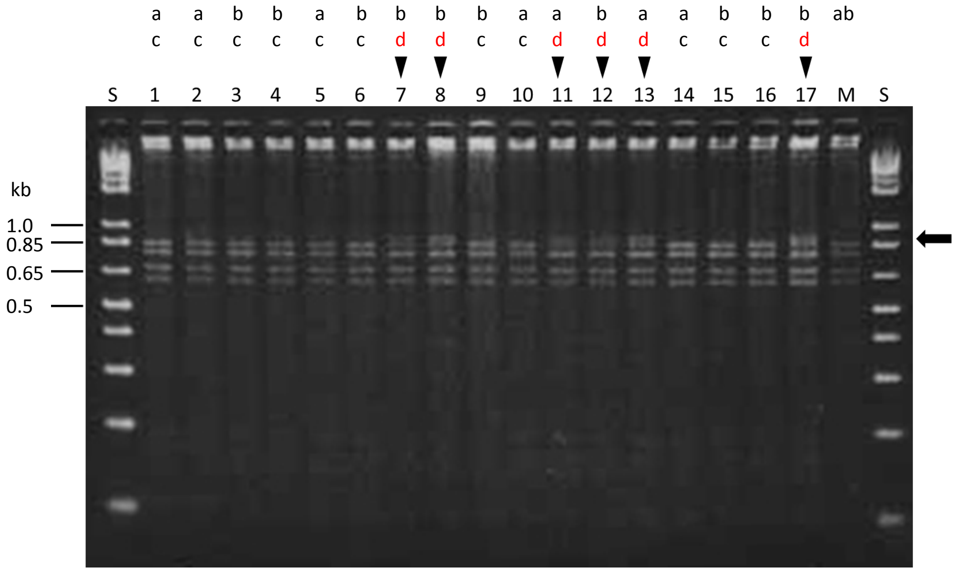

B

ATGATGCTGAAACTTGCCGTGCTGTCCTTGCTGGTGGCCCTGTGCCTGGC

TATAAGTGAGTATGGTGTCTAGGGAGCTCTTGGGGTGAATGCGCGGCCGC

TTAGCAGAGTATAGGAGAATTTAATTCGAAAGAGAACCTTTCGGCCTATC

TCTGTGCCTCC**CCTCTCT**TTGCAGGAACTCTCTCCTCAGTCCCATTCCTT

Fig. S15. Linkage analysis of banded houndshark *β2-m* gene.

A, Banded houndshark *β2-m* gene is linked with the classical MHC genes. The PCR products amplified with forward (F11) and reverse (R10) primers were digested with the restriction enzyme *Mbo*II and the products were analyzed using agarose gel electrophoresis. Arrowheads (on top) indicate the samples in which an additional band (indicated by an arrow on the right hand side) of slightly larger than 850 bp can be observed. The distribution of this additional band among the seventeen littermate sharks perfectly matches with that of the paternal haplotype "d" described in case of the classical MHC genes (Fig. S9; 17). Assumed classical MHC haplotype classifications are shown on top ("d" in red). The lengths of size markers (S) are indicated on the left. An agarose gel of 4 % was used. **B**, The genomic sequence near the leader-coding exon of the banded houndshark *β2-m* gene reveals haplotype variation. Red letters show the leader-encoding nucleotides starting with a methionine start codon, ATG. Green shading indicates a splicing signal sequence. Yellow shading indicates the seven nucleotides which are present in the paternal haplotype "d" of the banded houndshark individual No.12. The maternal haplotype "b" does not possess these seven nucleotides based on the analysis of the banded houndshark individual No.12 (haplotype "bd"). The maternal haplotype "a" and paternal haplotype "c" also do not appear to possess these seven nucleotides based on the result described in A. Additional information is described in *SI Appendix*.

Fig. S16

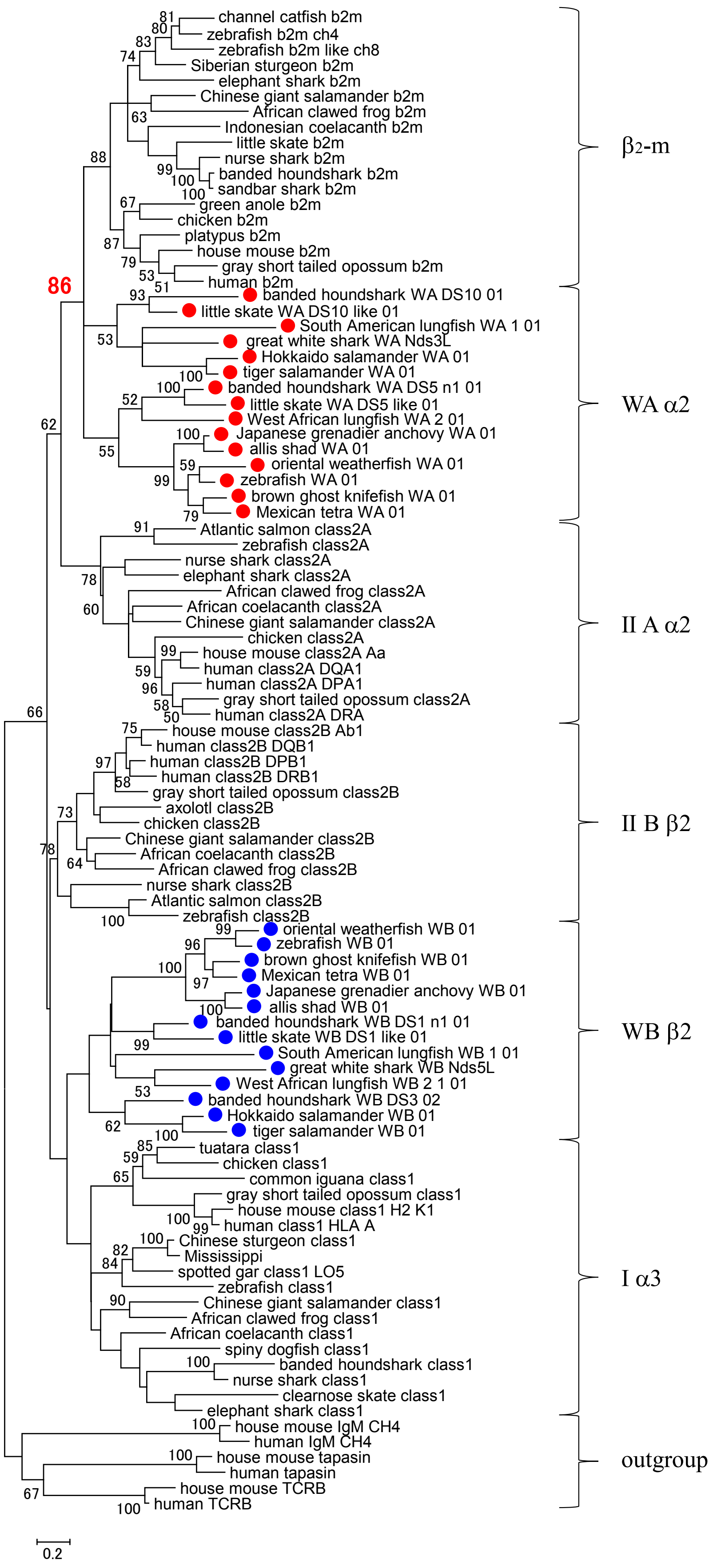

channel catfish b2m
zebrafish b2m ch4
zebrafish b2m like ch8
Siberian sturgeon b2m
elephant shark b2m
Chinese giant salamander b2m
African clawed frog b2m
Indonesian coelacanth b2m
little skate b2m
nurse shark b2m
banded houndshark b2m
sandbar shark b2m
green anole b2m
chicken b2m
platypus b2m
house mouse b2m
gray short tailed opossum b2m
human b2m
86
banded houndshark WA DS10 01
little skate WA DS10 like 01
South American lungfish WA 1 01
great white shark WA Nds3L
Hokkaido salamander WA 01
tiger salamander WA 01
banded houndshark WA DS5 n1 01
little skate WA DS5 like 01
West African lungfish WA 2 01
Japanese grenadier anchovy WA 01
allis shad WA 01
oriental weatherfish WA 01
zebrafish WA 01
brown ghost knifefish WA 01
Mexican tetra WA 01
Atlantic salmon class2A
zebrafish class2A
nurse shark class2A
elephant shark class2A
African clawed frog class2A
African coelacanth class2A
Chinese giant salamander class2A
chicken class2A
house mouse class2A Aa
human class2A DQA1
human class2A DPA1
gray short tailed opossum class2A
human class2A DRA
house mouse class2B Ab1
human class2B DQB1
human class2B DPB1
human class2B DRB1
gray short tailed opossum class2B
axolotl class2B
chicken class2B
Chinese giant salamander class2B
African coelacanth class2B
African clawed frog class2B
nurse shark class2B
Atlantic salmon class2B
zebrafish class2B
oriental weatherfish WB 01
zebrafish WB 01
brown ghost knifefish WB 01
Mexican tetra WB 01
Japanese grenadier anchovy WB 01
allis shad WB 01
banded houndshark WB DS1 n1 01
little skate WB DS1 like 01
South American lungfish WB 1 01
great white shark WB Nds5L
West African lungfish WB 2 1 01
banded houndshark WB DS3 02
Hokkaido salamander WB 01
tiger salamander WB 01
tuatara class1
chicken class1
common iguana class1
gray short tailed opossum class1
house mouse class1 H2 K1
human class1 HLA A
Chinese sturgeon class1
Mississippi
spotted gar class1 LO5
zebrafish class1
Chinese giant salamander class1
African clawed frog class1
African coelacanth class1
spiny dogfish class1
banded houndshark class1
nurse shark class1
clearnose skate class1
elephant shark class1
house mouse IgM CH4
human IgM CH4
house mouse tapasin
human tapasin
house mouse TCRB
human TCRB
0.2
β2-m
WA α2
II A α2
II B β2
WB β2
I α3
outgroup

Fig. S16. Phylogenetic tree analysis of W-category and MHC class I and class II molecules using DNA sequences. The phylogenetic tree was constructed with the DNA sequences of the membrane-proximal Ig-like C1-set domains of selected MHC molecules using Maximum Likelihood. The percentage of trees (bootstrap value, 50 or greater) in which respective sequences clustered together is shown next to the nodes. The bootstrap value at the WAα2/β_2-m node is shown in red. A more detailed explanation is described in the Materials and Methods. The alignment used for this analysis is based on the corresponding amino acid sequence alignment shown in Dataset S2. Red dots mark WA α2 sequences and blue dots mark WB β2 sequences.

Fig. S17

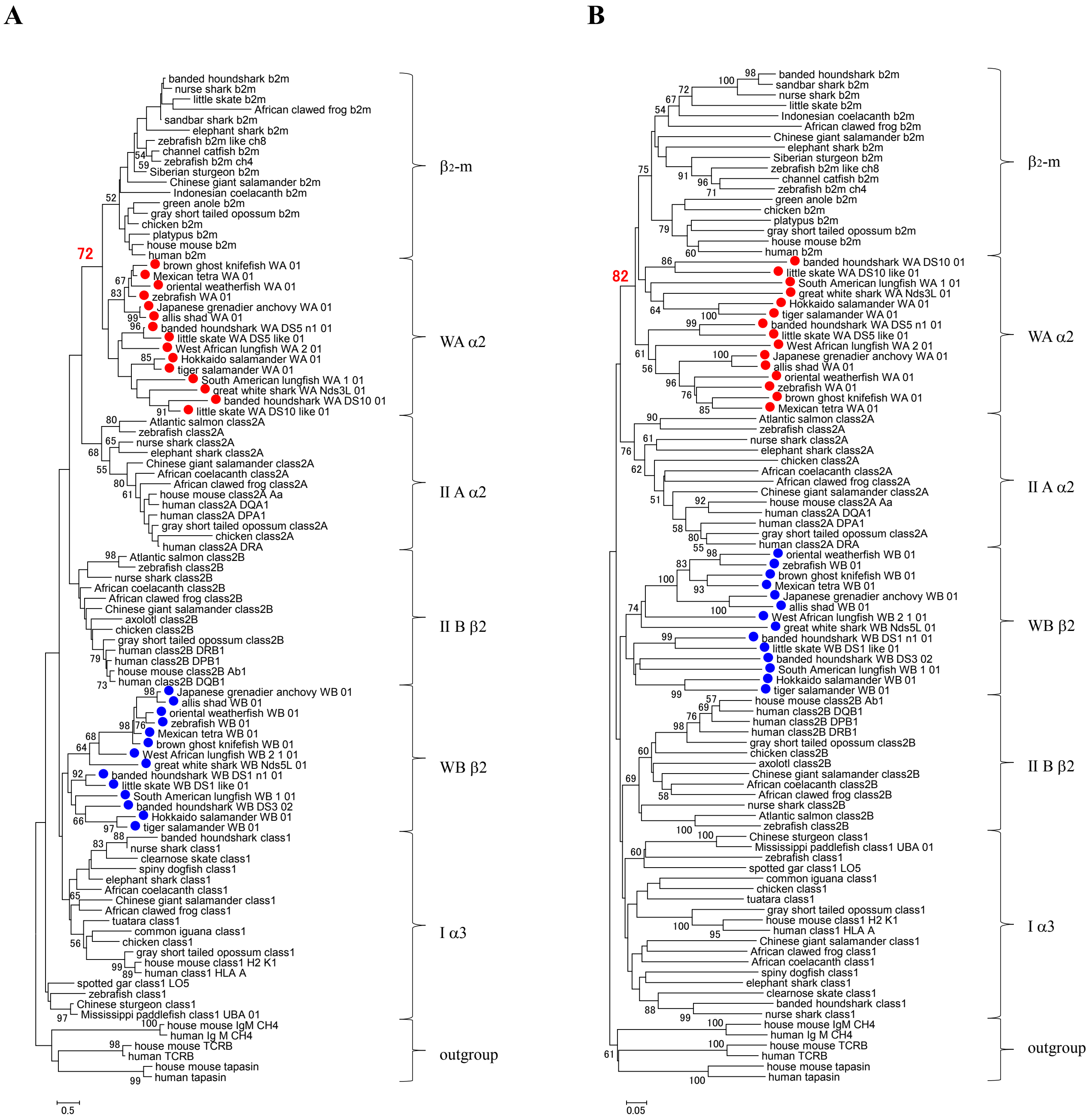

Fig. S17. Phylogenetic tree analyses of W-category and MHC class I and class II molecules using the Neighbor Joining method. The phylogenetic tree was constructed with membrane-proximal Ig-like C1-set domains of selected MHC molecules using the Neighbor Joining method with JTT model (**A**) or with p-distance model (**B**). The percentage of trees (bootstrap value, 50 or greater) in which respective sequences clustered together is shown next to the nodes. The bootstrap values at the WAα2/β_2-m node are shown in red. A more detailed explanation is described in the Materials and Methods. The alignment used for this analysis is shown in Dataset S2. Red dots mark WA α2 sequences and blue dots mark WB β2 sequences.

Fig. S18

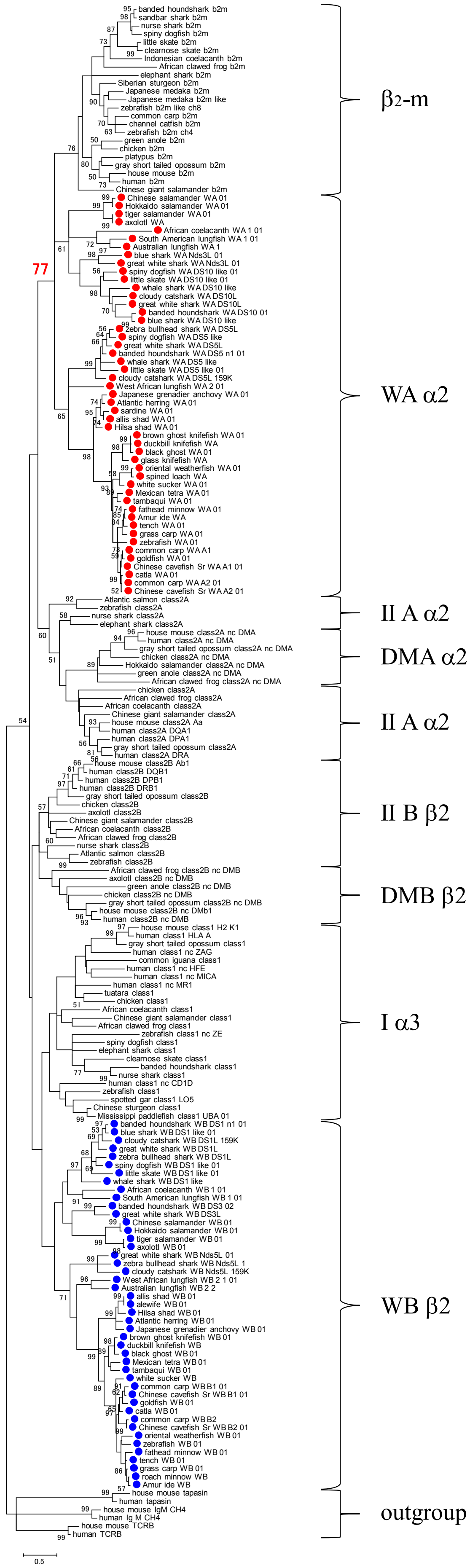
β2-m
WA α2
II A α2
DMA α2
II A α2
II B β2
DMB β2
I α3
WB β2
outgroup
77
0.5

Fig. S18. Phylogenetic tree analysis of W-category and MHC class I and class II molecules including nonclassical molecules. The phylogenetic tree was constructed with membrane-proximal Ig-like C1-set domains of selected MHC molecules using Maximum Likelihood. Several nonclassical class I and class II molecules are included in this analysis. The percentage of trees (bootstrap value, 50 or greater) in which respective sequences clustered together is shown next to the nodes. The bootstrap value at the WAα2/β_2-m node is shown in red. A more detailed explanation is described in the Materials and Methods. The alignment used for this analysis is shown in Dataset S2. Red dots mark WA α2 sequences and blue dots mark WB β2 sequences.

A Zebrafish WA

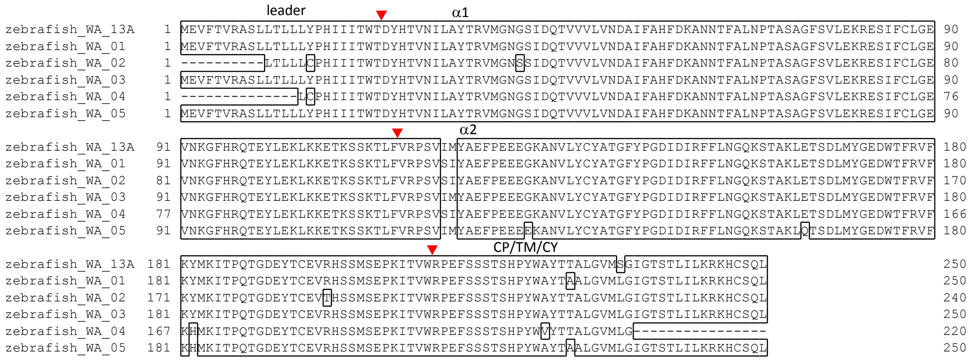

B Zebrafish WB

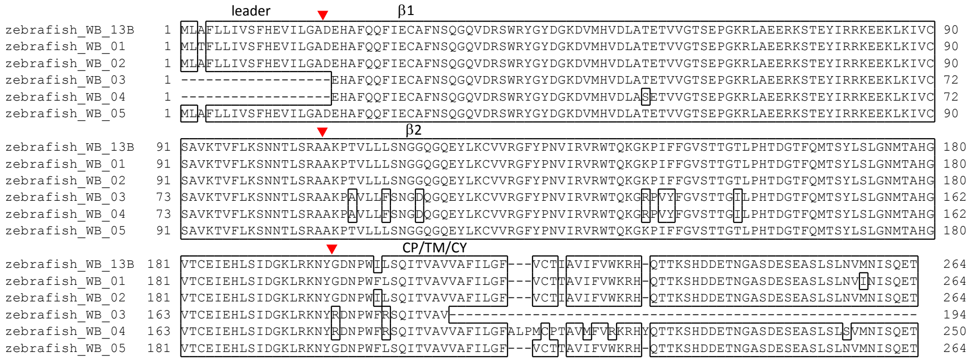

C

strain	individual	WA		WB	
AB	AB-1	WA-01		WB-01	
	AB-2	WA-01		WB-01	
IND	IND-1	WA-01		WB-01	
	IND-2	WA-01	WA-02	WB-01	
TL	TL-1	WA-03	WA-04	WB-02	WB-03
	TL-2	WA-04		WB-04	
WIK	WIK-1	WA-05		WB-05	
	WIK-2	WA-05		WB-05	

Fig. S19. Variations in W-category molecules observed for zebrafish.
A, Zebrafish WA sequences. **B**, Zebrafish WB sequences. Sequences labeled 01~05 were identified in individuals of four different zebrafish strains (AB, IND, TL and WIK) as summarized in C. WA_13A and WB_13B are taken from zebrafish genomic sequences. The primer sequences used to amplify zebrafish W-category cDNA are listed in Table S2. Red arrowheads indicate the corresponding positions of exon/intron borders in the genome. For some sequences, the amino and/or carboxyl terminal side(s) are not available because of the primer positions chosen. **C**, Distribution of zebrafish W-category sequences among various strains. One or two sequences for WA and for WB per individual could be identified using our procedure.

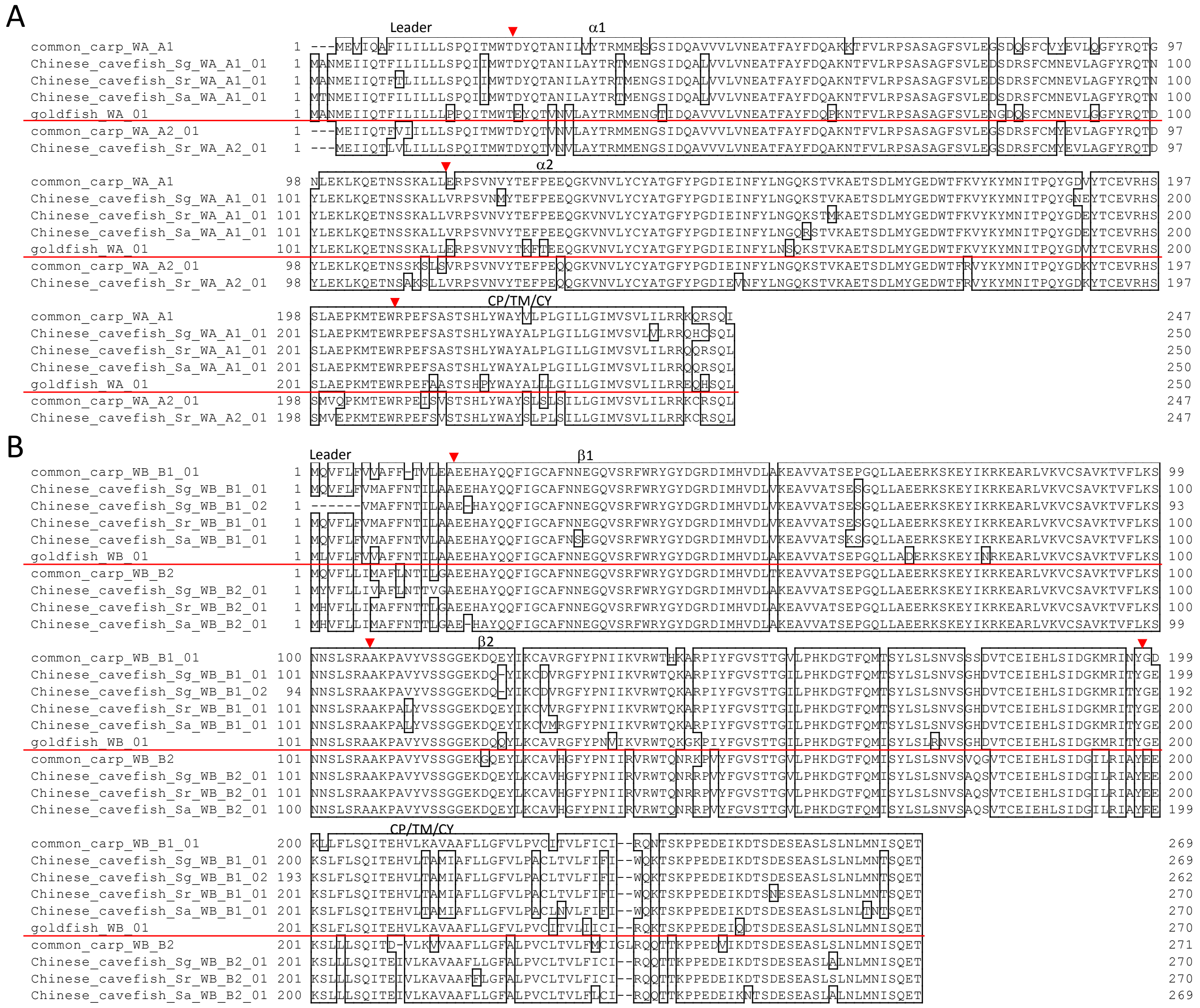

Fig. S20. Variations in W-category molecules of Chinese cavefish in comparison with common carp and goldfish molecules.

A, WA_A1 and WA_A2 sequences of different Chinese cavefish species, common carp and goldfish. A1 and A2 groups are separated with a red line. **B**, WB_B1 and WB_B2 sequences of Chinese cavefish, common carp and goldfish. B1 and B2 groups are separated with a red line. Goldfish WA_01 and WB_01 are also included in A and B, respectively. Goldfish WA_01 and WB_01 are similar to WA_A1 and WB_B1 of the other compared fishes, respectively. Chinese cavefish Sg = *Sinocyclocheilus graham,* Sr = *Sinocyclocheilus rhinocerous,* Sa = *Sinocyclocheilus anshuiensis.* Red arrowheads indicate the positions of exon/intron borders in the genome.

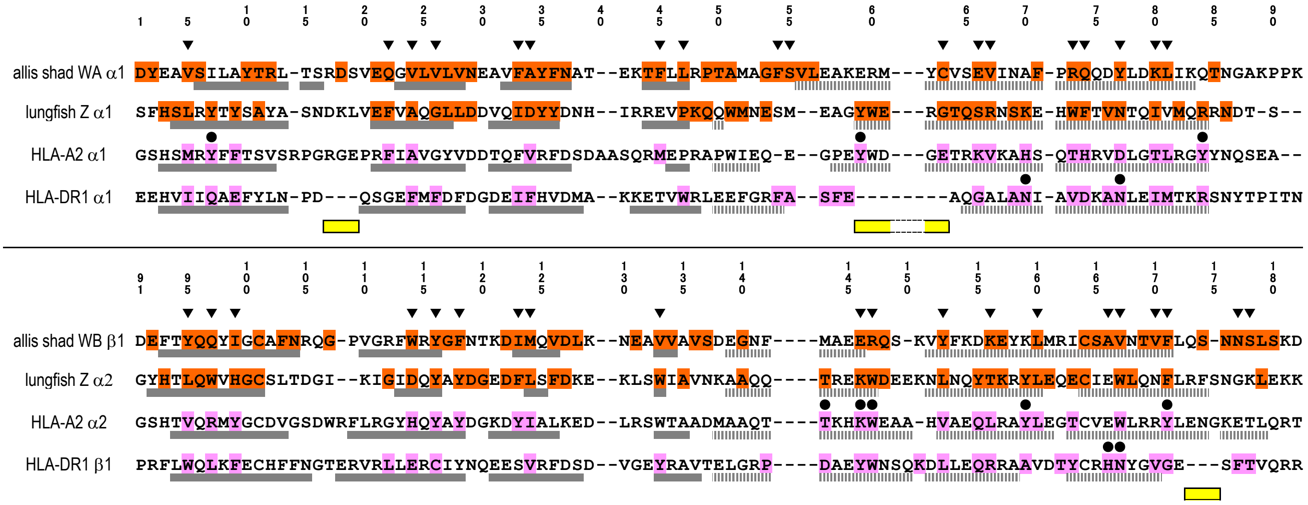

Fig. S21. Highly conserved positions in the membrane-distal domains of the teleost fish W-category molecules: similarity with those of MHC-Z molecules and with peptide-binding positions of the classical MHC molecules.

W-category amino acid residues of allis shad (*Alosa alosa*, Clupeidae, as a representative of the teleost fish) compared with lungfish MHC-Z molecule and with the classical MHC class I, HLA-A2 and the classical MHC class II, HLA-DR1. Orange-shaded residues of allis shad are invariable or highly conserved at the respective positions as verified among more than twenty teleost fish W-category sequences (Table S4). Orange-shaded residues of lungfish MHC-Z are invariable or highly conserved at the respective positions among MHC-Z molecules of lungfish and teleost fish (38). Magenta-shaded residues of HLA-A2 and of HLA-DR1 can interact with bound peptide antigens according to the references 29 and 31, respectively. Arrowheads indicate the amino acid positions where both the highly conserved teleost fish W-category amino acid positions and the peptide antigen binding positions of HLA-A2 and/or HLA-DR1 can be found. Large black dots above HLA-A2 sequence indicate amino acid residues highly conserved among the classical MHC class I molecules which interact with peptide termini of a bound peptide. Large black dots above HLA-DR1 sequence indicate amino acid residues highly conserved among the classical MHC class II molecules which interact with main chain residues of a bound peptide. Grey bars indicate positions of β-strands whereas dotted grey bars indicate positions of helical structures. For allis shad and lungfish molecules, these secondary structures were predicted as described in the Materials and Methods. Yellow boxes indicate gaps in class II molecules compared to class I molecules (Dataset S1). The residue numbers in this figure are based on those of HLA-A2 (29).

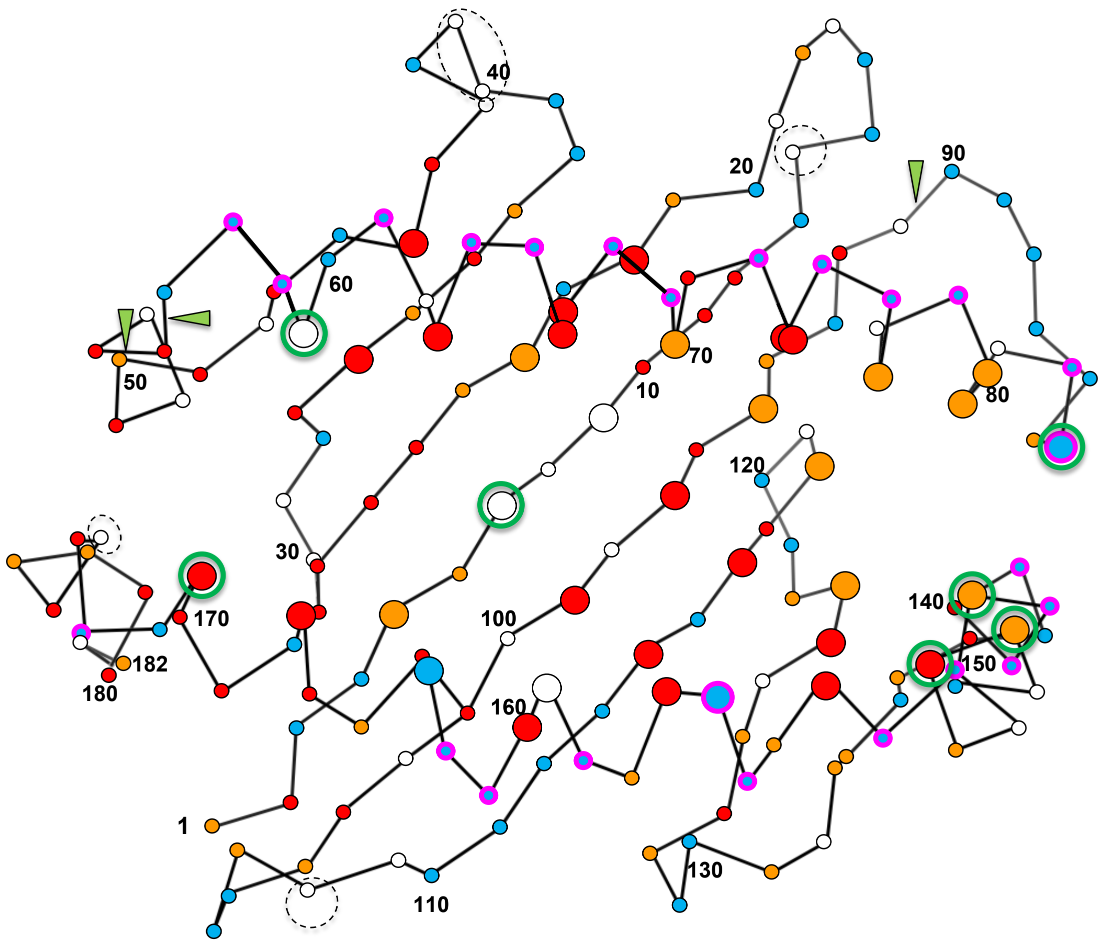

Fig. S22. Conservation profile in the hypothetical membrane-distal domains of the teleost fish W-category molecule based on the structure of HLA-A2: the highly conserved positions in the teleost fish W-category molecule largely correspond to the peptide-binding positions of HLA-A2.

Circles indicate positions of main chain Cα of HLA-A2. Large circles (total 37) indicate peptide-binding positions of HLA-A2. For the respective amino acid positions of teleost fish W-category molecules, Wu-Kabat variability was calculated (Table S4), and the obtained values are classified with various colors as follows: red, 1.0 (invariable); orange, 2.1 - 2.5 (relatively highly conserved); white, 2.5 < and </= 4; blue, 4 < (highly variable). The number of peptide-binding positions labeled with different colors are as follows: red, 19; orange, 11; white, 4; and blue, 3. In the α-helical regions, the positions with blue colors with especially high Wu-Kabat values (= or > 6) are labeled with magenta boundary lines, and many of them are positions on an α-helix that are away from the site including those toward TCR. In HLA-A2 molecule, three peptide-binding residues at positions 146 (orange), 155 (blue) and 163 (blue) are classified as residues with intermediate positions on an α-helix that point both into the peptide-binding site and away from the site (25). The eight positions with large green circles indicate the highly conserved amino acid positions of the classical MHC class I molecule, which interact with the N- or C-terminal of the bound peptide (the numbers of the positions are: Y7, Y59, (Y or R)84, T143, K146, W147, Y159, and Y171). In Fig. S21, these positions are shown with large black dots above the HLA-A2 sequence. The residues 1 to 90 correspond to WA α1 domain, and the residues 91 to 182 correspond to WB β1 domain. Dotted lines indicate presumed absence of residue(s) in case of teleost fish W-category molecules compared to HLA-A2. Green triangles indicate presumed locations of

the presence of additional position(s) of teleost fish W-category molecule compared to HLA-A2. The structure is based on HLA-A2 of Protein Data Bank ID: 3HLA (29).

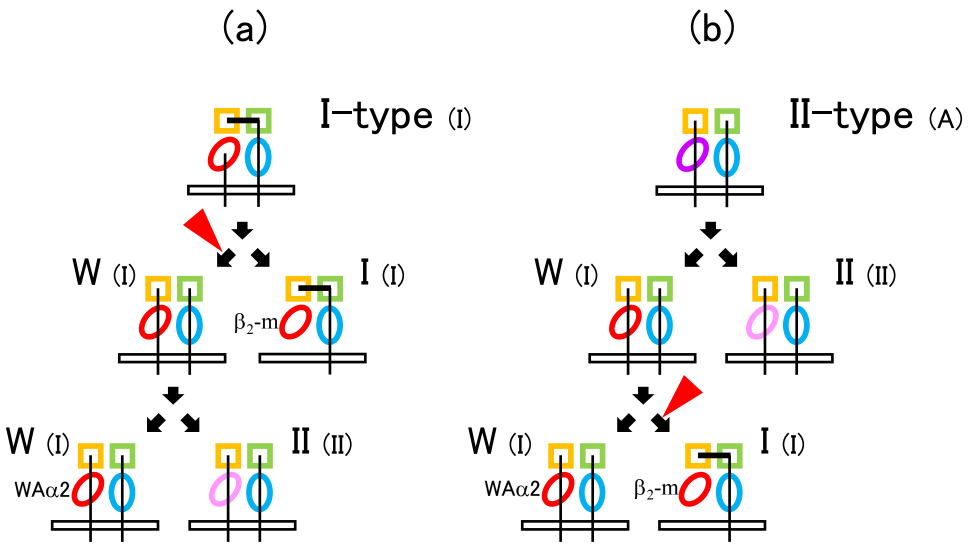

Fig. S23. Models for MHC class divergence with W-category as an intermediate.

(a) Class I-first model with W-category. The previous model (9) is modified with W-category as an intermediate. In this model, at the change from W (I) to II (II), inter-domain interfaces of W-category molecule should change into those of the conventional class II, including the loss of W61 of WAα2 (corresponding to IIAα2) and the acquisition of W61 of WBβ2 (corresponding to IIBβ2). Further, (F/L)37 of WAα2 should change back to W37 of IIAα2, which is highly conserved among many Ig superfamily members.

(b) Class II-first model with W-category. The previous model (7, 15) is modified with W-category as an intermediate. This is the same as Fig. 8C, but shown for comparison with (a).

Red arrowheads indicate the stages when alterations of domain architectures take place. The class I-type and the class II-type inter-domain interfaces are indicated by (I) and (II), respectively, and (A) indicates the ancestral state of the inter-domain interface.

Table S1

	Order	Family	animal	WA gene	WB gene	WA/WB linkage
Cartilaginous fish						
shark	Carcharhiniformes	Carcharhinidae	banded houndshark +	WA_DS5, WA_DS10	WB_DS1 WB_DS3	all linked
			blue shark	WA_DS5L # WA_DS10L WA_Nds3L	WB_DS1L WB_DS3L WB_Nds5L	
		Scyliorhinidae	cloudy catshark	WA_DS5L WA_DS10L	WB_DS1L WB_Nds5L	paired*
	Lamniformes	Lamnidae	great white shark	WA_DS5L WA_DS10L WA_Nds3L	WB_DS1L WB_DS3L WB_Nds5L	paired*
	Orectolobiformes	Rhincodontidae	whale shark	WA_DS5L WA_DS10L	WB_DS1L WB_DS3L	
		Hemiscylliidae	whitespotted bambooshark	WA_DS5L WA_DS10L WA_Nds3L	WB_DS1L WB_DS3L WB_Nds5L	
			brownbanded bambooshark	WA_DS5L WA_DS10L WA_Nds3L	WB_DS1L WB_DS3L WB_Nds5L	
	Heterodontiformes	Heterodontidae	zebra bullhead shark	WA_DS5L WA_DS10L	WB_DS1L WB_Nds5L	
	Squaliformes	Squalidae	spiny dogfish	WA_DS5L WA_DS10L WA_Nds3L	WB_DS1L WB_DS3L WB_Nds5L	
skate	Rajiformes	Rajidae	little skate	WA_DS5L WA_DS10L	WB_DS1L WB_Nds5L	
Ray-finned fish						
teleost fish	Clupeiformes	Engraulidae	Japanese grenadier anchovy	WA	WB	
		Clupeidae	Atlantic herring	WA	WB	paired
			sardine	WA	WB#	
			allis shad	WA	WB	
			alewife	WA	WB	
			Hilsa shad	WA	WB	paired
	Cypriniformes	Cobitidae	oriental weatherfish	WA	WB	
			spined loach	WA		
		Catostomidae	white sucker	WA	WB	
		Cyprinidae	common carp	WA_A1 WA_A2	WB_B1 WB_B2	paired*
			Chinese cavefish	WA_A1 WA_A2	WB_B1 WB_B2	paired*
			goldfish +	WA	WB	
			catla	WA	WB	
			tench	WA	WB	
			grass carp	WA	WB	
			fathead minnow	WA	WB	paired
			roach minnow	WA	WB	
			Amur ide	WA	WB	paired
			zebrafish +	WA	WB	paired
	Gymnotiformes	Apteronotidae	brown ghost knifefish	WA	WB	
			black ghost	WA	WB	
			duckbill knifefish	WA	WB	
		Sternopygidae	glass knifefish	WA		
	Characiformes	Characidae	Mexican tetra +	WA	WB	paired
		Serrasalmidae	tambaqui	WA	WB	
Lobe-finned fish						
coelacanth	Coelacanthiformes	Latimeriidae	African coelacanth	WA_1	WB_1	paired
			Indonesian coelacanth	WA_1#	WB_1#	
lungfish	Lepidosireniformes	Protopteridae	West African lungfish +	WA_1# WA_2	WB_1# WB_2_1 WB_2_2#	
			Slender lungfish	WA_1# WA_2#	WB_1# WB_2_1#	
		Lepidosirenidae	South American lungfish	WA_1	WB_1	
	Ceratodontiformes	Ceratodontidae	Australian lungfish	WA_1 WA_2#	WB_1# WB_2_1# WB_2_2	
Tetrapods						
salamander	Urodela (Caudata)	Hynobiidae	Chinese salamander	WA	WB	
			Hokkaido salamander	WA	WB	
		Ambystomatidae	tiger salamander +	WA	WB	
			axolotl	WA	WB	paired

Table S1. Summary of representative species from which W-category genes (*WA* and *WB*) were identified. In cloudy catshark, two *WA_DS5L* (*WA_DS5L_159K* and *WA_DS5L_82K*) and two *WB_Nds5L* (*WB_Nds5L_159K* and *WB_Nds5L_82K*) were identified. Plus (+) symbols following the animals' name indicate that the W-category genomic and/or cDNA sequences from the relevant animals were experimentally determined in the present study. Hash (#) symbols indicate that the gene fragments were identified in SRA (Sequence Read Archive; see *SI Appendix*). Asterisks (*) in the last column indicate that the *WA/WB* gene pairing situations are as follows: *WA_DS5L*/*WB_Nds5L* and *WA_DS10L*/*WB_DS1L* in cloudy catshark, *WA_DS5L*/*WB_Nds5L*, *WA_DS10L*/*WB_DS1L* and *WA_Nds3L*/*WB_DS3L* in great white shark, *WA_A1*/*WB_B1* and *WA_A2*/*WB_B2* in common carp and also in Chinese cavefish.

Table S2 Primer sequences: forward (F) / reverse (R)

animal	gene	F/R	name	purpose	location	primer sequence (5' --> 3')
banded houndshark	WA_DS5	F	DS5 5' UTR F1	cDNA	5' UTR	CATTCACGGAGCCTGAGAGATTCCAGTGAA
		R	DS5 3' UTR R1	cDNA	3' UTR	ATCAGCCAGAAACATCTGACAGGTCAAGAT
		F	DS5 5' UTR KpnI F1	genomic	5' UTR	ATGGGTACCCATTCACGGAGCCTGAGAGATTCCAGTGAA
		R	DS5 3' UTR SacI R1	genomic	3' part of exon 6, nc	ATGGAGCTCATCAGCCAGAAACATCTGACAGGTCAAGAT
		F	DS5-6	Southern blot	α2	TGTTACGCAGAGAAATTCTACCCT
		R	DS5-7	Southern blot	α2	CAGGCTGATATGATCCACTTGGCA
	WA_DS10	F	DS10-5	cDNA	5'UTR	GTCCGATCTCCACAGGAATG
		R	DS10-7	cDNA	3'UTR	GGATATTGCCTGGTCTCGAG
		F	DS10 5' UTR KpnI F1	genomic	5'UTR	ATGGGTACCGTCCCATCTCCACAGGAATGAGATTTCCTC
		R	DS10 3' UTR SacI R1	genomic	3'UTR	ATGGAGCTCAACTTTGGAGAGCGTGCAAAGAAAGTTCAC
		F	DS10-1	Southern blot	α2	TACGGTGAGGCAAACATTCTGAGC
		R	DS10-4	Southern blot	α2	GCAGGAATACATGTCCCCAGCTTC
		F	DS10-2	linkage, HhaI	α2	ACAATCACCATCACTCTTCAGGTC
		R	DS10-8	linkage, HhaI	3'UTR	AAGGTCTGGACCAATCCATT
		F	DS10-6	linkage, Amp	5'UTR	CAGGCTGGTTCCAGAAAGG
		R	DS10-14	linkage, Amp	α1	AAGAACCCCACGGTACTCCT
	WB_DS1	F	DS1-13	cDNA	5'UTR	AGGCAGTGAGAGGGTCTGAACC
		R	DS1-34	cDNA	3'UTR	GGTGCCTTCCAGTTTTGACGGT
		F	DS1-26	genomic, partial	β2	CCGTCAACATAACCACACAT
		R	DS1 3' UTR R1	genomic, partial	3'UTR	GAGGGAGCAGGTGCCTTCCAGTTTT
		F	DS1-48	Southern blot	β2	TGTCATGTGAATGGATTCTATCCA
		R	DS1-49	Southern blot	β2	GCTACTGCTGTGCTCCACTTGGCA
	WB_DS3	F	DS3-F1	cDNA	5' UTR	TCGCTTCCAACACAAACTAACTGCACCGTT
		R	DS3-R1	cDNA	3' UTR	CTCATTCGGAGAGTCAGTGAAGACATGACA
		F	DS3-16	genomic, partial	intron 4	CAGACGGCACTCAGTGACGTTAT
		R	DS3 intron 7 R	genomic, partial	intron 7	ATACTTGCACTTCAGGTGGGTGAACACAGT
		F	DS3-31	Southern blot	β2	TGCACAGTGTCCGGCTTCTACCCA
		R	DS3-32	Southern blot	β2	AAAGGTGACGTGCTCCACAAGGCA
	β_2-m	F	β2-m-F11	linkage	5' UTR	AGAAGTGCGGGAGGAGAGC
		R	β2-m-R10	linkage	3' UTR	AAAGAGAAAGGTTTTATTCACACTG
		F	β2-m-F12	Southern blot	5' of Ig-like domain	GTTCTCCAAATGTCCAAGTG
		R	β2-m-R11	Southern blot	3' of Ig-like domain	CAAGCTGAATCTCTCTTGAA
	β-actin	F	Trsc β-actin F1	cDNA, short	90-109 (AB084472)	ATGGATGATGAAATTGCAGC
		R	Trsc β-actin R1	cDNA, short	281-300 (AB084472)	TGGGGTACTTCAGGGTCAGG
		F	Trsc β-actin F2	cDNA, long	126-148 (AB084472)	GGATCTGGTATGTGCAAGGCTGG
		R	Trsc β-actin R2	cDNA, long	753-775 (AB084472)	GTGGCCATCTCCTGTTCAAAGTC
goldfish	WA	F	GF 13A 5' UTR F3	cDNA	5' UTR	GTTACTTTCTGTGAACCAAACTGCGTG
		R	GF 13A 3' UTR R2	cDNA	3' UTR	CCTCATCTATAAGCCACAATGTACTGC
	WB	F	GF 13B 5' UTR F2	cDNA	5' UTR	CTTTAAGTGTTGTGAATGTTGACAAAACAAA
		R	GF 13B 3' UTR R1	cDNA	3' UTR	ATGACAGGGTTTGGAGTCACACTC
zebrafish	WA	F	Zeb13A 5'UTR F1	cDNA	5' UTR	CACAACGGAACTAATTGTTCTTTATACAGC
		F	Zeb13A Lead F1	cDNA	leader	GTCCGAGCATCTCTGTTAACTCTTC
		R	Zeb13A TM R1	cDNA	TM	TTCAAAATCAGGGTAGATGTCCCGAT
		R	Zeb13A 3'UTR R1	cDNA	3' UTR	AAAACAGGACCCAGTATTGACCCT
	WB	F	Zeb13B 5'UTR F1	cDNA	5' UTR	GATCCGACGATAAAGCAAAGCATACGC
		F	Zeb13B Lead F1	cDNA	leader	ATGAAGTCATCCTTGGCGCAGAC
		R	Zeb13B TM R1	cDNA	TM	GTGCACACAAATCCAAGAATAAAAGCAAC
		R	Zeb13B 3'UTR R1	cDNA	3'UTR	ATAAAGTCTGTTTGTGCCCATAATCAG
Mexican tetra	WA	F	Mexican tetra WA lead F1	cDNA	leader	GCTTCTCCAGGTACCTGCTGT
		R	Mexican tetra WA TM R1	cDNA	TM	CACAAACAAGACCAGGTATCAGTC
	WB	F	Mexican tetra WB 5' UTR F1	cDNA	5' UTR	TTCCCCAGAGACGCTGAGTAAG
		R	Mexican tetra WB stop R1	cDNA	incl. stop codon	TCATATCTCTGATGGAAGATGCATCA
		R	Mexican tetra WB 3' UTR R1	cDNA	3' UTR	AACTGTAATTTACATAATCTGCAGTAATATG
West African lungfish	WA	F	Lungfish WA 5' UTR F1	cDNA	5' UTR	TGCTGCCAAGTCATATGTTCACATACTT
		F	Lungfish WA 5' UTR F2	cDNA	5' UTR	GCCAAGTCATATGTTCACATACTTTCTCTTTC
		R	Lungfish WA 3' UTR R1	cDNA	3' UTR	CTGCGGATGAATTCCTCTATGAAGATACTCTAG
		R	Lungfish WA 3' UTR R2	cDNA	3' UTR	GAGATATTCTGCTGCCTACATCTGCG
	WB	F	Lungfish WB 5' UTR F1	cDNA	5' UTR	CAGCTTTACTTGAGCATGCACGTTAC
		F	Lungfish WB start F1	cDNA	incl. start codon	CACAGCAGCATGCTTTGCTCATG
		R	Lungfish WB 3' UTR R1	cDNA	3' UTR	AGGAGTTCCTTCAAATTCAGAACACTGTAT
tiger salamander	WA	F	Sal-WA-F6	cDNA	5' UTR	GATGGACCGATAGAGTGGGAAGC
		R	Sal-WA-R6	cDNA	3' UTR	TGTACAACTGATAATGTGCAATTCTAGGAC
	WB	F	Sal-WB-F25	cDNA	5' UTR	TACTGAGCGCTGTGGTCTGCTACTC
		R	Sal-WB-R25	cDNA	3' UTR	ACATAGGAGTCATTGGAGATGAACA

Table S3 Accession numbers of the sequences in this study

Asterisks (∗) following accession numbers indicate the sequences which are clarified and registered in this study.

Classification	Animal species	Scientific name	Gene name	Accession No.	Information
WA	banded houndshark	*Triakis scyllium*	WA_DS5_n1_01	AB910515 ∗	individual N1
			WA_DS5_n2_01	AB910516 ∗	individual N1
			WA_DS5_n3_01	AB910517 ∗	individual N1
			WA_DS5_n4_01	AB910518 ∗	individual N1
			WA_DS5	LC200977 ∗	individual N0, genomic, α2
			WA_DS5_n3	LC009545 ∗	individual N1, genomic
			WA_DS10_01	LC196163 ∗	individual N1
			WA_DS10_02	LC196164 ∗	individual N1
			WA_DS10	LC009546 ∗	individual N1, genomic
			WA_DS10/WB_DS1_n2	LC218721 ∗	individual N1
	blue shark	*Prionace glauca*	WA_DS10-like	GFYY01081745	
			WA_DS10-like	GFYY01081744	
			WA_Nds3L	GFYY01021401	
	cloudy catshark	*Scyliorhinus torazame*	WA_DS5-like	BFAA01007584	159 K contig
			WA_DS5-like	BFAA01011934	82 K contig
			WA_DS10-like	BFAA01007584	159 K contig
	great white shark	*Carcharodon carcharias*	WA_DS5-like	QUOW01001706	285 K contig
			WA_DS10-like	QUOW01001706	285 K contig
			WA_Nds3L	QUOW01001706	285 K contig
	whale shark	*Rhincodon typus*	WA_DS5-like	LVEK01588988	α1
			WA_DS5-like	LVEK01625365	α2
			WA_DS10-like	LVEK01757095	α1
			WA_DS10-like	LVEK01573532	α2
	whitespotted bambooshark	*Chiloscyllium plagiosum*	WA_DS5-like	QPFF01095956	α1, α2
			WA_DS5-like	QPFF01329347	α2
			WA_DS5-like	BEZZ01383420	α1
			WA_DS10-like	QPFF01486458	α1, α2
			WA_Nds3L	QPFF01560724	α2
	brownbanded bambooshark	*Chiloscyllium punctatum*	WA_DS5-like	BEZZ01010960	α1
			WA_DS5-like	BEZZ01017577	α1
			WA_DS5-like	BEZZ01115237	α1
			WA_DS10-like	BEZZ01254771	α1
			WA_DS10-like	BEZZ01004761	α2, contains WB_DS3-like
			WA_Nds3L	BEZZ01099794	α2
	zebra bullhead shark	*Heterodontus zebra*	WA_DS5-like	GGGL01449115	contains WA_DS10-like
			WA_DS5-like	GGGL01449117	
			WA_DS5-like	GGGL01449119	contains WA_DS10-like
			WA_DS5-like	GGGL01449123	
			WA_DS5-like	GGGL01449125	
			WA_DS5-like	GGGL01449130	contains WA_DS10-like
			WA_DS5-like	GGGL01449131	contains WA_DS10-like
			WA_DS5-like	GGGL01449132	contains WA_DS10-like
			WA_DS5-like	GGGL01449136	contains WA_DS10-like
			WA_DS5-like	GGGL01449137	
			WA_DS5-like	GGGL01449140	
			WA_DS5-like	GGGL01449145	contains WA_DS10-like
			WA_DS5-like	GGGL01449146	
			WA_DS10-like	GGGL01449113	
			WA_DS10-like	GGGL01449114	
			WA_DS10-like	GGGL01449115	contains WA_DS5-like
			WA_DS10-like	GGGL01449119	contains WA_DS5-like
			WA_DS10-like	GGGL01449130	contains WA_DS5-like
			WA_DS10-like	GGGL01449131	contains WA_DS5-like
			WA_DS10-like	GGGL01449132	contains WA_DS5-like
			WA_DS10-like	GGGL01449133	
			WA_DS10-like	GGGL01449135	
			WA_DS10-like	GGGL01449136	contains WA_DS5-like
			WA_DS10-like	GGGL01449145	contains WA_DS5-like
	spiny dogfish	*Squalus acanthias*	WA_DS5-like	HAGW01089906	Fig. S1
			WA_DS5-like	HAGW01089902	
			WA_DS5-like	HAGW01089904	
			WA_DS5-like	HAGW01089907	
			WA_DS5-like	HAGW01089908	
			WA_DS5-like	HAGW01089910	
			WA_DS5-like	HAGT01020445	
			WA_DS5-like	HAGT01100681	
			WA_DS5-like	HAGV01095271	
			WA_DS10-like	HAGT01122485	partial α1, α2, partial TM/CY
			WA_Nds3L	HAGT01004242	α2, short
	little skate	*Leucoraja erinacea*	WA_DS5-like	AESE012173146	α1
			WA_DS5-like	AESE011681578	α2
			WA_DS10-like	AESE011594527	α1
			WA_DS10-like	AESE011710435	α2
			WA_DS10-like	AESE012658604	CP/TM/CY
	Japanese grenadier anchovy	*Coilia nasus*	WA_01	GFON01059655	α1, partial α2
	Atlantic herring	*Clupea harengus*	WA_01	JZKK01005006	34 K contig
			WA_01	OOIJ01000439	344 K contig
	sardine	*Sardina pilchardus*	WA_01	GGSC01089055	
			WA	GGSC01089054	
			WA	GGSC01089060	
			WA	GGSC01089061	
			WA	GGSC01089058	α2
			WA	GGSC01089059	α2
	allis shad	*Alosa alosa*	WA_01	GETY01010370	
	alewife	*Alosa pseudoharengus*	WA	GFCK01040569	partial α2, CP/TM/CY
	Hilsa shad	*Tenualosa ilisha*	WA_01	QYSC01123695	325 K contig
	oriental weatherfish (Dojo)	*Misgurnus anguillicaudatus*	WA_01	GAAD01002023	partial
	spined loach	*Cobitis taenia*	WA	GGJF01002240	partial α1, α2, CP/TM/CY
	white sucker	*Catostomus commersonii*	WA_01	GECX01063527	
	common carp	*Cyprinus carpio*	WA_A1	LN590696	26 M contig Songpu strain
			WA_A1	LN598268	890 K contig Songpu strain
			WA_A1 α2 fragment	LN590685	20 M contig Songpu strain
			WA_A1	LHQP01015169	107 K contig European strain
			WA_A2	LN594648	138 K contig Songpu strain
			WA_A2_01	LHQP01028415	82 K contig European strain
	Chinese cavefish Sg	*Sinocyclocheilus grahami*	WA_A1_01	XM_016288790	
			WA_A1	LCYQ01031371	102 K contig
			WA_A1	NW_015505413	2.6 M contig
			WA_A2	LCYQ01012883	29 K
			WA_A2	LCYQ01125997	2 K

	Chinese cavefish Sr	*Sinocyclocheilus rhinocerous*	WA_A1_01	XM_016571073	
			WA_A1	LAVF01079966	64 K
			WA_A1	LAVF01079967	37 K
			WA_A1	NW_015641068	124 K contig
			WA_A2_01	XM_016544921	
			WA_A2	LAVF01191476	50 K
			WA_A2	NW_015666971	1.2 M contig
	Chinese cavefish Sa	*Sinocyclocheilus anshuiensis*	WA_A1_01	XM_016474588	
			WA_A1	LAVE01180287	36 K
			WA_A1	NW_015557379	3.3 M contig
			WA_A2	LAVE01000366	31 K
			WA_A2	LAVE01180285	36 K
			WA_A2	NW_015536465	413 K contig
	goldfish	*Carassius auratus*	WA_01	LC198640 *	
	catla	*Gibelion catla, Catla catla*	WA_01	GEAE01049160	
	tench	*Tinca tinca*	WA_01	GFZX01071246	
	grass carp	*Ctenopharyngodon idella*	WA_01	GEUQ01023638	
	fathead minnow	*Pimephales promelas*	WA_01	JNCD01006382	111 K contig
			WA_01	JNCE01056294	23 K contig
	roach minnow	*Rutilus rutilus*	WA_01	GEBE01046214	leader, α1, partial α2
			WA	GEBE01046216	partial α2, CP/TM/CY
	Amur ide	*Leuciscus waleckii*	WA_01	FLSR01004870	28 M contig
	zebrafish	*Danio rerio*	WA_13A	NM_001098262	
			WA_01	LC198642 *	
			WA_02	LC198643 *	
			WA_03	LC198644 *	
			WA_04	LC198645 *	
			WA_05	LC198646 *	
	brown ghost knifefish	*Apteronotus leptorhynchus*	WA_01	GBKR010337083	
	black ghost	*Apteronotus albifrons*	WA_01	GFID01018756	
	duckbill knifefish	*Parapteronotus hasemani*	WA	GGHK01230896	partial
			WA	GGHK01278630	partial
	glass knifefish	*Eigenmannia virescens*	WA_01	GGJF01002240	
	Mexican tetra	*Astyanax mexicanus*	WA_01	XM_007254575	
			WA_01	NW_006749376	2433 K contig
			(fibulin-7 region)	NW_006739147	455 K contig
	tambaqui	*Colossoma macropomum*	WA_01	GGHL01010311	
	African coelacanth	*Latimeria chalumnae*	WA_1_01	XM_006000942	
			WA1_01	NW_005819663	1088 K contig
			(RXRB region)	NW_005821442	322 K contig
	West African lungfish	*Protopterus annectens*	WA_2_01	LC198652 *	
	South American lungfish	*Lepidosiren paradoxa*	WA_1_01	GEHZ01032426	
	Chinese salamander	*Hynobius chinensis*	WA	GAQK01112360	leader, partial α1
			WA	GAQK01026695	partial α2, CP/TM/CY
	Hokkaido salamander	*Hynobius retardatus*	WA_01	LE079167.1	
	tiger salamander	*Ambystoma tigrinum*	WA_01	LC195223 *	
			WA_02	LC195224 *	
	axolotl	*Ambystoma mexicanum*	WA	GFZP01140229	α2, CP/TM/CY
			WA	PGSH01013846	partial, L, α2, CP/TM/CY, 5397 K
			WA	PGSH01122872	α1
WB	banded houndshark	*Triakis scyllium*	WB_DS1_n1_01	AB910510 *	individual N1, long
			WB_DS1_n1	AB910511 *	individual N1, short
			WB_DS1_n1_02	AB910512 *	individual N1, long
			WB_DS1_n1	AB910513 *	individual N1, short
			WB_DS1_n1_cDNA	AY227971	individual N2 (partial)
			WB_DS1_n1	LC009542 *	individual N1, genomic PCR
			WB_DS1_n1	M85291	individual N0, genomic, β2
			WB_DS1_n1	LC200978 *	individual N0, genomic, β2
			WB_DS1_n2	LC200979 *	individual N0, genomic, β2
			WB_DS3_01	LC196165 *	individual N2
			WB_DS3_02	AB910514 *	individual N1
			WB_DS3_genomic_1	LC009543 *	individual N0, genomic
			WB_DS3_genomic_2	LC009544 *	individual N0, genomic PCR
	blue shark	*Prionace glauca*	WB_DS1-like	GFYY01012278	
			WB_DS3-like	GFYY01033062	
			WB_Nds5L	GFYY01040305	
	cloudy catshark	*Scyliorhinus torazame*	WB_DS1-like	BFAA01007584	159 K contig
			WB_Nds5L	BFAA01007584	159 K contig
			WB_Nds5L	BFAA01011934	82 K contig
	great white shark	*Carcharodon carcharias*	WB_DS1-like	QUOW01001706	285 K contig
			WB_DS3-like	QUOW01001706	285 K contig
			WB_Nds5L	QUOW01001706	285 K contig
	whale shark	*Rhincodon typus*	WB_DS1-like	LVEK01262083	β2
			WB_DS3-like	LVEK01746520	β1
	whitespotted bambooshark	*Chiloscyllium plagiosum*	WB_DS1-like	QPFF01495858	β2
			WB_DS3-like	QPFF01327377	β1
			WB_DS3-like	QPFF01162766	β2
			WB_Nds5L	QPFF01130085	β1
			WB_Nds5L	QPFF01212593	β1
	brownbanded bambooshark	*Chiloscyllium punctatum*	WB_DS1-like	BEZZ01084999	β2
			WB_DS3-like	BEZZ01004761	β1 and β2, contains WA_DS10-like
			WB_Nds5L	BEZZ01198256	β1
			WB_Nds5L	BEZZ01164644	β1
			WB_Nds5L	BEZZ01050759	β1
			WB_Nds5L	BEZZ01176133	β2
			WB_Nds5L	BEZZ01162851	β2
	zebra bullhead shark	*Heterodontus zebra*	WB_DS1-like	GGGL01058879	
			WB_DS1-like	GGGL01058880	
			WB_DS1-like	GGGL01058881	
			WB_DS1-like	GGGL01058882	
			WB_DS1-like	GGGL01058883	
			WB_DS1-like	GGGL01058886	
			WB_DS1-like	GGGL01058887	
			WB_Nds5L_1	GGGL01449143	
			WB_Nds5L_2	GGGL01449112	
			WB_Nds5L_3	GGGL01449138	
	spiny dogfish	*Squalus acanthias*	WB_DS1-like1	CV889148	
			WB_DS1-like2	CX663201	
			WB_DS1-like3	HAGW01089902	Fig. S2
			WB_DS1	HAGW01089904	
			WB_DS1	HAGW01089906	
			WB_DS1	HAGW01089907	
			WB_DS1	HAGW01089908	
			WB_DS1	HAGW01089910	
			WB_DS3-like1	CX196769	Fig. S2
			WB_DS3-like2	ES324258	
			WB_DS3-like3	ES883357	

	little skate	*Leucoraja erinacea*	WB_DS1-like	LTC44319	partial β1
			WB_DS1-like	AESE011933439	β2
			WB_Nds5L	AESE011737676	β2, partial
	Atlantic herring	*Clupea harengus*	WB_01	JZKK01005006	34 K contig
			WB_01	OOIJ01000439	344 K contig
	allis shad	*Alosa alosa*	WB_01	GETY01036995	
			WB_01	GETY01018251	
	Hilsa shad	*Tenualosa ilisha*	WB_01	QYSC01123695	325 K contig
	oriental weatherfish (Dojo)	*Misgurnus anguillicaudatus*	WB	GAAD01004277	partial
	white sucker	*Catostomus commersonii*	WB_01	GECX01114064	partial
	common carp	*Cyprinus carpio*	WB_B1	LN590696	26 M contig Songpu strain
			WB_B1	LN598268	890 K contig Songpu strain
			WB_B1_01	LHQP01015169	107 K contig European strain
			WB_B2	LN594648	138 K contig Songpu strain
			WB_B2	LHQP01028415	82 K contig European strain
	Chinese cavefish Sg	*Sinocyclocheilus grahami*	WB_B1_01	XM_016288788	
			WB_B1	XM_016288789	
			WB_B1	LCYQ01031371	102 K contig
			WB_B1	NW_015505413	2.6 M contig
			WB_B2_01	XM_016263664	
			WB_B2	LCYQ01125997	2 K
	Chinese cavefish Sr	*Sinocyclocheilus rhinocerous*	WB_B1_01	XM_016571072	
			WB_B1	LAVF01079966	64 K
			WB_B1	NW_015641068	124 K contig
			WB_B2_01	XM_016544920	
			WB_B2	LAVF01191476	50 K
			WB_B2	NW_015666971	1.2 M contig
	Chinese cavefish Sa	*Sinocyclocheilus anshuiensis*	WB_B1_01	XM_016474614	
			WB_B1	LAVE01180285	36 K
			WB_B1	NW_015557379	3.3 M contig
			WB_B2_01	XM_016486958	
			WB_B2	LAVE01000366	31 K
			WB_B2	NW_015536465	413 K contig
	goldfish	*Carassius auratus*	WB_01	LC198641 *	
	catla	*Gibelion catla, Catla catla*	WB_01	GEAE01019557	
	tench	*Tinca tinca*	WB_01	GFZX01039606	
	grass carp	*Ctenopharyngodon idella*	WB_01	GBKA01001259	
			WB	GEUQ01052457	
	fathead minnow	*Pimephales promelas*	WB_01	JNCD01006382	111 K contig
			WB	JNCE01056294	23 K contig
	roach minnow	*Rutilus rutilus*	WB	GEBE01052260	β1, partial β2
			WB	GEBE01052261	partial β2, CP/TM
	Amur ide	*Leuciscus waleckii*	WB_01	FLSR01004870	28 M contig
	zebrafish	*Danio rerio*	WB_13B	XM_001342488	genomic
			WB_01	LC198647 *	
			WB_02	LC198648 *	
			WB_03	LC198649 *	partial
			WB_04	LC198650 *	partial
			WB_05	LC198651 *	
	brown ghost knifefish	*Apteronotus leptorhynchus*	WB_01	GBKR010051454	
	black ghost	*Apteronotus albifrons*	WB_01	GFID01018754	
	duckbill knifefish	*Parapteronotus hasemani*	WB	GGHK01160239	partial
	Mexican tetra	*Astyanax mexicanus*	WB_01	XM_007254556	
			WB	NW_006749376	2433 K contig
	tambaqui	*Colossoma macropomum*	WB_01	GGHL01049603	
	African coelacanth	*Latimeria chalumnae*	WB_1_01	XM_014491414	partial
			WB_1	NW_005819663	1088 K contig, except leader
			WB_1	BAHO01266725	28 K, leader
	West African lungfish	*Protopterus annectens*	WB_2_1_01	LC198653 *	
	South American lungfish	*Lepidosiren paradoxa*	WB_1_01	GEHZ01040013	
	Chinese salamander	*Hynobius chinensis*	WB	GAQK01112360	leader, partial β1
			WB	GAQK01119707	partial β1, β2, CP/TM/CY
	Hokkaido salamander	*Hynobius retardatus*	WB_01	LE079819	
	tiger salamander	*Ambystoma tigrinum*	WB_01	LC195225 *	
			WB_02	LC195226 *	
	axolotl	*Ambystoma mexicanum*	WB_01	GFZP01045381	
			WB	PGSH01013846	partial, 5397 K contig
class IIA classical	nurse shark	*Ginglymostoma cirratum*	class IIA	M89950	
	elephant shark	*Callorhinchus milii*	class IIA	JX211142	
	Atlantic salmon	*Salmo salar*	class IIA	L77086	
	zebrafish	*Danio rerio*	class IIA	NM_001007205	
	African coelacanth	*Latimeria chalumnae*	class IIA	XM_006014225	
	Chinese giant salamander	*Andrias davidianus*	Anda-DAA*0101	KF611846	
	African clawed frog	*Xenopus laevis*	class IIA	AF454374	
	chicken	*Gallus gallus*	class IIA	AY357253	
	gray short-tailed opossum	*Monodelphis domestica*	class IIA	XM_001376714	
	house mouse	*Mus musculus*	H2-Aa	NM_010378	
	human	*Homo sapiens*	HLA-DPA1	NM_033554	
	human	*Homo sapiens*	HLA-DQA1	NM_002122	
	human	*Homo sapiens*	HLA-DRA	NM_019111	
	human	*Homo sapiens*	HLA-DRA	NG_002392	used in Fig. 5
DOA	house mouse	*Mus musculus*	H2-Oa	NM_008206	
	human	*Homo sapiens*	HLA-DOA	NM_002119	
class IIA nonclassical teleost	Atlantic salmon	*Salmo salar*	Sasa-DBA	EG757342	
	Atlantic salmon	*Salmo salar*	Sasa-DCA	DW549478	
	Atlantic salmon	*Salmo salar*	Sasa-DDA	DW557800	
	Atlantic salmon	*Salmo salar*	Sasa-DEA*0102	KC316032	
	Japanese medaka	*Oryzias latipes*	M5A	ENSORLG00000012794	
	Japanese medaka	*Oryzias latipes*	M16A	ENSORLP00000011499	
	stickleback	*Gasterosteus aculeatus*	GXVIIA	ENSGACG00000003731	
	tilapia	*Oreochromis niloticus*	O57A	ENSONIG00000002263	
	zebrafish	*Danio rerio*	D8.45A1	ENSDARG00000079593	
	zebrafish	*Danio rerio*	D8.46A	ENSDARG00000075932	
DMA	Hokkaido salamander	*Hynobius retardatus*	DMA-like	LE161217	
	African clawed frog	*Xenopus laevis*	DMA	BC061681	
	green anole	*Anolis carolinensis*	DMA	XM_008109234	
	chicken	*Gallus gallus*	BMA1	NM_001099353	
	gray short-tailed opossum	*Monodelphis domestica*	DMA	XM_007483660	
	house mouse	*Mus musculus*	H2-Dma	NM_010386	
	human	*Homo sapiens*	HLA-DMA	NM_006120	

class IIB classical	nurse shark	*Ginglymostoma cirratum*	class IIB	L20274	
	Atlantic salmon	*Salmo salar*	class IIB	X70166	
	zebrafish	*Danio rerio*	DAB	NM_131476	
	African coelacanth	*Latimeria chalumnae*	class IIB	XM_006010528	
	Chinese giant salamander	*Andrias davidianus*	Anda-DAB*0101	KF611873	
	axolotl	*Ambystoma mexicanum*	Amme-DAB_B1.021	AF209115	
	African clawed frog	*Xenopus laevis*	class IIB	D13688	
	chicken	*Gallus gallus*	BLB1	NM_001044694	
	gray short-tailed opossum	*Monodelphis domestica*	MODO-DAB1	NM_001032991	
	house mouse	*Mus musculus*	H2-Ab1	NM_207105	
	human	*Homo sapiens*	HLA-DPB1	NM_002121	
	human	*Homo sapiens*	HLA-DQB1	NM_002123	
	human	*Homo sapiens*	HLA-DRB1	NM_002124	
	human	*Homo sapiens*	HLA-DRB3	NG_002392	used in Fig. 5
DOB	house mouse	*Mus musculus*	H2-Ob	NM_010389	
	human	*Homo sapiens*	HLA-OB	NM_002120	
class IIB nonclassical teleost	Atlantic salmon	*Salmo salar*	Sasa-DBB	DY726096	
	Atlantic salmon	*Salmo salar*	Sasa-DCB*0103	KC316031	
	Atlantic salmon	*Salmo salar*	Sasa-DEB*0102	KC316036	
	Japanese medaka	*Oryzias latipes*	M5B1	ENSORLG00000012822	
	Japanese medaka	*Oryzias latipes*	M16B	ENSORLG00000009164	
	stickleback	*Gasterosteus aculeatus*	GXVIIB	ENSGACG00000003680	
	tilapia	*Oreochromis niloticus*	O57B	ENSONIG00000002259	
	swordtail fish	*Xiphophorus multilineatus*	DXB*04	AY671988	
	zebrafish	*Danio rerio*	D8_45B1	ENSDARG00000041705	
	zebrafish	*Danio rerio*	D8_45B2	ENSDARG00000088872	
DMB	axolotl	*Ambystoma mexicanum*	DMB-like	isotig117813	
	African clawed frog	*Xenopus laevis*	DMB	DQ268506	
	green anole	*Anolis carolinensis*	DMB	XM_008124369	
	chicken	*Gallus gallus*	DMB	NM_001135166	
	gray short-tailed opossum	*Monodelphis domestica*	DMB	XM_007483659	
	house mouse	*Mus musculus*	H2-DMb1	NM_010387	
	human	*Homo sapiens*	HLA-DMB	NM_002118	
class I classical	banded houndshark	*Triakis scyllium*	Trsc-UAA_101	AF034316	
	spiny dogfish	*Squalus acanthias*	class I classical type	AY150811	
	nurse shark	*Ginglymostoma cirratum*	class I classical	AF220063	
	clearnose skate	*Raja eglanteria*	class I classical type	KC335152	
	elephant shark	*Callorhinchus milii*	class I classical type	JX207562	
	Atlantic salmon	*Salmo salar*	UBA*0101	AF504019	
	Atlantic salmon	*Salmo salar*	UBA*0301	AF504022	
	common carp	*Cyprinus carpio*	Cyca-UA1*01	X91015	
	African coelacanth	*Latimeria chalumnae*	Lach-UA-01	U08043	used in Datasets S2 and S3
	African coelacanth	*Latimeria chalumnae*	class I classical type	XM_006013358	used in Dataset S1
	Chinese giant salamander	*Andrias davidianus*	Anda-UAA*0101	KF611820	
	African clawed frog	*Xenopus laevis*	Xela_UAA1f	L20733	
	tuatara	*Sphenodon punctatus*	Sppu-U*01	DQ145788	
	common iguana	*Iguana iguana*	Igig-UB*0101	EU604317	
	chicken	*Gallus gallus*	Gaga_BF12	M31012	
	gray short-tailed opossum	*Monodelphis domestica*	MODO_UB	NM_001079820	
	house mouse	*Mus musculus*	H2-K1	NM_001001892	
	human	*Homo sapiens*	HLA-A*0201	AY365426	
	human	*Homo sapiens*	HLA-A	NG_029217	used in Fig. 5
class I nonclassical	spiny dogfish	*Squalus acanthias*	class I nonclassical	AF515705	
	rainbow trout	*Oncorhynchus mykiss*	Onmy-UAA	AF091779	
	zebrafish	*Danio rerio*	ZE	NM_194425	
	marbled lungfish	*Protopterus aethiopicus*	ZE	AF206309	
	African coelacanth	*Latimeria chalumnae*	Lach_UB_01	U08034	
	axolotl	*Ambystoma mexicanum*	class I nonclassical	U83137	
	African clawed frog	*Xenopus laevis*	class I nonclassical	M58019	
	chicken	*Gallus gallus*	YF5	NM_001030675	
	gray short-tailed opossum	*Monodelphis domestica*	MR1	AB719956	
	human MR1	*Homo sapiens*	MR1	NM_001531	
	human HFE	*Homo sapiens*	HFE	U60319	
	human ZAG	*Homo sapiens*	AZGP1 (ZAG)	NM_001185	
	human MICA	*Homo sapiens*	MICA	NM_000247	
	common brushtail possum	*Trichosurus vulpecula*	FCGN	AF191647	
	human	*Homo sapiens*	FCGRT (FcRn)	NM_001136019	
	chicken	*Gallus gallus*	CD1.2	AY849320	
	northern brown bandicoot	*Isoodon macrourus*	CD1	DQ924533	
	human	*Homo sapiens*	CD1A	NM_001763	
	human	*Homo sapiens*	CD1D	NM_001766	
	human	*Homo sapiens*	HLA-E	NM_005516	
	human	*Homo sapiens*	HLA-F	NM_001098479	
	human	*Homo sapiens*	HLA-G	NM_002127	
β2-m	banded hounshark	*Triakis scyllium*	b2m	HQ630063	
	sandbar shark	*Carcharhinus plumbeus*	b2m	GQ865620	
	nurse shark	*Ginglymostoma cirratum*	b2m	HM625831	
	spiny dogfish	*Squalus acanthias*	b2m	CX197536	
	little skate	*Leucoraja erinacea*	b2m	DT045428	
	clearnose skate	*Raja eglanteria*	b2m	AF520476	
	elephant shark	*Callorhinchus milii*	b2m	JW878642	
	Siberian sturgeon	*Acipenser baerii*	b2m	AJ132766	
	common carp	*Cyprinus carpio*	b2m	L05536	
	zebrafish	*Danio rerio*	b2m	NM_131163	
	zebrafish	*Danio rerio*	b2m-like	NM_213126	
	channel catfish	*Ictalurus punctatus*	b2m	AF016041	
	Japanese medaka	*Oryzias latipes*	b2m	NM_001104660	
	Japanese medaka	*Oryzias latipes*	b2m-like	XM_004082991	
	Indonesian coelacanth	*Latimeria menadoensis*	b2m-like	GAPS01030276	
	Chinese giant salamander	*Andrias davidianus*	b2m	KF611890	
	African clawed frog	*Xenopus laevis*	b2m	AF217962	
	green anole	*Anolis carolinensis*	b2m	XM_003227482	
	chicken	*Gallus gallus*	b2m	AY989898	
	platypus	*Ornithorhynchus anatinus*	b2m	NM_001127618	
	gray short-tailed opossum	*Monodelphis domestica*	b2m	AY125947	
	house mouse	*Mus musculus*	b2m	NM_009735	
	human	*Homo sapiens*	b2m	AB021288	
	human	*Homo sapiens*	b2m	NG_012920	used in Fig. 5
IgM	house mouse	*Mus musculus*	IgM_CH4	4JVW_A	
	human	*Homo sapiens*	IgM_CH4	CAA47708	
TCRB	house mouse	*Mus musculus*	TCRB	AAA40199	
	human	*Homo sapiens*	TCRB	1OGA_E	
tapasin	house mouse	*Mus musculus*	tapasin	NM_001025313	
	human	*Homo sapiens*	tapasin	NM_003190	
Genome Information				Assembly	
	Mexican tetra	*Astyanax mexicanus*	genome	Astyanax_mexicanus-2.0	
	zebrafish	*Danio rerio*	genome	GRCz11	
	human	*Homo sapiens*	genome	GRCh38.p13	

Table S4

position	N	Nd	Nc	V
1	23	2	21	2.2
2	24	1	24	1
3	24	4	13	7.4
4	24	4	17	5.6
5	24	2	21	2.3
6	24	2	20	2.4
7	24	2	13	3.7
8	24	3	21	3.4
9	24	3	21	3.4
10	24	1	24	1
11	24	1	24	1
12	24	1	24	1
13	24	6	16	9
15	24	4	11	8.7
16	24	4	9	10.7
17	24	4	18	5.3
18	24	2	19	2.5
19	24	3	22	3.3
20	24	3	9	8
21	24	2	19	2.5
22	24	1	24	1
23	24	3	12	6
24	24	2	23	2.1
25	24	2	20	2.4
26	24	1	24	1
27	24	1	24	1
28	24	1	24	1
29	24	1	24	1
30	24	2	12	4
31	24	3	19	3.8
32	24	4	8	12
33	24	1	24	1
34	24	1	24	1
35	24	2	23	2.1
36	24	1	24	1
37	24	2	19	2.5
38	24	4	16	6
39	24	7	8	21
42	24	5	10	12
43	24	3	17	4.2
44	24	2	23	2.1
45	24	1	24	1
46	24	4	14	6.9
47	24	1	24	1
48	25	3	23	3.3
49	25	1	25	1
50	25	2	22	2.3
51	25	2	24	2.1
52	25	3	18	4.2
53	25	4	21	4.8
54a	24	1	24	1
54b	25	1	25	1
55a	25	1	25	1
55b	25	2	22	2.3
55c	25	1	25	1
56	25	4	21	4.8
57	25	8	6	33.3
58	25	7	11	15.9
59	25	3	19	3.9
60	25	3	13	5.8
61	25	5	12	10.4
62	25	3	20	3.8
63	25	1	25	1
64	25	3	10	7.5
65	25	7	8	21.9
66	25	2	24	2.1
67	25	1	25	1
68	25	4	14	7.1
69	25	8	9	22.2
70	25	3	20	3.8
71	25	2	24	2.1
72	26	4	16	6.5
73	26	1	26	1
74	26	1	26	1
75	26	6	8	19.5
76	26	5	11	11.8
77	26	2	25	2.1
78	26	3	23	3.4
79	26	4	12	8.7
80	26	2	24	2.2
81	26	2	25	2.1
82	26	3	18	4.3
83	26	5	14	9.3
84	26	5	15	8.7
85	26	2	21	2.5
86	26	6	15	10.4
87	24	5	13	9.2
88	26	4	14	7.4
89	26	4	20	5.2
90a	26	4	16	6.5
90b	26	4	14	7.4
90c	26	4	14	7.4

position	N	Nd	Nc	V
91	19	2	11	3.5
92	22	1	22	1
93	22	3	13	5.1
94	22	2	17	2.6
95	22	2	21	2.1
96	22	1	22	1
97	22	1	22	1
98	22	3	17	3.9
99	22	1	22	1
100	22	2	16	2.8
101	22	1	22	1
102	22	3	20	3.3
103	22	2	21	2.1
104	22	2	19	2.3
105	22	4	10	8.8
106	22	5	10	11
107	22	2	21	2.1
109	22	3	17	3.9
110	22	5	18	6.1
111	22	5	8	13.8
112	22	3	12	5.5
113	22	4	15	5.9
114	22	1	22	1
115	22	5	17	6.5
116	22	1	22	1
117	22	1	22	1
118	22	2	18	2.4
119	22	2	13	3.4
120	22	4	14	6.3
121	22	3	14	4.7
122	22	2	21	2.1
123	22	2	20	2.2
124	22	2	21	2.1
125	22	4	17	5.2
126	22	2	18	2.4
127	22	1	22	1
128	22	2	20	2.2
129	22	7	9	17.1
130	22	4	14	6.3
131	22	2	18	2.4
132	22	2	16	2.8
133	22	2	18	2.4
134	22	2	20	2.2
135	22	3	15	4.4
136	22	2	19	2.3
137	22	1	22	1
138	22	3	15	4.4
139	22	6	11	12
140	22	1	22	1
141	23	6	8	17.3
142	23	4	10	9.2
143	23	2	18	2.6
144	23	3	17	4.1
145	23	3	20	3.5
146	23	2	22	2.1
147	23	1	23	1
148	23	2	19	2.4
149	23	3	18	3.8
150b	23	6	13	10.6
151	23	7	15	10.7
152	23	1	23	1
153	23	2	18	2.6
154	23	5	17	6.8
155	23	6	13	10.6
156	23	1	23	1
157	23	2	20	2.3
158	23	7	10	16.1
159	23	2	15	3.1
160	23	1	23	1
161	23	7	7	23
162	23	4	14	6.6
163	23	3	16	4.3
164	23	1	23	1
165	23	2	22	2.1
166	23	1	23	1
167	23	1	23	1
168	23	4	13	7.1
169	23	2	22	2.1
170	23	1	23	1
171	23	1	23	1
172	23	4	18	5.1
173	23	4	13	7.1
174	23	1	23	1
176	23	1	23	1
177	23	2	19	2.4
178	23	2	21	2.2
179	23	1	23	1
180	23	1	23	1
181	23	3	19	3.6
182	23	3	20	3.5

Table S4. Conservation profile of the membrane-distal domains of W-category molecules in the teleost fish.

Variabilities of the WA $\alpha 1$ domain (left, positions 1-90c) and WB $\beta 1$ domain (right, positions 91-182) are shown. Wu-Kabat variability (V) is defined as the number of different amino acids at a given position (Nd) divided by the frequency of the most common amino acid at that position (Nc/N; Nc, the number of the most common amino acid at that position; N, the number of sequences studied at that position) as described in the Materials and Methods. The position numbers are based on those of the HLA-A2 molecule (29; Fig. S21). Position numbers with a, b or c are used where sequence length disparities are present between the HLA-A2 and teleost fish W-category molecules. The sequence alignment is based on Dataset S1. The values of Wu-Kabat variability are classified with various colors as follows: red, 1.0 (invariable); orange, 2.1 - 2.5(relatively highly conserved); white, 2.6 =/< and </= 4 (moderately conserved/variable); blue, 4 < (highly variable).

References

1. M. Nei, A. P. Rooney, Concerted and birth-and-death evolution of multigene families. *Annu. Rev. Genet.* **39**, 121-152 (2005).
2. J. Trowsdale, J. C. Knight, Major Histocompatibility Complex genomics and human disease. *Annu. Rev. Genomics Hum. Genet.* **14**, 301-323 (2013).
3. M. Wieczorek, E. T. Abualrous, J. Sticht, M. Alvaro-Benito, S. Stolzenberg, F. Noe, C. Freund, Major Histocompatibility Complex (MHC) Class I and MHC Class II proteins: Conformational plasticity in antigen presentation. *Front. Immunol.* **8**, 292 (2017).
4. J. F. Kaufman, C. Auffray, A. J. Korman, D. A. Shackelford, J. Strominger, The class II molecules of the human and murine major histocompatibility complex. *Cell* **36**, 1-13 (1984).
5. L. Hood, M. Kronnenberg, T. Hunkapiller, T cell antigen receptors and the immunoglobulin supergene family. *Cell* **40**, 225-229 (1985).
6. L. H. Martin, F. Calabi, C. Milstein, Isolation of CD1 genes: a family of major histocompatibility complex-related differentiation antigens. *Proc. Natl. Acad. Sci. USA* **83**, 9154-9158 (1986).
7. J. Kaufman, Vertebrates and the evolution of the Major Histocompatibility Complex (MHC) class I and class II molecules. *Verh. Dtsch. Zool. Ges.* **81**, 131-144 (1988).
8. D. A. Lawlor, J. Zemmour, P. D. Ennis, P. Parham, Evolution of class-I MHC genes and proteins: from natural selection to thymic selection. *Annu. Rev. Immunol.* **8**, 23-63 (1990).
9. M. F. Flajnik, C. Canel, J. Kramer, M. Kasahara, Which came first, MHC class I or class II? *Immunogenetics* **33**, 295-300 (1991).
10. A. L. Hughes, M. Nei, Evolutionary relationships of the classes of major histocompatibility complex genes. *Immunogenetics* **37**, 337-346 (1993).
11. J. Klein, C. O'hUigin, Composite origin of major histocompatibility complex genes. *Curr. Opin. Genet. Dev.* **3**, 923-930 (1993).
12. J. Klein, N. Nikolaidis, The descent of the antibody-based immune system by gradual evolution. *Proc.*

Natl. Acad. Sci. USA **102**, 169-174 (2005).

13. S. A. Porcelli, Bird genes give new insights into the origins of lipid antigen presentation. *Proc. Natl. Acad. Sci. USA.* **102**, 8399-8400 (2005).
14. M. F. Flajnik, L. Du Pasquier, "Evolution of the immune system." in *Fundamental Immunology* (Lippincott Williams & Wilkins, ed. 7, 2013).
15. J. Kaufman, Unfinished business: Evolution of the MHC and the adaptive immune system of jawed vertebrates. *Annu. Rev. Immunol.* **36**, 383-409 (2018).
16. K. Hashimoto, T. Nakanishi, Y. Kurosawa, Isolation of carp genes encoding major histocompatibility complex antigens. *Proc. Natl. Acad. Sci. USA.* **87**, 6863-6867 (1990).
17. K. Okamura, M. Ototake, T. Nakanishi, Y. Kurosawa, K. Hashimoto, The most primitive vertebrates with jaws possess highly polymorphic MHC class I genes comparable to those of humans. *Immunity* **7**, 777-790 (1997).
18. S. Bartl, M. A. Baish, M. F. Flajnik, Y. Ohta, Identification of class I genes in cartilaginous fish, the most ancient group of vertebrates displaying an adaptive immune response. *J. Immunol.* **159**, 6097-6104 (1997).
19. J. P. Cannon, R. N. Haire, G. W. Litman, Identification of diversified genes that contain immunoglobulin-like variable regions in a protochordate. *Nat. Immunol.* **3**, 1200-1207 (2002).
20. M. Kasahara, M. Vazquez, K. Sato, E. C. McKinney, M. F. Flajnik, Evolution of the major histocompatibility complex: isolation of class II A cDNA clones from the cartilaginous fish. *Proc. Natl. Acad. Sci. USA* **89**, 6688-6692 (1992).
21. S. Bartl, I. L. Weissman, Isolation and characterization of major histocompatibility complex class IIB genes from the nurse shark. *Proc. Natl. Acad. Sci. USA* **91**, 262-266 (1994).
22. A. F. Williams, A. N. Barclay, The immunoglobulin superfamily-domains for cell surface recognition. *Annu. Rev. Immunol.* **6**, 381-405 (1988).
23. K. Hashimoto, T. Nakanishi, Y. Kurosawa, Identification of a shark sequence resembling the major histocompatibility complex class I α3 domain. *Proc. Natl. Acad. Sci. USA.* **89**, 2209-2212 (1992).

24. P. Cosson, J. S. Bonifacino, Role of transmembrane domain interactions in the assembly of class II MHC molecules. *Science* **258**, 659-662 (1992).

25. P. J. Bjorkman et al., Structure of the human class I histocompatibility antigen, HLA-A2. *Nature* **329**, 506-512 (1987).

26. J. H. Brown et al., A hypothetical model of the foreign antigen binding site of class II histocompatibility molecules. *Nature* **332**, 845-850 (1988).

27. J. H. Brown et al., Three-dimensional structure of the human class II histocompatibility antigen HLA-DR1. *Nature* **364**, 33-39 (1993).

28. Y. Ohta et al., Primordial linkage of β_2-microglobulin to the MHC. *J. Immunol.* **186**, 3563-3571 (2011).

29. M. A. Saper, P. J. Bjorkman, D. C. Wiley, Refined structure of the human histocompatibility antigen HLA-A2 at 2.6 Å resolution. *J. Mol. Biol.* **219**, 277-319 (1991).

30. D. H. Fremont, M. Matsumura, E. A. Stura, P. A. Peterson, I. A. Wilson, Crystal structures of two viral peptides in complex with murine MHC class I H-2K^b. *Science* **257**, 919-927 (1992).

31. V. L. Murthy, L. J. Stern, The class II MHC protein HLA-DR1 in complex with an endogenous peptide: implications for the structural basis of the specificity of peptide binding. *Curr. Biol.* **5**, 1385-1396 (1997).

32. X.-L. Li, M.-K.Teng, E. L. Reinherz, J.-H. Wang, Strict major histocompatibility complex molecule class-specific binding by co-receptors enforces MHC-restricted αβ TCR recognition during T lineage subset commitment. *Front. Immunol.* **4**, 383 (2013).

33. Y. Ohta, K. Okamura, E.C. McKinney, S. Bartl, K. Hashimoto, M. F. Flajnik, Primitive synteny of vertebrate major histocompatibility complex class I and class II genes. *Proc. Natl. Acad. Sci. USA.* **97**, 4712-4717 (2000).

34. C. T. Amemiya et al., The African coelacanth genome provides insights into tetrapod evolution. *Nature* **496**, 311-316 (2013).

35. N. R. Saha et al., Genome complexity in the coelacanth is reflected in its adaptive immune system. *J. Exp. Zool. B Mol. Dev. Evol.* **322**, 438-463 (2014).

36. C. P. Kruiswijk, T. T. Hermsen, A. H. Westphal, H. F. Savelkoul, R. J. Stet, A novel functional class I

lineage in zebrafish (*Danio rerio*), carp (*Cyprinus carpio*), and large barbus (*Barbus intermedius*) showing an unusual conservation of the peptide binding domains. *J. Immunol.* **169**, 1936-1947 (2002).

37. A. Sato, H. Sultmann, W. E. Mayer, J. Klein, Mhc class I gene of African lungfish. *Immunogenetics* **51**, 491-495 (2000).

38. U. Grimholt, K. Tsukamoto, T. Azuma, J. Leong, B. F. Koop, J. M. Dijkstra, *BMC Evol. Biol.* **15**, 32 (2015).

39. Y. Inoue et al., Molecular cloning and preliminary expression analysis of banded dogfish (Triakis scyllia) CC chemokine cDNA by use of suppression subtractive hybridization. *Immunogenetics* **56**, 722-734 (2005).

40. S. F. Altschul et al., Gapped BLAST and PSI-BLAST: a new generation of protein database search programs. *Nucleic Acid Res.* **25**, 3389-3402 (1997).

41. A. Yates et al., Ensembl 2016 *Nucleic Acids Res.* **44**, D710-716 (2016).

42. J. Wyffels, B. L. King, J. Vincent, C. Chen, C. H. Wu, S. W. Polson, SkateBase, an elasmobranch genome project and collection of molecular resources for chondrichthyan fishes. *F1000Res.* **3**, 191 (2014).

43. N. W. Baddar, M. R. Woodcock, S. Khatri, D. K. Kump, S. R. Voss, Sal-Site: research resources for the Mexican axolotl. *Methods Mol. Biol.* **1290**, 321-336 (2015).

44. C. Burge, S. Karlin, Prediction of complete gene structures in human genomic DNA. *J. Mol. Biol.* 268, 78-94 (1997).

45. V. Solovyev, P. Kosarev, I. Seledsov, D. Vorobyev, Automatic annotation of eukaryotic genes, pseudogenes and promoters. *Genome Biol.* 7, Suppl 1: P.10.1-10.12. (2006).

46. T. N. Petersen, S. Brunak, G. von Heijne, H. Nielsen, SignalP 4.0: discriminating signal peptides from transmembrane regions. *Nat. Methods* 8, 785-786 (2011).

47. A. Drozdetskiy, C. Cole, J. Procter, G. J. Barton, JPred4: a protein secondary structure prediction server. *Nucleic Acids Res.* 43, W389-W394 (2015).

48. R. C. Edgar, MUSCLE: multiple sequence alignment with high accuracy and high throughput. *Nucleic Acids Res.* **32**, 1792-1797 (2004).

49. D. H. Fremont, F. Crawford, P. Marrack, W. A. Hendrickson, J. Kappler, Crystal structure of mouse H2-M. *Immunity* **9**, 385-393 (1998).

50. E. F. Pettersen et al., UCSF Chimera − a visualization system for exploratory research and analysis. *J Comput Chem.* **25**, 1605-1612 (2004).

51. K. Tamura, G. Stecher, D. Peterson, A. Filipski, S. Kumar, MEGA6: Molecular Evolutionary Genetics Analysis version 6.0. *Mol. Biol. Evol.* **30**, 2725-2729 (2013).

52. S. Whelan, N. Goldman, A general empirical model of protein evolution derived from multiple protein families using a maximum-likelihood approach. *Mol. Biol. Evol.* **18**, 691-699 (2001).

53. M. Nei, S. Kumar, *Molecular Evolution and Phylogenetics*, Oxford University Press, New York. (2000).

54. N. Saito, M. Nei, The neighbor-joining method: A new method for reconstructing phylogenetic trees. *Mol. Biol. Evol.* **4**, 406-425 (1987).

55. D. T. Jones, W. R. Taylor, J. M. Thornton, The rapid generation of mutation data matrices from protein sequences. *Computer Applications in the Biosciences* **8**, 275-282 (1992).

56. J. Felsenstein, Confidence limits on phylogenies: An approach using the bootstrap. *Evolution* **39**, 783-791 (1985).

57. B. Ortmann et al., A critical role for tapasin in the assembly and function of multimeric MHC class I-TAP complexes. *Science* **277**, 1306-1309 (1997).

58. Y. Wang, L. Y. Geer, C. Chappey, J. A. Kans, S. H. Bryant, Cn3D: sequence and structure views for Entrez. *Trends Biochem. Sci.* **25**, 300-302 (2000).

59. T. T. Wu, E. A. Kabat, An analysis of the sequences of the variable regions of Bence Jones proteins and myeloma light chains and their implications for antibody complementarity. *J. Exp. Med.* **132**, 211-250 (1970).

60. J. G. Inoue et al., Evolutionary origin and phylogeny of the modern Holocephalans (Chondrichthyes: Chimaeriformes): A mitogenomic perspective. *Mol. Biol. Evol.* **27**, 2576-2586 (2010).

61. M. Nakatani, M. Miya, K. Mabuchi, K. Saitoh, M. Nishida, Evolutionary history of Otophysi (Teleostei), a major clade of the modern freshwater fishes: Pangaean origin and Mesozoic radiation. *BMC Evol. Biol.* **11**, 177 (2011).

62. P. Xu et al., Genome sequence and genetic diversity of the common carp, *Cyprinus carpio*. *Nature genetics* **46**, 1212-1219 (2014).

63. C. V. Henkel et al., Comparison of the exomes of common carp (*Cyprinus carpio*) and zebrafish (*Danio rerio*). *Zebrafish* **9**, 59-67 (2012).

64. I. C. Kolder et al., A full-body transcriptome and proteome resource for the European common carp. *BMC Genomics* **17**, 701 (2016).

65. J. Yang et al., The *Sinocyclocheilus* cavefish genome provides insights into cave adaptation. *BMC Biol.* **14**, 1 (2016).

66. Y. Kuang et al., The genetic map of goldfish (*Carassius auratus*) provided insights to the divergent genome evolutions in the Cyprinidae family. *Sci. Rep.* **6**, 34849 (2016).

67. P. J. van den Elsen, A. Peijnenburg, M. C. J. A. van Eggermond, S. J. P. Gobin, Shared regulatory elements in the promoters of MHC class I and class II genes. *Immunology Today* **19**, 308-312, 1998.

68. K.-Q. Gao, N. H. Shubin, Late Jurassic salamandroid from western Liaoning, China. *Proc. Natl. Acad. Sci. USA.* **109**, 5767-5772 (2012).

69. W. Chen et al., Crystal structure of a bony fish β_2-microglobulin. *J. Biol. Chem.* **285**, 22505-22512 (2010).

70. T. Terado et al., Molecular cloning of C4 gene and identification of the class III complement region in the shark MHC. *J. Immunol.* **171**, 2461-2466 (2003).

71. P. J. Bjorkman, P. Parham, Structure, function, and diversity of class I major histocompatibility complex molecules. *Annu. Rev. Biochem.* **59**, 253-288 (1990).

72. J. K. Pullen, R. M. Horton, Z. Cai, L. R. Pease, Structural diversity of the classical H-2 genes: *K*, *D*, and *L*. *J. Immunol.* **148**, 953-967 (1992).

73. L. M. Amzel, R. J. Poljak, Three-dimensional structure of immunoglobulins. *Ann. Rev. Biochem.* **48**, 961-997 (1979).

74. K. C. Garcia et al., An αβ T cell receptor structure at 2.5 Å and its orientation in the TCR-MHC complex. *Science* **274**, 209-219 (1996).

75. M. Koch et al., Structures of an MHC class I molecule from B21 chickens illustrate promiscuous peptide binding. *Immunity* **27**, 885-899 (2007).

76. Z. Zeng et al., Crystal structure of mouse CD1: An MHC-like fold with a large hydrophobic binding groove. *Science* **277**, 339-345 (1997).

77. D. M. Zajonc, H. Striegl, C. C. Dascher, I. A. Wilson, The crystal structure of avian CD1 reveals a smaller, more primordial antigen-binding pocket compared to mammalian CD1. *Proc. Natl. Acad. Sci. USA.* **105**, 17925-17930 (2008).

78. W. P. Burmeister, L. N. Gastinel, N. E. Simister, M. L. Blum, P. J. Bjorkman, Crystal structure at 2.2 Å resolution of the MHC-related neonatal Fc receptor. *Nature* **372**, 336-343 (1994).

79. J. M. Dijkstra, U. Grimholt, J. Leong, B. F. Koop, K. Hashimoto, Comprehensive analysis of MHC class II genes in teleost fish genomes reveals dispensability of the peptide-loading DM system in a large part of vertebrates. *BMC Evol. Biol.* **13**, 260 (2013).

80. M. Durairaj, R. Sharma, J. C. Varghese, K. P. Kane, Requirement for Q226, but not multiple charged residues, in the class I MHC CD loop/D strand for TCR-activated CD8 accessory function. *Eur. J. Immunol.* **33**, 676-684 (2003).

81. R. Wang, K. Natarajan, D. H. Margulies, Structural basis of the CD8αβ/MHC class I interaction: focused recognition orients CD8β to a T cell proximal position. *J. Immunol.* **183**, 2554-2564 (2009).

82. Y. Li, Y. Yin, R. A. Mariuzza, Structural and biophysical insights into the role of CD4 and CD8 in T cell activation. *Frontiers Immunol.* **4**, 206, 1-11 (2013).

83. M. F. Flajnik, T. Tlapakova, M. F. Criscitiello, V. Krylov, Y. Ohta, Evolution of the B7 family: co-evolution of B7H6 and NKp30, identification of a new B7 family member, B7H7, and of B7's historical relationship with the MHC. *Immunogenetics* **64**, 571-590 (2012).

84. D. Hatherley, S. C. Graham, K. Harlos, D. I. Stuart, A. N. Barclay, Structure of signal-regulatory protein α. A link to antigen receptor evolution. *J. Biol. Chem.* **284**, 26613-26619 (2009).

85. J. Kaufman, J. Salomonsen, M. Flajnik, Evolutionary conservation of MHC class I and class II molecules – different yet the same. *Seminars in Immunology* **6**, 411-424 (1994).

86. R. F. S. Walters, W. F. DeGrado, Helix-packing motifs in membrane proteins. *Proc. Natl. Acad. Sci. USA.* **103**, 13658-13663 (2006).

87. L. –G. Lundin, D. Larhammar, F. Hallböök, Numerous groups of chromosomal regional paralogies strongly indicate two genome doublings at the root of the vertebrates. *J. Struc. Func. Genomics* **3**, 553-63 (2003).

88. M. F. Flajnik, M. Kasahara, Origin and evolution of the adaptive immune system: genetic events and selective pressures. *Nature Rev. Genet.* **11**, 47-59 (2010).

89. J. M. Dijkstra et al., A third broad lineage of major histocompatibility complex (MHC) class I in teleost fish; MHC class II linkage and processed genes. *Immunogenetics* **59**, 305-321 (2007).

90. L. A. Kelley et al., The Phyre2 web portal for protein modeling, prediction and analysis, *Nature protocols* **10**, 845-858 (2015).

91. A. Waterhouse et al., SWISS-MODEL: homology modelling of protein structures and complexes. *Nucleic Acids Res.* **46**, W296-W303 (2018).

Representative W-category sequences in FASTA format

>banded_houndshark_WA_DS5_n1_01
MELRNFLIIIAAVTAGTRAAIEFESVSEKLYYSYTPDSKRNIDIFSIDSIPFMVYDGEKMFVLNGNVT
DGIKELGQKEVTYYTERARGFSTRLQTITEEMIKLTNAKTIISKKPVVHIYTKEDYAPHHANTLYCY
AEKFYPFEIGVTFLVNGRPFAGLVNSSQLVVESDWTFNILKYIRIDPQDGDSYSCQVDHISLDKPLTV
SMDPPSPGPRSGIIVCAVGVITGVIGLLIGLYLVTTVCSRLGKPRSRQFRKE

>banded_houndshark_WA_DS5_n2_01
MELGNFLIIIAAVTAGTRAAIEFESISAMLYYSYTPDSKRNIDIFSIDSIPFMVYDYREKMFVLNGNVT
DGIKELGQKEVTYYTERARGFSTRLQTITEEMIKLTNAKTIISKKPVVHIYTKEDYAPHHANTLYCY
AEKFYPFEIGVTFLVNGRPFAGLVNSSQLVVELDWTFNILKYIRIDPQDGDSYSCQVDHISLDKPLTV
SMDPPSPGPRSGIIVCAVGVITGVIGLLIGLYLVTTVCSRLGKPRSRQFRKE

>banded_houndshark_WA_DS5_n3_01
MELRNFLIIIAAVTAGTRAAIEFESVSAMLYYSYTPDSKRNIDIFSIDSIPFMVYDYREKMFVLNGNV
TDGIKELGQKEVTYYTERARGFSTRLQTITEEMIKLTNAKTIISKKPVVHIYTKEDYAPHHANTLYC
YAEKFYPFEIGVTFLVNGRPFAGLVNSSQLVVELDWTFNILKYIRIDPQDGDSYSCQVDHISLDKPLT
VSMDPPSPGPRSGIIVCAVGVITGVIGLLIGLYLVTTVCSRLGKPRSRQFRKE

>banded_houndshark_WA_DS5_n4_01
MELRNFLIIIAAVTAGTRAAIEYKSVSERLYYSHTEDEETNGHIFTANAFPFLYYDNKKLKFVIHGQE
TDGIQELGQEEVTHFKERASGIQAGLQGIAKEITRLTKGSSVLKEKPVVHIYTEEDYAPHHANTLYC
YAEKFYPFEIGVTFLVNGRPFTGLVNSSQLVVESDWTFNILKYIRIDPQDGDSYSCQVDHISLDKPLT
VSMDPPSPGPRSGIIVCAVGVITGVIGLLIGLYLVTTVCSRLGKPRSRQFRKE

>banded_houndshark_WA_DS10_01
MSRESFLLLLLLLSPGTGLSATDTWRVLVACYITDDPNPLEMMCEVTADSRIFIYYDTSIHLLEGGLEE
YRGVLVELARDRNRAIASLRQTMALAAIATNFSSPPNEMADLLLFPEKEVTYGEANILSCLVSGAFP
PTITITLQVNGAPLHTDANSSRLWYGEDWRFQAVRRAHIRPEAGDMYSCQVQHTVSKEVKMVYW
EPEMPDSWEESILDSSQLGVLVGGVAVGALGMALGIGSCVWPKALSRNQSLLRIRSSLRSSSSV

>banded_houndshark_WB_DS1_n1_01
MLRSSVLLLLGLFQTAGTHITQFMLTFDNSQTPATTLSAAYDGTEFIQFNYSTNEFLATKPAAEPLV
QSLNANERLVERYVRYGSFAPLVAEAIIQAARRLIAKPSVNITTHRLHRGKDPLLLICHVNGFYPSGI
NATWLHNGGTIQQEVLSSRILPNTDGTFQTTLQISVTPQSRDTYTCQVEHSSSTDKLTATWAPKVKS
WPTHGYVAGIVIGVAGIIIALAGGIGRRRGFPACGSEAMPNQRCVNPDSSAADSGVWTQGEAEQLN
VENERNPSPAPAQD

>banded_houndshark_WB_DS1_n2_01
KPSVNITTHRLHRGKDPLLLICHVNGFYPSGINATWLHNGGTVQQEVLSSRILPNTDGTFQTTLQISV
TPQSRDTYTCQVEHSSSTDKLTATW

>banded_houndshark_WB_DS3_01
MPPARKVIPNGSILTVATFSCCFLSLISALDVVQQLLDCDQAAVTRKDISGCKWALAYNGHTLSYFD
PRADRLIVESSTVKSEVDSLNKDPKALKNIRNEIQDTVNFLSQLLEVGAGTLDRKVPPSVTISFIDLEA
SGHSSQLQCTVSGFYPRALNVSWLKDGRSTEQYVTQTPILPNNENTFQTTAYIKIEPSTVDTYTCLV
EHVTFPQGHRTDWVPRHRSSLSPAAIVGILFGIVGIITAVVGGVVRMKRQGHQSRMIEPKVLFQKCQ
ERRSNRSQTSMRSNASASSATSGDGLTKHTV

>banded_houndshark_WB_DS3_02

MPPARKVIPNGSILTVATFSCCFLSLISALDVVQQLLDCDQAAVTRKDISGCKWALAYNGHTLSYFD
PRADRLIVESSTVKSEVDSLNKDPKALKNIRNEIQDTVNFLSQLLEVGAGTLDRKVPPSVTISFIDLEA
SGHSSQLQCTVSGFYPRALNVSWLKDGRSTEQYVTQTPILPNNENTFQTTAYIKIEPSTVDTYTCLV
EHVTFPQGYRTDWVPRHRSSLSPAAIVGILFGIVGIITAVVGGVVRMKRQGHQSRMIEPKVLFQKCQ
ERRSNRSQSSMRSNASASSATSGDGLTKHTV

>blue_shark_WA_DS10-like
MKSHPGNSLLILLLTAGTGLPATDTWRVVVTCYVTDNPSPTEMMCELTADGRVLIYYDTSIHLLEG
NLEEYHGLLDELARDRNRTIASLRQTMALAAVATKFSSPPNEIADLLLFPEKEVTYGKANILSCLVS
GVFPPTITITLQVNGVPLHTDANSSRLWHGEDWRFQVVRRAHIQPEAGDMYSCQVLHTVSKEMKM
VYWEPEMPDSWEENILDSSQLGVLVGGVALGVLGMALGIGSCVWPKALNQNLSLLQMRSSLRSTS
SA

>blue_shark_WA_Nds3L_
MDTERKKNRIAFLRTLITAWVLLPATECGESRVYGTTYSTSAEQTSVFTFATSDMTFAYYNSSSPGF
EFLIKALYTLSNASQHLVQKYRNDLTILNKDILNEMVHRIPGKRTEVPTVLLYPTEPNILGRRTVLNC
FVFELFPPAAQVTFLKNDQPLVTKVNSSLLTFQTSWMFHVLNSVPIQPVPGDIYTCVVKLQNGEEIQ
VHWEAATSDTQRDAAQIAICTIGFLIGAVGMMIGLWLIFYKRGNTQVDSRDQRTVIVEERQ

>blue_shark_WB_DS1-like
MLRASVLLLLLLLRLFQTEGTHIIQLMITFDNSQTPATKLSAAYDGTEFIRFNYSTNQFLATQPAAESL
VQNLNANEKLVKRYVRYASFAPLLAETVIQVAHRLIAKPSVNITTHRLHREKDPLLLVCHVNGFYP
SGINATWLHNAGSVQQEVLSSRILPNTDGTFQTTLQISITPQSRDTYTCQVEHSSSTDKLTATWAPN
VKSWPTHGYVAGIVIGVAGIIIALAGGIGRYRGVWTQGEAE

>blue_shark_WB_DS3-like
FRLGGGFCLTQLLSLLLCRSQVGDFSSMPPVRNNGSILTVATFSCCFLSLISALDVVQQLLDCDEAA
VIHKNISSCKWALAYNGQTLSYFDTRVERLIVESSTVKSQVDSLNKDPKALKNIRNEIQDTVNFLSQ
LLQVGADTLDREVSPTVTISFIDQETTE

>cloudy_catshark_WA_DS5-like_82K
MELWSFLIMISSLTAVTRAEIGEYVESISARIYYRSAPDSTRNIYVFTINYIPFLLYDFETQKFVLNGK
MTEGLKELGQEEVIYYKAMARGLNVRLQKYTNEMIKLTSASSTITSKSLIYKKPLVHIYTKKIYAPH
QSNILFCYAENFYPFEIDITFLINGRPFTGLVNSSQLMIEMDWTFNILKYISIDPQDRDTYSCQVNHMS
LDEPMTVLMDQSYSQPHTGTIVCAVGVIAGALGLMVGLYLVTKLCSRQGKPCSRQFCK

>cloudy_catshark_WA_DS5-like_159K
MELWSFLIIISSLTAVTRAEIDVESISIYQYYGYTPDSENSLSSFAINAFPFMFMHHGLKNFIINGNVTN
GKNVLGEDEATFFREITRRFQLRLQRLTKELVKQTGTRIVHSKLLIYKKPVVHIYAEENYTPGQPNT
LYCYAEKFHPYEIEVTFLVNGRPFTGPSESSETMVAADWTFHVLKYIRIDPQEGDTYSCQVDHVSLD
KPMFVSM

>cloudy_catshark_WA_DS10-like_159K
MSRDSFLFFLLLCPGTGSSESDPWRVLVACFVTDNPSLQPLMCEVTVDDRMFIYYDSTLPSVQVLDS
GLEEYRWILAKLVSDRERTIASLRREMAFIATISNSSPPNDFGELFLYPESEVTYGEMNVLTCLVNGA
FPPTITVTLQMNGVPIDAGVNSSRLSFGEDWRFRVTRHAQIRPAAGDLYSCEVLHTISNEVKVVYW

>cloudy_catshark_WB_DS1-like_159K
MVRLLVLLVSGLLQIVDAHMIQFLFSYDRSHTPATNLSFAYDGTEVIRFNYTTNQFLATQPVAAPW
VQHLNDNERLVKRYVHYGSLAPIVGDAILRVARRLSAKPSINITAHHLPSEKDPLLLICRVDGFYPS

GFNATWLLNGDEVEQEVLSSSVLPNRDGTFQATLQVSITPRSGDAYTCQVEHSSSPDKLTATWAPK
VRSWPEHGYVSGITVGIVGIMIALAGGIGRWR

>cloudy_catshark_WB_Nds5L_82K
MSVLYRWVFVAVAATVASVLDLVDCSTEPHYFHMQCACVYGDNKPANLSSEMAYDGATILYYDS
AEKTFVAAQPIGQAEVNKRNKNINIVLSRIESLCDKIKETAIISNLTIEKMAPIPSKVLLQEKSGQMKL
VCLVKDFFPRDIKISWLRDGVVIVNAPQTVNIVPQDDKTFQARSLLTLNEDVSGSYICQVEHEALAR
KLQVPFRYDRITEHKSLIIIGAVLGILGISFAVVTGILYYCNLN

>cloudy_catshark_WB_Nds5L_159K
MPVFNRWVFVAVAATIISVLDMVDCLRDMRVYVTDCSCIYRDHKLLRVTWADAFDGASLYHYDS
VKRKIVADQAIGQAEADKLNSDSIFIQSLPSQIESLCDKMKQVAESTNTNVDKKAPIPSKVLLQEKSG
QMKLVCLVKNFFPRDIKMSWLRDGVVIVNAPQTVNIVPQRNRTFQARSLLSLNGDVNGSYSCQVE
HETLTEKLVLKF

>great_white_shark_WA_DS5-like
MELCSSLIIIASLTTVTWAEIEVESVSARLYYSYTPDSKRNVDIFNINSIPFLFYDYGAQMFVLNGNVT
DGIKELGQEEVTYYTERARGFNTRLQTITEEIIKLTNAKSIIHKKPVVHIYTKENYTPRESNTLFCYAE
KFYPFEIDITLLINGKSFTGPVNSSQLMVETDWTFNILKYIRIDPQDGDTYSCQVDHMSLDKPLTVLL
DQPPAQPHSGTIVCAVGVIAGVLGLLIGLYLVTKIHNRQGKPCSRPFCKE

>great_white_shark_WA_DS10-like
MSRDFFLFFLLLCPCPGTGNSGLDRWKVSVACFVTDNPSLLEMACELTTNDRILLYYDSTTPSVQFL
VSGFEDYRGIFEELVQDRDRALISLRRTMALTAAVTNHSSPPNEIAQLFLYPEKDVVYGQESILICLV
SGLFPPTIDITLQKNGAPLDAHVNSSKLSFGEDWRFQVLHYAQVRPEAGDTYSCKALHTISREEKIV
YWEPELPNPSEESALDSSQFAVLVLGLIVGLLGTATGICFCLWPILVNRNRLRSRMNSSVRSSSSA

>great_white_shark_WA_Nds3L
MGCPRQNNKVVFFCALPLAWLLGSAIECAEQRVRGMIFSVSDSRTSIVTSTTDDMTLAYHNSTESN
VVFLIEALNALHAESNNLIQKYQSYLTFNEILLNEMANRIPEKQNDDPTVLLYPGEPFTLGGKNTLN
CFVSHLFPPVAHVSFLKNDQPLSGQVRSSQFTFDNSWMFQILKYVQIEPAVGDKYSCVVELVTKEQ
FRAYWEATTSGNHKNPVQIAICAVGFVIGGLGMMTGLCLIFYKWGNE
VGSRDQQTIPAEEPQDSMLNGEGAQDEGTRVDRSDPNQPAEC

>great_white_shark_WB_DS1-like
MIGVFVLLVSVLFQTLGAHMLQAQFSIEKSHHAAVSLRFAYDGTEVIQFNYTTNQFMATEPLAKSF
AEQLNSDQKKVRRYIRYGSFANLVSDVVLRAAHQLITKPSVNITAHRLHSGKDPLHLICNVNGFYP
RGINATWLCNGDAFEQEVLRSRILPNTDGTFQVLFQISIAPRSSDTYTCQVEHSSSPDKLTAIWAPKM
KSWPTYGYVTGIVFMIFGIIALAGGISRWRGLHACGSQPEPNQSQVNTHSDAYSSNTDLGVWTQGE
ANHLYVESERTPSPIPN

>great_white_shark_WB_DS3-like
MPPLRNVIPHMAISAAALYFWCCVSLISALGVVQEVLDCDRAAVSQKDVTGCFCIVAYNKNILTRF
DTKAGRLTVENLTVKNEVDSLNKDPRALKIIQDGIQDTVNFLSLLLEAGADTLDREVSPSVTISFHD
LEDFDHSSQLQCTVAGFYPSALNVTWLKNDGNIDEQVVQTQILPNSEKAFQTAAFTNIDPKTEDTY
TCFVEHVTFPDGLRTNWVPQPKSSLSAAAIVGILFGIGGILTAITGAVFRMKRQGYQPRIPGLKVLF
WKNQQQQKSNRSQASMHSNTSASSATSGDGLTKDTV

>great_white_shark_WB_Nds5L
MPVFYRWIFVAVTAAVVSILDEVDGSSDLHIFHVQCECIYHDNKPANFSWQDGYDGITILYYDFTN
KTFVAVQSIAQTEVDRRNSNTDYVASVPRRIHSFCNKIKQTAITSNLTMEKMAPTSSRVFLQEKAGQ

RDLVCLVKNFFPRNIKVSWLRNGVAIVNGQETTNIAPQDDSTFQARSILTLNEGVGGSYFCQVEHEA
LTRKLLVPLEHDRITKNEALIIVGAVLGILGISFAVMTGILYCTLYRADQFNVHLTAKFTNRAGPCQ
MNPCNSSMRSSSSNTSNSSNTSCYDDLTKSRA

>whale_shark_WA_DS5-like
VSAVTESVYYVHTAHSENILDIFSINYVPFLFYNYGAQTFVINGQVTDGIRELGQEEVDYYHERARG
FQARLRGTTREMIHLTNFTAITKKKPSVHIYMEQNGGPGHSDILYCYAEKFYPFGIELNFLVNGRPF
RGQVNSSQLVVEPDWTFNILKYIRMERGSGDTYTCRVDHISLQQPLTVSL

>whale_shark_WA_DS10-like
RWNMQFGCFAADDPRFPELVCELQVEGRPFIYYDSSLPDIQFLAPGFEDYRAILTEFTQDRDRTVAS
LRRALAIAAVVTNHSNSPNEVGEIFLYPERLVEWGQPNVLVCLVTGLFPPSVAVSLHLDGAPLGSD
VNSSRIFFGEGWRFQALWYAHIRPGPGHLYSCVVRHNISREEKVVFW

>whale_shark_WB_DS1-like
KPSVNIRTHRMTGSSEPLLLSCHVNGFYPRDINISWLLNGAALEQADTSTLLPNADGTFQLMCHIGI
VPRNGDTYTCQVEHSSSSDKLTADW

>whale_shark_WB_DS3-like
ALQQLLDCDHEAVAREDLSGCTLSVAYNRQTLTHYDARARHFSVEDSTVKNEVDNLNKDPKFLEN
IDKILQTTLTLLHQLLESADPVLDRK

>zebra_bullhead_shark_WA_DS5-like
MELCGFLIIIASLTTVTRAAIEFESVSARLYYSNTLDLERNIDIFNINSIPFLFYDYGALMFVLNGKMT
DGVEELGQEEVTYFKERARGFQARLQIITEEMIKLTNAKRIISKKPAVHIYTKENYTPHQSNTLYCY
AEKFYPFEIDITFLINGRRFTGLVNSSQTVVEQDWTFNILKYIRIDPHDGDTYSCEVAHMSLEKPLTV
LLDQPPALPYTGTIVCAVGVIAGILGLVITLYLVTKIYKRRGKLCSDQLCKQ

>zebra_bullhead_shark_WA_DS10-like
SKLFPSLLLPSKLFPSLLLPSNLFPSLLLPSNLFPSLLLPSNPFPSLLLLPSKLFPFTLLPSKLFPSILLLSIL
LLLPISPMSALEKGKVQLACFVTDDPSLMDLACELKTEDRIFIYYDTTLSSVQFQVSGFEQYRGILAE
LVQQSSRTIASQRRMMDITIALTNNSPPPNEIAEVFLYPEKAVAFGETNVLTCLVNGLFPPTINITLQK
NEEPLDGDVNSSKLSFGDDWRFQVLRYAQIQPAAGDLYSCKVVHTISNEEMITYWEPGILDPSEETT
DSDSSQLACFIC

>zebra_bullhead_shark_WB_DS1-like
MSGGAENRLNIRGFILLSWGLTDTLGIHLIQLLFSCDGPHPPVSSLRFAYDGEDYIKFNYTTDKFVAA
NPLAQPFVNSFNSDAKMVRKYVRYGYYGPLVAEAVLQVAKTLTAKPSIAITAHRLSGGNGHLLLN
CRIGGFYPRAINTTWLQDGGTVEQEVLKSRILPNKDGTFQVTLQISIDPGRGDSYTCQVEHRSAPEK
LTAVWVPKRKNVLLTHGKVIGLILMVVGVIIAAIGAFIAWKGRHASGSQPGPNQGQANAHLDAYG
CNTNSGVCSQAEHLCEEIERSPSPTPAENEPSNSEGPPIFGDQVASVYVLAGLAESDSCRCPASRVEP
LAL

>zebra_bullhead_shark_WB_Nds5L_1
MSVFYSWVFVAVSVLDVVDSSSDLHLFHVQCECIYRDNKLADLSWQDAYDGATVMYYDTADKT
FVAVQPIARAEVDRRNSNTDYTESVPRLIEGLCDKIKQTAVTSNFTLEKMAPTFTRAFIEKKGSRSNL
VCLVKSFFPSDIKVSWARNGVTVNGSEITNILPQRDGTFQARSILTLSGDVDASYSCQVEHETIRGKL
IVLLERNRIAENEALIIVGAVLGILGISAAVVTGILYCCILNRGNQISVHPTAKFTNRARPCGVNPCNA
SVRSSTSNSSNSSSSSADGLTKSHA

>zebra_bullhead_shark_WB_Nds5L_2

MSVFYRWVFVAVSVLDVVDSSSDIHLFHVQCECVYDDGGLANIFLQYAYDGATIMFYDMGTRRY
VAVQPFAQAEVDRRNSQLDYVVSVPRRIESVCDEINQTAISSNLNLDGKAPTFTRAFIEKKGSRSNL
VCLVKSFFPSDIKVSWARNGVTVNGSEITNILPQRDGTFQARSILTLSGDVDASYSCQVEHETIRGKL
IVLLERNRIAENEALIIVGAVLGILGISAAVVTGILYCCILNRGNQISVHPTAKFTNRARPCGVNPCNA
SVRSSTSNSSNSSSSSADGLTKSHA

>zebra_bullhead_shark_WB_Nds5L_3
MSVFYRWVLVAVAVTAASVLDVVESSSELRLYQTRCGCVYRDQKFLNVFWEDAFDGTTIFYYDA
EKKQLVSAQPIIQAEVNRRNSDPNYIESVPFFIENLCEKTKQAAVASNTTLEKIAPTFTRAFIEKKAGR
SNLVCLVKFFSPSDIKVSWARNGVTVNGSEITNILPQRDGTFQARSILTLSGDVDASYSCQVEHETIR
GKLIVLLERNRIAENEALIIVGAVLGILGISAAVVTGILYCCILNRGNQISVHPTAKFTNRARPCGVNP
CNASVRSSTSNSSNSSSSSADGLTKSHA

>spiny_dogfish_WA_DS5-like
EFKTISERLYYSYSSDSETNGHIFDINASPFLFYDYKMHTFVIHGNVTNGIEELGQEEVTHFKERASGI
QAWQQGITKEMTKLTNGTSVLKRKPSVHIYTQESYTPRPSNTLYCYAEKFYPFEIDINFLINGRRFTG
PVHSSQLVVEADWTFNILKYIRIDPQDGDTYSCQAAHISLDKPLTVLLDQPPLMPYSGTIVCAVGVV
AGVIGLAITLYLMTKIYSRRGKFCTGQFCKQ

>spiny_dogfish_WA_DS10-like
MHDMIALTSNSQPPNEIGEVFLYSEKPVVFGQTNVLTCWVTGLFPPAVNATLYRNGEALASEVNSS
RLSFGEDWRFQTLRYAEIQPAAGDIYSCKVVHGTGKEEMVTYWELDAQ

>spiny_dogfish_WB_DS1-like-1
MVVASRARALLWIAGLGLGLLCSEARALHTGQLMFSCDGPQPSELILRFAYDGEELARFNYTTKKF
VATIPLVAPFINQLNSNPKDIKFFDRYSHYAPFLAETILQATQTLTAKPSITITSQRLNGGKNPLLLNC
RVDGFYPRDINTTWLRNGDAVQQEVLKSRIFPNTDGTFQLTLQVTVDPGRGDSYTCQLEHRSAPDK
LTAVWAPKRKNLAAYGYVVGIILGIAGIVIAVSGGI

>spiny_dogfish_WB_DS1-like-2
MDLASRARALLWIAGLGLGLLCSEARALHTGQLMFSCDGPQPSELILRFAYDGEELARFNYTTKKF
VATIPLVAPFINQLNSNPKDIKNTDRYSHYAPFLAETILQATQTLTAKPSITITSQRLNGGKNPLLLNC
RVDGFYPRDINTTWLRNGDAVQQEVLKSRIFPNTDGTFQLTLQVTVDPGRGDSYTCQLEHRSAPEQ
LTAVWAPKRKT

>spiny_dogfish_WB_DS1-like-3
MEVASRARALLWIAGLGLGLLCSEARALHTGQLMFSCDGPQPSELILRFAYDGEELARFNYTTKKF
VATIPLVAPFINQLNSNPKDIKNTDRYSHYAPFLAETILQATQTLTAKPSITITSQRLNGGKNPLLLNC
RVDGFYPRDINTTWLRNGDAVQQEVLKSRIFPNTDGTFQLTLQVTVDPGRGDSYTCQLEHRSAPDK
LTAVWAPKRKNLAAYGYVVGIILGIAGIVIAVSGGIIRWKVRYASGSQPGPNQSQANAHSDACVSD
TNSGVWSQDEAEQLCAEIGQSPSPAPADVTFPYNDHIHNTHQYLETG

>spiny_dogfish_WB_DS3-like-1
MLLLCNIIHKSPILLAAFCFYIGPSRISALDVVQQLLDCDLAAVLRKDVSGCLWSVAYNAKTLTRFE
TRTGRLELGNPVVKSEVNSLNKDKKALQNIQNEMQETVDFLSRLLHAGTATLDXQVPPSVTIDFHD
LEVHGHPSQLQCTLTGFY

>spiny_dogfish_WB_DS3-like-2
MLLLCNFIHKSPILLAAFCFYIGPSRISALDVVQQLLDCDLAAVLRKDVSGCLWSVAYNAKMLTRF
ETRTGRLEVGNPVVKSEVNSLNKDKKALQNIQNEMQETVDFLSRLLHAGTATLDXQVPPSVTIDFH
DLEVHGHPSQLQCTL

>spiny_dogfish_WB_DS3-like-3
MLLLCNIIHKSPILLAAFCFYIGPSRISALDVVQQLLDCDLAAVLRKDVSGCLWSVAYNAKTLTRFE
TRTGRLELGNPVVKSEVNSLNKDKKALQNIQNEMQETVDFLSRLLHAGNATLDRKVPPSVTIDFHD
P

>little_skate_WA_DS5-like
MGLRGLLTALLSLAAAVAEIEIETVSEHFVYGRSTSGLEKTLDILSVNSVPFLAYDHEARAFSINGQA
TDGREELGEEEVTYFVQRAEGFQNQIRSDMSELAAGNDNQPIGSKKPIAHIYTEEDYVLQRANTLY
CFAERFYPFEIELQFLVNGQPFTGPVHSSPLLVERDWTFNILKYIRIEPQRGDTFSCLVAHVSLDKAL
TLSL

>little_skate_WA_DS10-like
KTTLALTFFATDDPSLPDGVLEAKADGRSLGYYDTTLSKAQFHLSNLESLQPLVEELIERNIDSTLGL
VHSLLTQVMALTNNSLPSNEIGEALLYSEKPDVLGEENTLICFVSGFFPPAIEVALQKNGKPIVGEVN
SSHLSFSEDWRFYTLQYTHIQPTEGDIYSCKVFHNISKEERVTYWEPEVQEGKEAELDMSQLIVLICG
VIIGILGAAMGFSLCFWLRFTRQDSWNIRINPFVGVSHSS

>little_skate_WB_DS1-like
AGEDSSLAVLQFAFDGEEFIRFEYSSNTFVAKHPLAEPYAIELNKNERVIRNVVRYIRRWPMISHIVK
GAIRAETAKPAITITSRRPNAQGHPLVLTCRVDGFYPMDINTTWLRNGKDIDQEVLRSNILPNFDGTF
QMRLQLSVDPSGIGDTYSCHITHTSVPDNLTA

>Japanese_grenadier_anchovy_WA_01
MGRPSPLNVCFSVFLFLQRTTAWKDYEVVSILSYTRYTSRDPMEQGVVVLVNEAVFAYFNSTQKTF
QLRPSASAGFSVLEASKRMDCVSEVTKAFPRQEDYLERLIEQTNGAKPPKVSPSVNVYSHFPAMPG
TPNYLYCYATGFYPGDIEISFLLNGRPFPGPTETSDLVYGEDWTFKVFKYIRILPHPAEEYACLVNHS
SFAQPKTTVWRLQVSKTTGASYLWTGLVAAAFGATLGCFLSVFMCKRINRRK

>Japanese_grenadier_anchovy_WB_01
MIPLFCLIFLGLSSTHGKDEFTYQQHIGCTFNRHGHVGRFWRYGFNTKDIMHVDLENEAVVSTSDE
GNFMADERKSKLYFKRKEARLNMICSAVQTVFWQSNNSLSRAAKPTVRVSMEVQEGQEYLACSV
QGFYPIAISVHWVYRGKIVHFGNTKTGLLPHKDGTFQMTSYLPLGNKTLQDIVCETEHISIEGKLQA
TFEDESSNLGYIVGIALVSFLLSCLAPLGITALILFMRKRGPQSSVNESLDQSSDGDIAATVTLMALT
GENVQAEPNPEV

>Atlantic_herring_WA_01
MGKILALNVLFSVLLLSQNMMAWTDYEAVSIIAYTRLTSRDSVEQGVLVLVNEAVFAYFNSTQKTF
LLRPSATAGFSVLEASERMYCVSEVTNAFPRQKDYLDRLIKQTNRAKPPKLSPSVNVYSHVPAMEA
SPNYLYCYATGFYPGDIEISFLLNGRPFLGPTESSDLVYGQDWTFRVFKYISILPHPGEEYAFLVNHS
SIAQPKITYWRPEVSKTTGASVLLTAVVAVVAVATGGTLGCFISLLIWRRLIVQAK

>Atlantic_herring_WB_01
MIIQFCAILLGLSSLYARDEFTYQQYIGCAFNFQGPIGHFWRYGYNSKDIMHVDLGKEAVVSTSDEG
SFMAKERQGKDYFIGKEIRLKKICSAVQTVFSQSNNSLSRAAKPTVRVSLREMEGQEYLVCAVQGF
YPNTIRVRWVYGGQIVYFGTTTTGLLPHRDGTFQMTSYLTLGNKTRQDIVCETEHISIEGKLQATLE
DEYSNLGFLVGIAFMSFLMACLIPLGVTAIIVCMKKNRTQGSVNDSLNRSSDCDIPATVSLMNLEGA
QSPVQ

>sardine_WA_01

MGSSIVALYLLVSLLQFSQNMAWTDYEAVNILAYTRLTSRDSMEQGVLVLVNEAVFAYFNATEKS
FLLRPTAMAGFSVLEAKERMYCVSEVIKAFPRQQDYLDKLIKQTNVAKPPKLSPSVNVYSHSPAMP
GSPNYLYCYATGFYPGDIEISFSLNGHPFPGPAESSDLVYGEDWTFRVFKYISILPLPGEVYECFVNH
SSLAQPKRNVWQPEVSKTIGASVWLTAVVAAACGGTLGCFMSVFVWRWRNVTKFLIYFCSIWTNK
APFNLQKRHF

>allis_shad_WA_01
MGSILALHLLVLLLQFSQNMAWTDYEAVSILAYTRLTSRDSVEQGVLVLVNEAVFAYFNATEKTFL
LRPTAMAGFSVLEAKERMYCVSEVINAFPRQQDYLDKLIKQTNGAKPPKLSPSVNVYSHFPAMPGS
PNYLYCYATGFYPGDIEISFVLNGRPFPGPTESSDLVYGEDWTFRVFKYISILPLPGEVYECFVNHSSL
AQPKITVWRPEVSKTIGASVWLTAVVAAACGGALGCFMSVFVWRQRNVTK

>allis_shad_WB_01
MIIQLCAILLGLSSLNARDEFTYQQYIGCAFNRQGPVGRFWRYGFNTKDIMQVDLKNEAVVAVSDE
GNFMAEERQSKVYFKDKEYKLMRICSAVNTVFLQSNNSLSKDAKPTVRVSLEESEGKEYIMCSVQ
GFSPNTISVRWVYKGKIVHFARTTTGLLPRKDGTFQITSYLTLGNKTLQDIVCETEHISIEGTLQATL
DDKYSMGILVGVGILSFFLACLTPVGVTAFISCMKRRPQSSLNDSLEQSSDNSVNPASVSLMDIEPQ
ADQDPVA

>alewife_WB_01
MIIQLCAILLGLSSLNARDEFTYQQYIGCAFNRQGPVGRFWRYGFNTKDIMQVDLKNEAVVAVSDE
GNFMAEERQSKVYFKDKEYKLMRICSAVNTVFLQSNNSLSKDAKPTVRVSLEELEGKEYIMCSVQ
GFSPNTISVRWVYKGKIVHFARTTTGLLPRKDGTFQITSYLTLGNKTLQDIVCETEHISIEGTLQATL
DDKYSMGILVGVGILSFFLACLTPVGVTAFISCMKRRPQSSLNDSLEQSSDNSVNPASVSLMDIEPQ
AEQDPVA

>Hilsa_shad_WA_01
MGRILALNVLVSALLFPQNMAWKDYETASILVYTRLTNKDSVEQGVLVLVNDAVFAYFNSSQETF
LLRPSGMSFSVLGYEDSKYCLLQVTDSLPRQQDYLKKLISQANGAKPAKLSPSVTVYSQMSGSLRY
LYCYATGFYPGDIEISFLLNGHPFPGPRESSDLMYGEDWTFRVFKYIVIHPHPGAVYSCMVNHSSLV
QPKITIWRPEVWKTTGAAMWWIPAVAAGVGAALGCLISVCVWRKEGRAWTP

> Hilsa_shad_WB_01
MIIRLCALLLGLSSLYARDEFTYQQYIGCASNRQGPVKRFWRYGFNAKDIIDVDFKTETVVAVSDEG
NFMAEERQSKVYFKHKELKLARICSAVEAVFLKSNNSLSRTAKPSVRVSLEELDGKEYLACSVQGF
SPNTISVRWVYRGNIVYFAGTTTGLLPHKDGTFQMISYLSPENRTLQDIVCETEHISFEGKLQVTLEH
KNSTGFLVALSILSFLLPFLTPVVVTALLSRKRKRTHSSVNDSLEQSSDNNVHPASVGLMDLESEPE

>oriental_weatherfish_WA
PRQEKYVKKLKEETGSKPRFARPSIQVYTEFPEEKGKANTFYCYATHFYPGDIEMNLFVNGQIMKG
ETSDLTYGKDWTFRVYKYVTITPEPGDEYICEVKHSSMAEPKVIMWRPDFFEYVSHPYWAYALSLG
ILLGITVCV

>oriental_weatherfish_WB
AKPTVFLSSGGNKEQEYLKCVVHGFYPNVIRVRWSWTQTGRHIYYGVSTTGILPHRDGKFQITSFLS
LANISDHSVTCEIEHLSISGKLSIPYEKKEFTEHLPLAVTCFLLGFALFTCIPLLIRCIWQHTRKQPIDER
NDTSADSETSINLMNVSQET

>spined_loach_WA

TPSASAGFSVLEKRDSTFCLTEVIKGFPRQEKYVKKLKEETRSNPRLARPSIQVYTEFPEEMGKANTF
YCYATHFYPGDIEMNLFVNGQIVKGETSDLTYGKDWTFRVYTYVAITPEPGDEYMCEVKHSSIAEP
KVTMWRPDFSESVSHPYWAYALPGILLGITVSVLILRKWHSQL

>white_sucker_WA_01
MADILTVRAFVLILLLFPQTITMWTDYHTVNVLAYTRMTGNGSMDQAVVVLVNETVFAYFDQAK
NTFVLRPSATAGFSVLETRDSKFCLWEVLKGFTRQVEYLKKFREEARSSKPLLARPSVNMYTQFPE
EQGKTNMLYCYATEFYPGDIEMNFFLNGVMVKGETSDLMYKKDWTFRVYKYMNITPTPGDEYIC
EVKHSSMTEPKLTVWRPELSESHPYWAYTLPVGVLLGTIVSILIFRKTRNTQS

>white_sucker_WB_01
FLLGVALLDIVLGAEEHAYQQFIECAFNSEAQDEQLWSYGYDGKDIMHVDLEKEAVVATSEHGQR
LAEARKSKEYIKRKEEKLQKVCSAVKTVFFASNNSLSKAAKPAVYVSSGGEKKQEYLKCVVHGFY
PNTIRVQWTHNGRSIFYGVSTTGILPLIDGTFQMTSFLSLDKVNAHDVTCEIEHSSINGKLRKTYEEK
SSLSQITETFLAVLTFLLGFVLPV

>common_carp_WA_A1
MEVIQAFILILLLSPQITMWTDYQTANILVYTRMMESGSIDQAVVVLVNEATFAYFDQAKKTFVLRP
SASAGFSVLEGSDQSFCVYEVLQGFYRQTGNLEKLKQETNSSKALLERPSVNVYTEFPEEQGKVNV
LYCYATGFYPGDIEINFYLNGQKSTVKAETSDLMYGEDWTFKVYKYMNITPQYGDVYTCEVRHSS
LAEPKMTEWRPEFSASTSHLYWAYVLPLGILLGIMVSVLILRRKQRSQI

>common_carp_WA_A2_01
MEIIQTFVIILLLSPQITMWTDYQTVNVLAYTRMMENGSIDQAVVVLVNEATFAYFDQAKNTFVLR
PSASAGFSVLEGSDRSFCMYEVLAGFYRQTDYLEKLKQETNSSKSLSVRPSVNVYTEFPEQQGKVN
VLYCYATGFYPGDIEINFYLNGQKSTVKAETSDLMYGEDWTFRVYKYMNITPQYGDKYTCEVRHS
SMVQPKMTEWRPEISVSTSHLYWAYSLSLSILLGIMVSVLILRRKCRSQL

>common_carp_WB_B1_01
MQVFLFVVAFFTVLEAEEHAYQQFIGCAFNNEGQVSRFWRYGYDGRDIMHVDLAKEAVVATSEP
GQLLAEERKSKEYIKRKEARLVKVCSAVKTVFLKSNNSLSRAAKPAVYVSSGGEKDQEYIKCAVR
GFYPNIIKVRWTHKARPIYFGVSTTGVLPHKDGTFQMTSYLSLSNVSSSDVTCEIEHLSIDGKMRINY
GDKLLFLSQITEHVLKAVAAFLLGFVLPVCITVLFICIRQNTSKPPEDEIKDTSDESEASLSLNLMNIS
QET

>common_carp_WB_B2
MQVFLLIMAFLNTILGAEEHAYQQFIGCAFNNEGQVSRFWRYGYDGRDIMHVDLTKEAVVATSEP
GQLLAEERKSKEYIKRKEARLVKVCSAVKTVFLKSNNSLSRAAKPAVYVSSGGEKGQEYLKCAVH
GFYPNIIRVRWTQNRKPVYFGVSTTGVLPHKDGTFQMISYLSLSNVSVQGVTCEIEHLSIDGILRIAY
EEKSLLLSQITDVLKVVAAFLLGFALPVCLTVLFMCIGLRQQTTKPPEDVIKDTSDESEASLSLNLMN
ISQET

>Chinese_cavefish_Sg_WA_A1_01
MANMEIIQTFILILLLSPQIIMWTDYQTANILAYTRTMENGSIDQALVVLVNEATFAYFDQAKNTFV
LRPSASAGFSVLEDSDRSFCMNEVLAGFYRQTNYLEKLKQETNSSKALLVRPSVNMYTEFPEEQGK
VNVLYCYATGFYPGDIEINFYLNGQKSTVKAETSDLMYGEDWTFKVYKYMNITPQYGNEYTCEVR
HSSLAEPKMTEWRPEFSASTSHLYWAYALPLGILLGIMVSVLVLRRQHCSQL

>Chinese_cavefish_Sr_WA_A1_01
MANMEIIQTFTLILLLSPQIIMWTDYQTANILAYTRTMENGSIDQALVVLVNEATFAYFDQAKNTFV
LRPSASAGFSVLEDSDRSFCMNEVLAGFYRQTNYLEKLKQETNSSKALLVRPSVNVYTEFPEEQGK

VNVLYCYATGFYPGDIEINFYLNGQKSTMKAETSDLMYGEDWTFKVYKYMNITPQYGDEYTCEVR
HSSLAEPKMTEWRPEFSASTSHLYWAYALPLGILLGIMVSVLILRRQQRSQL

>Chinese_cavefish_Sa_WA_A1_01
MTNMEIIQTFILILLLSPQIIMWTDYQTANILAYTRTMENGSIDQALVVLVNEATFAYFDQAKNTFVL
RPSASAGFSVLEDSDRSFCMNEVLAGFYRQTNYLEKLKQETNSSKALLVRPSVNVYTEFPEEQGKV
NVLYCYATGFYPGDIEINFYLNGQRSTVKAETSDLMYGEDWTFKVYKYMNITPQYGDEYTCEVRH
SSLAEPKMTEWRPEFSASTSHLYWAYALPLGILLGIMVSVLILRRQQRSQL

>Chinese_cavefish_Sr_WA_A2_01
MEIIQTLVLILLLSPQITMWTDYQTVNVLAYTRMMENGSIDQAVVVLVNEATFAYFDQAKNTFVLR
PSASAGFSVLEGSDRSFCMYEVLAGFYRQTDYLEKLKQETNSAKSLLVRPSVNVYTEFPEQQGKVN
VLYCYATGFYPGDIEVNFYLNGQKSTVKAETSDLMYGEDWTFRVYKYMNITPQYGDKYTCEVRH
SSMVEPKMTEWRPEFSVSTSHLYWAYSLPLSILLGIMVSVLILRRKCRSQL

>Chinese_cavefish_Sg_WB_B1_01
MQVFLFVMAFFNTILAAEEHAYQQFIGCAFNNEGQVSRFWRYGYDGRDIMHVDLVKEAVVATSES
GQLLAEERKSKEYIKRKEARLVKVCSAVKTVFLKSNNSLSRAAKPAVYYSSGGEKDQYIKCDVRG
FYPNIIKVRWTQKARPIYFGVSTTGILPHKDGTFQMTSYLSLSNVSGHDVTCEIEHLSIDGKMRITYG
EKSLFLSQITEHVLTAMIAFLLGFVLPACLTVLFIFIWQKTSKPPEDEIKDTSDESEASLSLNLMNTSQ
ET

>Chinese_cavefish_Sr_WB_B1_01
MQVFLFVMAFFNTILAAEEHAYQQFIGCAFNNEGQVSRFWRYGYDGRDIMHVDLVKEAVVATSES
GQLLAEERKSKEYIKRKEARLVKVCSAVKTVFLKSNNSLSRAAKPALYVSSGGEKDQEYIKCVVRG
FYPNIIKVRWTQKARPIYFGVSTTGILPHKDGTFQMTSYLSLSNVSGHDVTCEIEHLSIDGKMRITYG
EKSLFLSQITEHVLTAMIAFLLGFVLPACLTVLFIFIWQKTSKPPEDEIKDTSNESEASLSLNLMNTSQ
ET

>Chinese_cavefish_Sa_WB_B1_01
MQVFLFVMAFFNTVLAAEEHAYQQFIGCAFNSEGQVSRFWRYGYDGRDIMHVDLVKEAVVATSK
SGQLLAEERKSKEYIKRKEARLVKVCSAVKTVFLKSNNSLSRAAKPALYVSSGGEKDQEYIKCVMR
GFYPNIIKVRWTQKARPIYFGVSTTGILPHKDGTFQMTSYLSLSNVSGHDVTCEIEHLSIDGKMRITY
GEKSLFLSQITEHVLTAMIAFLLGFVLPACLNVLFIFIWQKTSKPPEDEIKDTSDESEASLSLNLTNTS
QET

>Chinese_cavefish_Sg_WB_B2_01
MYVFLLIVAFLNTTVGAEEHAYQQFIGCAFNNEGQVSRFWRYGYDGRDIMHVDLAKEAVVATSEP
GQLLAEERKSKEYIKRKEARLVKVCSAVKTVFLKSNNSLSRAAKPAVYVSSGGEKDQEYLKCAVH
GFYPNIIRVRWTQNRRPVYFGVSTTGVLPHKDGTFQMISYLSLSNVSAQSVTCEIEHLSIDGILRIAYE
EKSLLLSQITEIVLKAVAAFLLGFALPVCLTVLFICIRQQTTKPPEDEIKDTSDESEASLALNLMNISQE
T

>Chinese_cavefish_Sr_WB_B2_01
MHVFLLIMAFFNTTLGAEEHAYQQFIGCAFNNEGQVSRFWRYGYDGRDIMHVDLAKEAVVATSEP
GQLLAEERKSKEYIKRKEARLVKVCSAVKTVFLKSNNSLSRAAKPAVYVSSGGEKDQEYLKCAVH
GFYPNIIRVRWTQNRRPVYFGVSTTGVLPHKDGTFQMISYLSLSNVSAQSVTCEIEHLSIDGILRIAYE
EKSLLLSQITEIVLKAVAAFFLGFALPVCLTVLFICIRQQTTKPPEDEIKDTSDESEASLSLNLMNISQE
T

>Chinese_cavefish_Sa_WB_B2_01

MHVFLLIMAFFNTTLGAEHAYQQFIGCAFNNEGQVSRFWRYGYDGRDIMHVDLAKEAVVATSEPG
QLLAEERKSKEYIKRKEARLVKVCSAVKTVFLKSNNSLSRAAKPAVYVSSGGEKDQEYLKCAVHG
FYPNIIRVRWTQNRRPVYFGVSTTGVLPHKDGTFQMISYLSLSNVSAQSVTCEIEHLSIDGILRIAYEE
KSLLLSQITEIVLKAVAAFLLGFALPVCLTVLFLCIRQQTTKPPEDEIKNTSDESEASLALNLMNISQE
T

>goldfish_WA_01
MANMEIIQTFILILLLPPQITMWTEYQTVNVLAYTRMMENGTIDQAVVVLVNEATFAYFDQPKNTF
VLRPSASAGFSVLENGDQSFCMNEVLGGFYRQTDYLEKLKQETNSSKALLERPSVNVYTKFTEEQG
KVNVLYCYATGFYPGDIEINFYLNSQKSTVKAETSDLMYGEDWTFKVYKYMNITPQYGDVYTCEV
RHSSLAEPKMTEWRPEFAASTSHPYWAYALLLGILLGIMVSVLILRREQHSQL

>goldfish_WB_01
MLVFLFVVAFFNTILAAEEHAYQQFIGCAFNNEGQVSRFWRYGYDGRDIMHVDLAKEAVVATSEP
GQLLADERKSKEYINRKEARLVKVCSAVKTVFLKSNNSLSRAAKPAVYVSSGGEKDQQYLKCAVR
GFYPNVIKVRWTQKGKPIYFGVSTTGILPHKDGTFQMISYLSLRNVSGHDVTCEIEHLSIDGKMRITY
GEKSLFLSQITEHVLKAVAAFLLGFVLPVCITVLIICIRQKTSKPPEDEIQDTSDESEASLSLNLMNISQ
ET

>catla_WA_01
MANMEIIQTFVLILLLSPRITMWTDYQTVNVLAYTRMMENGSIDQAVVVLVNEATFAYFDQANKT
FVLRPSASAGFSVLEGNDQSFCMYEVLAGFYRQTDYLEKLKQETNSSKSLLVRPSVNVYTEFPEQQ
GKANILYCYATGFYPGDIEINFYLNDQKSGVKAETSDLMYGEDWTFRVYKYMNITPWYGDKYTCE
VRHSSMPEPKMTEWRPEFSASTSHLYWAYSLPPGILLGIMVSVLILRRKWHS

>catla_WB_01
MLVFLLTMAFFNTILGAEHAYQQFIGCAFNNEGQVSRFWRYGYDGRDIMYVDLAKEAVVATSEPG
QLLAEERKSKEYIKRKEARLVKVCSAVKTVFLKSNNSLSTAAKPAVYVSSGGEKNQEYLKCVVHG
FYPNVIKVRWTKNRRPIYFGVSTTGILPHKDGTFQMTSYLSLSNVSTHDVTCEIEHLSIDGKLKIDYG
EKSLFLSQITEHVLKAVAAFLFGFVLPVCLTLLFICIRQKTRKAPENEITDTSDESEASLSLNLMNISQ
ET

>tench_WA_01
MANTETFQTFVMMILLLSPQITMWTDYQTVNVLAYTRMRENGSIDQAVVVLVNEATFAYFDQEK
KTFVLRPSASAGFSVLKGSERSFCMYEVLAGFYRQTNYLEKLKEETKSSKALLVRPSVNVYTEFPEE
EGKANVLYCYATGFYPGDIEINFFLKGHKSIVKVETSDLMYSKDWTYRVYKYTTITPQTGDEYTCE
VRHRSMAEPTMKAWRPKFSAPTSHLYWVYSLPLGILLGIMTSVLILRRKQRSQL

>tench_WB
EHAYQQFIGCSFNNEGQEGRFWLYGYDGRDIMHVDLVKEAVIATSEPGQLLAEERKSKAYIKRKES
RLVKLCSAVKTVFLKSNNSLSRAAKPAVYVSSGGEQDQEYLKCVVHGFYPNVIRVRWTQKGRPVY
FGVSSTGILPHRDGTFQMTSYLSLINMSAHGVTCEIEHLSLGGILRKTYEAKSQITEHVLWAMAAFL
LGFALPVFLTLLFRKRQQTTKPPEDKTKDTSDRSEASLSLNLMNMSQET

>grass_carp_WA_01
MANMEILPVRISVLTLLLYPQIITMWTDYYTVNVLAYTRMMGNGSMDQTVVVLVNDSIFAYFDQE
NKTFALRPSASAGFSVLEKRDSIFCLGEVTKGFYRQAEYLDKLKEETNSSKPLLVRPSVSVYTEFPEE
EGKANVLYCYATGFYPGDIEINLFLNGRKSIVKVETSDLIYSKDWTFRVYKYMTITPRTGDEYTCEV
RHSSMAEPKIKTWRPEFSASTSHLYWACSLPLGILLGIMTSVLILRRKQRSLL

>grass_carp_WB_01

MLAFFLIVTFHEIILGAEEHAYQQFIECAFNSQGQVDHTWNYGYDGKDIMHVDLVKKTVIATSEPG
KMLEEERKHIEYIKRKEEKLKMVCSAVKTVFLKSNNTLSRAAKPAVLLSAGGEQDQEYLKCVVHG
FYPNVIRVRWTQKGRPVYFGVSSTGILPHRDGTFQMTSYLSLNNMNADGVTCEIEHLSLDGILRKT
YEEKSQITEHVLWATVAFFLGFALPVFLTVIFILIRKQTTEPPEDTSDGSKASLSLNLMNMSQET

>fathead_minnow_WA_01
MSDMKILTVRASVLTLLLYPQIITMWTDYHTVNVLAYTRMMGNGSMDQAVVVLVNDSIFAYFDQ
ENKTFALRPSASAGFSVLEKRDSIFCLGEVTKGFYRQAEYLEKLKEETNSSKPLLVRPSVNVYTEFPE
EEGKANVLYCYATGFYPGDIEINFFQNGHKSIVKVETSDLMYSKDWTFRVYKYMIITPRTGDEYTC
EVRHSSMAEPTIKAWRPEFSAAHTPHLYWACSLPLGVLLGIMTSVLILRRKWNSQL

>fathead_minnow_WB_01
MLAFLLIVAFHEIILGADEHAYQQFIECAFDSQGQVDHTWSYGYDGKDIMHVDFVKKAVVATSEPG
KMLEEERKHAEYIKKKEERLKMVCSAVKTVFLKSNNSLSRAAKPAVVLSAGGEQDQKYLKCVVH
GFYPNVIRVRWTRKGRPVYFGVSSTGILPHRDGTFQMTSYLSLINMSAHDITCETQHLSLDGILRKT
YEEKSQIIEHVLWAMAAFFPFLAGFLLPVLPTSLLLIRKRRHMTKPPEDTSDTSEASLSLNLMNMSQ
ET

>roach_minnow_WA
MEILTVRTSVLTLLLYPQIITMWTEYHTVNVLAYTRMMGNGSMDQAVVVLVNDSVFAYFDQENK
TFALRPSASAGFSVLEKRDSIFCLGEVTKGFYRQAEYLEKLKEETNSSKPLLVRPSVNVYTEFPEEEG
KANVLYCYATGFY*********QNGHKSIVRVKTSDLMYSKDWTFRVFKYMTITPRTGDEYTCEVRH
SSMAEPTMKAWRPEFSAAHTSHPYWAYSLPLGILLGFMTSVLILRRTWHSQL

>roach_minnow_WB
RTGYSQWNFEEHAYQQFIECAFDSQGQVDHTWCYGYDGKDIMHVDLVKKAVVATSEPGKMLEEE
RKHMEYIKRKEEKLKMVCSAVKTVFLKSNNSLSRAAKPTVLLSAGGEQDQKYLKCVVHGFYPNVI
RVRWTQKGRPVYFGVSSTGILPHRDGTFQMTSYLSLINMSAHGVTCETEHLSLDGILRKTYEEKSQI
TE

>Amur_ide_WA_01
MEILTVRTSVLTLLLYPQIITMWTDYHTVNVLAYTRMMGNGSMDQAVVVLVNDSVFAYFDQENK
TFALRPSASAGFSVLEKRDSSFCLGEVTKGFYRQAEYLEKLKEETNSSKPLLVRPSVNVYTEFPEEE
GKANVLYCYATGFYPGDIEINFFQNGHKSIVKVKTSDLMYSKDWTFRVYKYMTITPRTGDEYTCEV
RHSSRAEPTMKAWRPEFSAAHTSHLYWAYSLPLGILWGIMTSVLILRRTWHSQL

>Amur_ide_WB_01
MLAFLLIVAFHEIILGAEEHAYQQFIECAFDSQGQVDHTWRYGYDGKDIMHVDLVKKAVVATSEP
GKMLEEERKHMEYIKRKEEKLKMVCSAVKTVFLKSNNSLSRAAKPAVLLSAGGKQDQKYLKCVV
HGFYPNVIRVSWTQKGRPVYFGVSSTGILPHRDGTFQMTSYLSLINMSAHGVTCETEHLSLDGILRK
NYEEKSQITEHVLWAMAAFFLGFTLPVFLTLLFIWKRRHTTKPPEDTSDRSEASLSLNLMNMSQET

>zebrafish_WA_13A
MEVFTVRASLLTLLLYPHIIITWTDYHTVNILAYTRVMGNGSIDQTVVVLVNDAIFAHFDKANNTFA
LNPTASAGFSVLEKRESIFCLGEVNKGFHRQTEYLEKLKKETKSSKTLFVRPSVIMYAEFPEEEGKA
NVLYCYATGFYPGDIDIRFFLNGQKSTAKLETSDLMYGEDWTFRVFKYMKITPQTGDEYTCEVRHS
SMSEPKITVWRPEFSSSTSHPYWAYTTALGVMSGIGTSTLILKRKHCSQL

>zebrafish_WA_01
MEVFTVRASLLTLLLYPHIIITWTDYHTVNILAYTRVMGNGSIDQTVVVLVNDAIFAHFDKANNTFA
LNPTASAGFSVLEKRESIFCLGEVNKGFHRQTEYLEKLKKETKSSKTLFVRPSVSIYAEFPEEEGKAN

VLYCYATGFYPGDIDIRFFLNGQKSTAKLETSDLMYGEDWTFRVFKYMKITPQTGDEYTCEVRHSS
MSEPKITVWRPEFSSSTSHPYWAYTAALGVMLGIGTSTLILKRKHCSQL

>zebrafish_WA_02
LTLLLCPHIIITWTDYHTVNILAYTRVMGNSSIDQTVVVLVNDAIFAHFDKANNTFALNPTASAGFS
VLEKRESIFCLGEVNKGFHRQTEYLEKLKKETKSSKTLFVRPSVSIYAEFPEEEGKANVLYCYATGF
YPGDIDIRFFLNGQKSTAKLETSDLMYGEDWTFRVFKYMKITPQTGDEYTCEVTHSSMSEPKITVW
RPEFSSSTSHPYWAYTTALGVMLGIGTSTLILKRKHCSQL

>zebrafish_WA_03
MEVFTVRASLLTLLLYPHIIITWTDYHTVNILAYTRVMGNGSIDQTVVVLVNDAIFAHFDKANNTFA
LNPTASAGFSVLEKRESIFCLGEVNKGFHRQTEYLEKLKKETKSSKTLFVRPSVIMYAEFPEEEGKA
NVLYCYATGFYPGDIDIRFFLNGQKSTAKLETSDLMYGEDWTFRVFKYMKITPQTGDEYTCEVRHS
SMSEPKITVWRPEFSSSTSHPYWAYTTALGVMLGIGTSTLILKRKHCSQL

>zebrafish_WA_04
LCPHIIITWTDYHTVNILAYTRVMGNGSIDQTVVVLVNDAIFAHFDKANNTFALNPTASAGFSVLEK
RESIFCLGEVNKGFHRQTEYLEKLKKETKSSKTLFVRPSVSIYAEFPEEEGKANVLYCYATGFYPGDI
DIRFFLNGQKSTAKLETSDLMYGEDWTFRVFKHMKITPQTGDEYTCEVRHSSMSEPKITVWRPEFS
SSTSHPYWVYTTALGVMLG

>zebrafish_WA_05
MEVFTVRASLLTLLLYPHIIITWTDYHTVNILAYTRVMGNGSIDQTVVVLVNDAIFAHFDKANNTFA
LNPTASAGFSVLEKRESIFCLGEVNKGFHRQTEYLEKLKKETKSSKTLFVRPSVIMYAEFPEEEEKA
NVLYCYATGFYPGDIDIRFFLNGQKSTAKLQTSDLMYGEDWTFRVFKHMKITPQTGDEYTCEVRHS
SMSEPKITVWRPEFSSSTSHPYWAYTAALGVMLGIGTSTLILKRKHCSQL

>zebrafish_WB_13B
MLAFLLIVSFHEVILGADEHAFQQFIECAFNSQGQVDRSWRYGYDGKDVMHVDLATETVVGTSEP
GKRLAEERKSTEYIRRKEEKLKIVCSAVKTVFLKSNNTLSRAAKPTVLLLSNGGQGQEYLKCVVRG
FYPNVIRVRWTQKGKPIFFGVSTTGTLPHTDGTFQMTSYLSLGNMTAHGVTCEIEHLSIDGKLRKNY
GDNPWILSQITVAVVAFILGFVCTIAVIFVWKRHQTTKSHDDETNGASDESEASLSLNVMNISQET

>zebrafish_WB_01
MLTFLLIVSFHEVILGADEHAFQQFIECAFNSQGQVDRSWRYGYDGKDVMHVDLATETVVGTSEP
GKRLAEERKSTEYIRRKEEKLKIVCSAVKTVFLKSNNTLSRAAKPTVLLLSNGGQGQEYLKCVVRG
FYPNVIRVRWTQKGKPIFFGVSTTGTLPHTDGTFQMTSYLSLGNMTAHGVTCEIEHLSIDGKLRKNY
GDNPWFLSQITVAVVAFILGFVCTTAVIFVWKRHQTTKSHDDETNGASDESEASLSLNVINISQET

>zebrafish_WB_02
MLAFLLIVSFHEVILGADEHAFQQFIECAFNSQGQVDRSWRYGYDGKDVMHVDLATETVVGTSEP
GKRLAEERKSTEYIRRKEEKLKIVCSAVKTVFLKSNNTLSRAAKPTVLLLSNGGQGQEYLKCVVRG
FYPNVIRVRWTQKGKPIFFGVSTTGTLPHTDGTFQMTSYLSLGNMTAHGVTCEIEHLSIDGKLRKNY
GDNPWILSQITVAVVAFILGFVCTIAVIFVWKRHQTTKSHDDETNGASDESEASLSLNVMNISQET

>zebrafish_WB_03
EHAFQQFIECAFNSQGQVDRSWRYGYDGKDVMHVDLATETVVGTSEPGKRLAEERKSTEYIRRKE
EKLKIVCSAVKTVFLKSNNTLSRAAKPAVLLFSNGDQGQEYLKCVVRGFYPNVIRVRWTQKGRPV
YFGVSTTGILPHTDGTFQMTSYLSLGNMTAHGVTCEIEHLSIDGKLRKNYRDNPWFRSQITVAV

>zebrafish_WB_04

EHAFQQFIECAFNSQGQVDRSWRYGYDGKDVMHVDLASETVVGTSEPGKRLAEERKSTEYIRRKE
EKLKIVCSAVKTVFLKSNNTLSRAAKPAVLLFSNGDQGQEYLKCVVRGFYPNVIRVRWTQKGRPV
YFGVSTTGILPHTDGTFQMTSYLSLGNMTAHGVTCEIEHLSIDGKLRKNYRDNPWFRSQITVAVVA
FILGFALPMCPTAVMFVRKRHYQTTKSHDDETNGASDESEASLSLSVMNISQET

>zebrafish_WB_05
MLAFLLIVSFHEVILGADEHAFQQFIECAFNSQGQVDRSWRYGYDGKDVMHVDLATETVVGTSEP
GKRLAEERKSTEYIRRKEEKLKIVCSAVKTVFLKSNNTLSRAAKPTVLLLSNGGQGQEYLKCVVRG
FYPNVIRVRWTQKGKPIFFGVSTTGTLPHTDGTFQMTSYLSLGNMTAHGVTCEIEHLSIDGKLRKNY
GDNPWFLSQITVAVVAFILGFVCTTAVIFVWKRHQTTKSHDDETNGASDESEASLSLNVMNISQET

>brown_ghost_knifefish_WA_01
MMSLKTFMLVLLKIPLIMMWTDYQMVNVLAYTRMRSNGSVDQGVVVLVNDAIFAYFDQKIKTFV
LRPSASAGFSALEYSDRSFCMNEVLRGFYRQQEYLSKLKEHAHVATSPFLRPSVNVYVQFPVVEGQ
ANVLYCYATGFYPGDIEITFFHNGRMSEVEGVLSDLMYGDNWTFRIYKYMVISPQPGDEYVCQVK
HSSMAEPKVSEWRPEFPPYTPASFWAFGLASGVILGFVISGLLIR

>brown_ghost_knifefish_WB_01
MAGIMMFLLFACVLNAWSSIGADENAYQQFIGCAFNSTGQVGHFWRYGYNSKDIMHIDLVKETM
VSTSNAGRFLAEERKSVEYIHSKQERLRKVCSAVQTVFLLSNSSLSRAAKPTVHVRVQGEQGKGFL
MCQVHGFYPNVIRVQWTRQGKSVYYGVSTAGILPHKDGTFQMTSYLSLGNSSAHGVTCNVEHISF
GGKMRATLEERTILLLSTAVTVGAFLAGLVLPVGTVVVVIRFTRKRAPIQNEDVSNEHSEDSGTPPS
MGLLL

>black_ghost_WA_01
MMSLKTFMLVLLKVPIIMMWTDYQMVNVLAYTRMRSNGSVDQGVVVLVNDAIFAYFDQKNKTF
VLRPSASAGFSALENTDRSFCLNEVLRGFYRQQEYLSKLKEHADVATPPFLRPSVNVYVQFPVAEG
QANVLYCYATGFYPGDIEITFFHNRRMSEVKGVLSDLMYGDKWTFRIYKYMVISPQPGDEYVCQV
KHSSMAEPKVSEWRPEFPPYTPTSFWAFGLASGIILGFVISGLLIRNV

>black_ghost_WB_01
MAGIMMFLLFACVLNAWSSIGTDENAYQQFIGCAFNSTGQVGHFWRYGYNSKNIMHIDLVNETMV
STSNAGHFLAEERKSVEYIHSKQERLRKVCSAVQTVFLLSNSSLSRAAKPAVHVRVQGEQGKEFLM
CQVNRFYPSVIRVQWTHQGESVYYGVSTTGILPHKDGTFQMTSYLSLGNLSAHGVTCNVEHISFAG
KMRATLEERSILLLSTGVTVGSFLAGLVLPIGTVVVVIRFTRKRAPIQNEDASNEHSEDSGTPPSMSL
LL

>duckbill_knifefish_WA
TFMLVLLKVPIIMMWTDYQMVNVLAYTRMRSNGSVDQGVVVLVNDAIFAYFDQKNKTFVLRPSA
SAGFSALEYSDRSFCMNEVLRGFYRQQEYLSKLKEHADVATSPSLRPSVNVYVQFPVAEGQANVL
YCYATGFYPGDIEITFFHNGRMSEVEGVLSDLMYGDNWTFRIYKYMVISPQPGDEYVCQVKHSSM
AKPKVSEWRPEFPPYTPTSFWAFGLASGIILGFVISGLLIRNV

>duckbill_knifefish_WB
RFLAEERKSVKYIHSKQERLMKVCSAVQTVFLLSNSSLSRAAKPAVHVRVQGEQGKEFLMCQVHG
FYPNVIRVQWTRQGKSVYYGVSTTGILPHKDGTFQMTSYLSLGNSSAHGVTCNVEHISFEGKMRAT
L

>glass_knifefish_WA
YQTVNILAYTRMRSNGSVDQGVVVLVNDASFAYFDQTNKTFVLRPSASTGFSVLEDKDRSFCMYE
VLRGFYRQQEYMSKLKMYTDVATPPFVRPSVNVYAQFPVAEGQANILYCFATGFYPADIEITFFING

RLSVAEGVTSDLMYGDEWTFRIYKYMAISPRPGEEYACQVKHSSMAEPKDSVWRPEFPASTLGSL
WAFGMASGLICGIVISGLLVRRF

>Mexican_tetra_WA_01
MSRLTFILTLLQVPAVLLWTDYQTVNIQAYTRIRSNGSVDQGVVVLVNDAIFAYFDSGNKTFVLRPS
ATMGFSVLGESDRTFCLYEVLHGFYRQKDYLSKLREKTGGTKPLLVRPSVNVYPQFPVTEGQANV
LYCYATGFYPGDIEINFFLNGRPSAEDGIMSDLMYSEDWTYRVYKYMAITPRPGEEYSCEVKHSSM
AEPKMSLWRPEFPAFTPGFSWIFGLIPGLVCGIIVSAVLFRRKLHFQL

>Mexican_tetra_WB_01
MADIMLFLMLVCALIVQPSLCADENAYQQFIGCAFNSQGQADHLWRYGYNGVDVMHIDLQRETM
VSTSEAGHFLTEERKSVTYIKSKEERLKKVCSAVQTVFSLSNSSLSRAVKPSVHVRMGGEAGREFLR
CLVHGFYPNIIRVQWNRNGRPIFFGTSTTGILPHKDGMFQMTSYLSLGNTSTHGVTCEVEHISIDGK
MKATLETNPIHLLFSVVIAVVAGLAGCICPVGITTLIIYLRNKTASKPLNTTNDSSEASETPPSMSLMH
LPSEI

>tambaqui_WA
MSLKSFVLMLLQAPAVLLWTDYQTVNIQAYTRMKGNGSVDQAVVVLVNDATFAYFDKAKKTFV
LRPSATTGFSVLDDSDQTFCMYEVLHGFHRQEEYLSKLREQTGAVRPPLVRPSVNVYAQFPVAEGQ
ANVLYCYATGFYPGDIEINFFLNGHPSVDKGITSDLMYGDDWTYRVYKYMAITPRPGEEYTCEVEH
SSMAEPKMSTWSEYVMINSTV

>tambaqui_WB_01
MAATLTSLLLLYVLNARPSLGADENAYQQFIGCAFNSRGQVGHFWRYGYNSKDIMYIDLVREAMV
STSETGHFLTEERKSVEYIKSKEKRLRKLCTAVQTVFSLSNNSLSRAVKPAVHVKLREEKGREFLLC
LVHGFYPNVIRVRWTRDGKNIYYGTSTTGVLPHKDGTFQMTNYLSLSNTSVHGVTCEIEHISIDGK
MRATLEETPSLLLSVVLAAAAFFAGIAFPVGTVTLIIRLRRKQKPKDTTNDSSEASTPPSVSLMHLQA
ES

>African_coelacanth_WA_1_01
MELLRTWPFWGFLWGPLLVTGVEEDGDFFGMHVLTADMDSVSFGVAVVVSYNDESIAHWDQEK
EDTVMEIPGIQHHMTELFNLLINNRERAYHFHEEVKTFVYPHVPKPPNVMPDIYLYMENPITFGKKN
RLNCYAMNFFPPNLRAVLLENENPLLVKKVAIWDLTFNLGGNLQLLLSVEVVPKLRDSYSCTMEH
LKLRQNRTIVLDAQEPYKPAADHSETAALAIGIIVGIIGMLVGIVLIAKH

>African_coelacanth_WB_1_01
MPLAGKGLVLWVVLLSVLFFSQLPNVQGVFNYLQHQFFCAEDSSTNASFCRWLVSYDSRKLFYLD
SRVNRFVPLQQFLEKKMEEKNSNKLTFQQYNSICQLACEYKEKVLASAKATLNRKVPPMVNIGIM
KDEEIQTDTLSLICSIRGFYPQAISTTWLRNGEDIKSETFTTGLLPNVDKTFSLDLYVDIVESDWNEYT
CQVEHSGLTEPLHLTLDRKQPGKSGIIIGAVFAAVGVIVMVTGLIVRIRRNGSDAAETISINSISEMVP
MS

>West_African_lungfish_WA_2_01
MATFQGFGLLLWLLFWINMADATFRFRKLEVLPFTTLDMKTIQMQSLAILVNDVPIGLFDQHENKF
LIRLNISEEACNKMKQLAVLYMREAKGNMDWQKVLIRMMETKQKVHPPLEPPSVSMYSEKPYVP
GQNNTIYCYATGFYPPEIDIVFFENGNHFAGKVYASDIAFSEDWRFKIMKFIHVVPQQTTLYSCKVN
HISFKQPRELTLGNSMDHHARVPVLFIVSVPCALIGIPLGIYFLKDSRLSSFR

>West_African_lungfish_WB_2_1_01
MLCSCCGIALGLVLFSLLQNNACGGDHVREAMNTLQFRMGCSMDQGKMKYFWQRGYNQKNIKR
YDYTEQRAIMEHECVKDEVEADNRNPILRKSRQETAEKFCQKARSIFDELPVHLGELGNNSVSISLE

NQGKENYLVCFVRGFHPSNIKVTWLKDGKEELYGVTTTGVLPLRDGTFVMKMYLFLADTTKGLYI
CQVEHESLKGKIRVLWEQKSEVQSFPDSIIWITFILALLLILLLACISKIQKDHQTATTKIPEDRKEMD
KLCSSSMGVGNEEE

>South_American_lungfish_WA_1_01
MCGKILNSLSAIILWSSALLLLAEKDTFFFDAYLFSSDNDVSKNNFQLSVSVNGLELMQWDVTNQT
FLEQIGGMEEVMPFLNSVVRLNIKGTMALHQHIFQVMSEETHSAPAPNELPDIYSYFEKPIKFGEKN
NLSCFAVRFYPPSIQINILKNNVVIPSEIKPSGAMFDINWKFQMLKSAEVTPMKGDIYSCSVEHLILK
QTRNVTIEPKEPYIPQVDHSETIVCAAGTIVGITGLLLGIFLIRKQHTNRDVM

>South_American_lungfish_WB_1_01
MFTNRYACQWQKFYLSFLIILIFAFQCELHFLHHHVHCTSFNTGNKTLSWEVAYDSLTIAHVDNEN
VGIAATSPYVHEEIESFNRRKDLIKRYNNIMHDVWPFLDLIASLGNSTLDRKVAPSVTIVVDPVDHGI
LNLACYIHGFYPQNIEVFWLQNGEDITEKMATTDILPNTDNTFSLITYVDAEENNKHKYTCMVKHS
SVTNGISATVDFNQGFGIGYYVGGVLALVGIIAGMTGIILRYRLKALRVCMQRRNLGPAETVHRTQ
PTRQELEQLDGSPDSLHENVFSSSNYEAAYLTIQDIPVIGLTSDIPLDQSTSTTVE

>Australian_lungfish_WA_1
MRSALCVVWSLVLMSVAEEDGYVFDVYFITADNNSNAPVIHSSVAVNGIEIIRWDEKKQKVLQQIG
GLEELMPFVNNLLYGNIRGALKEHNEVVKLMTEATHNSVPPNELPDIYLYFEKPIKFGEKNNLSCFII
RFFPLAVQIEILRNNISIPGEIKSFRLMFDDDWKFQILKSMEVVPRAGDIYSCSVEHLILRQKKLVTLG
KLIWAYNYV

>Australian_lungfish_WB_2_2
MILHKLVLSFLCILLHRKDACGEATSTFQYRAGCVLEKGRNTYFWRSSYNNKDIEHYDYERQCLVA
DYPSAKDRAEEYNKSPSFPMYWNNLITDLCSSAKKLFDELPAELGRLERSSVSVSLETKEKDKYLE
CLVHGFHPSYINVTWLRDGKEQFYGVTSSGILPLKDGTFHIRKYLPIGDSVGGSYACQVEHESLVEA
ILVHWDPVRKTSFLEKILGIPVLIIGVCFPIFGYIYKRKFPKRGRKETSSISTSSLEGNPQAAPLNPEEN
TD

>Chinese_salamander_WA_01
MNSLRTIAWMCLLAANSCQQGIKDLFLEAFTSTGSPGASNLTVALLADTVVAAYYSGTNGTFQFPP
SQLEALGRFAANQIITNQTVAFHQHSMDTLCQRSNCSDPVFVFPDVQFYPEIPVVLGQENRLLCFLK
EFFPPEVSVAFLKNGKPFRGQIQSSELTFGRNWTFQVLKSITVEPGAGDTYSCTVEHGNRQSHRDLD
FEKLPMTVENNAHIVVLAVGLAVGILGFAMGLLLFLQYSTRNPYMQWLNRNSVSS

>Chinese_salamander_WB_01
MFSHTWIPLLHIISSISTADAHIFQQTLGCSTRSASFPEITECWWRAAYNGEEVWKFDLLNSTSVYFS
PLMVEDERLFLHHFRNSILPSGVDIVASLQSFMNQPARETVTPNVTVSLADTEVGAPLKSLCCRVSE
FYPPDLNVTWSLDGSTLALGNGLKEPVILPNSDGTFQTTSCIPFNSSHPQGQHYSCTVQHLSTLLGL
VAKWEAPPLDNRMVKAEMVIGILAGLVGIVFLSSALIYHGCIREGRISSCCKSEDRGMEISEIDTVSE
G

>Hokkaido_salamander_WA_01
MNSLRTIAWICLLAANSCQQGIKDLFLEAFTSTGSPGASNLTVALLADTVVAAYYSGTNGTFQFPPS
QLEALGRLAANQIITNKTVAFHQHSMETLCQRSNCSDPVFVFPDVQFYPEIPVVLGQENRLLCFLKE
FFPPEVSVAFLKNGKPFMGQIQSSELTFGRNWTFQVLKSITVEPGAGDTYSCTVEHGNRQSRRDLAC
GKLPMTVENNAHIVVLAVGLAVGILGFAMGLLLFLQYSTRNPYMQWLNRNSVSS

>Hokkaido_salamander_WB_01

MFSLTWIPLLHIISSRSTADAHIFQQTLGCSTRSASFPEITECWWRAAYNGEEVWKFDLLNSTSVYFS
PLMVEDERLLLRHFRNSILPPGVDIVASLQSFMNQPARETVTPNVTVSLVNTEVGAPQKSLCCRVSE
FYPPDLNVTWSLDGSTLALGNGLKEPVILPNSDGTFQTTSCIPFNSSHPQGKRYSCTVQHLSTPLGLV
AKWELPPSENRMVKAEMVIGILAGLAGIIFLTSALIYHGCIRKGRISSCCKSEDRGMEISEIDTASEGA
SASA

>tiger_salamander_WA_01
MNSLPASLCMCILTVVRCQGGSKNIQLAAFTSTDSPAASNLTLALLADTVVAAYYDGSTDTFQIPDT
GLKDTVQTFAHFFPMESVSNFHQMVMKDMCMKINCSDPVSVFPEVQFYIEAPVVLGQENRLLCFL
KGFFPPEVRVSFLKNEQPFPGQMQSSELIFGRNWTFQVLKYILVKPQAEDTYSCIVEHGYLQSRQNL
TWGRPVSLENKTHVTVLIVGLTVGFLGFVVGLILCIHGKNLKCLARNPYQR

>tiger_salamander_WA_02
MNSLPAYLCMCILTVVRCQEGSKNIQLAAFTSTDSPAASNLTLDLLADTVVAAYYDGRTNTFQIPD
TGLKDTVQTFAHFFPMERVSNFHQMVMKDMCMKINCSDPVSVFPEVQFYIEAPVVLGQENRLLCF
LKGFFPPEARVSFLKNGQPFPGQMQSSELIFGRNWTFQVLKYILVKPQDEDTYSCIVEHGYLQSRQN
LTWGRPVSLENKTHVTVLIVGLTVGFLGFVVGLILCIHGKNLKCLASNPYQR

>tiger_salamander_WB_01
MPPLAWITILLALCCQFTAGSQIFQQLIECSKSPSSPATPQCWWRAAYNREQLWDFNLLNSNIDLLL
LDLNRRFGLPLEYLLRELQYPLQNTPFAANVFMSLTAKEIVAPRVTINAEETEVGAPLYTLCCRATG
FYPPNINVTWFLDGSPLAHDHGMKELVILPNSNGTFQTTSCMPFSISHTQAKNYLCAVQHISTPEGL
NATWEAPGLEEHKAELAIGILAGLAGVFFASSALVWHGCCKKGRITSCWKHEGLMVEISEMAAAS
EAASPSV

>tiger_salamander_WB_02
MPPLAWITILLALSCPFTAGSQIFQQLIECSKSPSSPATPQCWWRAAYNREQLWDFNLLNSNIDLLLL
DLNRRFGLPLVYLLRELEYPLQNTPFAANVFMSLTAKEIVAPHVTINAEETEVGAPLYTLCCRATGF
YPPNINVTWFMDGSPLAHDHGMKELVILPNSNGTFQTTSCMPFSISHTQAKNYLCAVQHISTPEGLK
ATWEAPGLEEHKAELAIGILAGLAGIFFSSSALVWHGCCKKGRVTSCWKHEGLMVEISEMAAASEA
ASPSV

>axolotl_WA
MNRLPASLCMCILTVVRCQEGSKNIQLAAFTSTDSPAASNLALALLADTVVAAYYDGSTDTFQIPD
TGLKDTVQTFANFFPMERVSNFHQMVMKDMCMKINCSDPVSVFPEVQFYIEAPVVLGQENRLLCF
LKGFFPPEVRVSFLKNEQPFPGQMQSSELIFGRNWTFQVLKYILVKPQAEDTYSCIVEHGYLQSRQN
LTWGRPVSLENKTHITVLIVGLTVGFLGFVVGLILCIHGKNLKCLASNRYQR

>axolotl_WB_01
MPPLAWITILLALSCPFTAGSQIFQQLIECSKSPSSPATPQCWWRAAYNREQLWDFNLLNSNIDLLLL
DLNRRFGLPLEYLLRELQYPLQNTPFAANVFMSLTAKEIVAPHVTINAEETEVGAPLYTLCCRATGF
YPPNINVTWFLDGSPLAHDHGMKELVILPNSNGTFQTTSYMPFSISHTQAKNYLCAVQHISTPEGLN
ATWEAPGLEEHKAELAIGILAGLAGIFFASSALVWHGCCKKGRITSCWKHEGLMVEISEMAAASEA
ASPSV
